# Design and synthesis of novel cytotoxic fluoroquinolone analogs through topoisomerase inhibition, cell cycle arrest, and apoptosis

**DOI:** 10.1038/s41598-023-30885-5

**Published:** 2023-03-13

**Authors:** Mohamed A. Elanany, Essam Eldin A. Osman, Ehab Mohamed Gedawy, Sahar M. Abou-Seri

**Affiliations:** 1 Department of Pharmaceutical Chemistry, School of Pharmacy and Pharmaceutical Industries, Badr University in Cairo (BUC), Badr City, 11829 Cairo Egypt; 2 Department of Pharmaceutical Chemistry, Faculty of Pharmacy, Cairo University, Cairo, 11562 Egypt; 3 Department of Pharmaceutical Organic Chemistry, Faculty of Pharmacy, Cairo University, Cairo, 11562 Egypt

**Keywords:** Cancer, Medicinal chemistry, Drug discovery and development, Screening

## Abstract

To exploit the advantageous properties of approved drugs to hasten anticancer drug discovery, we designed and synthesized a series of fluoroquinolone (FQ) analogs via functionalization of the acid hydrazides of moxifloxacin, ofloxacin, and ciprofloxacin. Under the NCI-60 Human Tumor Cell Line Screening Assay, (**IIIf**) was the most potent among moxifloxacin derivatives, whereas (**VIb**) was the only ofloxacin derivative with significant effects and ciprofloxacin derivatives were devoid of activity. (**IIIf**) and (**VIb**) were further selected for five-dose evaluation, where they showed potent growth inhibition with a mean GI_50_ of 1.78 and 1.45 µM, respectively. (**VIb**) elicited a more potent effect reaching sub-micromolar level on many cell lines, including MDA-MB-468 and MCF-7 breast cancer cell lines (GI_50_ = 0.41 and 0.42 µM, respectively), NSCLC cell line HOP-92 (GI_50_ = 0.50 µM) and CNS cell lines SNB-19 and U-251 (GI_50_ = 0.51 and 0.61 µM, respectively). (**IIIf**) and (**VIb**) arrested MCF-7 cells at G1/S and G1, respectively, and induced apoptosis mainly through the intrinsic pathway as shown by the increased ratio of Bax/Bcl-2 and caspase-9 with a lesser activation of the extrinsic pathway through caspase-8. Both compounds inhibited topoisomerase (Topo) with preferential activity on type II over type I and (**VIb**) was marginally more potent than (**IIIf**). Docking study suggests that (**IIIf**) and (**VIb**) bind differently to Topo II compared to etoposide. (**IIIf**) and (**VIb**) possess high potential for oral absorption, low CNS permeability and low binding to plasma proteins as suggested by in silico ADME calculations. Collectively, (**IIIf**) and (**VIb**) represent excellent lead molecules for the development of cytotoxic agents from quinolone scaffolds.

## Introduction

**Figure 1:**
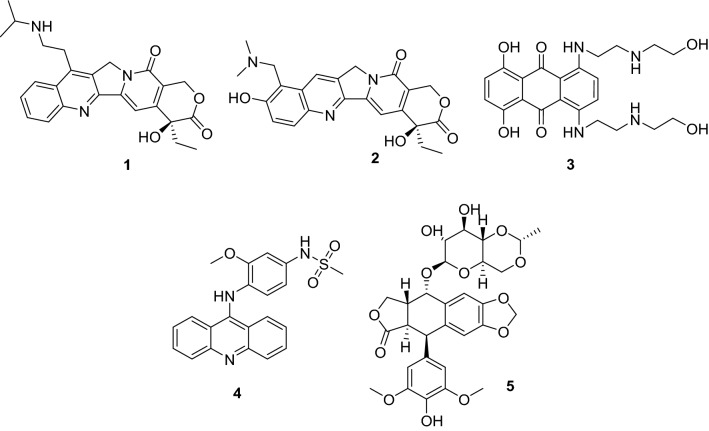
Chemical structures of selected clinically approved Topoisomerase I & II inhibitors (**1–5**).Generated using ChemDraw 2021 (https://perkinelmerinformatics.com/products/research/chemdraw)^19^.

**Figure 2:**
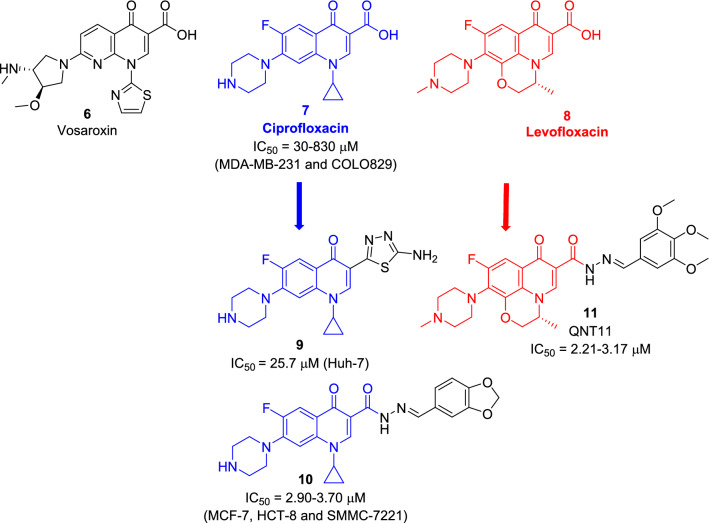
Chemical structures of vosaroxin (**6**) and some reported FQ-based Topo II inhibitors (**9–11**). Generated using ChemDraw 2021 (https://perkinelmerinformatics.com/products/research/chemdraw)^19^.

Cancer therapy has shown a successful decrease in mortality over the past decade^1,2^. However, statistics show slow progress in some types of cancer such as breast and colon, and a lack of progress in other types as seen in prostate cancer^3^. One of the successful strategies in cancer treatment is to utilize the highly elevated rate of cell proliferation of the tumor cells characterized by an abundance of DNA-managing enzymes such as topoisomerase (Topo) and helicase^4^. Topo is a class of tyrosine kinase enzymes responsible for managing the topology of DNA via relaxing and supercoiling as the need arises^5,6^. It cleaves the phosphate backbone of one strand (Topo I) or both strands (Topo II) of DNA followed by unwinding and resealing the backbone again^7^. Several Topo I inhibitors show promise in established therapeutic regimens exemplified by belotecan (**1**) for the treatment of non-small cell lung cancer (NSCLC) and ovarian cancer, and topotecan (**2**) which is a second-line therapy for metastatic ovarian and small-cell lung cancer, including recurrent cases^8–10^. On the other hand, mitoxantrone (**3**), amascarine (**4**), and etoposide (**5**) are effective Topo II inhibitors currently in use against several cancers such as leukemia, lymphoma, prostate, and breast cancers (Fig. 1)^11–16^. The naphthyridone derivative vosaroxin (**6**, voreloxin) is another example of potent Topo II inhibitors currently ongoing clinical trials in acute myeloid leukemia with broad-spectrum anti-tumor activity (Fig. 2)^17,18^

The promising properties of vosaroxin (**6**) nudged the research focus into using the antibacterial fluoroquinolones (FQs) in cancer therapy, based on the mechanistic similarities and sequence homologies between bacterial and eukaryotic topoisomerases and the established favorable pharmacokinetic and pharmacodynamic profiles of those FQs^20–22^. The concept of anticancer FQs gained strong momentum with the emergence of scientific reports describing the cytotoxic properties and apoptotic effects of the clinically approved FQs (Fig. 2). Ciprofloxacin (**7**, Fig. 2), a 2nd generation and one of the most widely used FQs, was found to inhibit the growth of melanoma (COLO829) and breast (MDA-MB-231) cells while inducing apoptosis and S cell cycle arrest^23^. Moreover, the screening of 3rd generation levofloxacin (**8**, Fig. 2) against multiple cell lines showed its inhibitory action on viability as well as G2/M cell arrest^24,25^. Consequently, optimizations were deployed, mainly through *C*3 or *C*7 derivatization, resulting in derivatives with increased potency^26^. While both approaches provided more potent cytotoxic agents, *C*3 derivatization seems to shift the activity towards human topoisomerase since it eliminates the carboxylic acid antibacterial pharmacophoric moiety^27^. FQs featuring fused heterocyclic ring as a bioisostere of the carboxylic acid moiety showed higher cytotoxic activity and excellent water solubility as seen with the 1,3,4-thiadiazole ciprofloxacin derivative (**9**) that inhibited the growth of hepatocellular carcinoma Huh-7 cell line^28,29^. Other examples include the piperonal ciprofloxacin hydrazone derivative (**10**), which showed cytotoxic and apoptotic effects on MCF-7, HCT-8 and SMMC-7721 cell lines through Topo II inhibition and the trimethoxy-benzaldehyde levofloxacin hydrazone (**11**) derivative, QNT11, that was reported to inhibit tumor cells selectively via its potent Topo II poisoning and apoptotic induction effects^30,31^.

**Figure 3:**
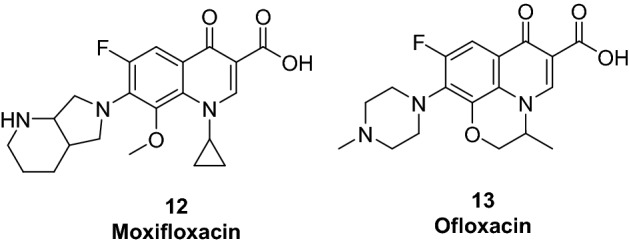
Chemical structures of moxifloxacin (**12**) and ofloxacin (**13**). Generated using ChemDraw 2021 (https://perkinelmerinformatics.com/products/research/chemdraw)^19^.

Exploration of the cytotoxic potential of higher generation FQ scaffolds such as moxifloxacin (**12**, 4th generation FQ, Fig. 3) is an enticing prospect because of their enhanced pharmacokinetic profile as well as their higher safety when compared to earlier generations (ciprofloxacin and ofloxacin) which caused phototoxicity and CNS adverse effects^32,33^. The scientific basis of these allegations is evident upon reviewal of cytotoxicity data of moxifloxacin (**12**, Fig. 3), which showed inhibitory activity against several cell lines such as melanoma (COLO829) and pancreatic (Panc-1 and PaCa-2)^34,35^.

**Figure 4:**
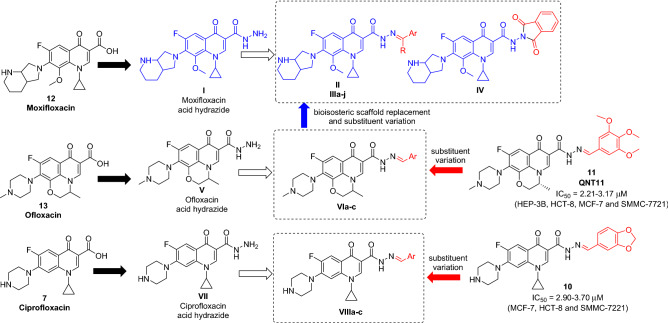
Design and general structure of the newly synthesized derivatives. Generated using ChemDraw 2021 (https://perkinelmerinformatics.com/products/research/chemdraw)^19^.

Encouraged by these findings, a series of moxifloxacin-based derivatives was designed via functionalization of its acid hydrazide to provide the hydrazone derivatives (**II)** and (**IIIa–j**) or phthalimide derivative (**IV)** for exploration of their cytotoxic potential. The design strategy exploited the promising cytotoxicity of *C*3 acid hydrazone derivatives (**10**) and (**11**) through bioisosteric replacement of the FQ scaffold with moxifloxacin and/or substituent variation (Fig. 4). Furthermore, to effectively analyze the effects of the used FQ on cytotoxicity, ciprofloxacin (**7**) and ofloxacin (**13**) analogues bearing the same substitutions were obtained for comparison (Fig. 4).

## Results and discussion

### Chemistry

**Figure 5:**
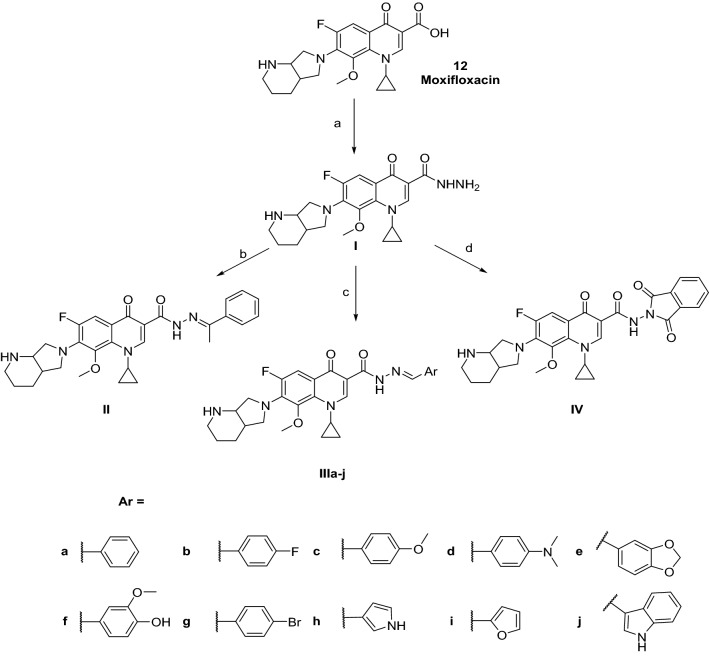
Synthetic pathway of moxifloxacin derivatives. Generated using ChemDraw 2021 (https://perkinelmerinformatics.com/products/research/chemdraw)^19^. Reagents and conditions: (a) NH_2_NH_2_ 80%, reflux, 24 h (61%); (b) C_6_H_5_COCH_3_, ethanol, reflux, 6 h (48%); (c) ArCHO, ethanol, reflux, 6 h (20–88%); (d) phthalic anhydride, acetic acid, reflux, 12 h (80%).

The synthetic route to moxifloxacin derivatives is described in Fig. 5*.* Initially, moxifloxacin (**12**) was directly heated with hydrazine hydrate (80%) to prepare its carbohydrazide (**I**) with a yield of 61% after washing it with water multiple times. The IR spectrum of (**I**) showed a downshift of the carbonyl groups from 1708 to 1666 cm^−1^. Its ^1^H NMR showed the appearance of the D_2_O exchangeable signals of the NH_2_ and NH hydrazide proton at *δ* 3.26–3.41 and 10.76 ppm, respectively. Condensation of (**I**) with acetophenone to yield (**II,** 48%) was performed in absolute ethanol. Its ^1^H NMR spectrum featured the appearance of methyl group singlet peak and phenyl protons at *δ* 2.45 and 7.34–7.89 ppm, respectively. Similarly, successful condensation and purification of various aldehydes to produce (**IIIa–j**, 20–88%) were performed in absolute ethanol. Their ^1^H NMR featured an extra singlet peak of azomethine proton at *δ* 8.06–8.40 ppm besides the appearance of characteristic peaks of the additional aryl moieties. Finally, condensation with phthalic anhydride using glacial acetic acid as solvent yielded (**IV**, 80%).

**Figure 6:**
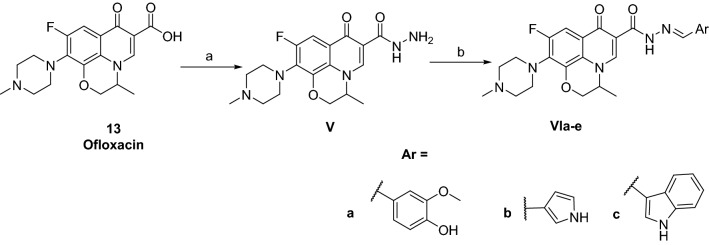
Synthetic pathway of ofloxacin derivatives. Generated using ChemDraw 2021 (https://perkinelmerinformatics.com/products/research/chemdraw)^19^. Reagents and conditions: (a) NH_2_NH_2_ 80%, reflux, 24 h (40%); (b) ArCHO, ethanol, reflux, 6 h (16–41%).

**Figure 7:**
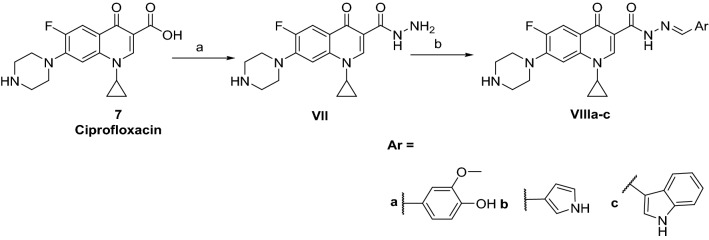
Synthetic pathway of ciprofloxacin derivatives. Generated using ChemDraw 2021 (https://perkinelmerinformatics.com/products/research/chemdraw)^19^. Reagents and conditions: (a) NH_2_NH_2_ 80%, reflux, 24 h (30%); (b) ArCHO, ethanol, reflux, 6 h (23–38%).

The synthetic routes utilizing ciprofloxacin (**7**) and ofloxacin (**13**) as starting materials are shown in Figs. 6 and 7*,* respectively. The hydrazones were obtained successfully by directly heating the acid hydrazides with the respective aldehydes in absolute ethanol. Afterward, the compounds were recrystallized to yield (**VIa–e**) and (**VIIIa–c**).

### Biological evaluation

#### NCI-60 human tumor cell lines screen single-dose assay

**Figure 8:**
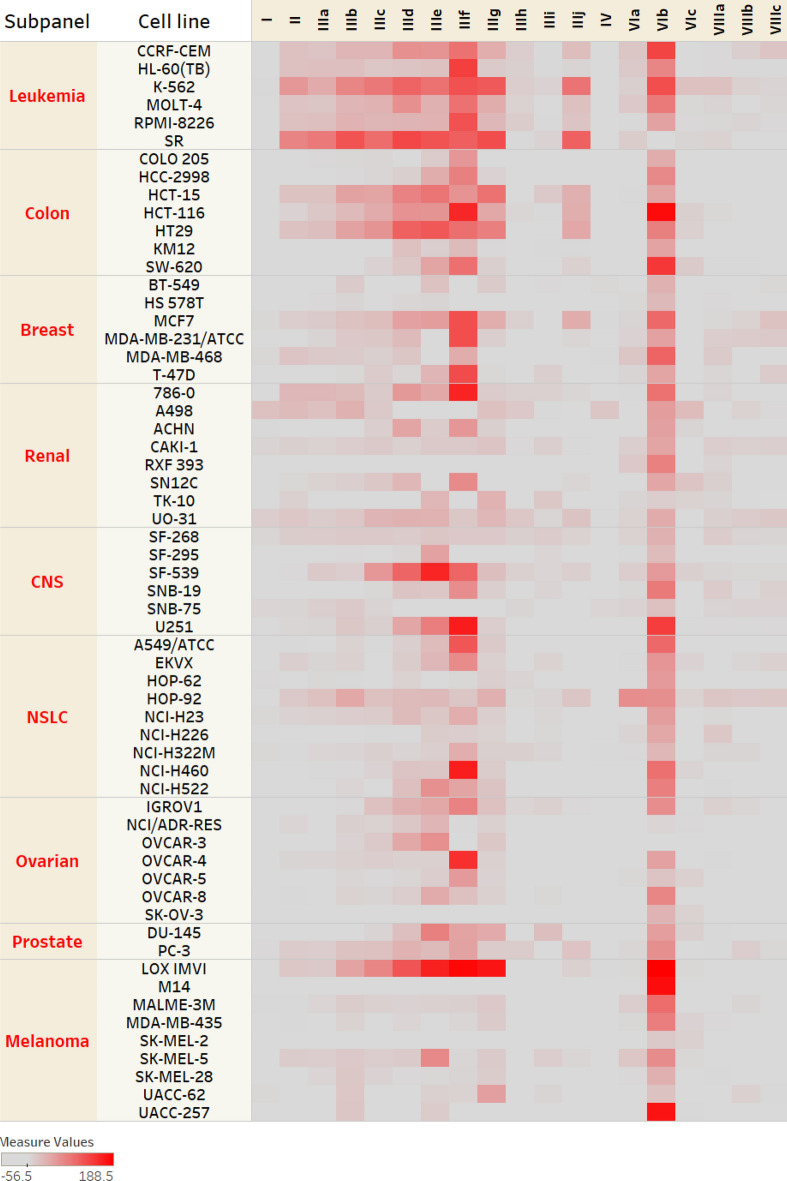
Heat map of the mean growth inhibition % of the tested derivatives in the single dose NCI-60 Human Tumor Cell Lines Screen at 10 µM. Red color intensity corresponds to more potent growth inhibition. Generated using Tableau 2022.3. (2022). (https://www.tableau.com/products/desktop)^36^.

All the synthesized compounds were screened by The Developmental Therapeutic Program (DTP) at the National Cancer Institute, USA (NCI) in the NCI-60 Human Tumor Cell Lines Screen at an initial single dose of 10 µM. For comparative analysis of the single-dose assay data, a graphical heat map was constructed using the percentage growth inhibition of each compound against the respective cell line (Fig. 8). Ciprofloxacin (**7**), moxifloxacin (**12**), and ofloxacin (**13**) data were obtained from the NCI repository for comparison. The mean growth inhibition percent (average of growth inhibition percentage across the whole 60 cell line) for each compound is shown in Table 1, whereas the full growth inhibition data is depicted in the supporting information section (Supplementary Tables 1S and 2S)*.* Examination of moxifloxacin analogs (**I**, **II**, **IIIa–j** and **IV**) revealed that leukemia and colon cancer cell lines were the most sensitive subpanels. In contrast, the melanoma subpanel proved to be the least sensitive. Studying the impact of derivatization of moxifloxacin on cytotoxicity revealed that; modification of the COOH group into a carbohydrazide in compound (**I**) or phthalic anhydride hydrazone (**IV**) decreased the cytotoxic effects significantly. However, replacing the COOH with methyl benzylidene hydrazone (**II**) increased activity by three folds. Furthermore, the removal of the methyl group of azomethine (**IIIa**, Ar = Ph) maintained the cytotoxicity, hinting that this methyl is not essential for cytotoxicity. Substitution on the phenyl ring with either electron-donating groups (**IIIc**, Ar = 4-OCH_3_Ph), **(IIId**, Ar = 4-N(CH_3_)_2_Ph), **(IIIe**, Ar = 1,3-benzodioxol-5-yl), or electron-withdrawing groups (**IIIb**, Ar = 4-FPh), **(IIIg**, Ar = 4-BrPh) increased the cytotoxicity with no particular preference. Finally, replacing the phenyl ring with a heterocyclic ring (**IIIh**, Ar = 3-pyrrolyl), **(IIIi**, Ar = 2-furyl), or a fused heterocyclic system (**IIIj**, Ar = 3-indolyl) decreased the activity greatly. The vanillin hydrazone derivative of moxifloxacin (**IIIf**, Ar = 4-OH-3-OCH_3_Ph) was the most potent moxifloxacin derivative and the first compound promoted for five-dose screening. Its percentage growth inhibition (GI%) ranged from 1.48% and reached lethality up to 183.25% in multiple cell lines such as breast’s MCF-7 and MDA-MB-231/ATCC.

**Table 1:** Mean growth inhibition % of the tested derivatives in the single dose NCI-60 Human Tumor Cell Lines Screen at 10 µM.

Compound	Mean growth inhibition %	Compound	Mean growth inhibition%
**I**	− 2.66%	**IIIj**	5.48%
**II**	7.29%	**IV**	− 6.62%
**IIIa**	8.59%	**VIa**	4.41%
**IIIb**	17.97%	**VIb**	**74.29%**
**IIIc**	17.67%	**VIc**	0.35%
**IIId**	29.15%	**VIIIa**	2.49%
**IIIe**	39.24%	**VIIIb**	0.26%
**IIIf**	**64.43%**	**VIIIc**	-1.65%
**IIIg**	25.87%	**Ciprofloxacin (7)**	-5.05%
**IIIh**	0.03%	**Moxifloxacin (12)**	2.34%
**IIIi**	1.85%	**Ofloxacin (13)**	-2.03%

Significant values are in bold.

Moxifloxacin (NSC:758875), ofloxacin (NSC: 758178), and ciprofloxacin (NSC: 758467) data were obtained from the NCI repository.

**Figure 9:**
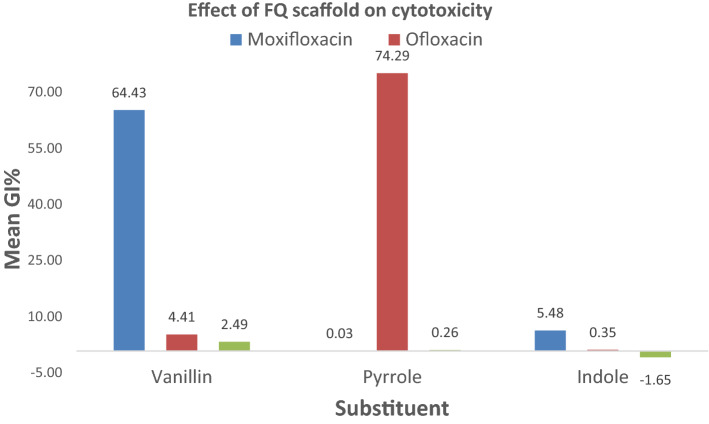
Effect of FQ scaffold on cytotoxicity using identical analogues of moxifloxacin, ofloxacin and ciprofloxacin. Generated using Tableau 2022.3. (2022). (https://www.tableau.com/products/desktop)^36^.

To attain a representative comparative analysis of the scaffold’s effect on cytotoxicity, (**IIIf**,**h**,**j**) counterparts bearing ofloxacin (**VIa–c**) and ciprofloxacin (**VIIIa–c**) nuclei were prepared and screened. Generally, the moxifloxacin scaffold was the most promising among the three nuclei, and ciprofloxacin was the least, with all of its derivatives showing a near lack of cytotoxic effects (Fig. 9). When the vanillin moiety (Ar = 4-OH-3-OCH_3_Ph) was paired with moxifloxacin in (**IIIf**, mean GI% = 64.43%), it exhibited higher cytotoxicity than when conjugated with ofloxacin (**VIa**, mean GI% = 4.41%) and ciprofloxacin **(VIIIa**, mean GI% = 2.49%). Similarly, the same pattern was observed in a less prominent fashion with the indole analogues (Ar = 3-indolyl), showing mean GI% of 5.48, 0.35 and − 1.65% for derivatives of moxifloxacin (**IIIj**), ofloxacin (**VIc**) and ciprofloxacin (**VIIIc**), respectively. Despite this predisposition, in the case of coupling with pyrrole moiety (Ar = 3-pyrrolyl), ofloxacin (**VIb**) was more cytotoxic than moxifloxacin analogue (**IIIh**) and ciprofloxacin analogue (**VIIIb**) with mean GI% of 74.29, 0.03 and 0.26%, respectively. (**VIb**) showed potent growth inhibition (GI% = 5.80–188.5%) and was promoted for five-dose screening. It exhibited lethality in several cases, such as MCF-7.

#### NCI-60 human tumor cell lines screen five-dose assay

The 2nd phase of the NCI-60 Human Tumor Cell Lines Screen involved the evaluation of the most potent compounds (**IIIf**) and (**VIb**) at five doses (100, 10, 1, 0.1, and 0.01 µM) for the determination of half-maximal Growth Inhibition (GI_50_), Total Growth Inhibition (TGI) and half-maximal Lethal Concentration (LC_50_). (**IIIf**) and (**VIb**) showed broad spectrum cytotoxic activity against most of the tested cell lines with an average GI_50_ of 1.78 and 1.45 µM, respectively (Table 2). However, both (**IIIf**) and (**VIb**) exhibited decreased activity against NCI/ADR-RES with GI_50_ of 58.80 and 100 µM, respectively. Individually, (**IIIf**) exhibited an overall homogenous spectrum of activity with GI_50_ ranging from 1.43 to 2.36 µM, with the highest activity on SNB-75, MDA-MB-468, and MCF-7 (GI_50_ = 1.43, 1.63, and 1.67 µM, respectively). Meanwhile, (**VIb**) elicited more potent inhibition (GI_50_ = 0.41–5.40 µM) reaching sub-micromolar GI_50_ against multiple cell lines such as MDA-MB-468, MCF-7, HOP-92, MOLT-4 and SNB-19 (GI_50_ = 0.41, 0.42, 0.50, 0.50 and 0.51 µM, respectively).

**Table 2:** Concentrations for 50% growth inhibition (GI_50_) of compounds (**IIIf**) and (**VIb**) expressed in µM as determined in the NCI-60 Human Tumor Cell Lines Screen five-dose assay.


Cell line	GI_50_ (µM) IIIf	GI_50_ (µM) VIb	Cell line	GI_50_ (µM) IIIf	GI_50_ (µM) VIb
Leukemia			Breast cancer		
CCRF-CEM	2.36	0.96	MCF-7	1.67	0.42
HL-60(TB)	1.79	0.67	MDA-MB-231/ATCC	1.69	1.48
K-562	1.64	0.70	HS 578T	2.06	2.03
MOLT-4	2.30	0.50	BT-549	1.82	2.32
RPMI-8226	1.88	2.19	T-47D	2.35	1.27
SR	1.98	0.84	MDA-MB-468	1.63	0.41
NSCL cancer			Melanoma		
A549/ATCC	1.81	1.55	LOX IMVI	1.78	0.72
EKVX	1.74	1.32	MALME-3M	1.69	1.04
HOP-62	1.77	1.32	M14	1.81	1.67
HOP-92	1.66	0.50	MDA-MB-435	1.78	1.52
NCI-H226	1.95	1.54	SK-MEL-2	1.85	1.57
NCI-H23	1.85	1.44	SK-MEL-28	1.88	1.47
NCI-H322M	1.67	1.62	SK-MEL-5	17.60	1.40
NCI-H460	1.87	0.77	UACC-257	1.71	1.64
NCI-H522	1.79	1.24	UACC-62	1.72	1.70
Colon cancer			Ovarian cancer		
COLO 205	1.76	1.27	IGROV1	1.78	0.94
HCC-2998	1.89	0.52	OVCAR-3	ND	ND
HCT-116	1.92	0.71	OVCAR-4	1.68	1.30
HCT-15	1.82	1.68	OVCAR-5	1.65	1.51
HT29	1.83	1.41	OVCAR-8	1.82	1.64
KM12	1.67	ND	NCI/ADR-RES	**58.8**	**100**
SW-620	1.73	1.30	SK-OV-3	1.87	1.80
CNS cancer			Renal cancer		
SF-268	1.75	1.45	786-0	1.82	1.42
SF-295	1.72	1.54	A498	1.76	2.22
SF-539	1.73	1.28	ACHN	1.70	1.45
SNB-19	1.83	0.51	CAKI-1	1.67	1.54
SNB-75	1.43	1.48	RXF 393	1.57	1.06
U251	1.86	0.61	SN12C	1.83	1.73
Prostate cancer			TK-10	1.68	5.40
PC-3	1.74	1.52	UO-31	1.53	2.71
DU-145	1.86	1.53			

*ND* not determined.

Structures Generated using ChemDraw 2021 (https://perkinelmerinformatics.com/products/research/chemdraw)^19^.

#### Evaluation of cytotoxicity in VERO normal cell line

For safe in vivo anticancer application, cytotoxic drug molecules must attain high selectivity towards cancer cells over normal cells to avoid cellular toxicity. Thus, the half-maximal cytotoxic concentration (CC_50_) of (**IIIf**) and (**VIb**) was determined against the non-cancerous VERO cell line (African Green Monkey Kidney cell line) to assess their relative toxicity to normal cells. The corrected averages of GI_50_ of (**IIIf**) and (**VIb**), obtained from the NCI five-dose assay excluding the outlier extreme GI_50_ value of NCI/ADR-RES cell line, were compared to their respective CC_50_ against VERO cells (Table 3). (**IIIf**) and (**VIb**) achieved high selectivity against tumor cells with selectivity indices of 53.71 and 30.93, respectively. Moreover, (**VIb**) potency and selectivity were remarkable against the most sensitive MCF-7 cell lines reaching over 100-fold selectivity. These results reflect the potential safety of (**IIIf**) and (**VIb**) on normal cells compared to cancer cells and encouraged us to perform further mechanistic studies to explore the cytotoxic effect of (**IIIf**) and (**VIb**).

**Table 3:** Cytotoxicity of (**IIIf**) and (**VIb**) against normal and tumor cells.

	**IIIf**	**VIb**
GI_50_ in NCI-60 assay corrected average (µM)	1.80	1.38
GI_50_ in MCF-7 (µM)	1.67	0.42
CC_50_ in vero cell line (µM ± SD)	96.68 ± 6.68	42.68 ± 2.06
Full NCI panel selectivity index (using GI_50_ corrected) average	53.71	30.93
Selectivity index against MCF-7 breast cancer cell line	57.89	101.62

#### Topoisomerase I & II enzyme inhibition assay

The web-based tool COMPARE provided by the NCI Developmental Therapeutic Program (DTP) was used to aid in the prediction of the mechanism of action of (**IIIf**) and (**VIb**) by comparing their growth inhibition patterns to that of previously tested compounds in the NCI databases based on the assumption that similar patterns of cytotoxicity are usually due to similar cellular targets^37^. The COMPARE analysis was performed against three databases containing: approved drugs, standard chemotherapeutic agents, and investigational drugs. COMPARE analysis results (Supplementary Figs. 63S and 64S) showed that (**IIIf**) and (**VIb**) possess growth inhibition patterns that are similar to several Topo inhibitors (doxorubicin and etoposide **5**). The cytotoxic profile of etoposide (**5**) (NSC 141540 and NSC 757804) was obtained from the NCI repository for comparison. As mentioned earlier, the corrected average was used to compare GI_50_ to alleviate the effects of outlier data of NCI/ADR-RES. Both compounds showed equipotent effects to etoposide in leukemia and breast subpanels. Furthermore, both out-performed etoposide in several subpanels, such as CNS, colon, and ovarian cancer. Individually, (**IIIf**) possessed a 3.5 to 18-fold increase in activity while (**VIb**) showed a 2.5 to 22-fold increase, as shown in Table 4.

**Table 4:** Mean GI_50_ concentrations (µM) of (**IIIf**), (**VIb**) and etoposide (**5**) across various subpanels in the NCI-60 human tumor cell lines screen five-dose assay.

Subpanel	GI_50_ (µM)		
	IIIf	VIb	Etoposide (5)
Leukemia	1.99	0.98	1.11
NSCLC	1.79	1.25	6.01
Colon cancer	1.80	1.15	16.56
CNS cancer	1.72	1.15	6.68
Melanoma	1.78	1.41	9.44
Ovarian cancer	1.75	1.44	31.41
Renal cancer	1.70	2.19	4.93
Prostate cancer	1.80	1.53	0.75
Breast cancer	1.87	1.32	1.70

Corrected average is used to express inhibition more accurately, minimizing the effect of the outlier value of NCI/ADR-RES cell line.

**Figure 10:**
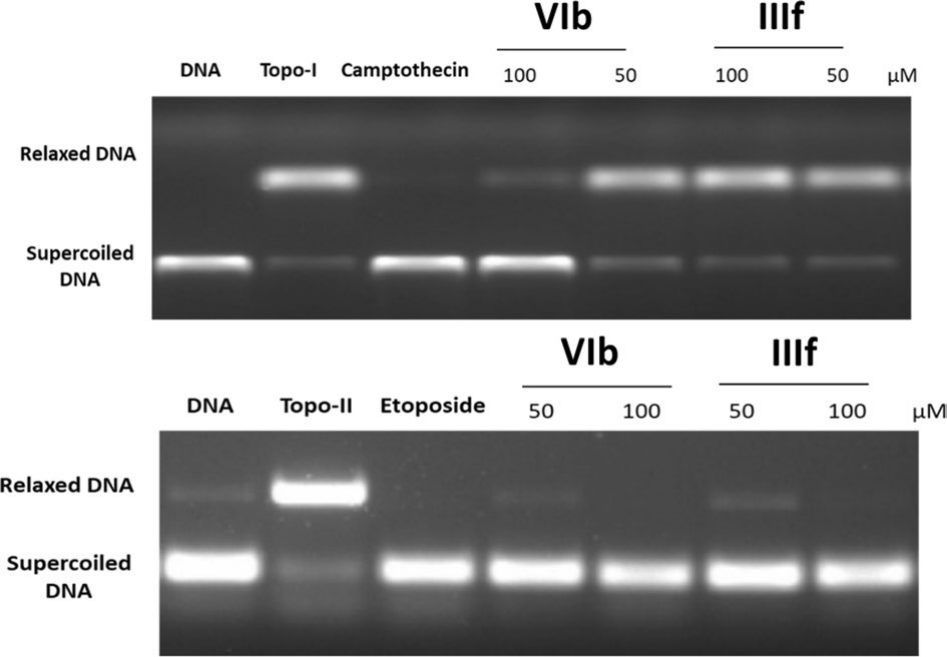
Recombinant Topo I & II inhibitory activities of (**IIIf**) and (**VIb**). DNA bands were visualized by transillumination with UV light and quantitated using AlphaImagerTM (http://genetictechnologiesinc.com, Alpha Innotech Corporation).

As suggested by COMPARE analysis results, we investigated the inhibitory effects of (**IIIf**) and (**VIb**) on the ability of Topo I and II to relax supercoiled plasmid DNA at 50 and 100 µM concentrations. Camptothecin and etoposide were used as positive controls on Topo I and II, respectively, at 100 µM. The products of Topo I and II relaxation assays were analyzed by electrophoretic mobility and developed in ethidium bromide in the presence of UV light. The inhibition of Topo **II** was prominent for both (**IIIf**) and (**VIb**) even at the lower tested concentration, while the inhibition of Topo **I** required a high concentration of (**VIb**) (Fig. 10). The overall inhibition behavior was of greater impact on Topo II than on Topo I, as evidenced by the values in Table 5.

**Table 5:** Recombinant Topo I and II inhibitory activities (% inhibition) of (**IIIf**) and (**VIb**).

Compound	Topo I % inhibition	Topo II % inhibition
**Etoposide** (100 µM)	–	96.84
**Camptothecin** (100 µM)	86.98	–
**IIIf** (50 µM)	9.67	73.84
**IIIf** (100 µM)	12.48	83.54
**VIb** (50 µM)	15.84	69.87
**VIb** (100 µM)	41.87	85.29

#### Cell cycle analysis using flow cytometry

**Figure 11:**
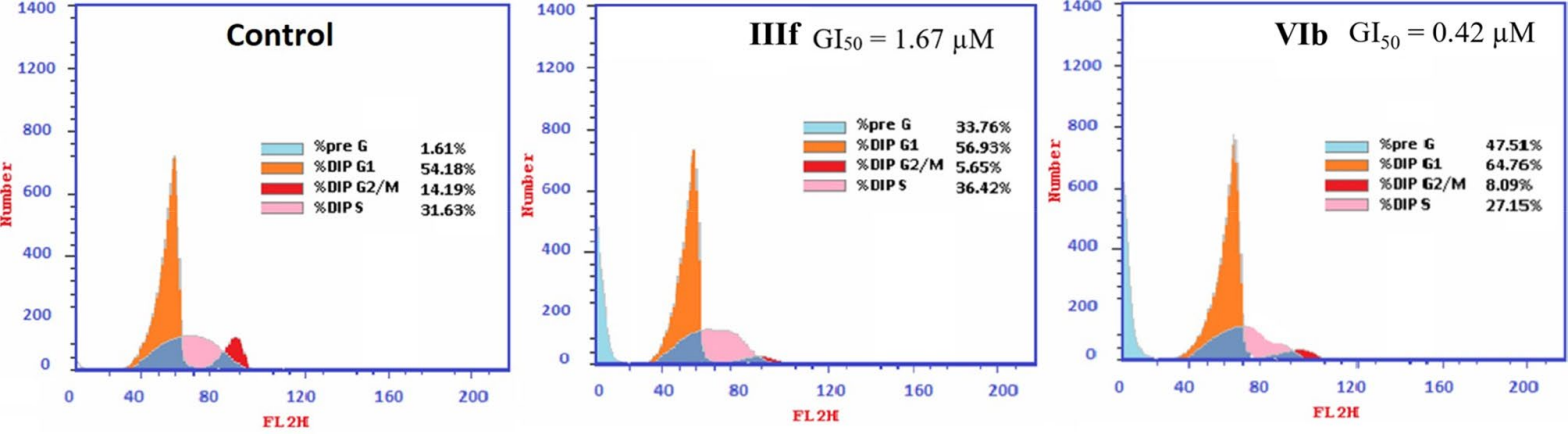
Cell cycle analysis of (**IIIf**) and (**VIb**) on MCF-7 cells following 24 h incubation at their respective GI_50_. Analysis performed using Cell Quest software 5.2.1 (https://www.bdbiosciences.com, Becton Dickinson Immunocytometry Systems, San Jose, CA).

Next, we analyzed the cell population of the most selective cell line (MCF-7) following treatment by (**IIIf**) and (**VIb**) at their respective GI_50_ (1.67 and 0.42 µM, respectively) for 24 h. As shown in Fig. 11, (**IIIf**) and (**VIb**) exhibited about a 21-fold and 29.5-fold increase in the preG phase, respectively. This points to the ability of the compounds to induce apoptosis. (**IIIf**) demonstrated an increase in S (36.42 ± 1.46%) and a decrease in G2/M (5.65 ± 0.29%) phases, compared to the untreated control S (31.63 ± 1.48%) and G2/M (14.19 ± 1.35%) hinting G1/S phase arrest. On the other hand, (**VIb**) caused G1 phase arrest evidenced by an elevation in G1 (64.76 ± 1.55%) and a decrease in both S (27.15 ± 0.34%) and G2/M (8.09 ± 1.35%) compared to control G1 (54.18 ± 1.31%), S (31.63 ± 1.48%), and G2/M (14.19 ± 1.35%).

#### Apoptosis analysis (annexin V-FITC assay)

**Figure 12:**
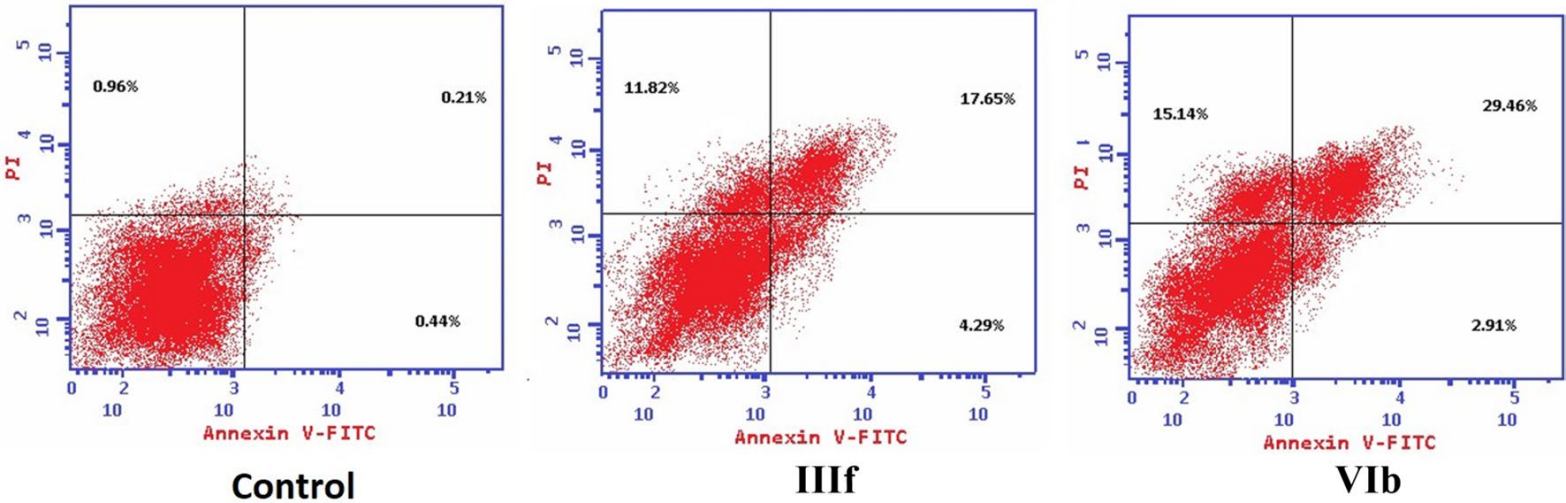
Apoptosis population distribution of (**IIIf**) and (**VIb**) treated MCF-7 cells after 24 h incubation. Analysis performed using BD FACS Calibur (https://www.bdbiosciences.com, BD Biosciences, San Jose, CA).

Given the selectivity and potency of (**IIIf**) and (**VIb**), the apoptotic effects of the compounds were further assessed in the MCF-7 cell line upon treatment by (**IIIf**) and (**VIb**) for 24 h at their respective GI_50_ using annexin V. Both compounds produced a significant elevation in the total apoptotic cellular population reaching 33.75-fold and a 49.80-fold increase for (**IIIf**) and (**VIb**), respectively (Table 6 and Fig. 12). These data hint at the activation of the apoptosis cascade through intrinsic and/or extrinsic pathways.

**Table 6:** Apoptosis population percentage of (**IIIf**) and (**VIb**) treated MCF-7 cells after 24 h incubation.

Sample code	Tested conc (µM)	Early apoptosis %	Late apoptosis %	Necrosis %
IIIf	1.67	4.29 ± 0.69	17.65 ± 1.52	11.82 ± 0.93
VIb	0.42	2.91 ± 0.15	29.46 ± 2.04	15.14 ± 0.56
Control	0	0.44 ± 0.02	0.21 ± 0.01	0.96 ± 0.10

#### Western blot analysis (Bax/Bcl-2)

**Figure 13:**
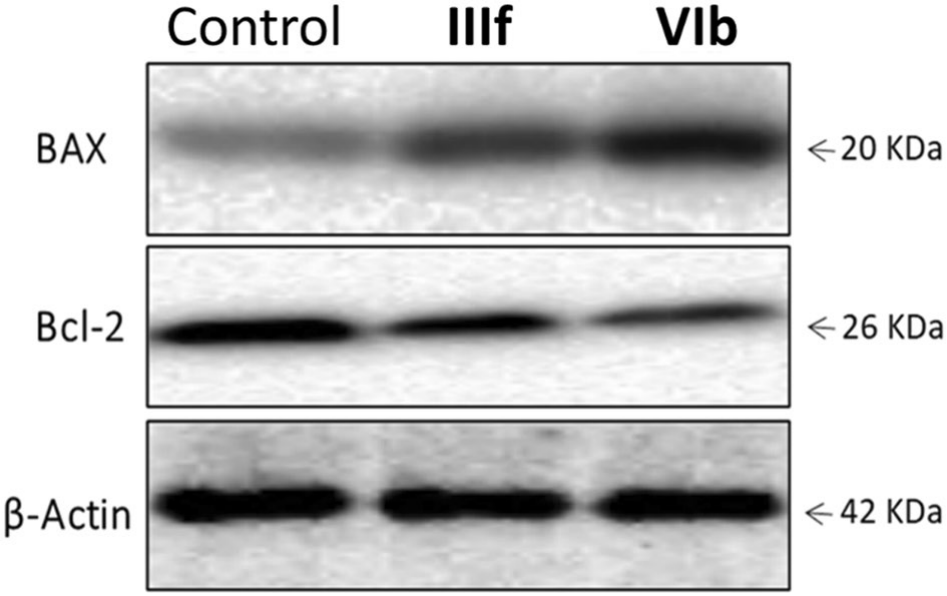
Bax and Bcl-2 expressions in MCF-7 cells after treatment with (**IIIf**) and (**VIb**). Chemiluminescent signals were captured using a CCD camera-based imager (https://www.bio-rad.com, Chemi Doc imager, Biorad, USA).

The pro-apoptotic Bax and anti-apoptotic Bcl-2 are two major members of the Bcl-2 family that affects tumor progression or inhibition via the intrinsic apoptotic pathways triggered by mitochondrial dysfunction^39,40^. This intricate balance between pro-and anti-apoptotic members of this family can determine the cellular fate^41^. The expression of apoptotic markers Bax and Bcl-2 were assessed in the MCF-7 cell line using western blotting in the presence of (**IIIf**) and (**VIb**) at their GI_50_ concentrations. Both compounds demonstrated an increase in the Bax/Bcl-2 ratio compared to β-actin as a control (Fig. 13). (**IIIf**) produced a 1.76-fold increase in Bax expression and about half Bcl-2 expression. While (**VIb**) increased the Bax over two-folds and decreased Bcl-2 to less than half. The Bax/Bcl-2 ratio increased by 3 and 6-folds upon treatment with (**IIIf**) and (**VIb**), respectively, compared to the β-actin ratio (Table 7). Based on these results, the activation of the intrinsic apoptotic pathways contributes to the cellular death observed in the MCF-7 cell line.

**Table 7:** Effects of (**IIIf**) and (**VIb**) on Bax and Bcl-2 protein expression in MCF-7 cells.

Sample	Tested conc. (µM)	Bax	Bcl-2	Bax/Bcl-2 ratio
Control	0	1.00	1.00	1.00
IIIf	1.67	1.76	0.52	3.38
VIb	0.42	2.31	0.39	5.92

All the data are normalized to β-actin which is set to ‘1’.

Values are given as fold changes from the control.

#### ELISA analysis (caspase-8/9 assay)

The caspases-8 and 9 are two initiator proteases involved in the apoptosis cascade, activated via extrinsic death receptors activation, and intrinsic disruption of mitochondrial membrane and release of cytochrome C, respectively^42^. After activation, both activate executioner caspases-3, 6, and 7, resulting in apoptosis. However, genetic studies of some cancer cells detected aberrations in the apoptotic cascade, such as in the case of MCF-7, which lacks caspase-3^43,44^. Accordingly, the expression of caspases-8 and 9 were assessed in the MCF-7 cell line using ELISA (enzyme-linked immunosorbent assay) in the presence of (**IIIf**) and (**VIb**) at their GI_50_ concentrations. Both compounds demonstrated an increase in both caspase-8 and 9 compared to the control. (**IIIf**) and (**VIb**) both produced a two-fold increase in caspase-8 and around a three-fold increase in caspase-9 compared to control (Table 8), suggesting the activation of both intrinsic and extrinsic apoptotic pathways with a more prominent effect on the intrinsic pathway.

**Table 8:** Effects of (**IIIf**) and (**VIb**) on Caspase-8 and 9 protein expression in MCF-7 cells.

Sample	Tested conc (µM)	Caspase-8 (ng/mL)	Caspase-9 (ng/mL)
Control	0	1.39 ± 0.06	29.12 ± 2.41
IIIf	1.67	2.87 ± 0.05	78.94 ± 2.48
VIb	0.42	2.68 ± 0.16	89.41 ± 4.00

### In silico study

#### Pharmacokinetic profiling

**Figure 14:**
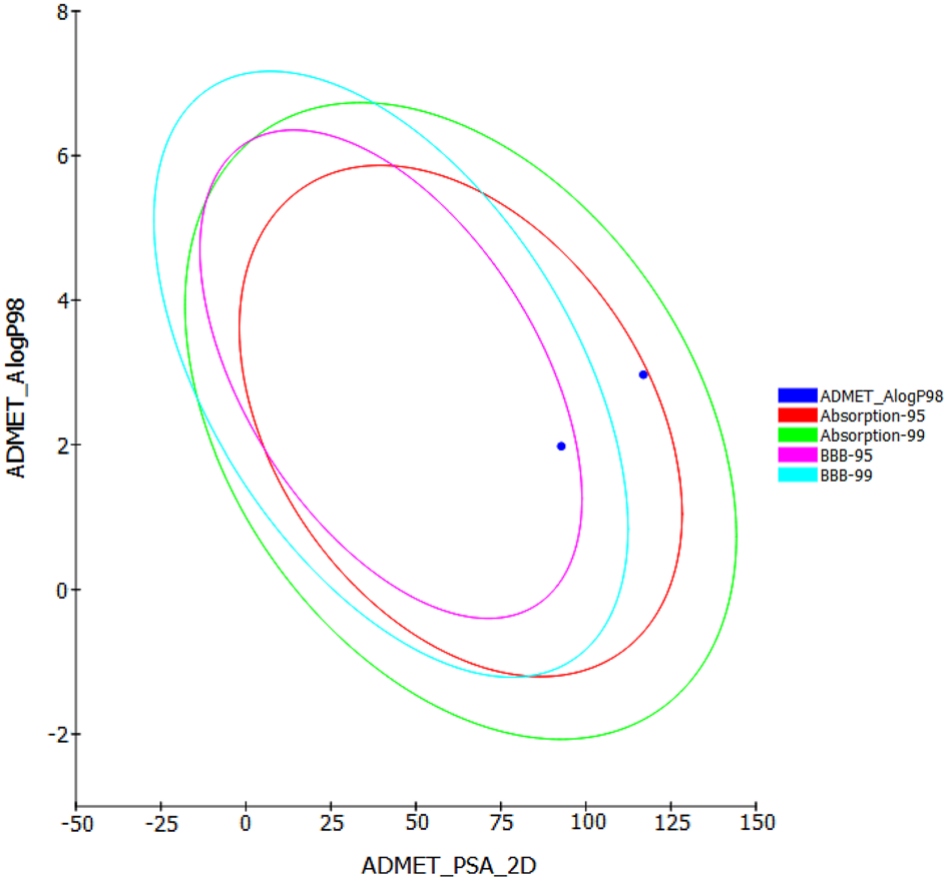
ADME plot of the (**IIIf**) and (**VIb**). (Dassault Systèmes. (2016). BIOVIA Discovery Studio Studio was used to generate this figure, https://discover.3ds.com^45^.

Determination of the pharmacokinetic behavior of the drugs is a critical process in drug discovery. Thus, many tools are employed to predict the biological accessibility of the compound to the target. Accordingly, Biovia Discovery Studio was used to predict the in silico ADME profiles of (**IIIf**) and (**VIb**)^45^. Consistent with our design, the two compounds were predicted to have good intestinal absorption and low BBB permeability. The two compounds showed no interaction with cytochrome P450 2D6 (CYP2D6) and no plasma proteins binding (PPB) (Fig. 14).

To rationalize the higher potency of (**VIb**) over (**IIIf**), Chemicalize (www.chemicalize.org) server was employed to assess several physicochemical properties of both compounds^46^. While most of the computed properties for (**IIIf**) and (**VIb**) were similar or almost identical, ofloxacin derivative (**VIb**) showed higher relative rigidity over moxifloxacin derivative (**IIIf**) as demonstrated by the lower number of conformers as calculated using Molecular Operating Environment (MOE, 2008.10) software^47^. Additionally, (**VIb**) exhibited higher solubility compared to (**IIIf**). Both factors appear to contribute to the superior cytotoxicity of (**VIb**) (Table 9).

**Table 9:** Computed physiochemical properties of (**IIIf**) and (**VIb**).

	IIIf	VIb
No. of systematic conformational search iterations	110,592	144
No. of low-energy conformers	71	21
	More flexible	More rigid
Solubility category	moderate	high

#### Docking study

**Figure 15:**
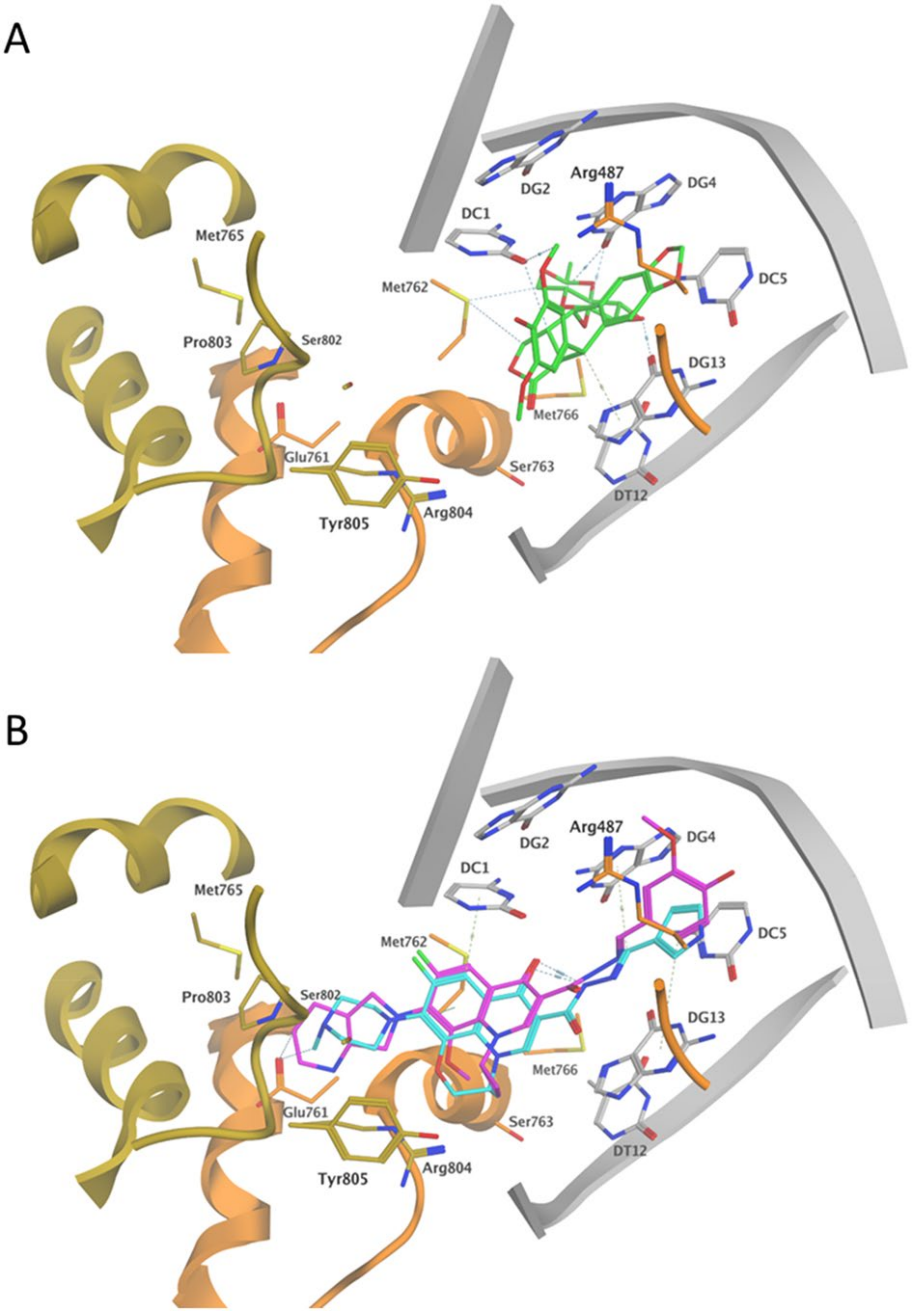
(**A**) 3D representation of etoposide (**5**) (green) in stick view bound to Topo II (chain A: orange, chain B: gold) and DNA (grey) in carton representation (PDB: 6ZY7). (**B**) 3D representation of the top docking poses of (**IIIf**) (magenta) and (**VIb**) (cyan) in stick view bound to Topo II (chain A: orange, chain B: gold) and DNA (grey) in carton representation. Generated by MOE (Molecular Operating Environment, www.chemcomp.com) 2008.10 software^47^.

Finally, to explore the binding modes of (**IIIf**) and (**VIb**) to Topo II, the two compounds were docked into the binding site of the recently published cryo-EM structure of the entire human topoisomerase II alpha nucleoprotein complex trapped by etoposide (**5**) (PDB:6ZY7)^48^. The structure is comprised of two protein chains (A and B) in complex with 30 DNA base pairs solved at a resolution of 4.64 Å^48^. The docking protocol was validated by redocking of etoposide (**5**) at the active site where an RMSD (Root Mean Square Deviation) of 0.55 Å was obtained for the superimposition of the coordinates of etoposide in the cryo-EM structure and its top docking pose (Supplementary Fig. 66S). The 3D interactions of etoposide (**5**) show its ability to form a ternary complex with both DNA and Topo II, simultaneously thus stabilizing the Topo-DNA complex and preventing its cleavage with a docking score of − 10.63 kcal/mol. The planar fused tetracyclic ring system of etoposide interacts solely with DC1, DG4, DC5, and DG13 DNA nucleobases via distance-dependent hydrophobic interactions. The dimethoxy phenolic moiety formed hydrogen bonds (HB) to DC1 in addition to distance-dependent hydrophobic interactions with DC1 and DG2 nucleobases and topo II residues GLY462A, ASP463A, ARG487A and TYR805B. On the other hand, the *trans* sugar moiety was engaged in hydrophobic interactions with DC1, DG4, DC5, and DG13 as well as MET762A and MET766A in addition to HB to DG4, and DG13 nucleobases (Supplementary Fig. 67S). The docking results of (**IIIf**) and (**VIb**) suggest favorable bindings with negative docking scores where (**IIIf**) achieved a comparable docking score of − 10.42 kcal/mol, whereas surprisingly, (**VIb**) had a lower docking score of − 9.76 kcal/mol. In agreement with the design hypothesis, an intramolecular HB between the hydrazide NH and the quinolinone carbonyl moiety was observed in several docking poses. Similar to etoposide, (**IIIf**) and (**VIb**) interacted with both DNA nucleobases and topo II amino acids, as shown in Fig. 15. The arylidene hydrazone moieties of (**IIIf**) and (**VIb**) occupied the position of the tetracyclic ring of etoposide, where it interacted with the same set of DNA nucleobases in addition to DG2 and ARG487A. The quinolinone core of (**IIIf**) and (**VIb**) occupied the center of the binding site interacting with DC1 nucleobase and MET762A as well as TYR805B. The positively charged alicyclic moieties at *C*7 of the quinoline core in (**IIIf**) and (**VIb**) were placed at a spot not occupied by etoposide extending toward both chains of Topo II. (**IIIf**) and (**VIb**) formed charged HB interaction with GLU761A and engaged in several distance-dependent hydrophobic interactions with ALA745B, MET765B, SER802B, PRO803B, ARG804B, TYR805, GLU761A and MET762A. Thus, docking results of (**IIIf**) and (**VIb**) highlight some similarities and distinctions in their binding mode compared to etoposide, where the lack of HB with the nucleobases was compensated by numerous hydrophobic interactions with the residues of the two chains of topo II and a charged HB to GLU761A. This binding mode also hints at the possibility of future structure extension towards the spots occupied by the dimethoxy phenolic and/or sugar moieties of etoposide to achieve superior binding affinities.

## Conclusion

We designed and synthesized a series of FQ-based cytotoxic agents by functionalizing the acid hydrazides of three clinically approved FQ drugs (moxifloxacin, ofloxacin, and ciprofloxacin). Moxifloxacin derivatives (**IIIa–j**) were the most promising in activity with (**IIIf**) reaching a GI_50_ of 1.43 µM against the SNB-75 CNS cell line. (**VIb**) was the only active ofloxacin derivative eliciting a more potent effect reaching sub-micromolar level against many cell lines including MDA-MB-468 and MCF-7 breast cancer cell lines (GI_50_ = 0.41 and 0.42 µM, respectively), NSCLC cell line HOP-92 (GI_50_ = 0.50 µM) and CNS cell lines SNB-19 and U-251 (GI_50_ = 0.51 and 0.61 µM, respectively). Both (**IIIf**) and (**VIb**) were highly selective towards the NCI-60 panel of cell lines compared to Vero normal cells, especially against the MCF-7 breast cancer cell line with selectivity indices of 57.89 and 101.62, respectively. Treatment of MCF-7 cells with (**IIIf**) resulted in the accumulation of cells at G1/S, whereas (**VIb**) treatment resulted in cell accumulation at G1. Both compounds induced apoptosis mainly through the intrinsic pathway as shown by the increased ratio of Bax/Bcl-2 and caspase-9.

Additionally, the extrinsic pathway was activated to a lesser extent as indicated by the increased concentration of caspase-8. COMPARE analysis of (**IIIf**) and (**VIb**) predicted growth inhibition patterns mimicking several Topo inhibitors. Both compounds inhibited Topo I and II at 50 and 100 µM with preferential effect on Topo II. Docking study of (**IIIf**) and (**VIb**) highlights a different binding mode to Topo II compared to etoposide involving the formation of numerous hydrophobic interactions with the residues of the two chains of the enzyme and a charged HB to GLU761A in a spot not occupied by etoposide. It also hints at the possibility of future structure extension towards the spots occupied by the dimethoxy phenolic and/or sugar moieties of etoposide and not by (**IIIf**) and (**VIb**) to achieve superior binding affinities. The in silico pharmacokinetic study predicted favorable profiles for both compounds with high potential for oral bioavailability, low toxicity, and minimal drug interactions.

Moreover, it suggests that (**IIIf**) and (**VIb**) differences in activity could be attributed to solubility and relative rigidity differences. In conclusion, (**IIIf**) and (**VIb**) represent good lead molecules for the development of cytotoxic agents from quinolone scaffolds. Follow-up with in-depth mechanistic studies are warranted.

## Experimental

### Chemistry

#### General

Melting points were determined by the open capillary method on the Stuart SMP10 apparatus and the values were uncorrected. Yield values are calculated after the purification of each compound. IR spectra were determined as KBr discs on Shimadzu IR 8400 s spectrophotometer and values were represented in cm^−1^. ^1^H NMR spectra were carried out using Bruker 400-BB (400 MHz) using tetramethylsilane (TMS) as internal standard and chemical shift values were recorded in ppm on *δ* scale at Microanalytical Unit, Faculty of Pharmacy, Cairo University, Egypt. ^13^C NMR spectra were carried out using Bruker 400-BB (101 MHz) using tetramethylsilane (TMS) as internal standard and chemical shift values were recorded in ppm on *δ* at Microanalytical Unit, Faculty of Pharmacy, Cairo University, Egypt. Mass spectra were run at 70 eV on Shimadzu GCMS-QP/MS-QP5050A spectrometer. Element analyses and other biological evaluation studies were carried out at the Regional Center for Mycology and Biotechnology, Faculty of Pharmacy, Al Azhar University, Egypt. All chemicals used were purchased from Sigma-Aldrich, Alfa Aesar, and Loba chem. and used without further purification.

#### Synthesis of 1-cyclopropyl-6-fluoro-8-methoxy-7-(octahydro-*6H*-pyrrolo[3,4-*b*]pyridin-6-yl)-4-oxo-1,4-dihydroquinoline-3-carbohydrazide (I)

Moxifloxacin HCl **I** (4.37 g, 10 mmol) and hydrazine hydrate (80%, 25 mL) were heated under reflux for 24 h. The reaction mixture was cooled. The formed precipitate was filtered, washed with water, dried and recrystallized from ethanol to afford (**I)** as a brownish buff powder (yield: 61%), m.p. 221–223 °C; IR (KBr, ν cm^-1^): 3441 (NH stretching), 3313, 3209 (NH_2_ stretching), 3066 (CH aromatic stretching), 2927 (CH aliphatic stretching), 1666 (2C=O stretching).; ^1^H NMR (400 MHz, CDCl_3_) *δ* ppm: 0.96–1.23 (m, 4H, 2CH_2_), 1.45–1.82 (m, 4H, 2CH_2_), 2.23–2.34 (m, 1H, CH), 2.61–3.04 (m, 2H, CH_2_), 3.26–3.41 (m, 5H, CH, CH_2_, NH_2_, D_2_O exchangeable), 3.52 (s, 3H, –OCH_3_), 3.85–3.97 (m, 4H, CH, CH_2_, NH, D_2_O exchangeable), 7.68 (d, 1H, *J* = 14.20 Hz, C5–H), 8.70 (s, 1H, C2–H), 10.76 (s, 1H, NH, D_2_O exchangeable); ^13^C NMR (101 MHz, CDCl_3_) *δ* ppm: 8.70, 10.20, 22.02, 23.38, 36.82, 39.76, 44.94, 52.32, 56.58, 58.46, 60.89, 107.77, 109.66, 119.80, 133.88, 136.42, 140.46, 148.47, 152.08, 165.75, 174.58; Anal. Calcd. for C_21_H_26_FN_5_O_3_ (415.47): C, 60.71; H, 6.31; N, 16.86; Found: C, 60.89; H, 6.44; N, 17.12.

#### Synthesis of 1-cyclopropyl-6-fluoro-8-methoxy-7-(octahydro-*6H*-pyrrolo[3,4-*b*]pyridin-6-yl)-4-oxo-*N'*-(1-phenylethylidene)-1,4-dihydroquinoline-3-carbohydrazide (II)

An equimolar amount of moxifloxacin hydrazide (**I**) (0.42 g, 1 mmol) and acetophenone (0.12 g, 1 mmol) were dissolved in ethanol (10 mL) and acetic acid (0.20 mL). The mixture was heated under reflux for 4 h. The solvent was evaporated under vacuum and the residue was crystallized from ethanol to yield compound (**II**) as brown powder (yield: 48%), m.p. 98–102 °C; IR (KBr, ν cm^−1^): 3417, 3394 (2NH stretching), 3155 (CH aromatic stretching), 2939 (CH aliphatic stretching), 1678 (2C=O stretching); ^1^H NMR (400 MHz, CDCl_3_) *δ* ppm: 0.96–1.26 (m, 4H, 2CH_2_), 1.62–1.84 (m, 4H, 2CH_2_), 2.45 (s, 3H, CH_3_), 2.47–2.54 (m, 1H, CH), 2.77–3.23 (m, 2H, CH_2_), 3.54–3.69 (m, 6H, –OCH_3_, CH_2_, NH, D_2_O exchangeable), 3.86–4.01 (m, 3H, CH, CH_2_), 4.72–4.76 (m, 1H, CH), 7.34–7.39 (m, 3H, Ar–H), 7.80 (d, 1H, *J* = 14.20 Hz, C5–H), 7.85–7.89 (m, 2H, Ar–H), 8.88 (s, 1H, C2–H), 13.13 (s, 1H, NH, D_2_O exchangeable); ^13^C NMR (101 MHz, CDCl_3_) *δ* ppm: 8.89, 10.14, 14.69, 19.69, 22.34, 35.59, 40.00, 42.80, 52.44, 55.13, 55.54, 61.29, 107.97, 110.17, 120.16, 126.81, 128.27, 129.31, 133.76, 135.94, 138.30, 140.74, 149.45, 152.01, 152.46, 155.22, 161.91, 174.93, 176.55; Anal. Calcd. for C_29_H_32_FN_5_O_3_ (517.61): C, 67.29; H, 6.23; N, 13.53; Found: C, 67.43; H, 6.45; N, 13.78.

#### General procedure for the synthesis of compounds (IIIa–j)

In absolute ethanol (10 mL), equimolar amounts of moxifloxacin carbohydrazide (**I**) (0.21 g, 0.5 mmol) and the corresponding aldehyde (0.5 mmol) were dissolved and heated under reflux for 2–6 h. After cooling, the solvent was evaporated in vacuum and the residue was crystallized from ethanol to produce the required components (**IIIa–j**).

##### *N'*-Benzylidene-1-cyclopropyl-6-fluoro-8-methoxy-7-(octahydro-6*H*-pyrrolo[3,4-*b*]pyridin-6-yl)-4-oxo-1,4-dihydroquinoline-3-carbohydrazide (IIIa)

Pale brown powder (yield 88%), m.p. 100–104 °C; IR (KBr, ν cm^−1^): 3452, 3321 (2NH stretching), 3062 (CH aromatic stretching), 2927 (CH aliphatic stretching), 1678 (2C=O stretching); ^1^H NMR (400 MHz, DMSO-*d*_*6*_) *δ* ppm: 0.94–1.27 (m, 4H, 2CH_2_), 1.37–1.76 (m, 4H, 2CH_2_), 2.16–2.27 (m, 1H, CH), 2.51, 2.93 (m, 2H, CH_2_), 3.20–3.31 (m, 4H, CH_2_, CH, NH, D_2_O exchangeable), 3.56 (s, 3H, –OCH_3_), 3.80–3.97 (m, 2H, CH_2_), 4.04–4.14 (m, 1H, CH), 7.38–7.50 (m, 3H, Ar–H), 7.60 (d, 1H, *J* = 14.20 Hz, C5–H), 7.73 (d, 2H, *J* = 6.50 Hz, Ar–H), 8.34 (s, 1H, –CH=N–), 8.67 (s, 1H, C2–H), 13.13 (s, 1H, NH, D_2_O exchangeable); ^13^C NMR (101 MHz, DMSO-*d*_*6*_) *δ* ppm: 8.74, 10.33, 21.85, 23.29, 36.76, 40.02, 44.85, 52.37, 56.48, 58.33, 60.99, 107.71, 109.77, 119.51, 127.76, 128.57, 130.14, 134.12, 136.65, 140.38, 148.05, 149.42, 152.14, 154.61, 162.02, 174.84; Anal. Calcd. for C_28_H_30_FN_5_O_3_ (503.58): C, 66.78; H, 6.01; N, 13.91; Found: C, 66.94; H, 6.23; N, 14.17.

##### 1-Cyclopropyl-6-fluoro-*N'*-(4-fluorobenzylidene)-8-methoxy-7-(octahydro-*6H*-pyrrolo[3,4-*b*]pyridin-6-yl)-4-oxo-1,4-dihydroquinoline-3-carbohydrazide (IIIb)

White powder (yield 75%), m.p. 250–253 °C; IR (KBr, ν cm^−1^): 3444, 3340 (2NH stretching), 3087 (CH aromatic stretching), 2927 (CH aliphatic stretching), 1670 (2C=O stretching); ^1^H NMR (400 MHz, DMSO-*d*_*6*_) *δ* ppm: 0.93–1.24 (m, 4H, 2CH_2_), 1.41–1.74 (m, 4H, 2CH_2_), 2.19–2.26 (m, 1H, CH), 2.48–2.90 (m, 2H, CH_2_), 3.17–3.37 (m, 4H, CH_2_, CH, NH, D_2_O exchangeable), 3.56 (s, 3H, –OCH_3_), 3.84–4.00 (m, 2H, CH_2_), 4.08–4.13 (m, 1H, CH), 7.24–7.37 (m, 2H, Ar–H), 7.63 (d, 1H, *J* = 14.10 Hz, C5–H), 7.74–7.84 (m, 2H, Ar–H), 8.40 (s, 1H, –CH=N–), 8.68 (s, 1H, C2–H), 13.15 (s, 1H, NH, D_2_O exchangeable); ^13^C NMR (101 MHz, DMSO-*d*_*6*_) *δ* ppm: 8.77, 10.27, 21.63, 23.20, 36.67, 39.98, 44.69, 52.44, 56.41, 58.06, 61.06, 107.75, 109.83, 115.58, 119.67, 119.75, 129.53, 130.44, 136.55, 140.50, 146.69, 149.39, 152.16, 154.63, 161.96, 162.66, 165.15, 174.83; Anal. Calcd. for C_28_H_29_F_2_N_5_O_3_ (521.57): C, 64.48; H, 5.60; N, 13.43; Found: C, 64.31; H, 5.49; N, 13.70.

##### 1-Cyclopropyl-6-fluoro-8-methoxy-*N'*-(4-methoxybenzylidene)-7-(octahydro-*6H*-pyrrolo[3,4-*b*]pyridin-6-yl)-4-oxo-1,4-dihydroquinoline-3-carbohydrazide (IIIc)

Pale brown crystals (yield 75%), m.p. 108–112 °C; IR (KBr, ν cm^−1^): 3498, 3313 (2NH stretching), 3078 (CH aromatic stretching), 2931 (CH aliphatic stretching), 1678 (2C=O stretching); ^1^H NMR (400 MHz, DMSO-*d*_*6*_) *δ* ppm: 0.93–1.26 (m, 4H, 2CH_2_), 1.36–1.70 (m, 4H, 2CH_2_), 2.13–2.25 (m, 1H, CH), 2.48–2.91 (m, 2H, CH_2_), 3.15–3.23 (m, 4H, CH_2_, CH, NH, D_2_O exchangeable), 3.55 (s, 3H, –OCH_3_), 3.80 (s, 3H, –OCH_3_), 3.83–3.93 (m, 2H, CH_2_), 4.03–4.11 (m, 1H, CH), 6.99 (d, 2H, *J* = 8.30 Hz, Ar–H), 7.57 (d, 1H, *J* = 14.30 Hz, C5–H), 7.66 (d, 2H, *J* = 8.40 Hz, Ar–H), 8.23 (s, 1H, –CH=N–), 8.64 (s, 1H, C2-H), 13.01 (s, 1H, NH, D_2_O exchangeable); ^13^C NMR (101 MHz, DMSO-*d*_*6*_) *δ* ppm: 8.73, 10.29, 21.80, 23.25, 36.68, 40.01, 44.75, 52.34, 55.35, 56.41, 58.23, 60.97, 107.64, 107.88, 109.75, 114.02, 114.31, 119.48, 126.81, 129.32, 133.96, 136.58, 140.36, 147.90, 149.29, 152.09, 154.56, 161.83, 174.79; Anal. Calcd. for C_29_H_32_FN_5_O_4_ (533.60): C, 65.28; H, 6.04; N, 13.12; Found: C, 65.13; H, 6.21; N, 13.36.

##### 1-Cyclopropyl-*N'*-(4-(dimethylamino)benzylidene)-6-fluoro-8-methoxy-7-(octahydro-*6H*-pyrrolo[3,4-*b*]pyridin-6-yl)-4-oxo-1,4-dihydroquinoline-3-carbohydrazide (IIId)

White powder (yield 37%), m.p. 257–260 °C; IR (KBr, ν cm^−1^): 3308 (2NH stretching), 3080 (CH aromatic stretching), 2924 (CH aliphatic stretching), 1670 (2C=O stretching); ^1^H NMR (400 MHz, CDCl_3_) *δ* ppm: 0.76–1.25 (m, 4H, 2CH_2_), 1.48–1.85 (m, 4H, 2CH_2_), 2.27–2.33 (m, 1H, CH), 2.65–3.09 (m, 2H, CH_2_), 3.00 (s, 6H, –N(CH_3_)_2_), 3.31–3.50 (m, 4H, CH_2_, CH, NH, D_2_O exchangeable), 3.54 (s, 3H, –OCH_3_), 3.89–4.01 (m, 3H, CH, CH_2_), 6.67 (d, 2H, *J* = 8.50 Hz, Ar–H), 7.65 (d, 2H, *J* = 8.40 Hz, Ar–H), 7.76 (d, 1H, *J* = 14.20 Hz, C5–H), 8.08 (s, 1H, –CH=N–), 8.86 (s, 1H, C2–H), 13.04 (s, 1H, NH, D_2_O exchangeable); ^13^C NMR (101 MHz, CDCl_3_) *δ* ppm: *δ* 8.77, 10.26, 21.67, 23.23, 36.65, 39.88, 40.18, 44.68, 52.42, 56.40, 57.95, 61.01, 107.67, 108.49, 110.10, 111.64, 119.68, 121.86, 129.26, 133.93, 136.42, 140.48, 148.97, 149.11, 151.71, 152.11, 154.59, 161.26, 174.79; Anal. Calcd. for C_30_H_35_FN_6_O_3_ (546.65): C, 65.92; H, 6.45; N, 15.37; Found: C, 65.70; H, 6.71; N, 15.62.

##### *N'*-(Benzo[*d*][1,3]dioxol-5-ylmethylene)-1-cyclopropyl-6-fluoro-8-methoxy-7-(octahydro-6*H*-pyrrolo[3,4-*b*]pyridin-6-yl)-4-oxo-1,4-dihydroquinoline-3-carbohydrazide (IIIe)

Grey powder (yield 59%), m.p. 237–239 °C; IR (KBr, ν cm^−1^): 3454, 3332 (2NH stretching), 3070 (CH aromatic stretching), 2927 (CH aliphatic stretching), 1678 (2C=O stretching); ^1^H NMR (400 MHz, CDCl_3_) *δ* ppm: 0.76–1.25 (m, 4H, 2CH_2_), 1.46–1.80 (m, 4H, 2CH_2_), 2.23–2.33 (m, 1H, CH), 2.59–3.05 (m, 2H, CH_2_), 3.27–3.42 (m, 4H, CH_2_, CH, NH, D_2_O exchangeable), 3.53 (s, 3H, –OCH_3_), 3.86–3.99 (m, 3H, CH_2_, CH), 5.96 (s, 2H, O–CH_2_–O), 6.77 (d, 1H, *J* = 8.00 Hz, Ar–H), 7.06 (d, 1H, *J* = 8.00 Hz, Ar–H), 7.44 (s, 1H, Ar–H), 7.73 (d, 1H, *J* = 14.20 Hz, C5–H), 8.06 (s, 1H, –CH=N–), 8.84 (s, 1H, C2–H), 13.14 (s, 1H, NH, D_2_O exchangeable); ^13^C NMR (101 MHz, CDCl_3_) *δ* ppm: 8.71, 10.24, 21.92, 23.33, 36.79, 39.98, 44.79, 52.37, 52.44, 56.46, 58.38, 60.97, 101.41, 106.31, 107.67, 107.91, 109.78, 119.57, 123.81, 128.76, 133.97, 136.61, 140.53, 147.69, 149.28, 152.17, 154.64, 161.70, 174.79.; Anal. Calcd. for C_29_H_30_FN_5_O_5_ (547.59): C, 63.61; H, 5.52; N, 12.79; Found: C, 63.78; H, 5.66; N, 12.98.

##### 1-Cyclopropyl-6-fluoro-*N'*-(4-hydroxy-3-methoxybenzylidene)-8-methoxy-7-(octahydro-6*H*-pyrrolo[3,4-*b*]pyridin-6-yl)-4-oxo-1,4-dihydroquinoline-3-carbohydrazide (IIIf)

Yellowish white powder (yield 75%), m.p. 189–193 °C; IR (KBr, ν cm^−1^): 3448 (OH phenolic stretching), 3313, 3290 (2NH stretching), 3078 (CH aromatic stretching), 2931 (CH aliphatic stretching), 1666 (2C=O stretching); ^1^H NMR (400 MHz, DMSO-*d*_*6*_) *δ* ppm: 0.94–1.27 (m, 4H, 2CH_2_), 1.40–1.75 (m, 4H, 2CH_2_), 2.19–2.30 (m, 1H, CH), 2.51–2.93 (m, 2H, CH_2_), 3.18–3.36 (m, 5H, CH_2_, CH, NH, D_2_O exchangeable, OH, D_2_O exchangeable), 3.53 (s, 3H, –OCH_3_), 3.83 (s, 3H, –OCH_3_), 3.80–3.99 (m, 2H, CH_2_), 4.04–4.11 (m, 1H, CH), 6.81 (d, 1H, *J* = 8.10 Hz, Ar–H), 7.06 (d, 1H,* J* = 8.10 Hz, Ar–H), 7.24 (s, 1H, Ar–H), 7.52 (d, 1H,* J* = 14.30 Hz, C5–H), 8.12 (s, 1H, –CH=N–), 8.64 (s, 1H, C2–H), 12.98 (s, 1H, NH, D_2_O exchangeable); ^13^C NMR (101 MHz, DMSO-*d*_*6*_) *δ* ppm: 8.86, 10.16, 20.92, 22.87, 36.66, 39.98, 44.72, 51.26, 56.14, 56.46, 57.88, 61.02, 107.88, 108.22, 109.75, 112.83, 115.15, 123.50, 126.69, 133.83, 139.88, 147.54, 148.09, 148.29, 149.46, 151.42, 154.11, 161.55, 174.72.; Anal. Calcd. for C_29_H_32_FN_5_O_5_ (549.60): C, 63.38; H, 5.87; N, 12.74; Found: C, 63.42; H, 5.79; N, 12.96.

##### *N'*-(4-Bromobenzylidene)-1-cyclopropyl-6-fluoro-8-methoxy-7-(octahydro-6*H*-pyrrolo[3,4-*b*]pyridin-6-yl)-4-oxo-1,4-dihydroquinoline-3-carbohydrazide (IIIg)

Brown powder (yield 48%), m.p. 249–253 °C; IR (KBr, ν cm^−1^): 3429, 3360 (2NH stretching), 3074 (CH aromatic stretching), 2924 (CH aliphatic stretching), 1670 (2C=O stretching); ^1^H NMR (400 MHz, DMSO-*d*_*6*_) *δ* ppm: 0.85–1.24 (m, 4H, 2CH_2_), 1.37–1.76 (m, 4H, 2CH_2_), 2.22–2.26 (m, 1H, CH), 2.52–2.85 (m, 2H, CH_2_), 3.19–3.31 (m, 4H, CH_2_, CH, NH, D_2_O exchangeable), 3.56 (s, 3H, –OCH_3_), 3.82–3.99 (m, 2H, CH_2_), 4.07–4.15 (m, 1H, CH), 7.59–7.69 (m, 5H, 4H, Ar–H, 1H, C5–H), 8.36 (s, 1H, –CH=N–), 8.68 (s, 1H, C2–H), 13.19 (s, 1H, NH, D_2_O exchangeable); ^13^C NMR (101 MHz, DMSO-*d*_*6*_) *δ* ppm: 8.74, 10.14, 22.06, 23.52, 36.87, 39.78, 44.98, 52.31, 56.60, 58.83, 61.50, 106.55, 109.06, 118.74, 123.66, 129.40, 132.23, 134.14, 134.32, 140.91, 146.77, 149.87, 151.98, 153.87, 161.33, 174.33; Anal. Calcd. for C_28_H_29_BrFN_5_O_3_ (582.47): C, 57.74; H, 5.02; N, 12.02; Found: C, 57.93; H, 5.14; N, 12.29.

##### *N'*-((1*H*-Pyrrol-3-yl)methylene)-1-cyclopropyl-6-fluoro-8-methoxy-7-(octahydro-6*H*-pyrrolo[3,4-*b*]pyridin-6-yl)-4-oxo-1,4-dihydroquinoline-3-carbohydrazide (IIIh)

Dark brown powder (yield 20%), m.p. 74–76 °C; IR (KBr, ν cm^−1^): 3430, 3251 (3NH stretching), 3078 (CH aromatic stretching), 2926 (CH aliphatic stretching), 1654 (2C=O stretching); ^1^H NMR (400 MHz, CDCl_3_) *δ* ppm: 0.93–1.43 (m, 4H, 2CH_2_), 1.71–2.00 (m, 4H, 2CH_2_), 2.49–2.57 (m, 1H, CH), 2.90–3.33 (m, 2H, CH_2_), 3.56–3.69 (m, 4H, CH_2_, CH, NH, D_2_O exchangeable), 3.70 (s, 3H, –OCH_3_), 4.07–4.19 (m, 3H, CH_2_, CH), 6.32–6.38 (m, 1H, Ar–H), 6.60 (d, 1H, *J* = 3.60 Hz, Ar–H), 7.09 (s, 1H, Ar–H), 7.41 (s, 1H, NH, D_2_O exchangeable), 7.85 (d, 1H, *J* = 14.30 Hz, C5–H), 8.12 (s, 1H, –CH=N–), 8.95 (s, 1H, C2–H), 13.25 (s, 1H, NH, D_2_O exchangeable); ^13^C NMR (101 MHz, CDCl_3_) *δ* ppm: 8.73, 10.25, 21.27, 23.06, 36.49, 40.00, 44.41, 52.44, 56.20, 57.73, 61.03, 107.68, 109.61, 111.23, 114.75, 122.76, 127.31, 136.50, 139.85, 140.38, 148.94, 152.07, 154.55, 162.03, 174.67, 180.13; Anal. Calc. for C_26_H_29_FN_6_O_3_ (492.56): C, 63.40; H, 5.93; N, 17.06; Found: C, 63.67; H, 6.09; N, 17.24.

##### 1-Cyclopropyl-6-fluoro-*N'*-(furan-3-ylmethylene)-8-methoxy-7-(octahydro-6*H*-pyrrolo[3,4-*b*]pyridin-6-yl)-4-oxo-1,4-dihydroquinoline-3-carbohydrazide (IIIi)

Pale brown powder (yield 56%), m.p. 179–183 °C; IR (KBr, ν cm^−1^): 3448, 3325 (2NH stretching), 3086 (CH aromatic stretching), 2927 (CH aliphatic stretching), 1665 (2C=O stretching); ^1^H NMR (400 MHz, CDCl_3_) *δ* ppm: 0.81–1.29 (m, 4H, 2CH_2_), 1.54–1.85 (m, 4H, 2CH_2_), 2.33–2.39 (m, 1H, CH), 2.69–3.12 (m, 2H, CH_2_), 3.37–3.49 (m, 4H, CH_2_, CH, NH, D_2_O exchangeable), 3.56 (s, 3H, –OCH_3_), 3.39–4.01 (m, 3H, CH_2_, CH), 6.45–6.51 (m, 1H, Ar–H), 6.83 (d, 1H, *J* = 3.40 Hz, Ar–H), 7.53 (s, 1H, Ar–H), 7.75 (d, 1H, *J* = 14.30 Hz, C5–H), 8.12 (s, 1H, –CH=N–), 8.86 (s, 1H, C2–H), 13.22 (s, 1H, NH, D_2_O exchangeable); ^13^C NMR (101 MHz, CDCl_3_) *δ* ppm: 8.75, 10.26, 21.44, 23.12, 36.56, 39.98, 44.54, 52.44, 56.30, 57.85, 61.06, 107.72, 109.67, 111.91, 112.94, 119.57, 133.82, 136.55, 137.47, 140.43, 144.46, 147.40, 149.36, 154.56, 161.99, 174.74; Anal. Calcd. for C_26_H_28_FN_5_O_4_ (493.54): C, 63.27; H, 5.72; N, 14.19; Found: C, 63.14; H, 5.88; N, 14.35.

##### *N'*-((1*H*-Indol-3-yl)methylene)-1-cyclopropyl-6-fluoro-8-methoxy-7-(octahydro-6*H*-pyrrolo[3,4-*b*]pyridin-6-yl)-4-oxo-1,4-dihydroquinoline-3-carbohydrazide (IIIj)

Pale brown powder (yield 36%), m.p. 185–187 °C; IR (KBr, ν cm^−1^): 3387, 3174 (3NH stretching), 3051 (CH aromatic stretching), 2927 (CH aliphatic stretching), 1662 (2C=O stretching); ^1^H NMR (400 MHz, DMSO-*d*_*6*_)* δ* ppm: 0.79–1.26 (m, 4H, 2CH_2_), 1.49–1.83 (m, 4H, 2CH_2_), 2.27–2.34 (m, 1H, CH), 2.65–3.10 (m, 2H, CH_2_), 3.25–3.49 (m, 4H, CH_2_, CH, NH, D_2_O exchangeable), 3.54 (s, 3H, –OCH_3_), 3.87–4.01 (m, 3H, CH_2_, CH), 7.12–7.23 (m, 2H, Ar–H), 7.40 (d, 1H, *J* = 7.70 Hz, Ar–H), 7.53 (s, 1H, Ar–H), 7.78 (d, 1H, *J* = 14.30 Hz, C5–H), 8.26 (d, 1H, *J* = 7.50 Hz, Ar–H), 8.40 (s, 1H, –CH=N–), 8.88 (s, 1H, C2–H), 9.85 (s, 1H, NH, D_2_O exchangeable), 13.08 (s, 1H, NH, D_2_O exchangeable); ^13^C NMR (101 MHz, DMSO-*d*_*6*_) *δ* ppm: 8.71, 10.16, 21.79, 23.25, 36.72, 40.00, 44.79, 52.38, 56.48, 58.24, 60.97, 107.66, 109.97, 111.55, 112.24, 121.13, 121.71, 123.05, 125.17, 128.21, 133.95, 136.47, 136.58, 144.32, 150.13, 152.13, 154.60, 161.51, 174.80, 184.07; Anal. Calcd. for C_30_H_31_FN_6_O_3_ (542.62): C, 66.41; H, 5.76; N, 15.49; Found: C, 66.36; H, 5.89; N, 15.72.

#### Synthesis of 1-cyclopropyl-*N*-(1,3-dioxoisoindolin-2-yl)-6-fluoro-8-methoxy-7-(octahydro-6*H*-pyrrolo[3,4-*b*]pyridin-6-yl)-4-oxo-1,4-dihydroquinoline-3-carboxamide (IV)

A mixture of moxifloxacin hydrazide (**I**) (0.42 g, 1 mmol) and phthalic anhydride (0.15 g, 1 mmol) were dissolved in glacial acetic acid (10 mL) and heated under reflux for 12 h. The solvent was evaporated under vacuum and the residue was crystallized from ethanol to yield compound (**IV**) as brown powder (yield: 80%), m.p. 244–246 °C; IR (KBr, ν cm^−1^): 3441, 3417 (2NH stretching), 3074 (CH aromatic stretching), 2937 (CH aliphatic stretching), 1791, 1736, 1679 (3C=O stretching),; ^1^H NMR (400 MHz, CDCl_3_) *δ* ppm: 1.02–1.14 (m, 4H, 2CH_2_), 1.87–2.11 (m, 4H, 2CH_2_), 2.36–2.38 (m, 1H, CH), 2.66–3.11 (m, 2H, CH_2_), 3.36–3.49 (m, 3H, CH_2_, NH, D_2_O exchangeable), 3.55 (s, 3H, –OCH_3_), 3.89–3.94 (m, 4H, CH, CH, CH_2_), 7.60 (d, 1H, *J* = 14.30 Hz, C5–H), 7.77–7.94 (m, 4H, Ar–H), 8.79 (s, 1H, C2-H), 11.89 (s, 1H, NH, D_2_O exchangeable); ^13^C NMR (101 MHz, CDCl_3_) *δ* ppm: 8.94, 9.80, 18.52, 21.77, 35.04, 40.03, 42.23, 52.59, 54.76, 61.16, 61.44, 108.84, 120.49, 123.83, 130.33, 131.11, 132.78, 133.53, 133.71, 134.48, 135.84, 141.08, 149.74, 151.85, 154.32, 164.58, 165.30, 171.63, 174.43; Anal. Calcd. for C_29_H_28_FN_5_O_5_ (545.57): C, 63.84; H, 5.17; N, 12.84; Found: C, 63.71; H, 5.28; N, 13.06.

#### Synthesis of 9-fluoro-3-methyl-10-(4-methylpiperazin-1-yl)-7-oxo-2,3-dihydro-7*H*-[1,4]oxazino[2,3,4-*ij*]quinoline-6-carbohydrazide (V)

Ofloxacin (**13**) (3.61 g, 10 mol) and hydrazine hydrate (80%, 25 mL) were heated under reflux for 24 h. The reaction mixture was cooled and filtered. The precipitate was washed with water several times, dried and recrystallized from ethanol to afford (**V**) as a yellow powder (yield 40%), m.p. 216–219 °C (reported 238–242 °C)^49^.

#### General procedure for the synthesis of compounds (VIa–c)

Equimolar amounts of ofloxacin carbohydrazide (**V**) (0.19 g, 0.5 mmol) and the appropriate aldehyde (0.5 mmol) were dissolved in absolute ethanol (10 mL) and refluxed for 1–6 h. After cooling, the solvent was removed in vacuum and the residue was crystallized from ethanol yielding the desired compounds (**VIa–c**).

##### 9-Fluoro-*N'*-(4-hydroxy-3-methoxybenzylidene)-3-methyl-10-(4-methylpiperazin-1-yl)-7-oxo-2,3-dihydro-7*H*-[1,4]oxazino[2,3,4-*ij*]quinoline-6-carbohydrazide (VIa).

Brown powder (yield = 41%), m.p. 274–276 °C; IR (KBr, ν cm^−1^); 3417 (OH phenolic stretching), 3282 (NH stretching), 2943 (CH aliphatic stretching), 1643 (2C=O stretching); ^1^H NMR (400 MHz, DMSO-*d*_*6*_) *δ* ppm: 1.50 (d, 3H, *J* = 6.80 Hz, CH_3_), 2.51 (s, 3H, –NCH_3_), 3.21–3.40 (m, 8H, 4CH_2_), 3.86 (s, 3H, –OCH_3_), 4.36 (d, 1H, *J* = 11.10 Hz, CH_2_), 4.49 (d, 1H, *J* = 11.30 Hz, CH_2_), 4.82–4.90 (m, 1H, CH), 6.81–6.87 (m, 1H, Ar–H), 7.12 (d, 1H, *J* = 8.20 Hz, Ar–H), 7.27 (s, 1H, Ar–H), 7.78–8.07 (m, 1H, C5-H), 8.24 (s, 1H, –CH=N–), 8.73 (s, 1H, C2-H), 9.13 (s, 1H, OH, D_2_O exchangeable), 13.37 (s, 1H, NH, D_2_O exchangeable); ^13^C NMR (101 MHz, DMSO-*d*_*6*_) *δ* ppm: 18.43, 54.72, 55.70, 56.02, 56.15, 68.37, 98.64, 109.43, 109.87, 115.74, 116.54, 120.45, 122.62, 126.17, 126.60, 127.12, 127.23, 140.87, 141.64, 144.74, 148.29, 149.39, 161.79, 174.94; Anal. Calcd. for C_26_H_28_FN_5_O_5_ (509.54): C, 61.29; H, 5.54; N, 13.74; Found: C, 62.89; H, 5.61; N, 14.85.

##### *N'*-((1*H*-Pyrrol-3-yl)methylene)-9-fluoro-3-methyl-10-(4-methylpiperazin-1-yl)-7-oxo-2,3-dihydro-7*H*-[1,4]oxazino[2,3,4-*ij*]quinoline-6-carbohydrazide (VIb)

Yellow powder (yield 29%), m.p. 255–259 °C; IR (KBr, ν cm^−1^); 3417, 3290 (2NH stretching), 3105 (CH aromatic stretching), 2943 (CH aliphatic stretching), 1661 (2C=O stretching); ^1^H NMR (400 MHz, DMSO-*d*_*6*_) *δ* ppm: 1.43 (d, 3H, *J* = 6.70 Hz, CH_3_), 2.23 (s, 3H, –NCH_3_), 2.52–3.68 (m, 8H, 4CH_2_), 4.37 (d, 1H, *J* = 11.00 Hz, CH_2_), 4.53 (d, 1H, *J* = 11.50 Hz, CH_2_), 4.58–4.81 (m, 1H, CH), 6.12–6.18 (m, 1H, Ar–H), 6.91 (d, 1H, *J* = 15.50 Hz, Ar–H), 7.84 (s, 1H, Ar–H), 8.01–8.20 (m, 2H, C5–H, –CH=N–), 8.73 (s, 1H, C2–H), 9.08 (s, 1H, NH, D_2_O exchangeable), 13.32 (s, 1H, NH, D_2_O exchangeable); ^13^C NMR (101 MHz, DMSO-*d*_*6*_) *δ* ppm: 18.38, 46.54, 49.48, 54.71, 55.65, 68.33, 109.39, 111.24, 114.04, 120.34, 122.98, 126.47, 127.58, 128.37, 135.41, 140.62, 141.17, 142.96, 144.73, 161.67, 174.86; Anal. Calcd. for C_23_H_25_FN_6_O_3_ (452.49): C, 61.05; H, 5.57; N, 18.57; Found: C, 61.34; H, 5.80; N, 18.79.

##### *N'*-((*1H*-Indol-3-yl)methylene)-9-fluoro-3-methyl-10-(4-methylpiperazin-1-yl)-7-oxo-2,3-dihydro-7*H*-[1,4]oxazino[2,3,4-*ij*]quinoline-6-carbohydrazide (VIc)

Brown powder (Yield 16%), m.p. 229–231 °C; IR (KBr, ν cm^−1^); 3387, 3213 (2NH stretching), 3059 (CH aromatic stretching), 2927 (CH aliphatic stretching), 1705, 1680 (2C=O stretching); ^1^H NMR (400 MHz, DMSO-*d*_*6*_) *δ* ppm: 1.45 (d, 3H, *J* = 6.70 Hz, CH_3_), 2.29 (s, 3H, –NCH_3_), 2.59–2.87 (m, 4H, 2CH_2_), 3.35–3.59 (m, 4H, 2CH_2_), 4.36 (dd, 1H,* J* = 11.60, 2.50 Hz, CH_2_), 4.50 (dd, 1H, *J* = 11.50, 2.00 Hz, CH_2_), 4.75–4.85 (m, 1H, CH), 7.19–7.25 (m, 2H, Ar–H), 7.44–7.48 (m, 1H, Ar–H), 7.67–7.71 (m, 1H, C5–H), 7.81 (s, 1H, Ar–H), 8.27–8.36 (m, 1H, Ar–H), 8.64 (s, 1H, –CH=N–), 9.02 (s, 1H, C2-H), 10.90 (s, 1H, NH, D_2_O exchangeable), 11.45 (s, 1H, NH, D_2_O exchangeable); ^13^C NMR (101 MHz, DMSO-*d*_*6*_) *δ* ppm: 18.36, 46.57, 49.49, 54.45, 55.82, 68.34, 97.77, 109.48, 112.33, 112.77, 120.20, 120.68, 121.97, 122.93, 124.72, 126.67, 127.13, 128.71, 137.55, 139.45, 141.19, 142.24, 144.70, 161.36, 164.81, 174.42; Anal. Calcd. for C_27_H_27_FN_6_O_3_ (502.55): C, 64.53; H, 5.42; N, 16.72; Found: C, 64.76; H, 5.57; N, 16.89.

#### Synthesis of 1-cyclopropyl-6-fluoro-4-oxo-7-(piperazin-1-yl)-1,4-dihydroquinoline-3-carbohydrazide (VII)

Ciprofloxacin (**7**) (3.31 g, 10 mmol) and hydrazine hydrate (80%, 25 mL) were heated under reflux for 24 h. The reaction mixture was cooled, then the formed precipitate was filtered and washed several times with water. After it was dried, recrystallization was performed using ethanol to afford (**VII**) as a yellow-white powder (yield 30%), m.p. 238–242 °C (reported 238–242 °C)^50^.

#### General procedure for the synthesis of compounds (VIIIa–c)

Equimolar amounts of ciprofloxacin carbohydrazide (**VII**) (0.17 g, 0.5 mmol) and the appropriate aldehyde (0.5 mol) were dissolved in absolute ethanol (10 mL) and heated under reflux for 2–5 h. After cooling, the formed precipitate was filtered, dried and recrystallized from ethanol to yield a solid powder (**VIIIa–c**).

##### 1-Cyclopropyl-6-fluoro-*N'*-(4-hydroxy-3-methoxybenzylidene)-4-oxo-7-(piperazin-1-yl)-1,4-dihydroquinoline-3-carbohydrazide (VIIIa)^51^

*N'*-((1*H*-Pyrrol-3-yl)methylene)-1-cyclopropyl-6-fluoro-4-oxo-7-(piperazin-1-yl)-1,4-dihydroquinoline-3-carbohydrazide (**VIIIb**).

Yellow powder (yield 23%), m.p. 165–170 °C; IR (KBr, ν cm^−1^): 3387, 3221 (3NH stretching), 3105 (CH aromatic stretching), 2951 (CH aliphatic stretching), 1659 (2C=O stretching); ^1^H NMR (400 MHz, DMSO-*d*_*6*_) *δ* ppm: 1.06–1.11 (m, 2H, CH_2_), 1.26–1.31 (m, 2H, CH_2_), 2.88–2.93 (m, 4H, 2CH_2_), 3.14–3.18 (m, 4H, 2CH_2_), 3.67–3.73 (m, 2H, CH, NH, D_2_O exchangeable), 6.16–6.20 (m, 1H, Ar–H), 6.60 (s, 1H, Ar–H), 6.99 (s, 1H, C8–H), 7.41 (d, 1H, *J* = 7.40 Hz, Ar–H), 7.76 (d, 1H, *J* = 13.50 Hz, C5–H), 8.38 (s, 1H, –CH=N–), 8.56 (s, 1H, C2-H), 10.58 (s, 1H, NH, D_2_O exchangeable), 11.57 (s, 1H, NH, D_2_O exchangeable); ^13^C NMR (101 MHz, DMSO-*d*_*6*_) *δ* ppm: 8.01, 35.41, 45.75, 51.11, 106.14, 108.62, 110.17, 111.46, 115.26, 117.60, 123.71, 127.82, 138.78, 145.66, 146.49, 151.06, 152.68, 164.13, 174.27; Anal. Calcd. for C_22_H_23_FN_6_O_2_ (422.46): C, 62.55; H, 5.49; N, 19.89; Found: C, 62.79; H, 5.32; N, 19.72.

##### *N'*-((1*H*-Indol-3-yl)methylene)-1-cyclopropyl-6-fluoro-4-oxo-7-(piperazin-1-yl)-1,4-dihydroquinoline-3-carbohydrazide (VIIIc)

Buff powder (yield 38%), m.p. 297–300 °C; IR (KBr, ν cm^−1^): 3498, 3240, 3178 (3NH stretching), 3043 (CH aromatic stretching), 2924 (CH aliphatic stretching), 1668 (2C=O stretching); ^1^H NMR (400 MHz, DMSO-*d*_*6*_) *δ* ppm: 1.10–1.20 (m, 2H, CH_2_), 1.26–1.38 (m, 2H, CH_2_), 2.86–2.93 (m, 4H, 2CH_2_), 3.14–3.21 (m, 4H, 2CH_2_), 3.45 (s, 1H, NH, D_2_O exchangeable), 3.75–3.85 (m, 1H, CH), 7.14–7.24 (m, 2H, Ar–H), 7.44–7.50 (m, 2H, C8–H, Ar–H), 7.80 (s, 1H, Ar–H), 7.84–7.91 (m, 1H, Ar–H), 8.29 (d, 1H, *J* = 13.50 Hz, C5–H), 8.51 (s, 1H, –CH=N–), 8.70 (s, 1H, C2-H), 11.59 (s, 1H, NH, D_2_O exchangeable), 12.93 (s, 1H, NH, D_2_O exchangeable); ^13^C NMR (101 MHz, DMSO-*d*_*6*_) *δ* ppm: 8.04, 18.99, 35.68, 45.85, 51.27, 56.51, 106.30, 110.18, 111.53, 112.12, 121.00, 122.56, 123.10, 124.81, 130.94, 132.27, 137.46, 138.95, 145.14, 147.25, 152.69, 153.99, 155.56, 160.61, 174.59; Anal. Calcd. for C_26_H_25_FN_6_O_2_ (472.52): C, 66.09; H, 5.33; N, 17.79; Found: C, 66.27; H, 5.48; N, 18.01.

### Biological evaluation

#### NCI-60 cytotoxicity assay^52^

The 60-cell line screening was done using sulforhodamine B (SRB) protein assay at the Developmental Therapeutic Program, National Cancer Institute, Bethesda, USA^52–55^. The first evaluation uses a single dose concentration (10^–5^ Molar). Cancer cell lines of the screening panel are grown in RPMI 1640 medium containing 5% fetal bovine serum and 2 mM l-glutamine. Cells are inoculated into 96 well microtiter plates in 100 μL at plating densities ranging from 5000 to 40,000 cells/well, depending on the doubling time of individual cell lines. Plates are incubated at 37 °C, with 5% CO_2_, 95% air, and 100% relative humidity for 24 h before the addition of the experimental drug. After 24 h, two plates of each cell line are fixed in situ with trichloroacetic acid (TCA) to represent a measurement of the cell population for each cell line at the time of drug addition (Tz). The compounds are dissolved in dimethyl sulfoxide (DMSO) at 400-fold the desired final maximum test concentration and stored frozen before use. On use, the frozen concentrate is thawed and diluted to twice the desired final maximum test concentration with a complete medium containing 50 μg/mL gentamicin. Plates are incubated for an additional 48 h at 37 °C, with 5% CO_2_, 95% air, and 100% relative humidity. Cells are fixed in situ by adding 50 μL of cold 50% (w/v) TCA (final concentration, 10% TCA) and incubated for 60 min at 4 °C. The supernatant is discarded, and the plates are washed with tap water and air-dried five times. Then, SRB solution (100 μL) at 0.4% (w/v) in 1% acetic acid was added to each well and incubated for 10 min at room temperature. Unbound dye is removed by washing five times with 1% acetic acid and the plates are air-dried. The bound stain is dissolved with a 10 mM trizma base, and the absorbance is read on an automated plate reader at a wavelength of 515 nm. Using the seven absorbance measurements [time zero, (Tz), control growth, (C), and test growth in the presence of the drug at the five concentration levels (Ti)], the percentage growth is calculated at each of the drug concentration levels. Percentage growth inhibition is calculated as (Ti − Tz)/(C − Tz) × 100 (for concentrations for which Ti >/= Tz) and (Ti − Tz)/Tz × 100 (for concentrations for which Ti < Tz). The output from the single-dose screen is reported as a mean graph and is available for analysis by the COMPARE program. In which, Pearson correlation coefficient is calculated using commercial statistical package procedure (the SAS procedure PROC CORR) for each compound in the database. Those compounds with the highest correlation coefficient are most similar to the seed (https://dtp.cancer.gov/databases_tools/docs/compare/compare_methodology.htm)^55^.

#### NCI-60 five-dose assay

The most potent two compounds were selected to undergo five-dose testing (10^–4^, 10^–5^, 10^–6^, 10^–7^ and 10^–8^ Molar) using the same steps as before to provide five drug concentrations and control. Three dose response parameters are calculated for each experimental agent. Growth inhibition of 50% (GI_50_) is calculated from [(Ti − Tz)/(C − Tz)] × 100 = 50, which is the drug concentration resulting in a 50% reduction in the net protein increase (as measured by SRB staining) in control cells during the drug incubation. The drug concentration resulting in total growth inhibition (TGI) is calculated from Ti = Tz. The LC_50_ (concentration of drug resulting in a 50% reduction in the measured protein at the end of the drug treatment as compared to that at the beginning) indicating a net loss of cells following treatment is calculated from [(Ti − Tz)/Tz] × 100 =  − 50. Values are calculated for each of these three parameters if the level of activity is reached; however, if the effect is not reached or is exceeded, the value for that parameter is expressed as greater or less than the maximum or minimum concentration tested.

#### Topoisomerase I & II enzyme inhibition assay^56^

Compounds (**IIIf**) and (**VIb**) were tested for their ability to inhibit DNA topoisomerase I & II-mediated relaxation of supercoiled DNA pBR322 in vitro using the described method at two concentrations (50 and 100 µM). DNA bands were visualized by transillumination with UV light and quantitated using AlphaImagerTM (Alpha Innotech Corporation)^56^.

#### Evaluation of selective cytotoxicity^57–59^

Vero cells (African Green Monkey Kidney cell line) were obtained from the American Type Culture Collection (ATCC, Rockville, MD)^59^. The cells were propagated via Dulbecco’s modified Eagle’s medium (DMEM) supplemented with 10% heat-inactivated Fetal Bovine Serum, 1% l-glutamine, HEPES buffer, and 50 µg/mL gentamycin. All cells were maintained at 37 °C in a humidified atmosphere with 5% CO_2_ and were sub-cultured two times a week. MTT assay was done after the incubation of cells to estimate the cytotoxicity^57,58^. The experiment was repeated using serial dilutions of (**IIIf**) and (**VIb**) and the relation between surviving cells and drug concentration was plotted to get the survival curve of each case after treatment. The Cytotoxic concentration (CC_50_) required to cause toxic effects in 50% of intact cells was estimated from graphic plots of the dose–response curve for each concentration using Graphpad Prism software (San Diego, CA, USA). The experiment was repeated, and data are presented with mean standard deviation for triplicates.

#### Cell cycle analysis (DNA-flow cytometry analysis)^60,61^

MCF-7 cells were exposed to (**IIIf**) and (**VIb**) at their respective GI_50_ and analyzed using CycleTEST™ PLUS DNA Reagent Kit (Becton Dickinson Immunocytometry Systems, San Jose, CA). After following the procedure provided by the kit, the cells were stained with propodium iodide (PI) stain and then ran on the flow cytometer. Each sample's cell distribution was quantitatively examined using at least 10,000 cells per sample (data are presented with mean standard deviation for triplicates). Cell-cycle distribution was calculated using Cell Quest software 5.2.1 (Becton Dickinson Immunocytometry Systems, San Jose, CA)^60,61^.

#### Apoptotic analysis (annexin V-FITC assay)^52,62,63^

MCF-7 was used again in this assay to analyze the apoptotic effects of (**IIIf**) and (**VIb**) at their respective GI_50_. The cells were cultured to a confluent monolayer and treated with the compounds at their respective GI_50_ concentration as described earlier. After treatment for 24 h, the cells were then harvested and rinsed twice in phosphate buffer saline (PBS) for 20 min. each, followed by the binding buffer. Moreover, MCF-7 cells (treated or non-treated) were re-suspended in 100 µL of kit binding buffer with the addition of 1 µL of FITC-Annexin V (Becton Dickinson BD Pharmingen™, Heidelberg, Germany) followed by 40 min. incubation at 4 °C. Cells were then washed and re-suspended in 150 µL of binding buffer with the addition of 1 µL of 4′,6-diamidino-2-phenylindole (DAPI) (1 µg/mL in PBS) (Invitrogen, Life Technologies, Darmstadt, Germany). Then, the cells were analyzed using the flow cytometer BD FACS Calibur (BD Biosciences, San Jose, CA)^52,62,63^. Data are presented with mean standard deviation for triplicates.

#### Western blot analysis (Bax/Bcl-2)^64–66^

MCF-7 cells were treated and incubated with (**IIIf**) and (**VIb**) at their respective GI_50_ for 24 h. After incubation, the experiment was terminated, cells were lysed, and the total protein concentrations were determined colorimetrically in the supernatant using the Bradford method before proceeding to the western blotting. Then the protein samples were loaded into SDS–polyacrylamide gel and separated by the Cleaver electrophoresis unit (Cleaver, UK). The gel was then processed, and the chemiluminescent signals were captured using a CCD camera-based imager (Chemi Doc imager, Biorad, USA). The band intensities were then measured with β-actin as an internal reference protein^64–66^.

#### ELISA analysis (caspase-8/9)^67^

MCF-7 cells were cultured to a monolayer and then treated with compounds at the GI_50_ concentration as described earlier. Cells were then harvested through trypsinization and rinsed twice in PBS. The levels of the apoptotic markers caspase-8 and caspase-9 were assessed using ELISA colorimetric kits per the manufacturer's instructions, as reported earlier^67^. The experiment was repeated, and data are presented with mean standard deviation for triplicates.

### In-silico study

#### Pharmacokinetic profiling

The compounds were drawn using the built-in sketcher of Biovia Discovery Studio DS (www.3ds.com/products-services/biovia), and the ADMET simulation was performed using default parameters. Additionally, chemicalize (www.chemicalize.org) was employed to analyze the physiochemical parameters^46^.

#### Docking study

Docking was performed on (**IIIf**) and (**VIb**) on human topoisomerase II using MOE (Molecular Operating Environment, www.chemcomp.com) 2008.10 software^47^. The receptor file was obtained from Protein Data Bank (PDB ID: 6ZY7). The standard protocol for structure optimization was used to prepare the receptors and ligands, followed by energy minimization under AMBER12: EHT force field. Using the DOCK wizard, the active site was set to the pocket surrounding the co-crystallized ligand and the ligand placement through London dG and GBVI/WSA dG. The co-crystallized ligand, etoposide, was used for docking validation and as a reference for our compounds. The docking experiments' validation process was achieved by re-docking the co-crystalized ligand into its corresponding active site, and RMSD was also calculated. The obtained results were visualized and analyzed by DS visualizer available from Biovia Inc^68^.

## Supplementary Information

Supplementary Information.

## Data Availability

All data generated or analyzed during this study are included in this published article (and its Supplementary Information files).
