# Supplementary material for: Design and synthesis of novel cytotoxic fluoroquinolone analogs through topoisomerase inhibition, cell cycle arrest, and apoptosis
Source: Sci Rep. 2023 Mar 13;13:4144. doi: 10.1038/s41598-023-30885-5 (PMC10011602; doi:10.1038/s41598-023-30885-5)
Supplement: Supplementary file 1 — Supplementary Information. [file 41598_2023_30885_MOESM1_ESM.docx]

**Design and synthesis of novel cytotoxic fluoroquinolone analogs through topoisomerase inhibition, cell cycle arrest, and apoptosis**

Mohamed Adel Elanany^a*^, Essam Eldin A. Osman^b^, Ehab Mohamed Gedawy^a,c^, Sahar M. Abou-Seri^b*^

*^a^Department of Pharmaceutical Chemistry, School of Pharmacy and Pharmaceutical Industries, Badr University in Cairo (BUC), Badr City, Cairo 11829, Egypt.*

*^b^Department of Pharmaceutical Chemistry, Faculty of Pharmacy, Cairo University, Cairo 11562, Egypt.*

*^c^Department of Pharmaceutical Organic Chemistry, Faculty of Pharmacy, Cairo University, Cairo 11562, Egypt.*

**Corresponding author:* [*mohamed.elanany@buc.edu.eg*](mailto:mohamed.elanany@buc.edu.eg) *(Mohamed A. Elanany);* [*sahar.shaarawy@pharma.cu.edu.eg*](mailto:sahar.shaarawy@pharma.cu.edu.eg) *(Sahar M. Abou-Seri)*

*Tel.:(+20)1004816309*

**Supplementary data**

| Contents | Caption | Pages |
| --- | --- | --- |
| 1. Compounds analysis data | Figure 1S – 62S | **2 – 34** |
| 1. NCI-60 Human Tumor Cell line single-dose screening Percentage growth inhibition results | Table 1S, 2S | **35 – 40** |
| 1. COMPARE analysis results | Figure 63S, 64S | **41** |
| 1. Molecular docking results | Figure 65S, 66S | **42 - 43** |
| 1. NCI-60 Human Tumor Cell Lines Screening single-dose assay results | Figure 67S – 85S | **43 - 61** |
| 1. NCI-60 Human Tumor Cell Lines Screening five-dose assay results | Figure 86S – 91S | **62 – 67** |
| 1. Original gel for recombinant Topo I of (IIIf) and (VIb). | Figure 92S | **68** |
| 1. Original gel for recombinant Topo II of (IIIf) and (VIb). | Figure 93S | **69** |

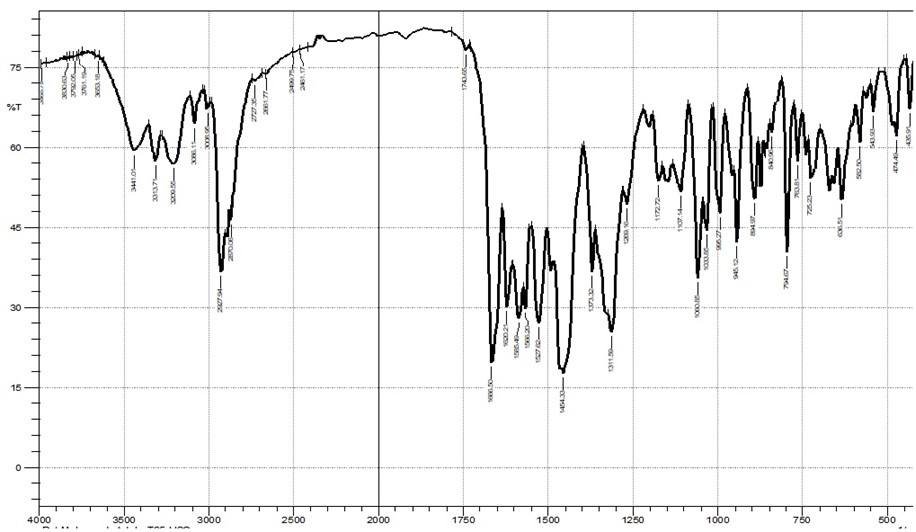


Figure 1S. IR spectrum of **I**.


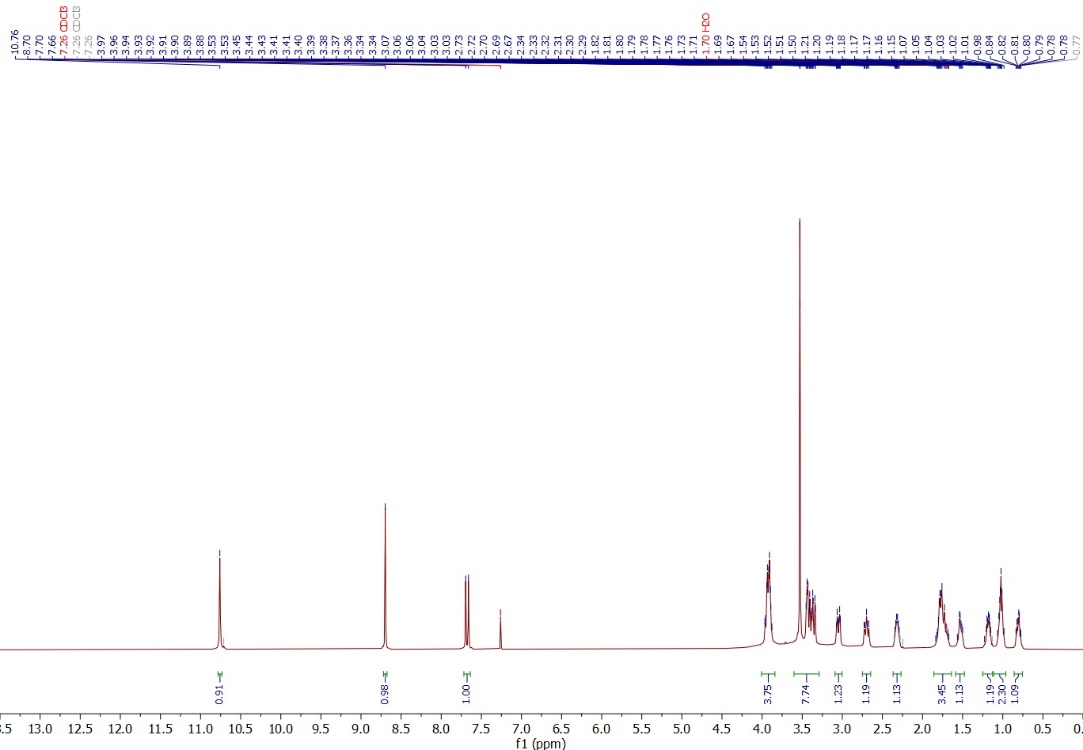


Figure 2S. ^1^H NMR spectrum of **I**.


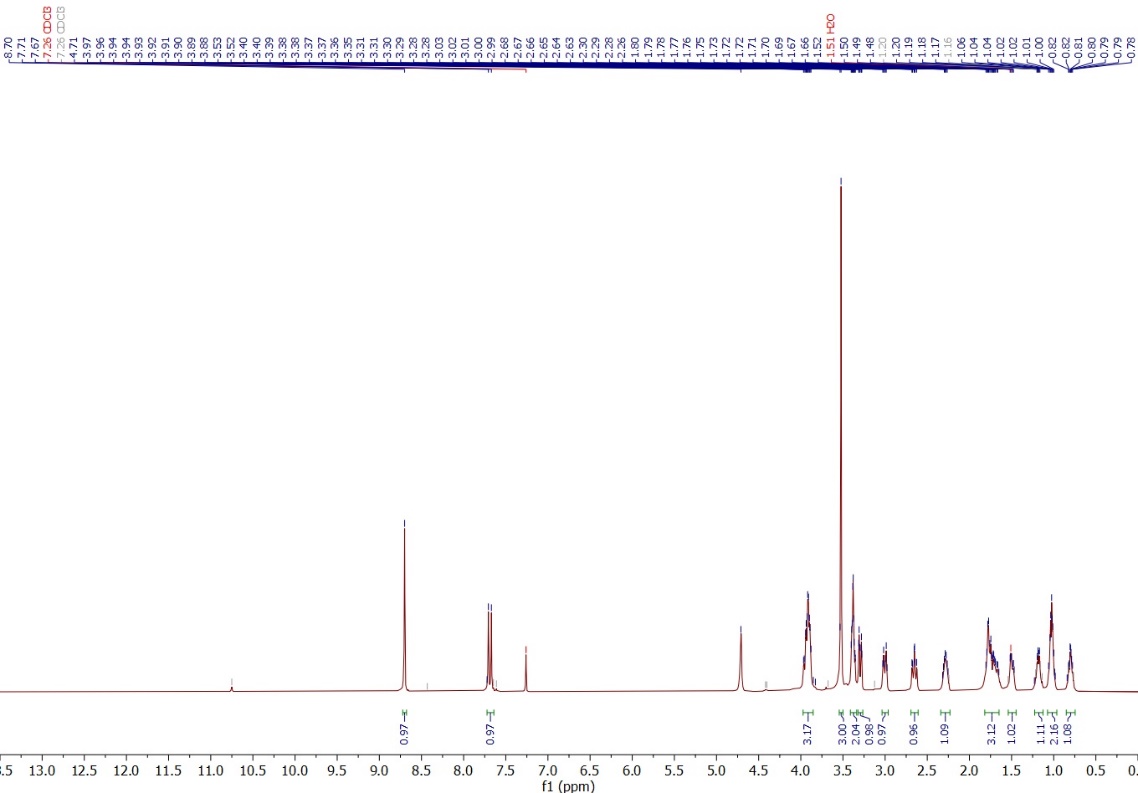


Figure 3S. ^1^H NMR (D_2_O) spectrum of **I**.


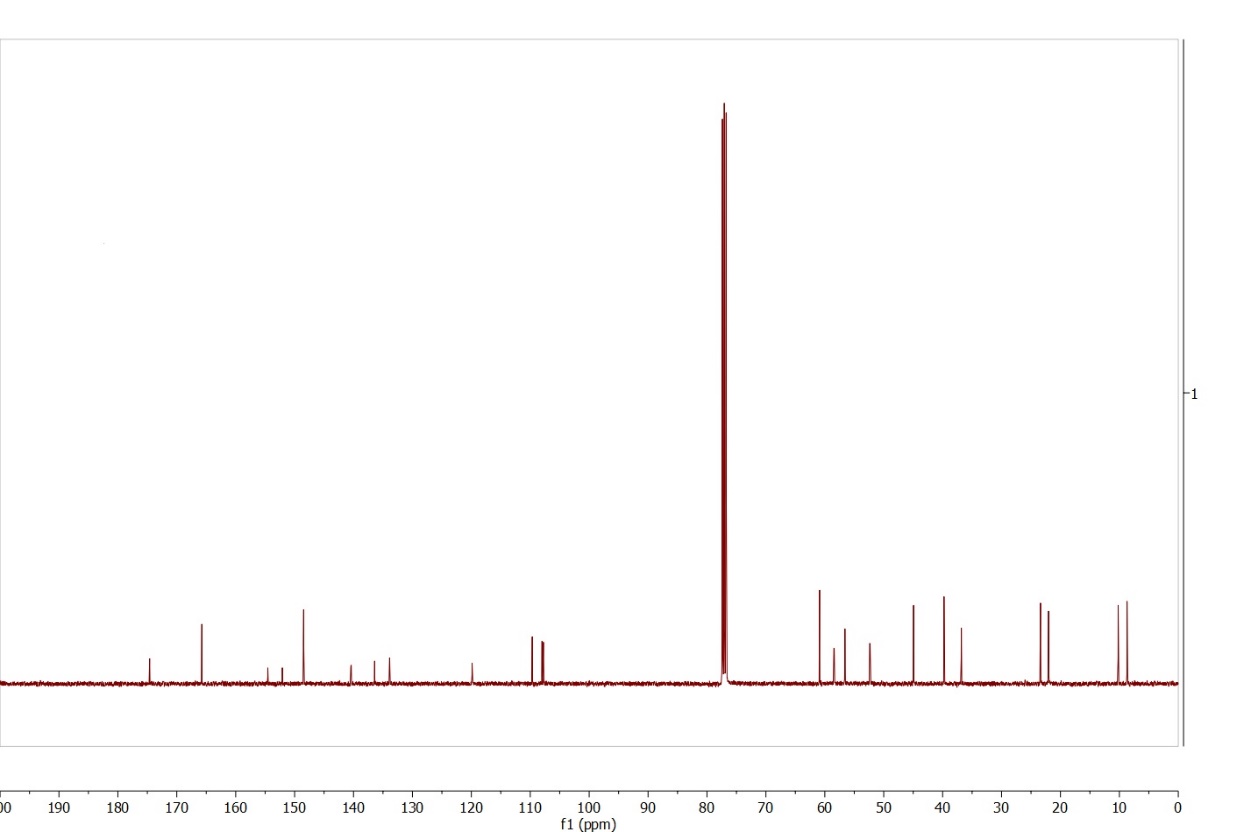


Figure 4S. ^13^C NMR spectrum of **I**

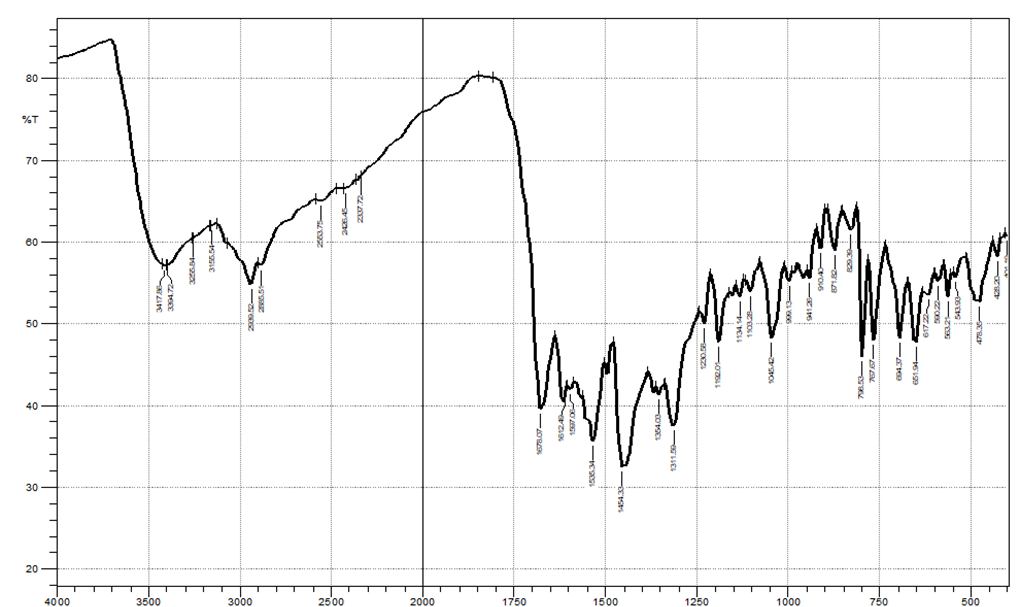

Figure 5S. IR spectrum of **II**.


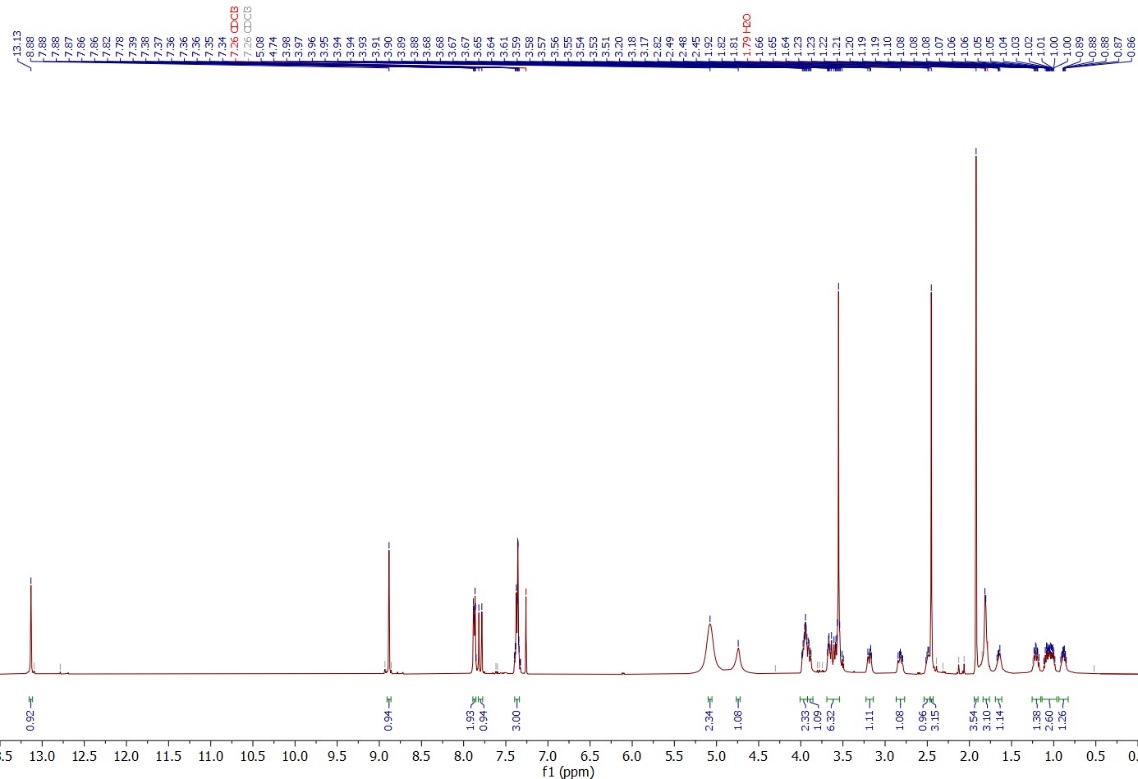


Figure 6S. ^1^H NMR spectrum of **II**.


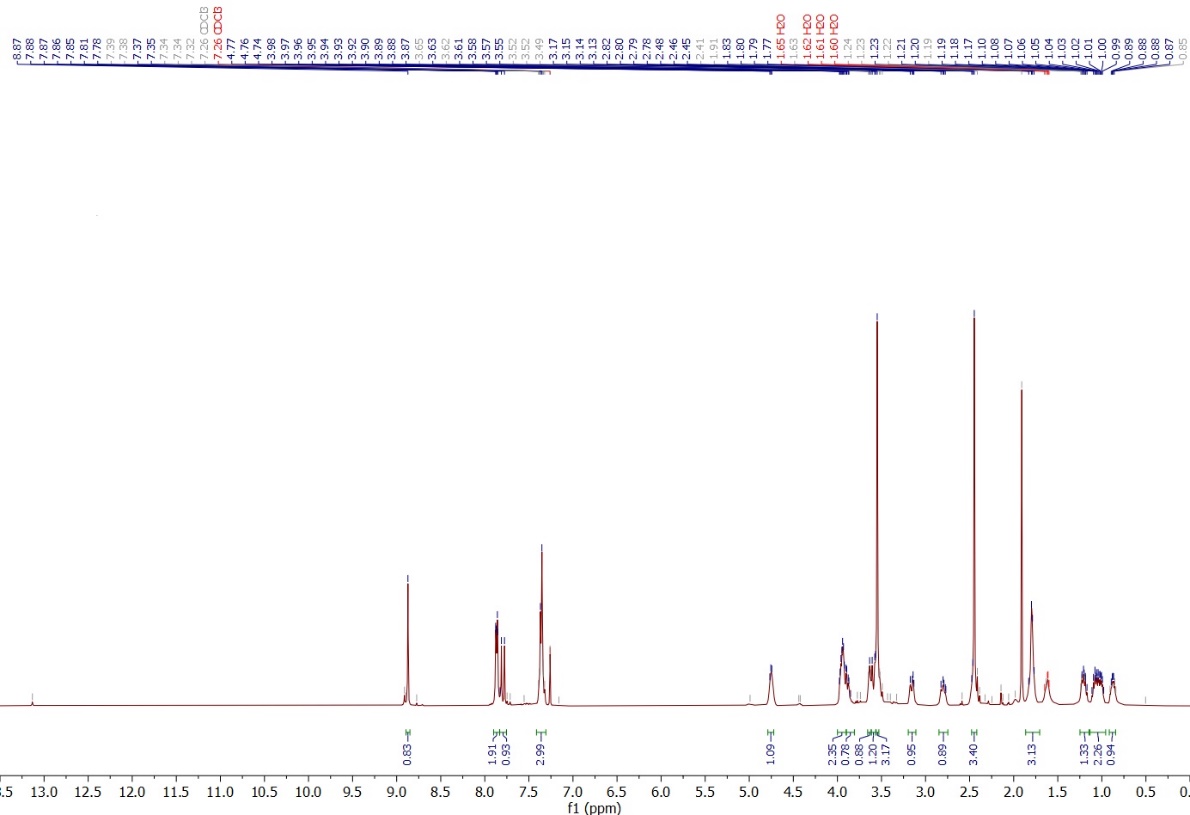


Figure 7S. ^1^H NMR (D_2_O) spectrum of **II**.


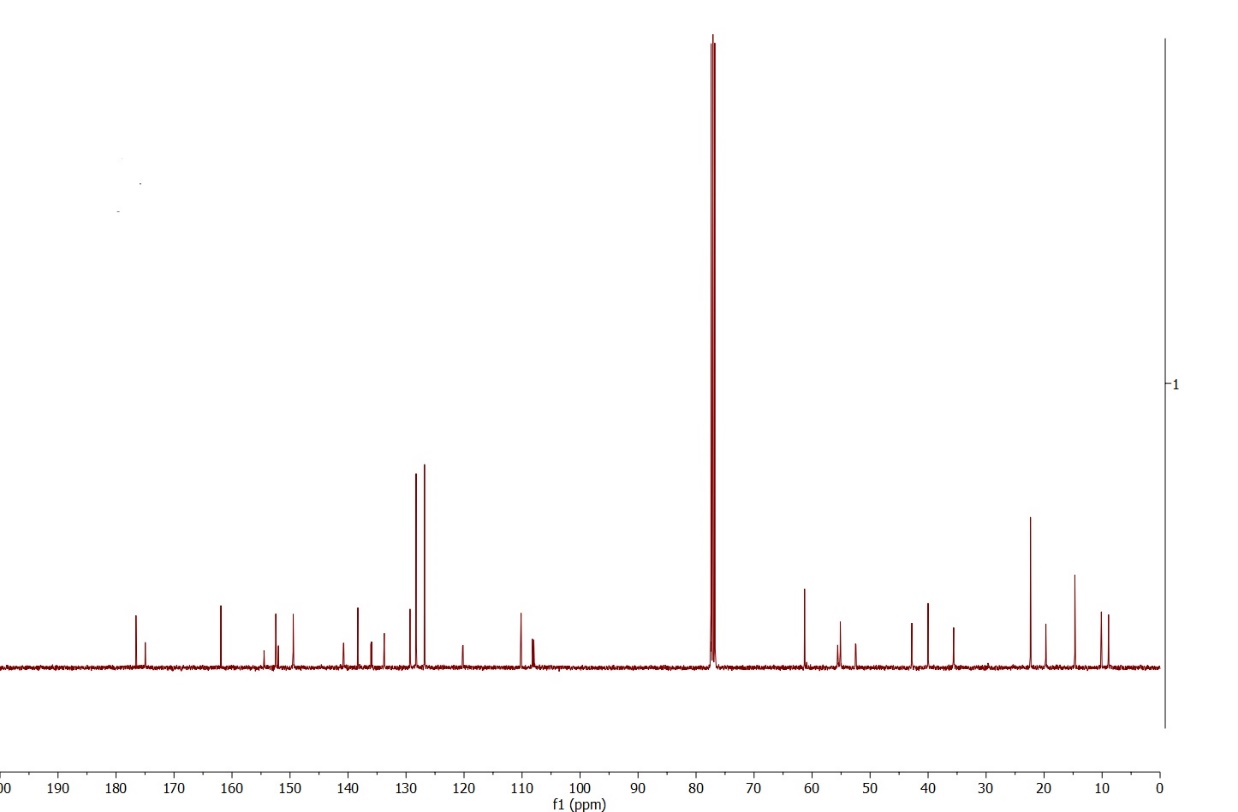


Figure 8S. ^13^C NMR spectrum of **II**.

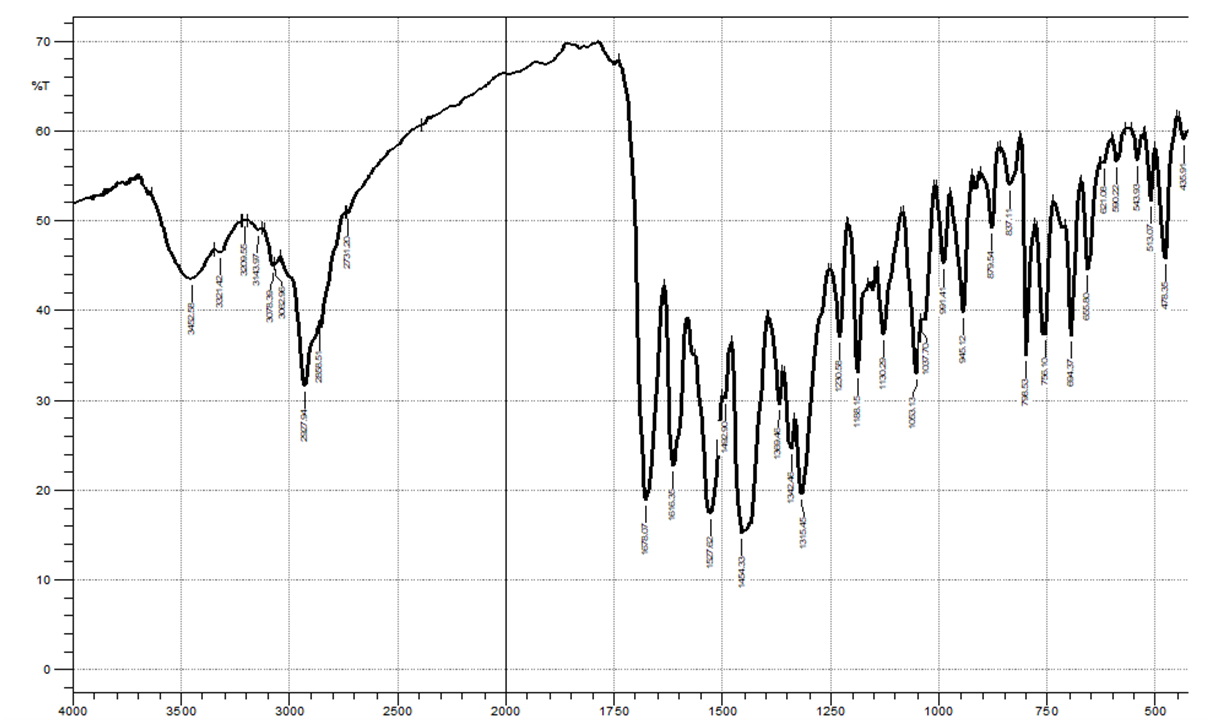

Figure 9S. IR spectrum of **IIIa**.


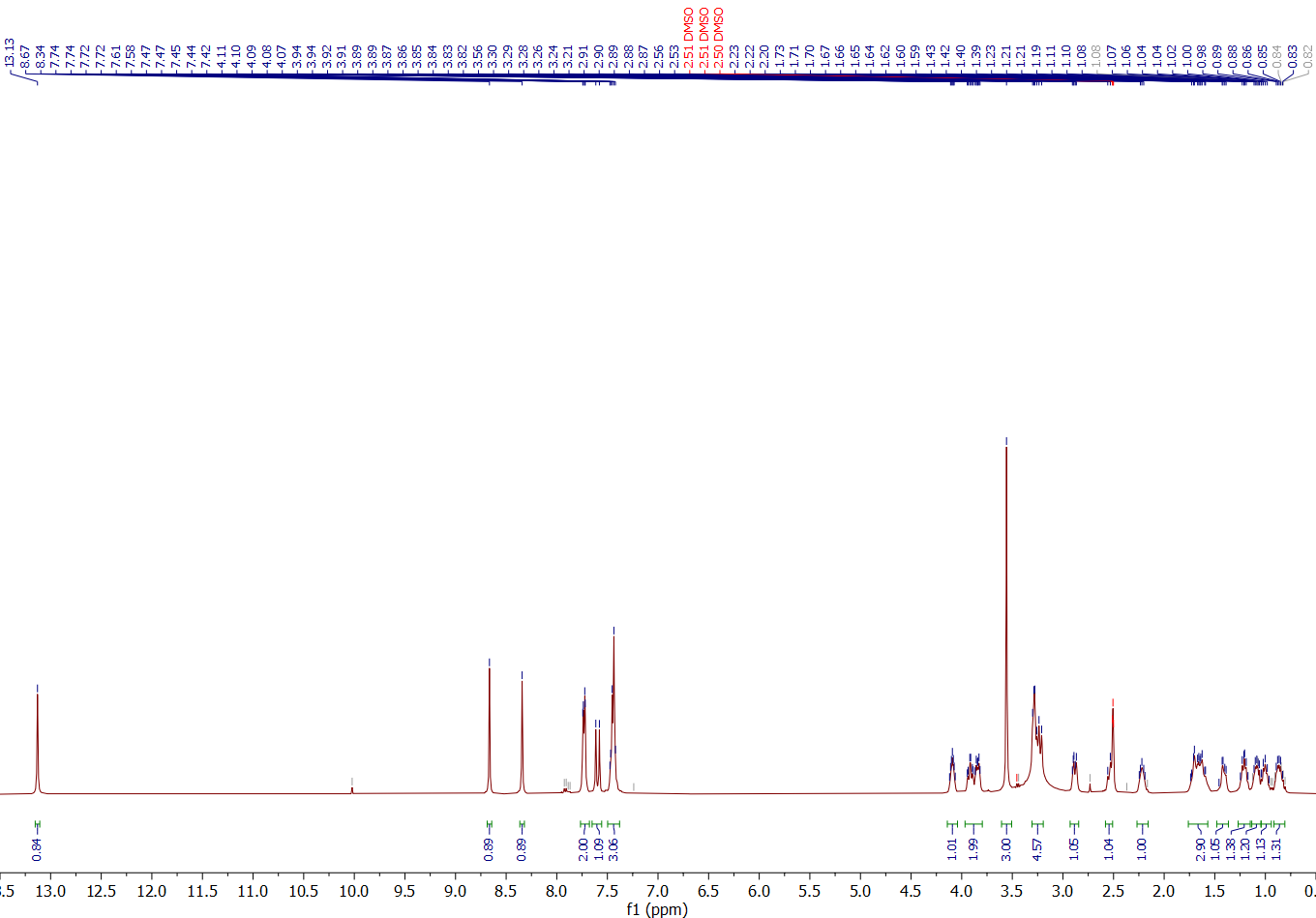


Figure 10S. ^1^H NMR spectrum of **IIIa**.


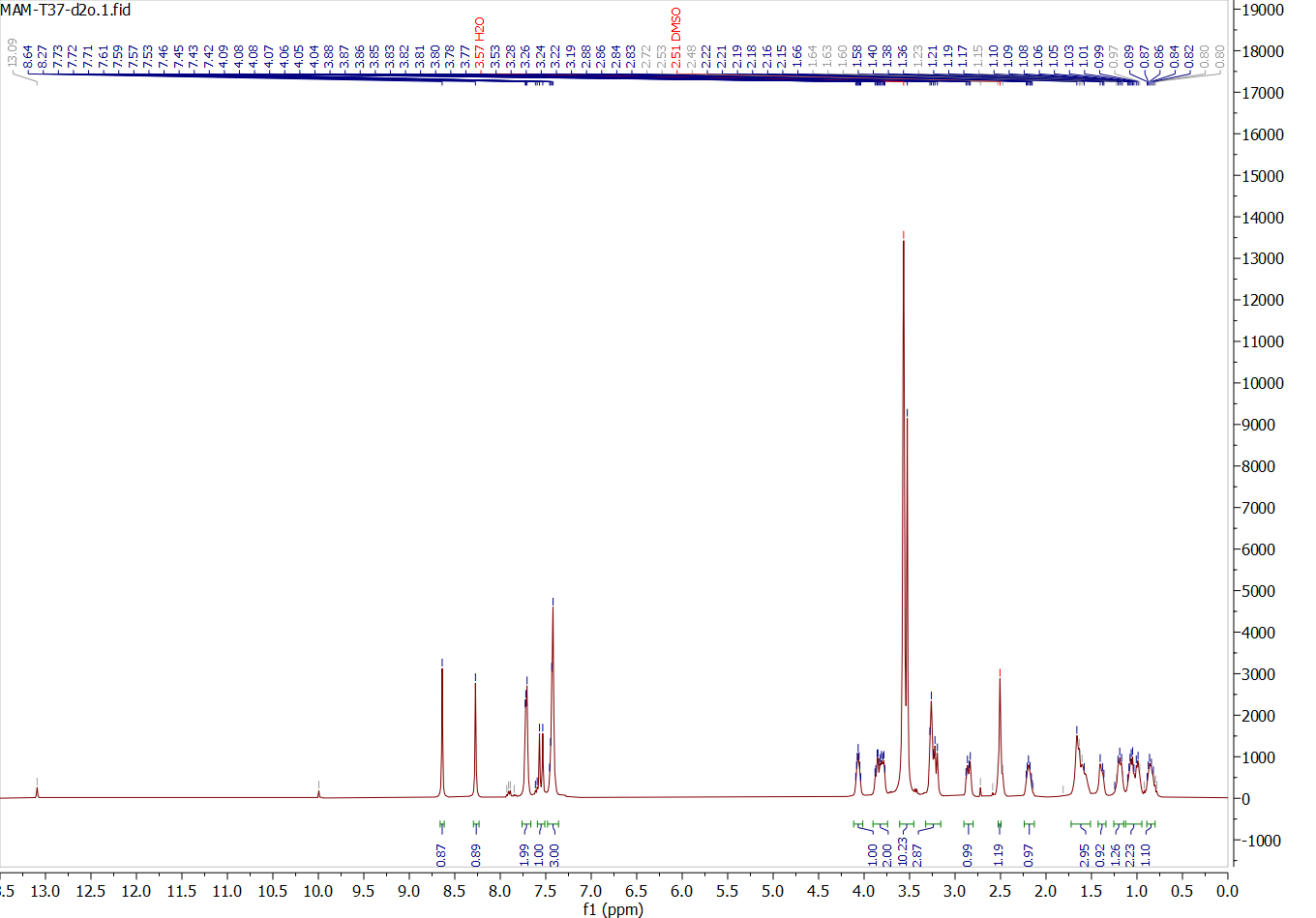

Figure 11S. ^1^H NMR (D_2_O) spectrum of **IIIa**.
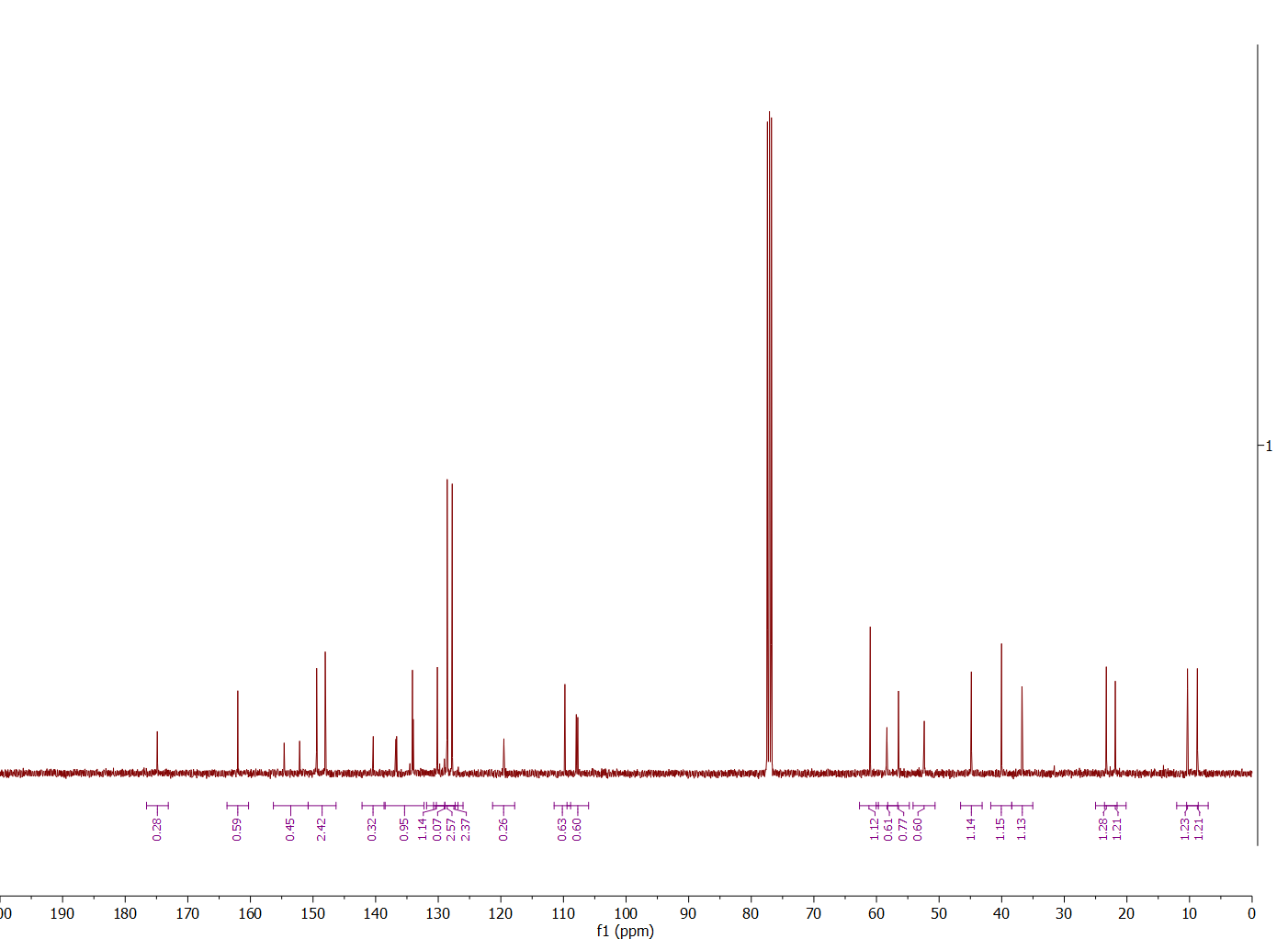


Figure 12S. ^13^C NMR spectrum of **IIIa**.

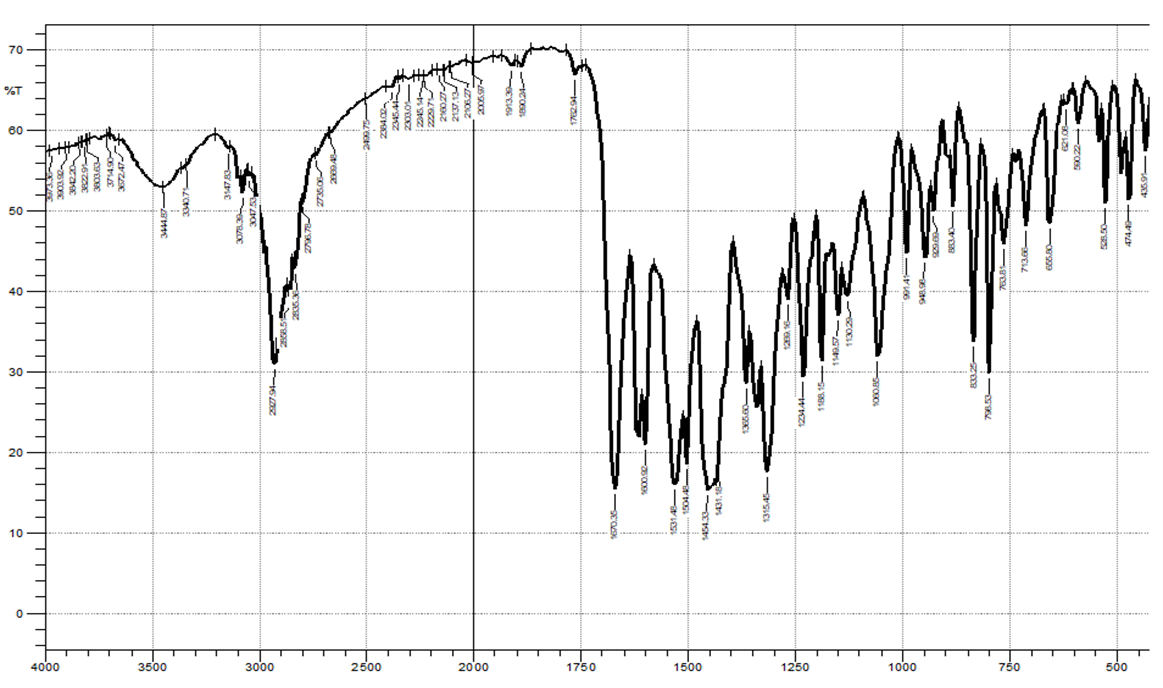


Figure 13S. IR spectrum of **IIIb**.


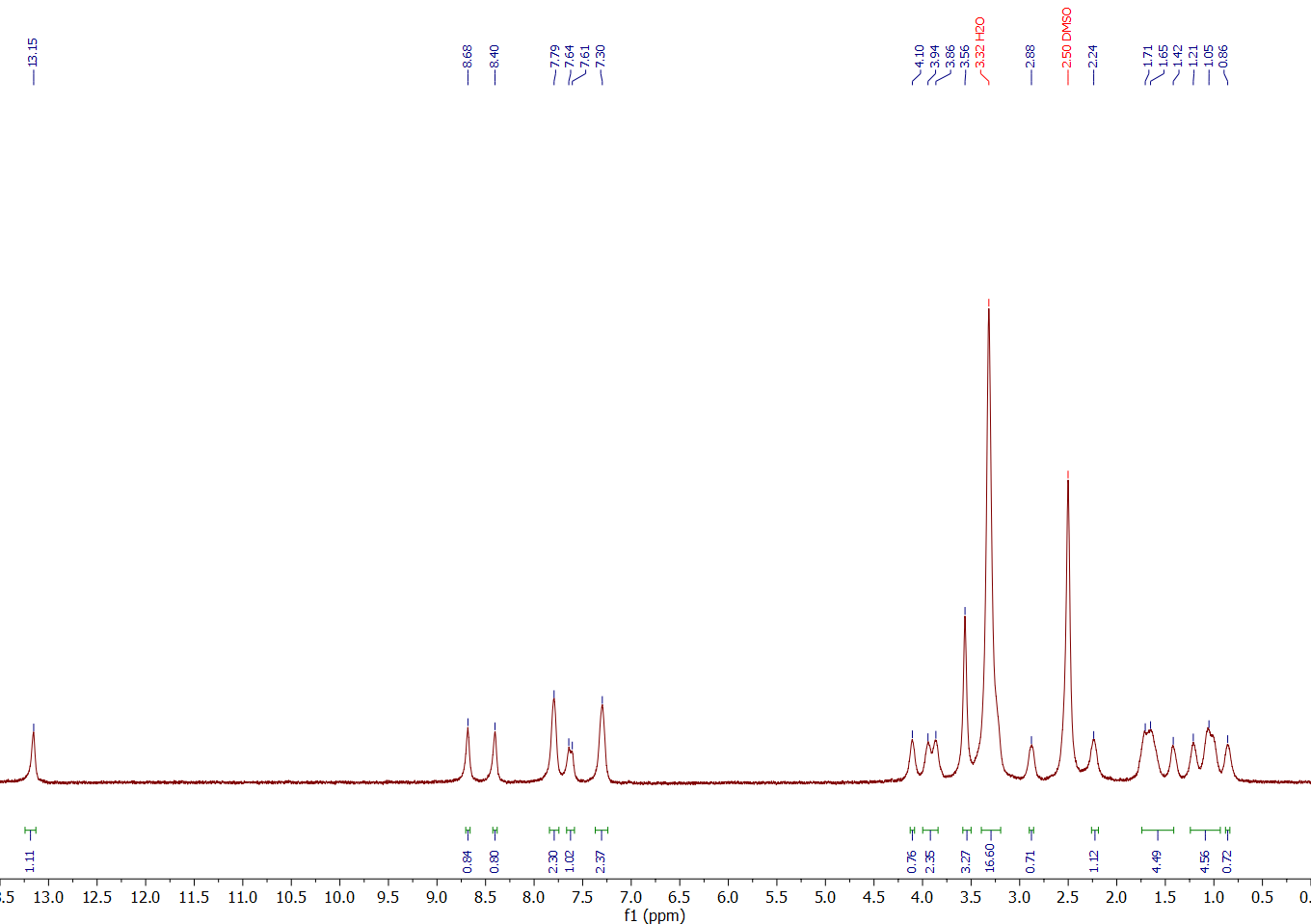


Figure 14S. ^1^H NMR spectrum of **IIIb**.


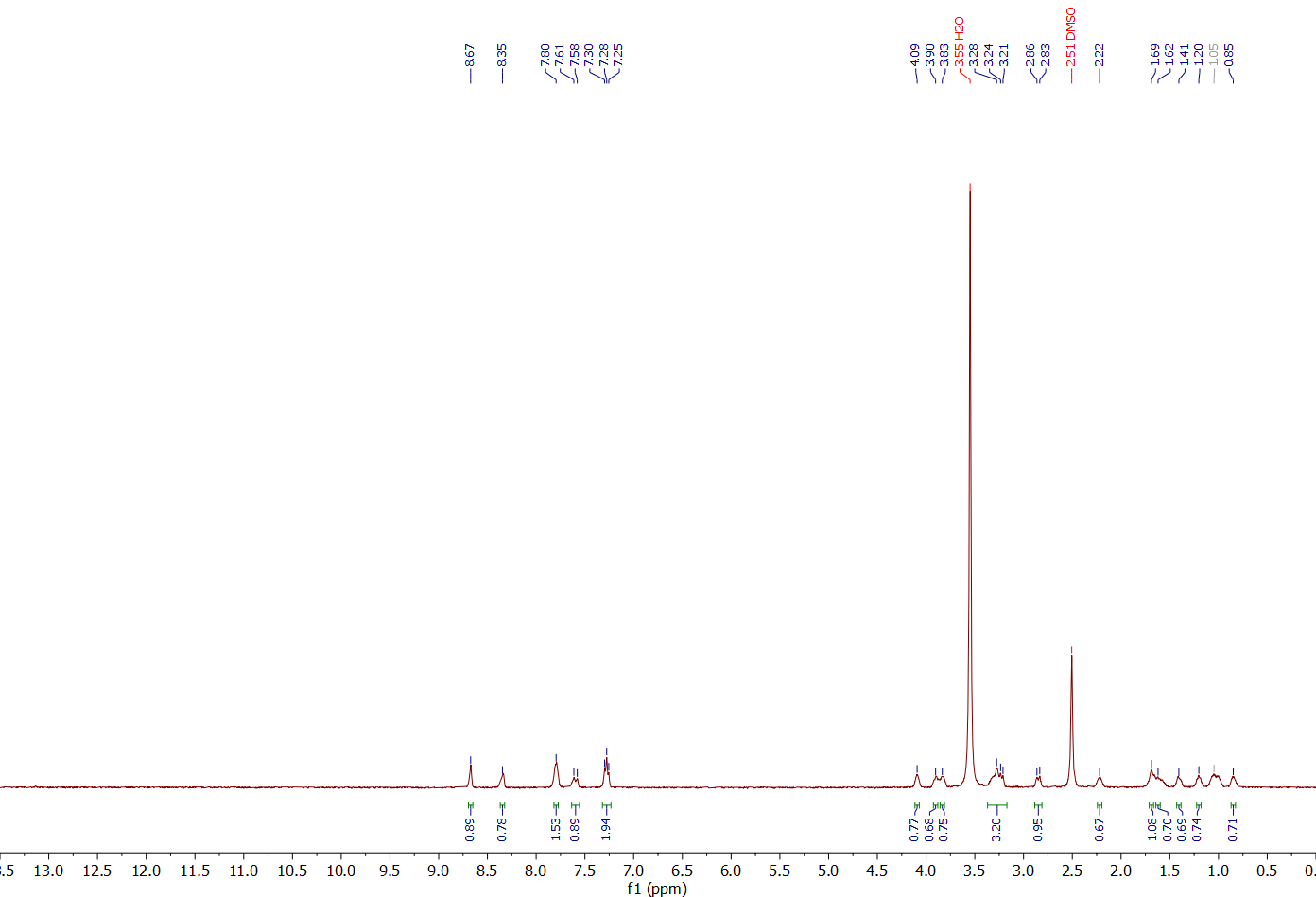


Figure 15S. ^1^H NMR (D_2_O) spectrum of **IIIb**.


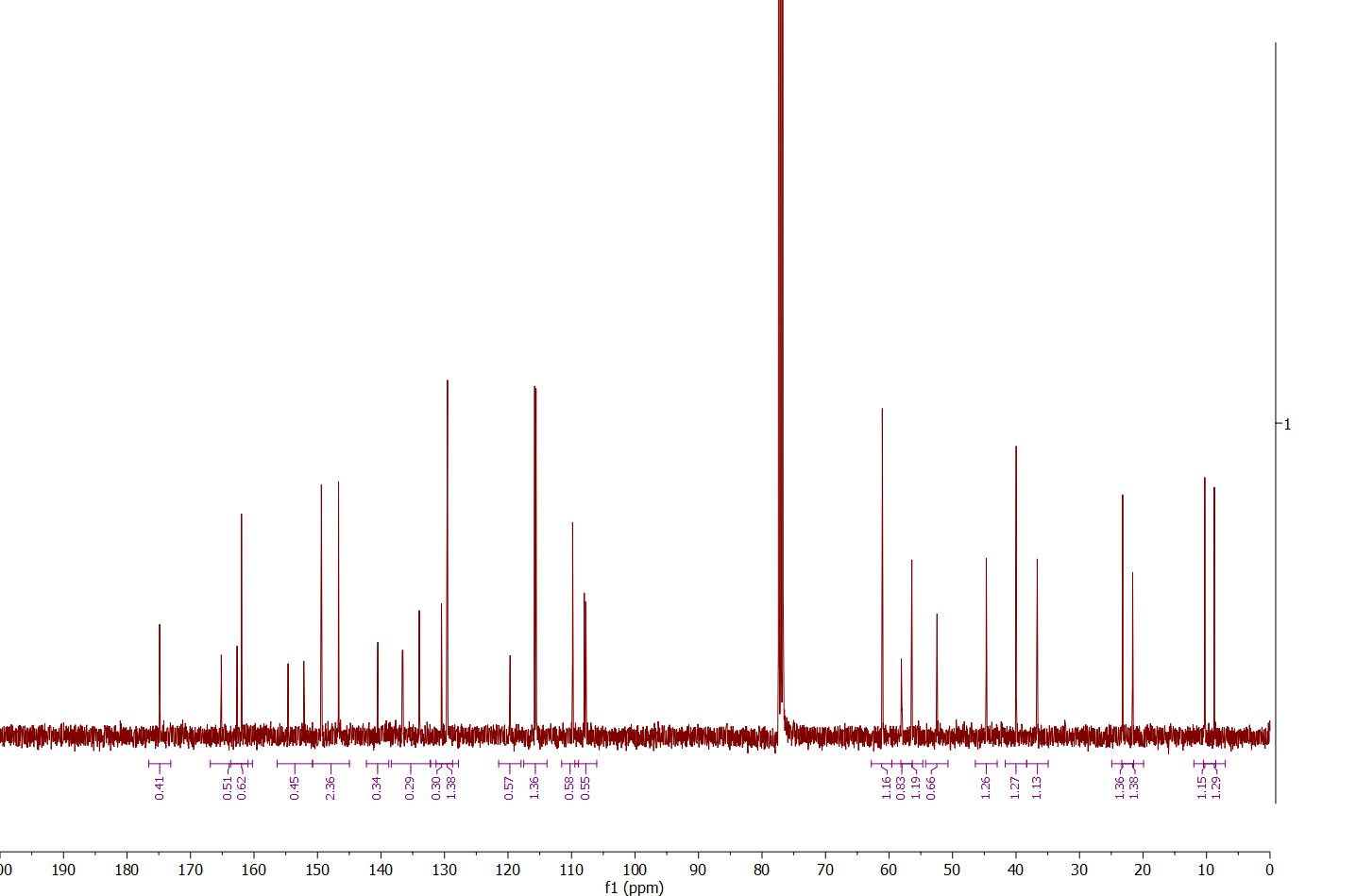


Figure 16S. ^13^C NMR spectrum of **IIIb**.

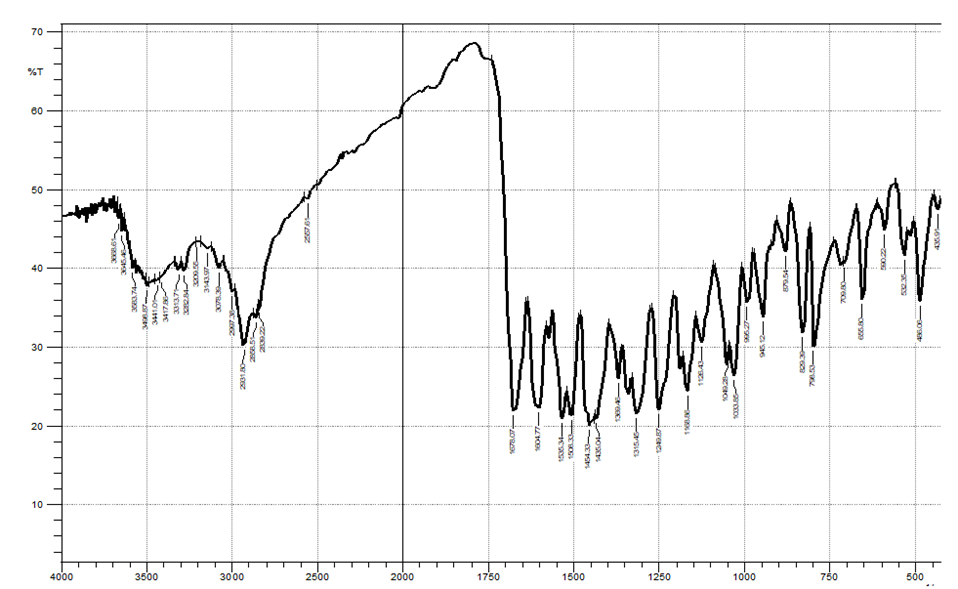


Figure 17S. IR spectrum of **IIIc**.


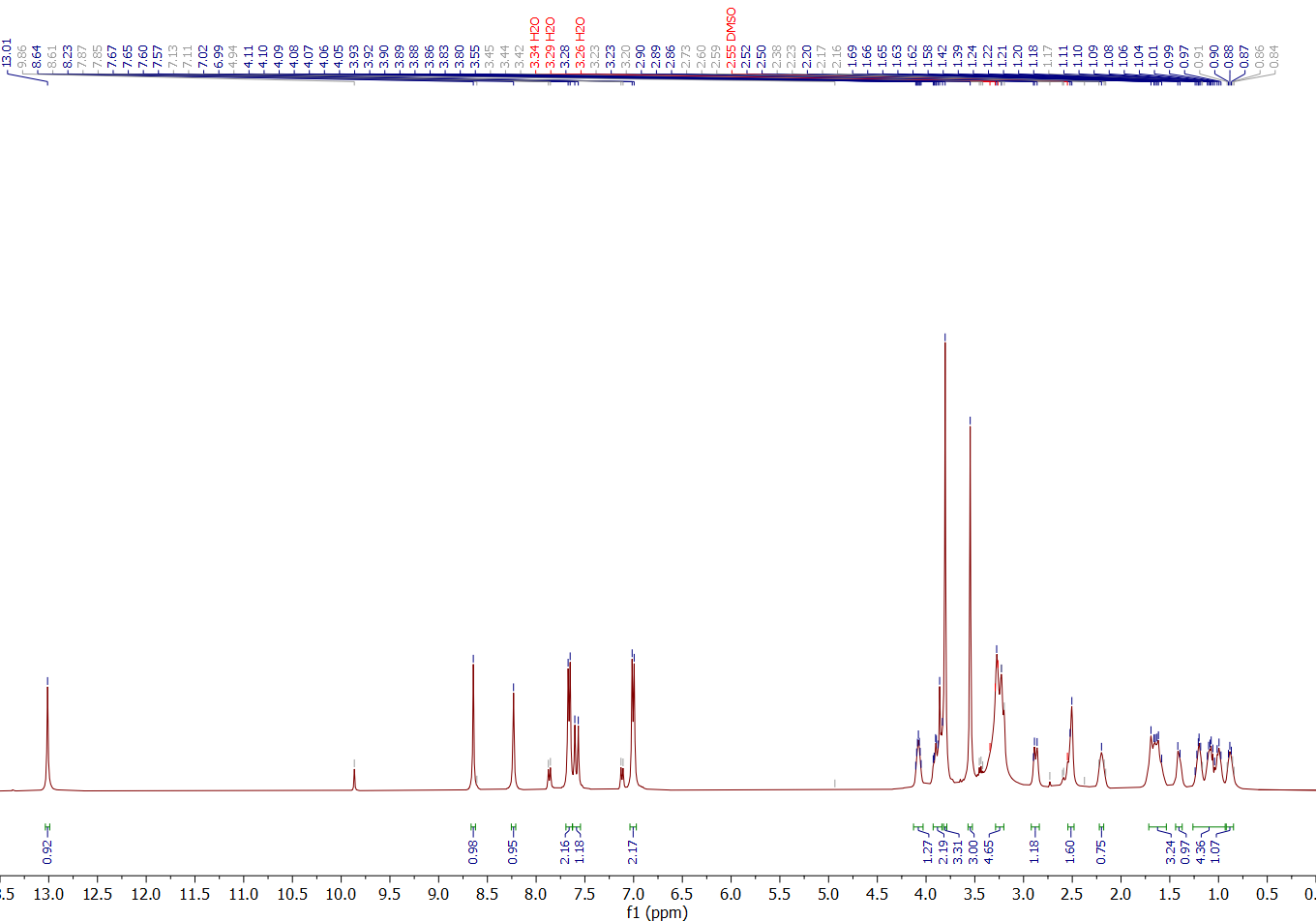


Figure 18S. ^1^H NMR spectrum of **IIIc**.


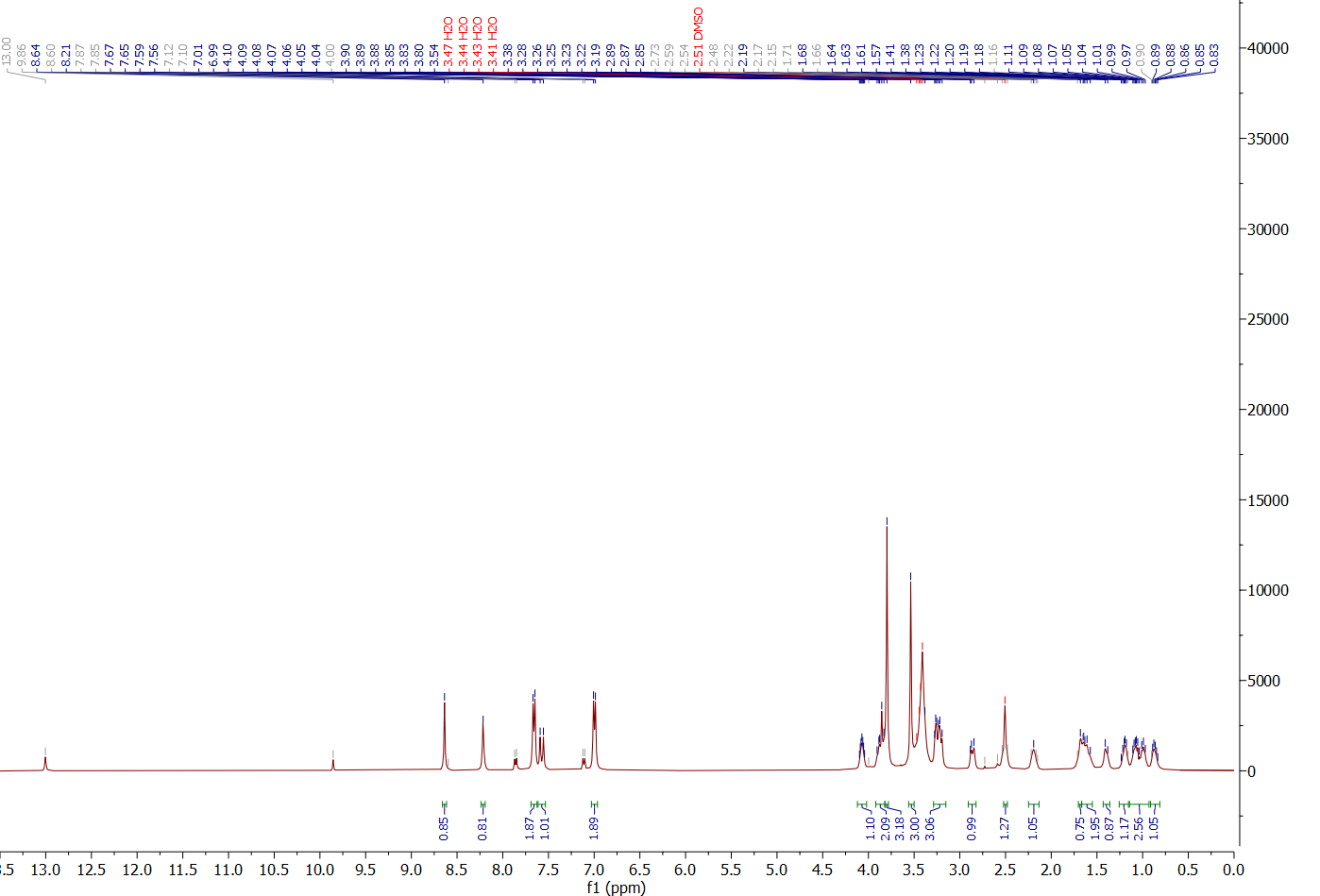


Figure 19S. ^1^H NMR (D_2_O) spectrum of **IIIc**.


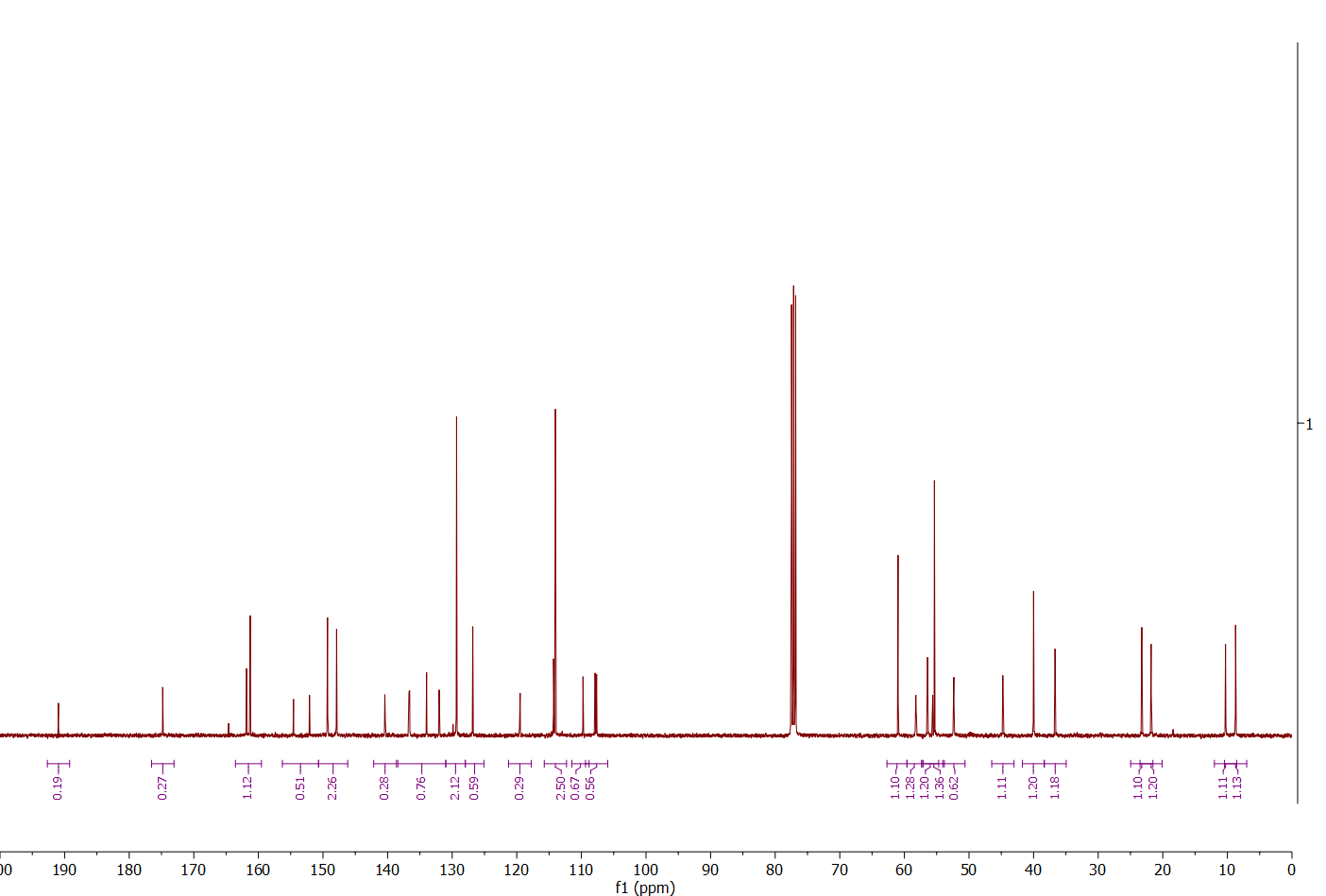


Figure 20S. ^13^C NMR spectrum of **IIIc**.


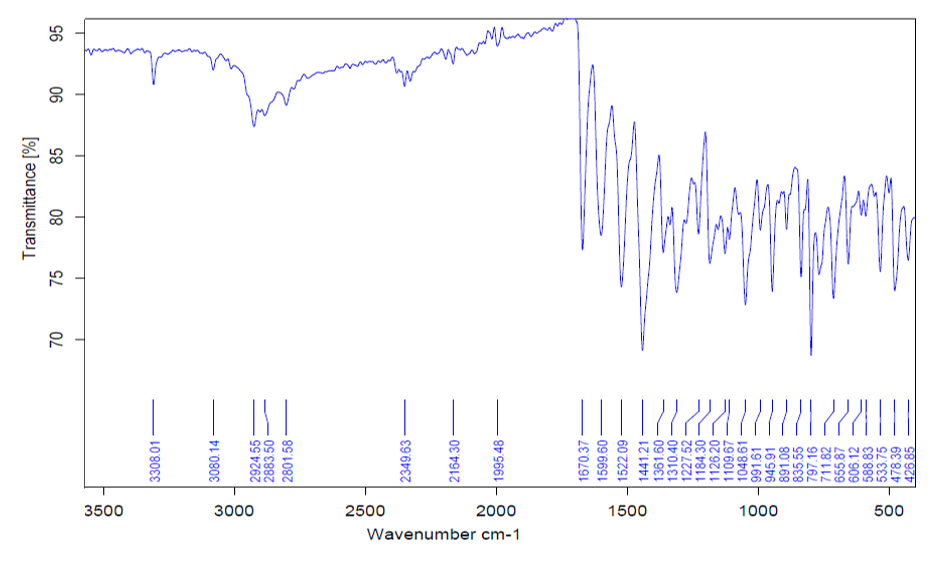


Figure 21S. IR spectrum of **IIId**.


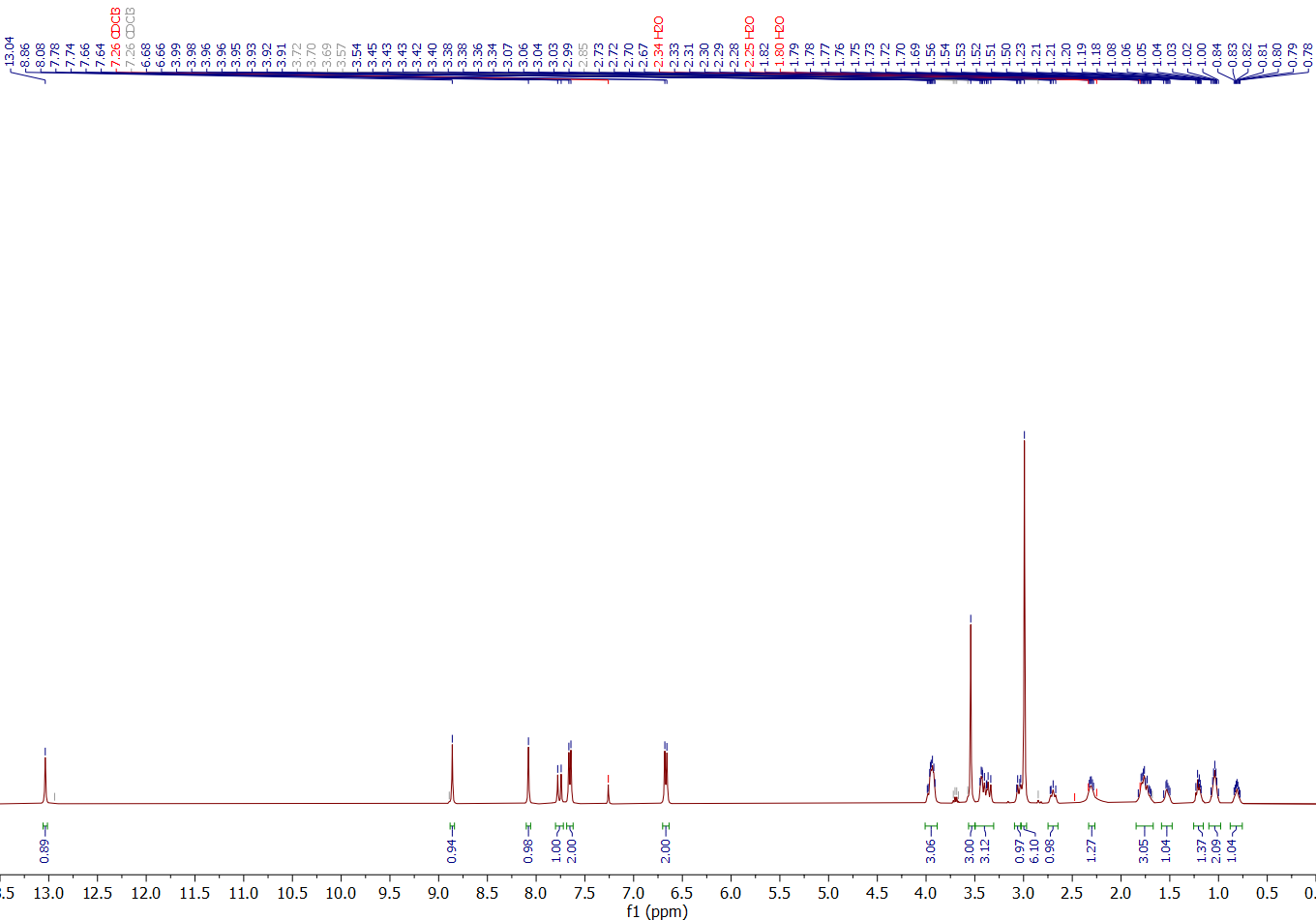


Figure 22S. ^1^H NMR spectrum of **IIId**.


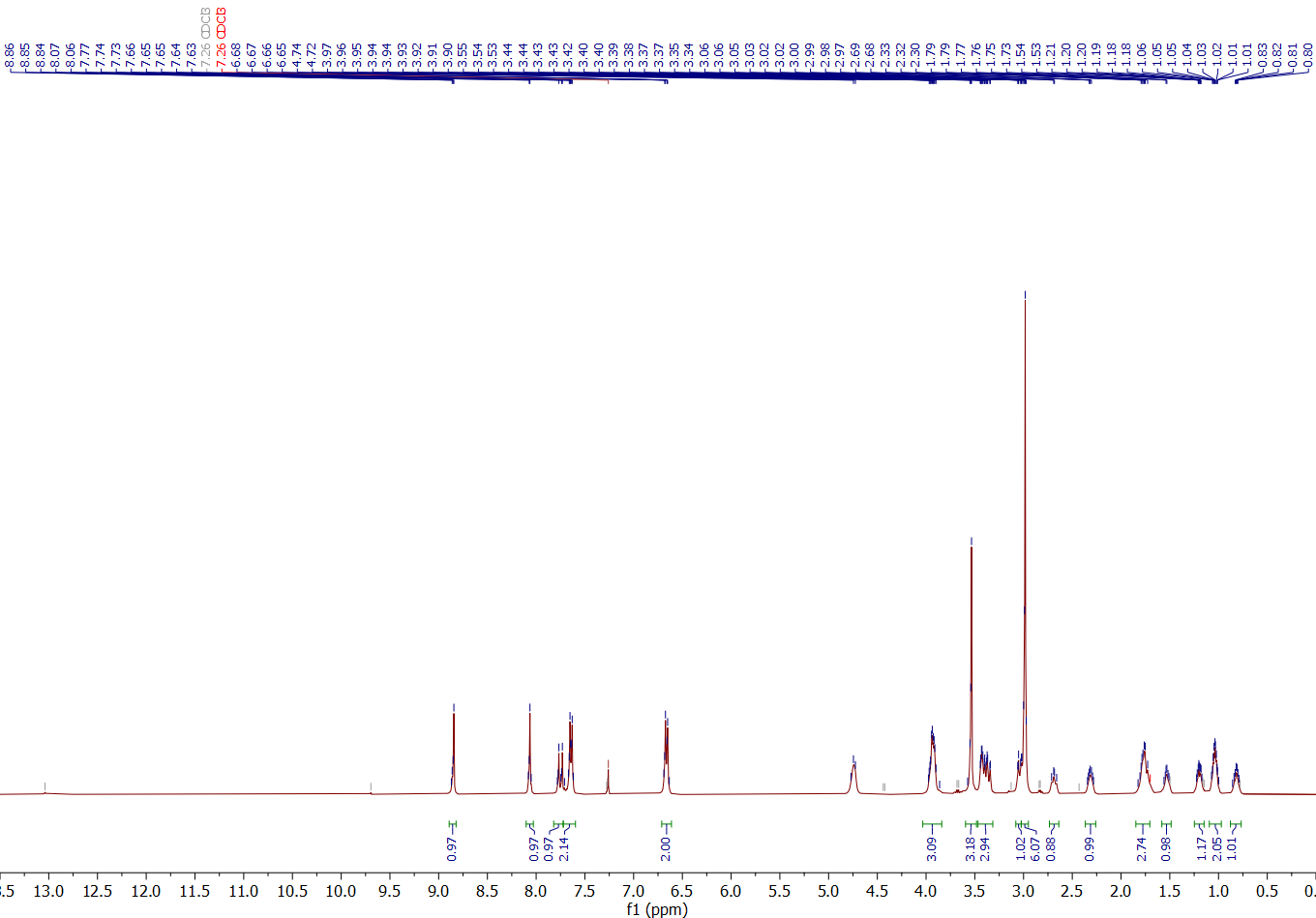


Figure 23S. ^1^H NMR (D_2_O) spectrum of **IIId**.


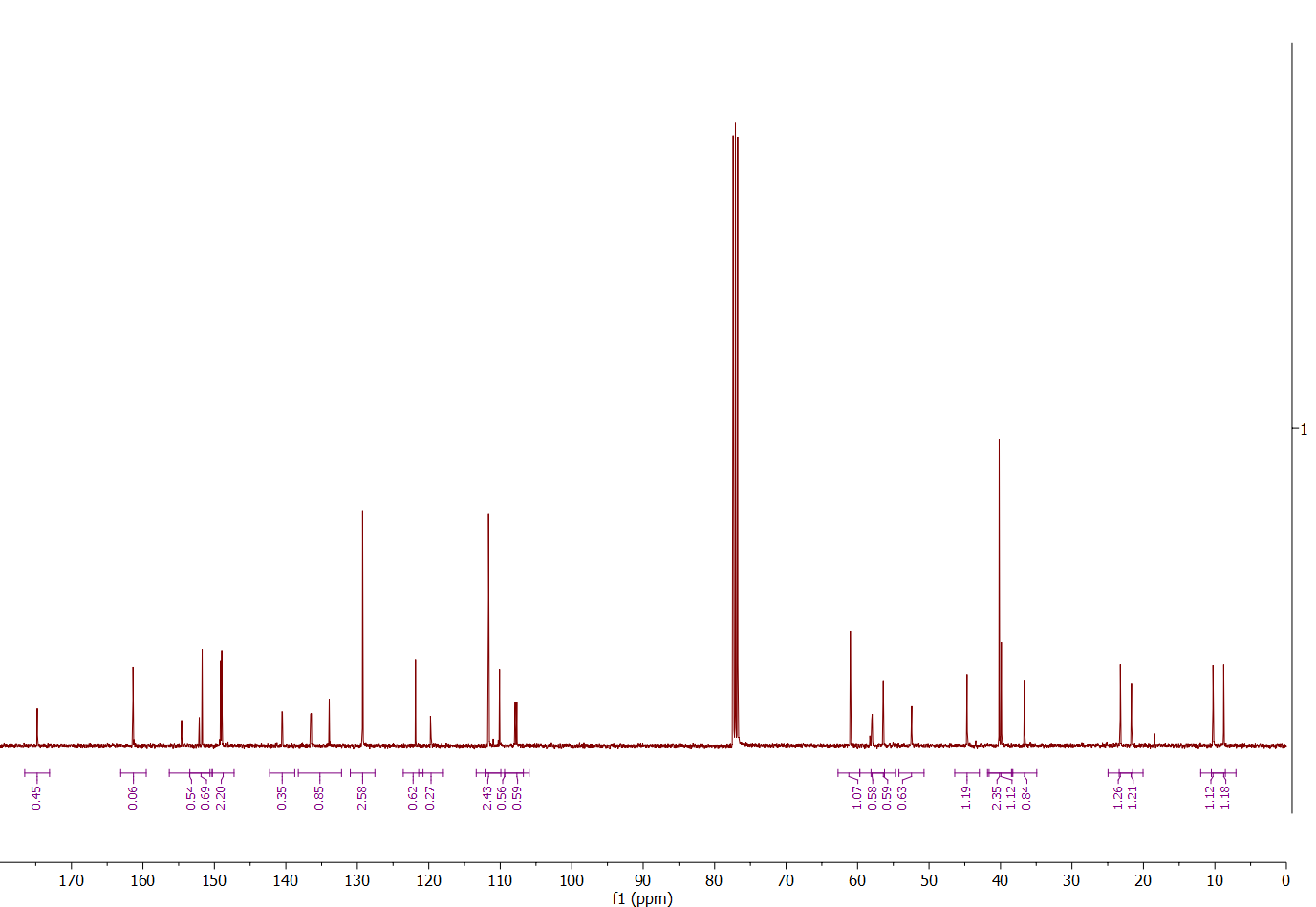


Figure 24S. . ^13^C NMR spectrum of **IIId**.

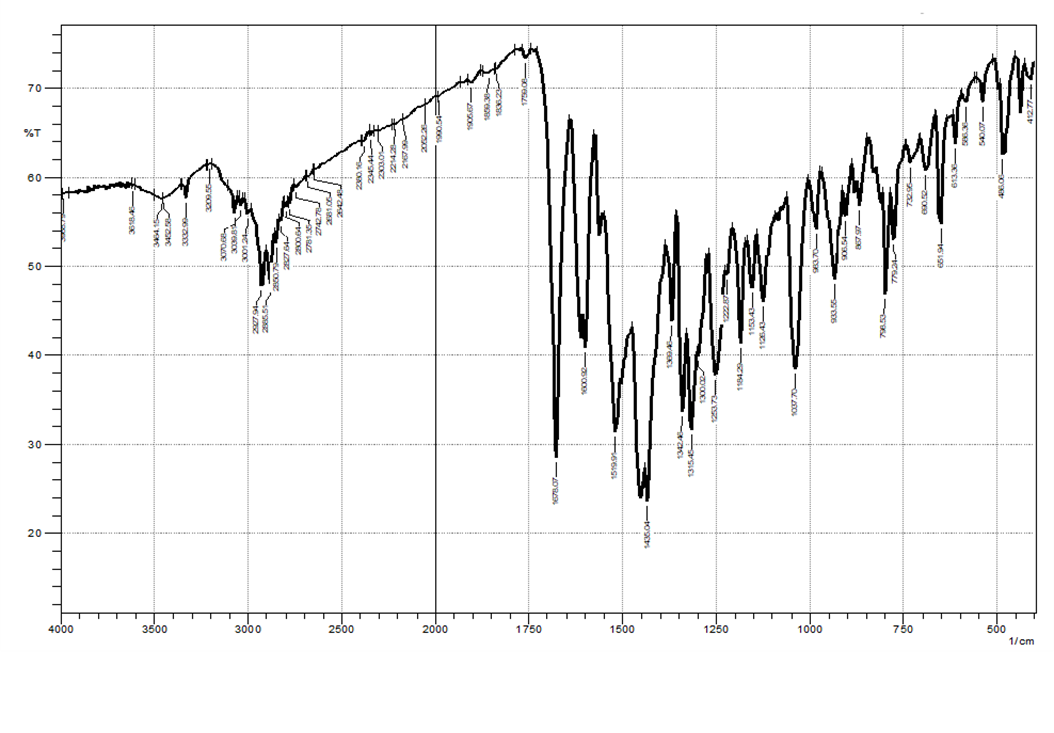


Figure 25S. IR spectrum of **IIIe**.


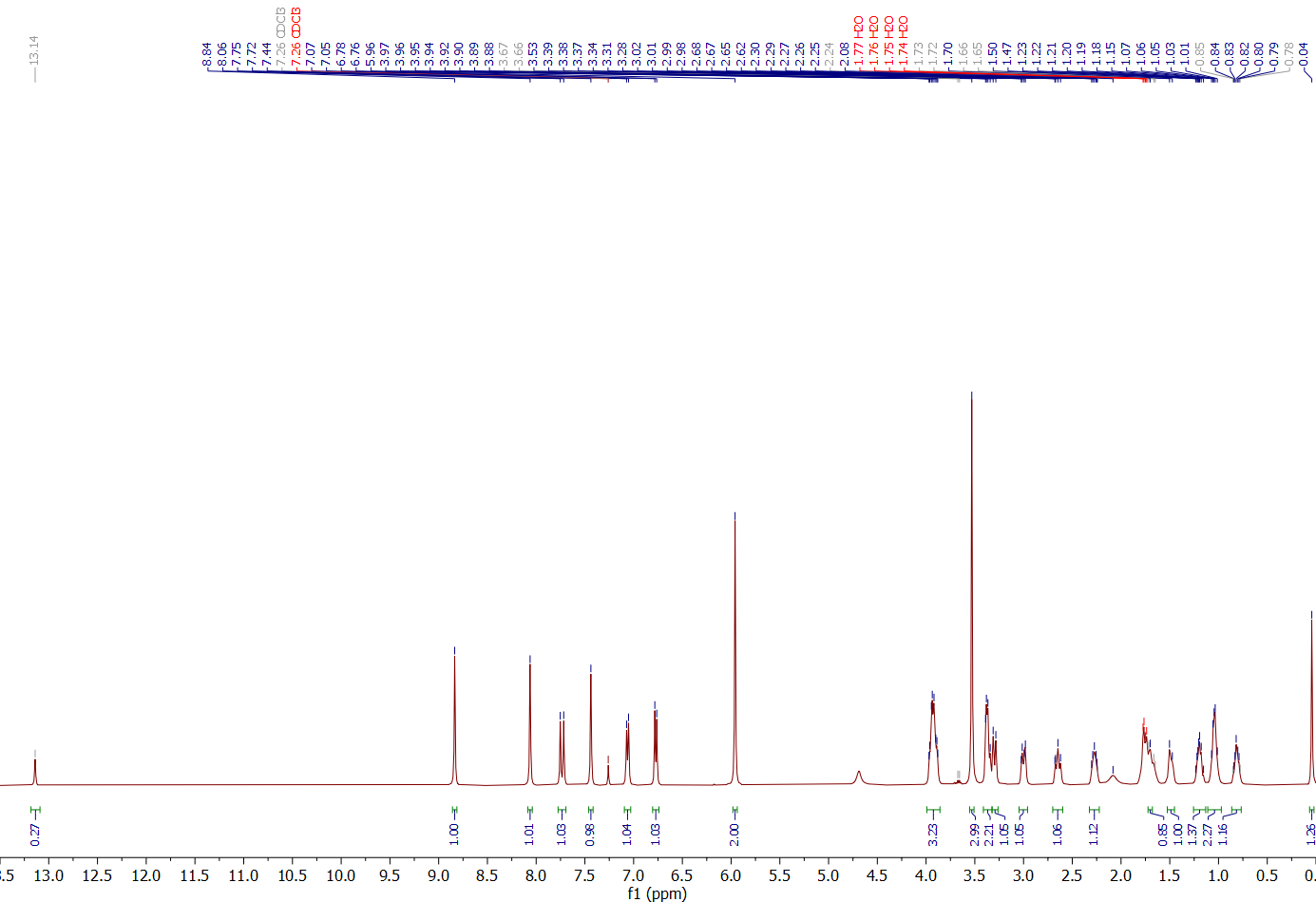


Figure 26S. ^1^H NMR spectrum of **IIIe**.


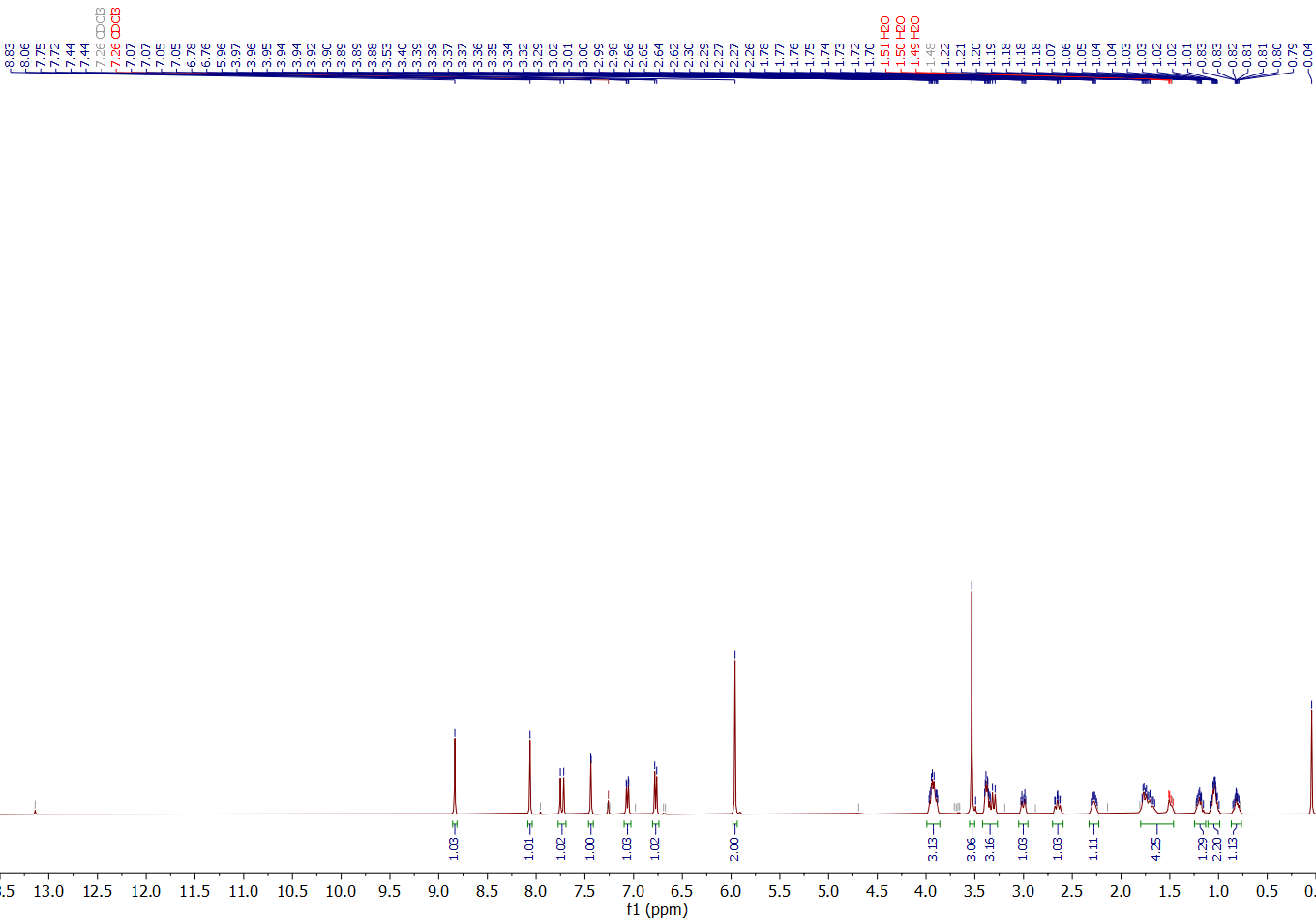


Figure 27S. ^1^H NMR (D_2_O) spectrum of **IIIe**.


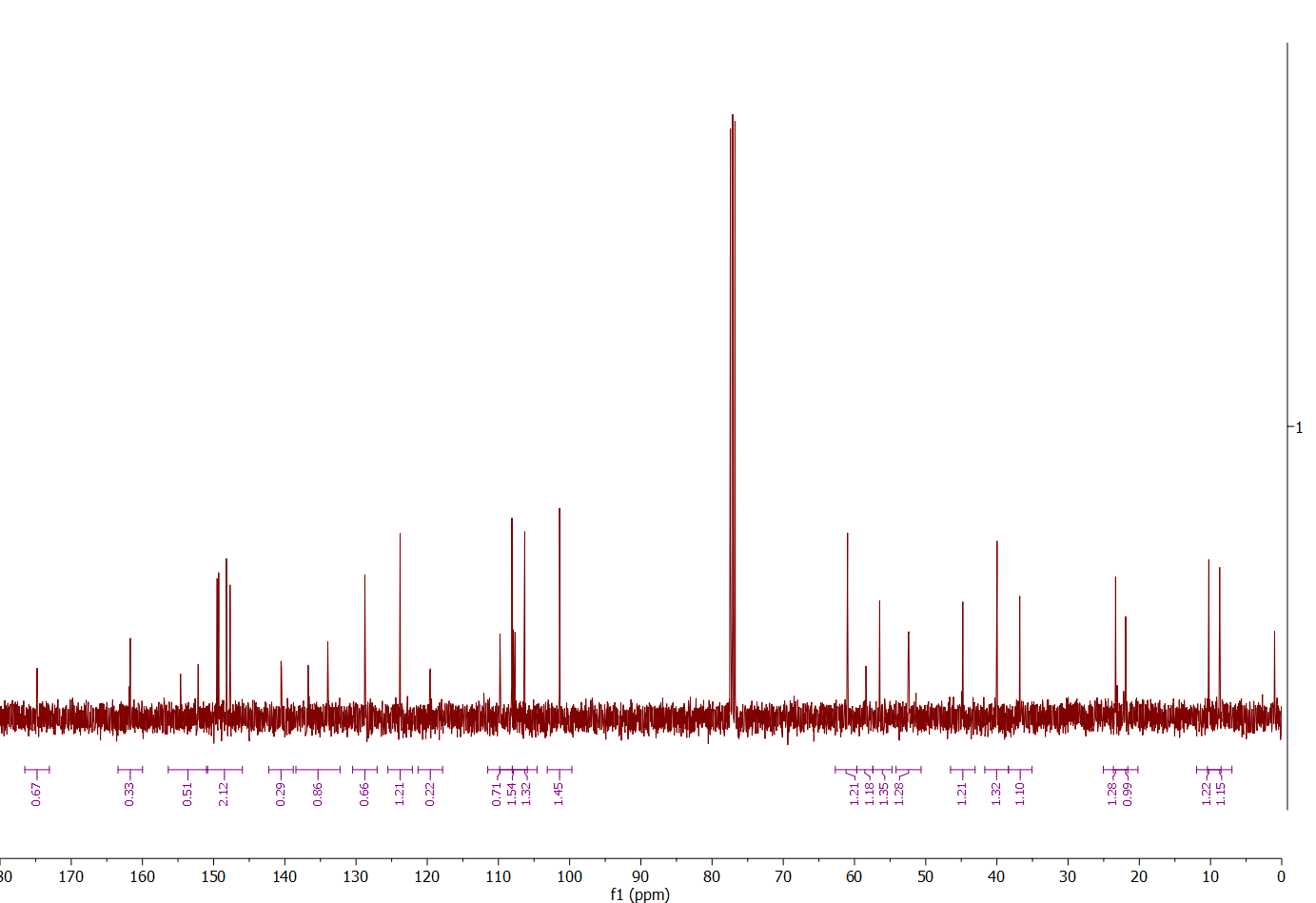


Figure 28S. ^13^C NMR spectrum of **IIIe**.

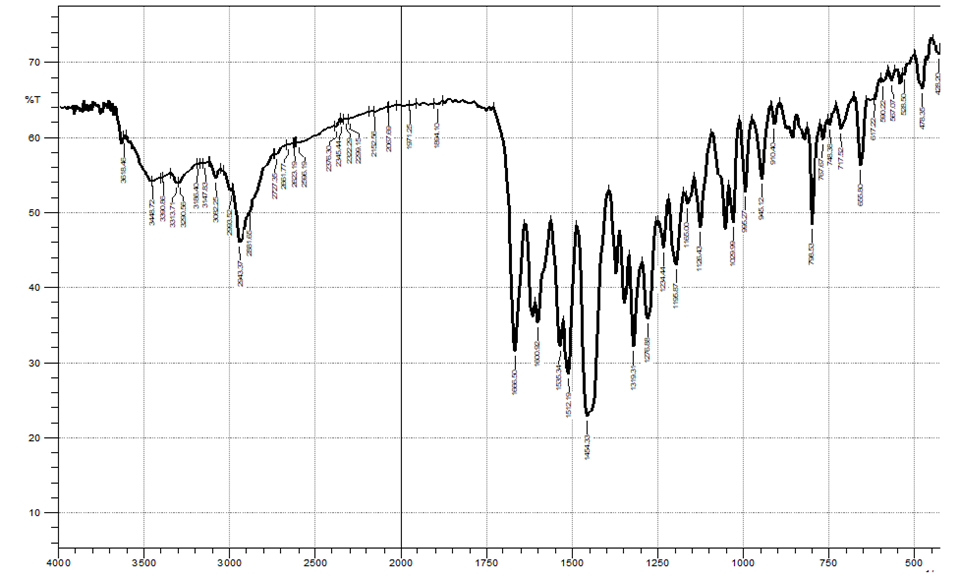


Figure 29S. IR spectrum of **IIIf**.


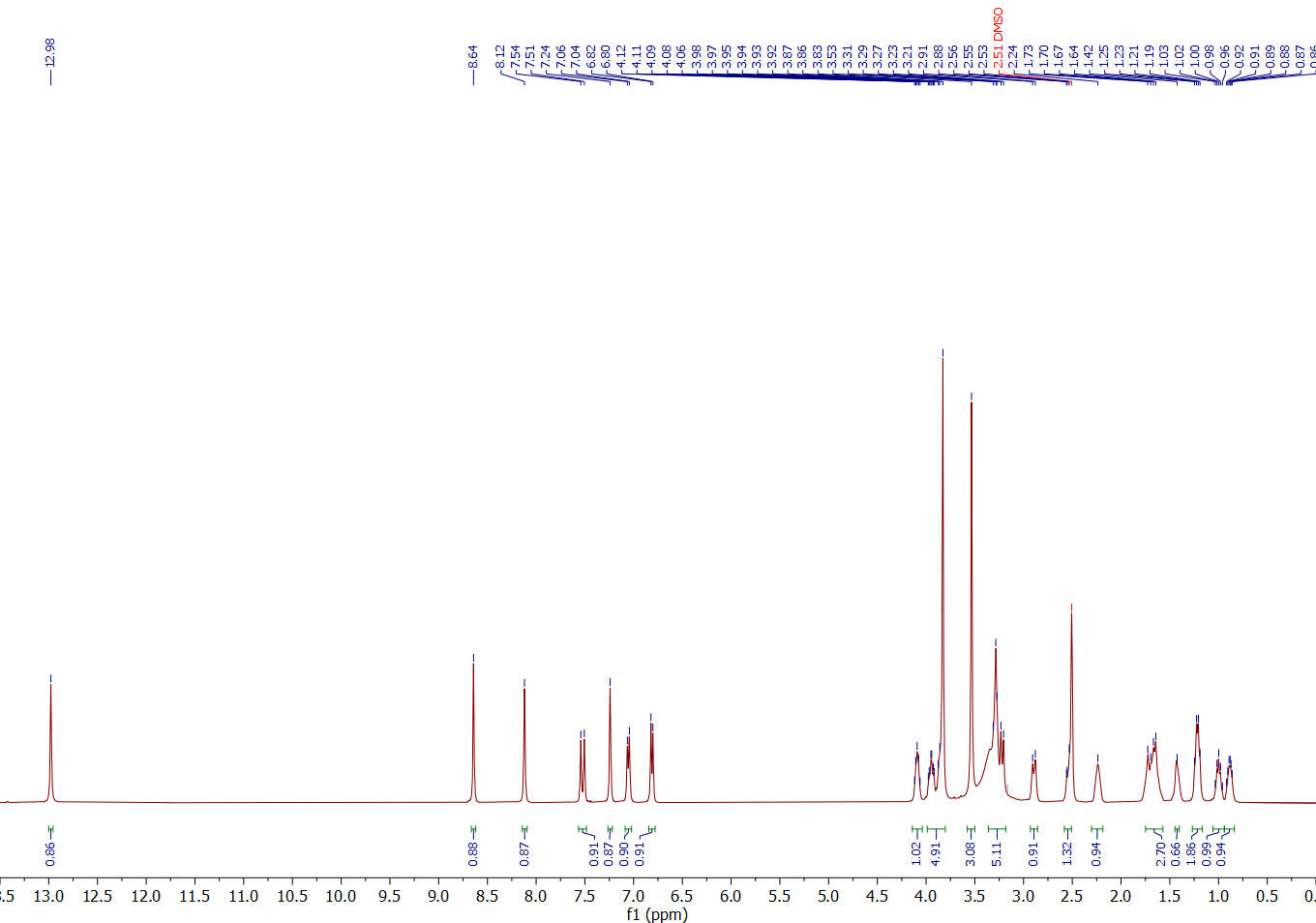


Figure 30S. ^1^H NMR spectrum of **IIIf**.


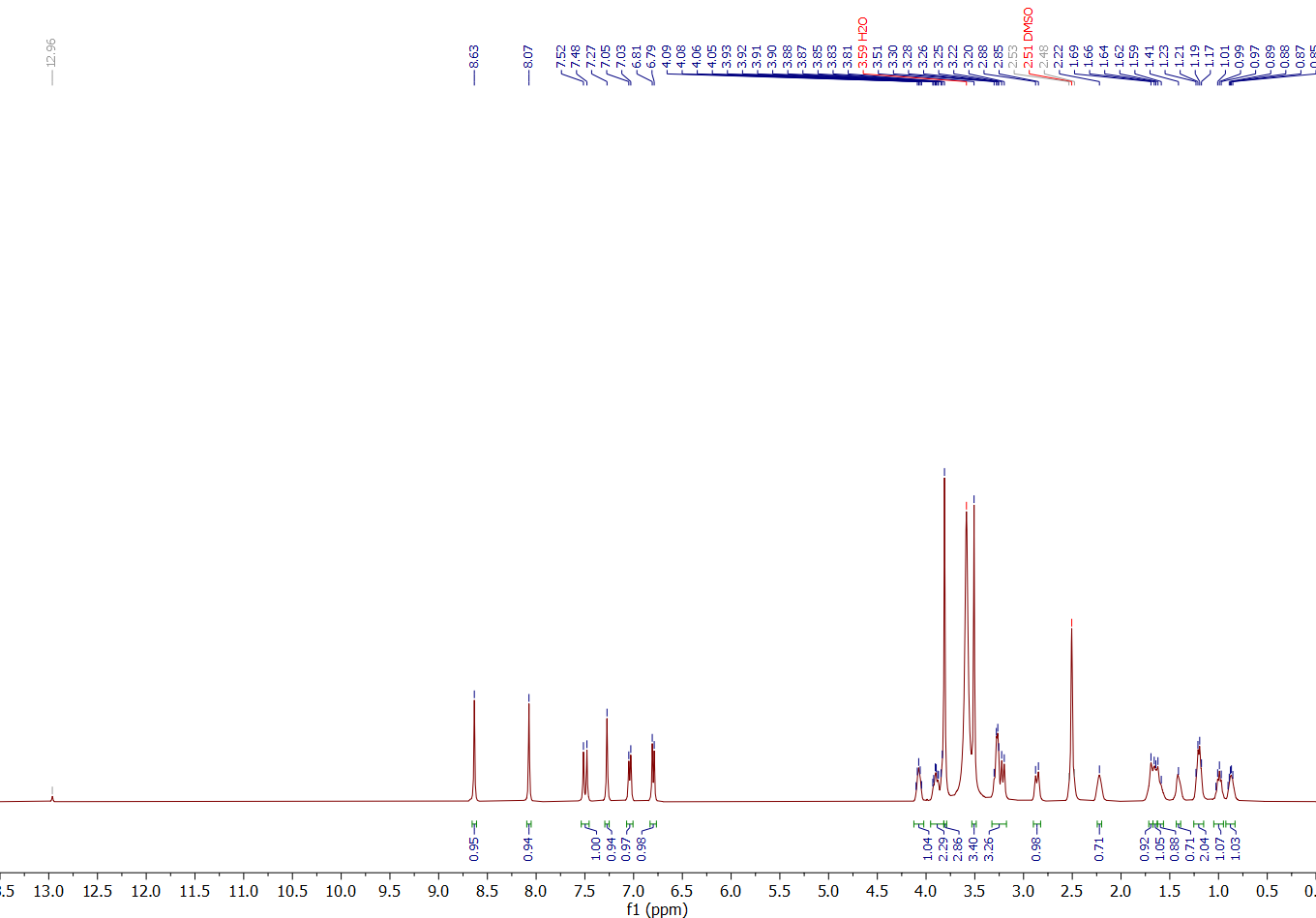


Figure 31S. ^1^H NMR (D_2_O) spectrum of **IIIf**.


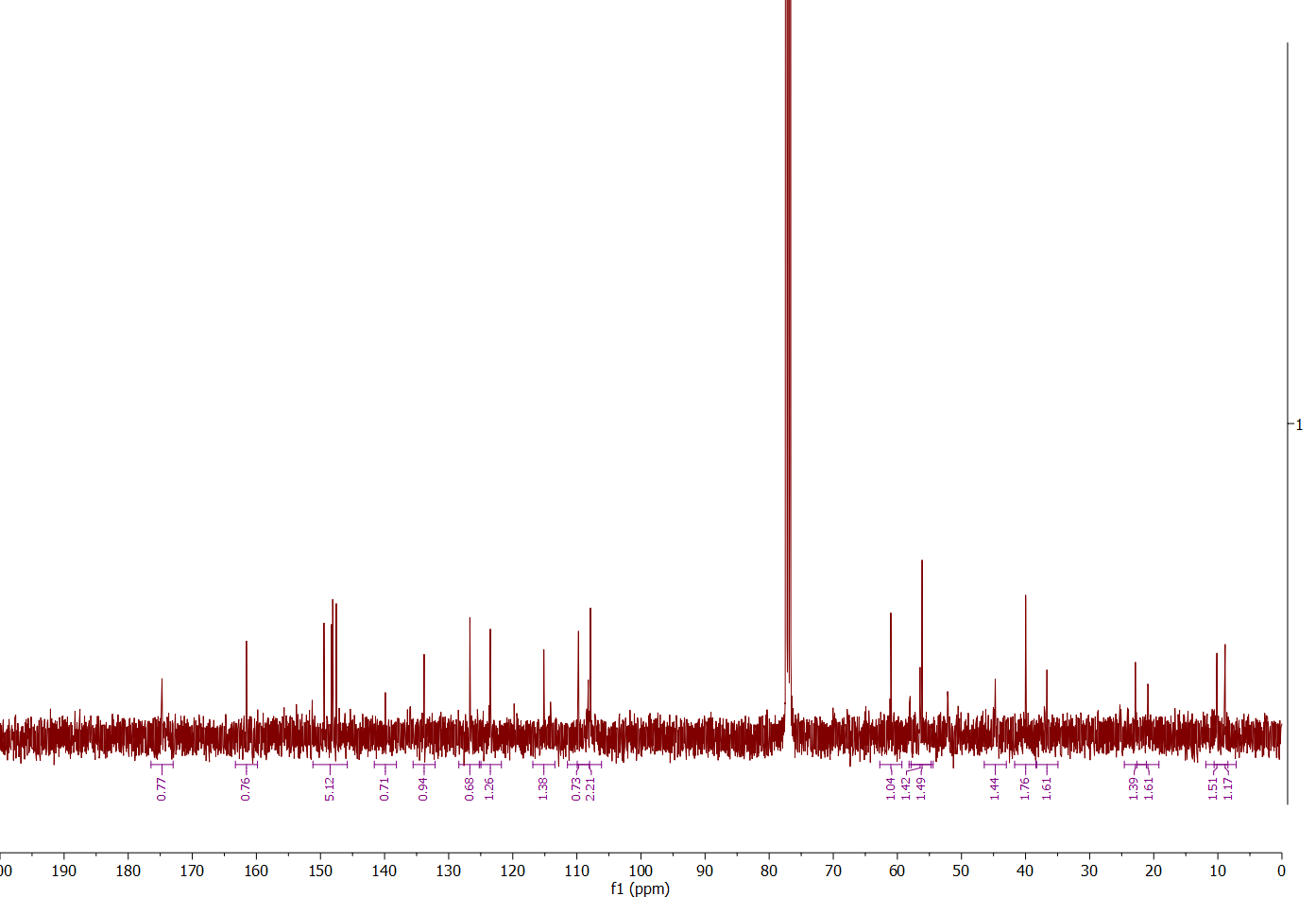


Figure 32S. ^13^C NMR spectrum of **IIIf**.

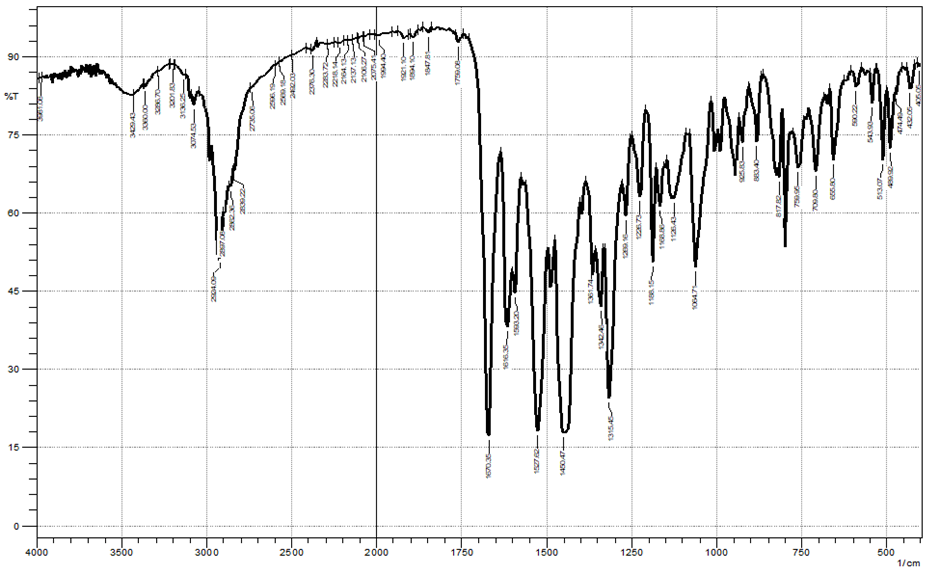


Figure 33S. IR spectrum of **IIIg**.


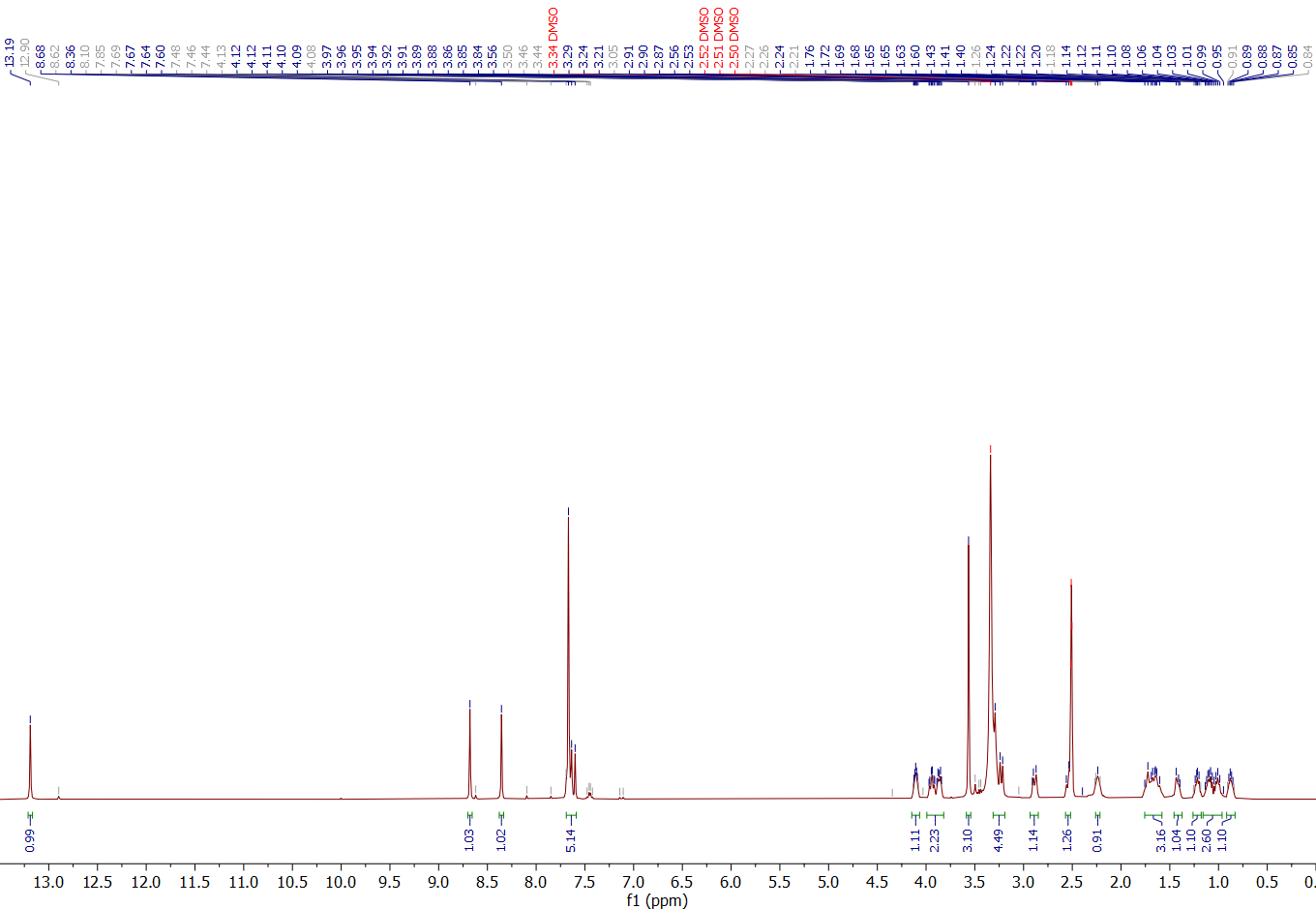


Figure 34S. ^1^H NMR spectrum of **IIIg**.


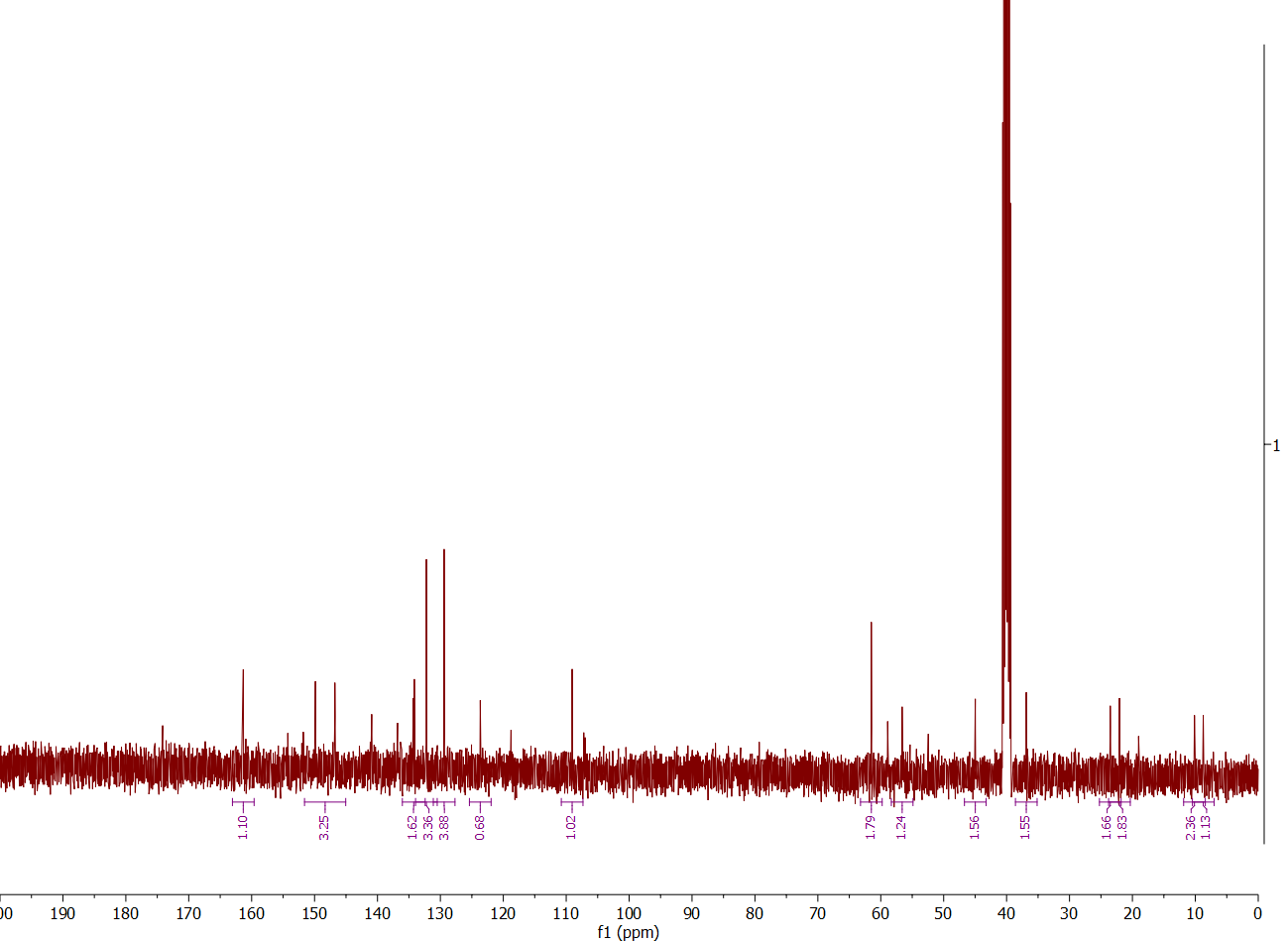


Figure 35S. ^13^C NMR spectrum of **IIIg**.


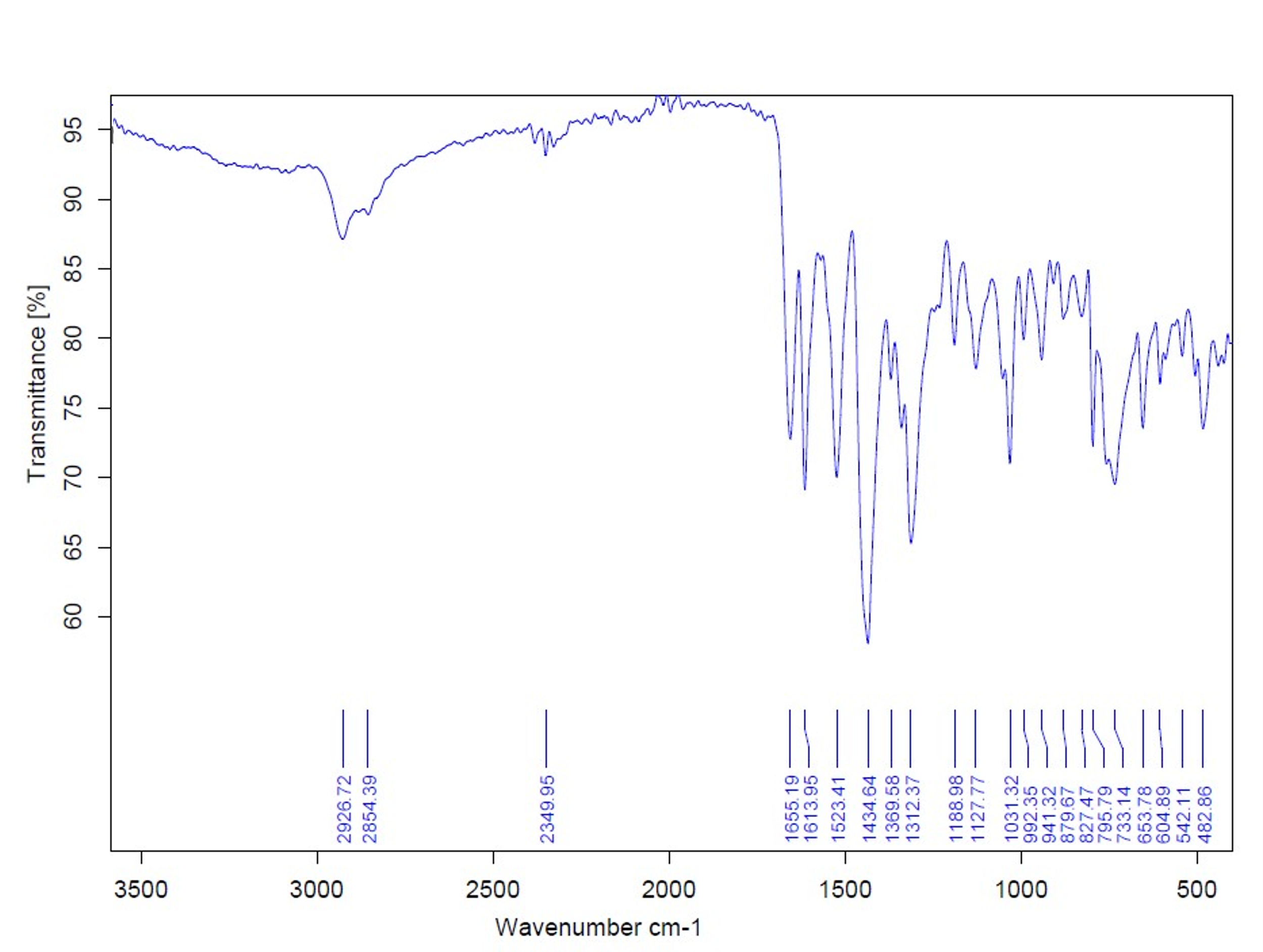


Figure 36S. IR spectrum of **IIIh**.


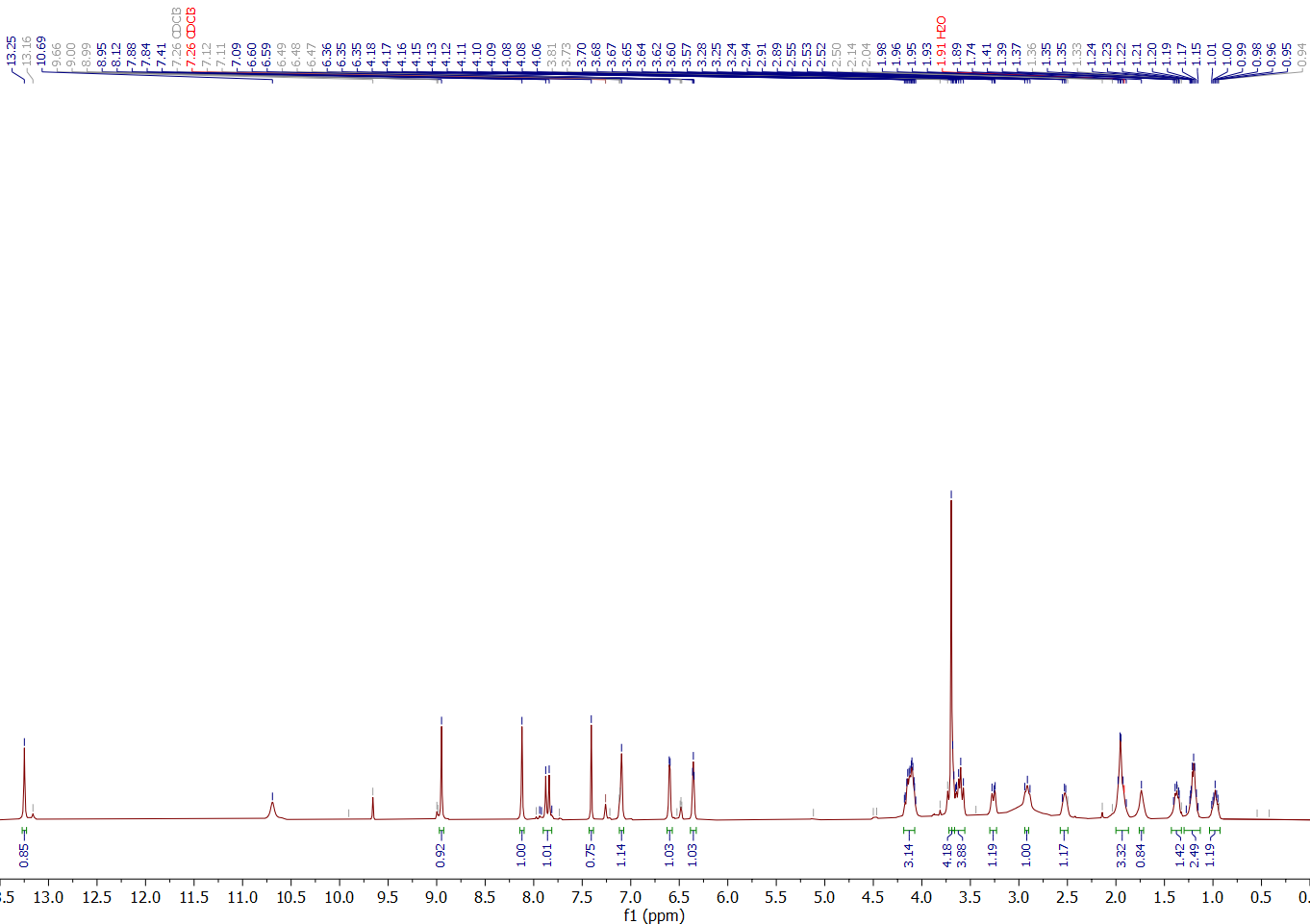


Figure 37S. ^1^H NMR spectrum of **IIIh**.


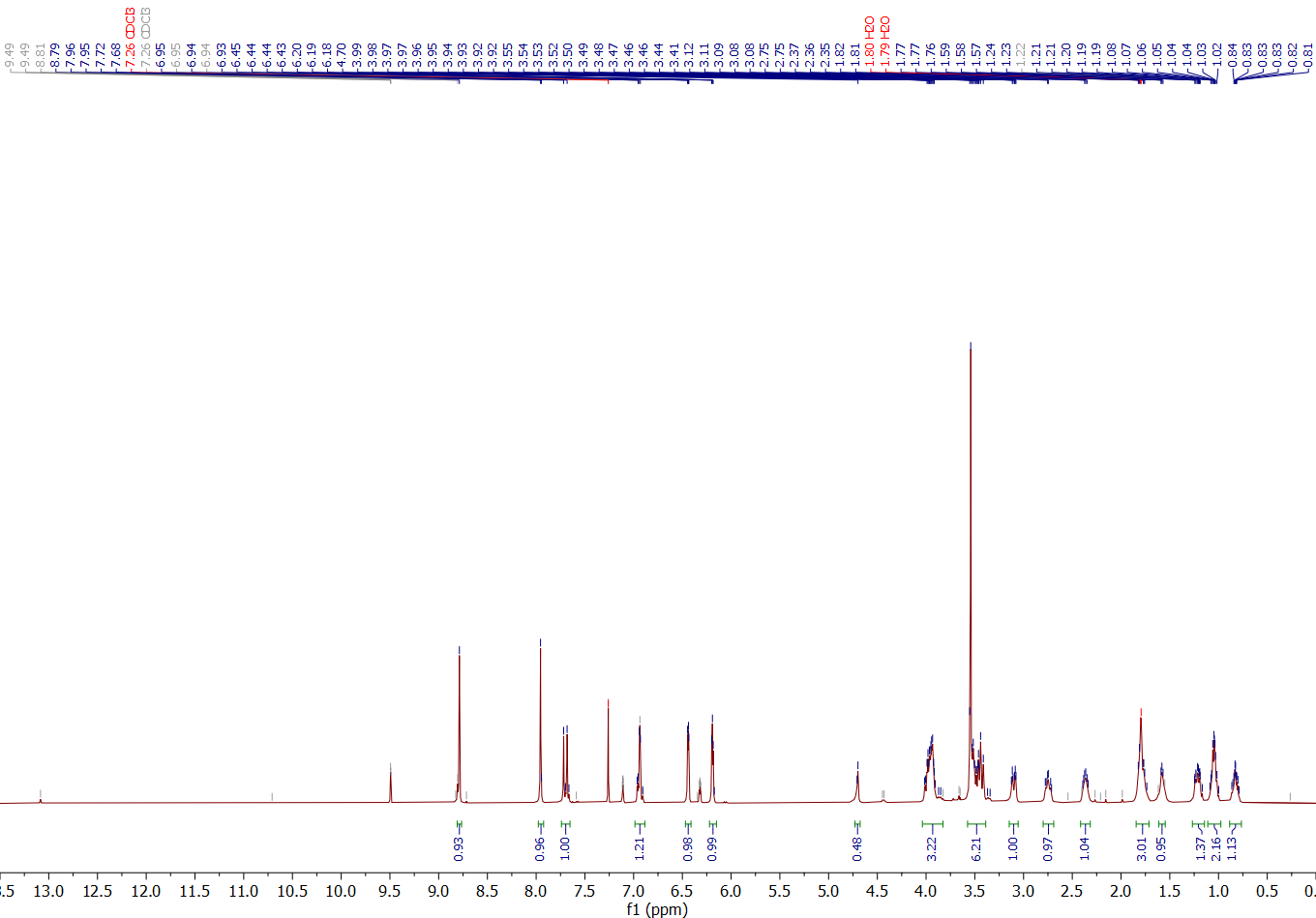


Figure 38S. ^1^H NMR (D_2_O) spectrum of **IIIh**.


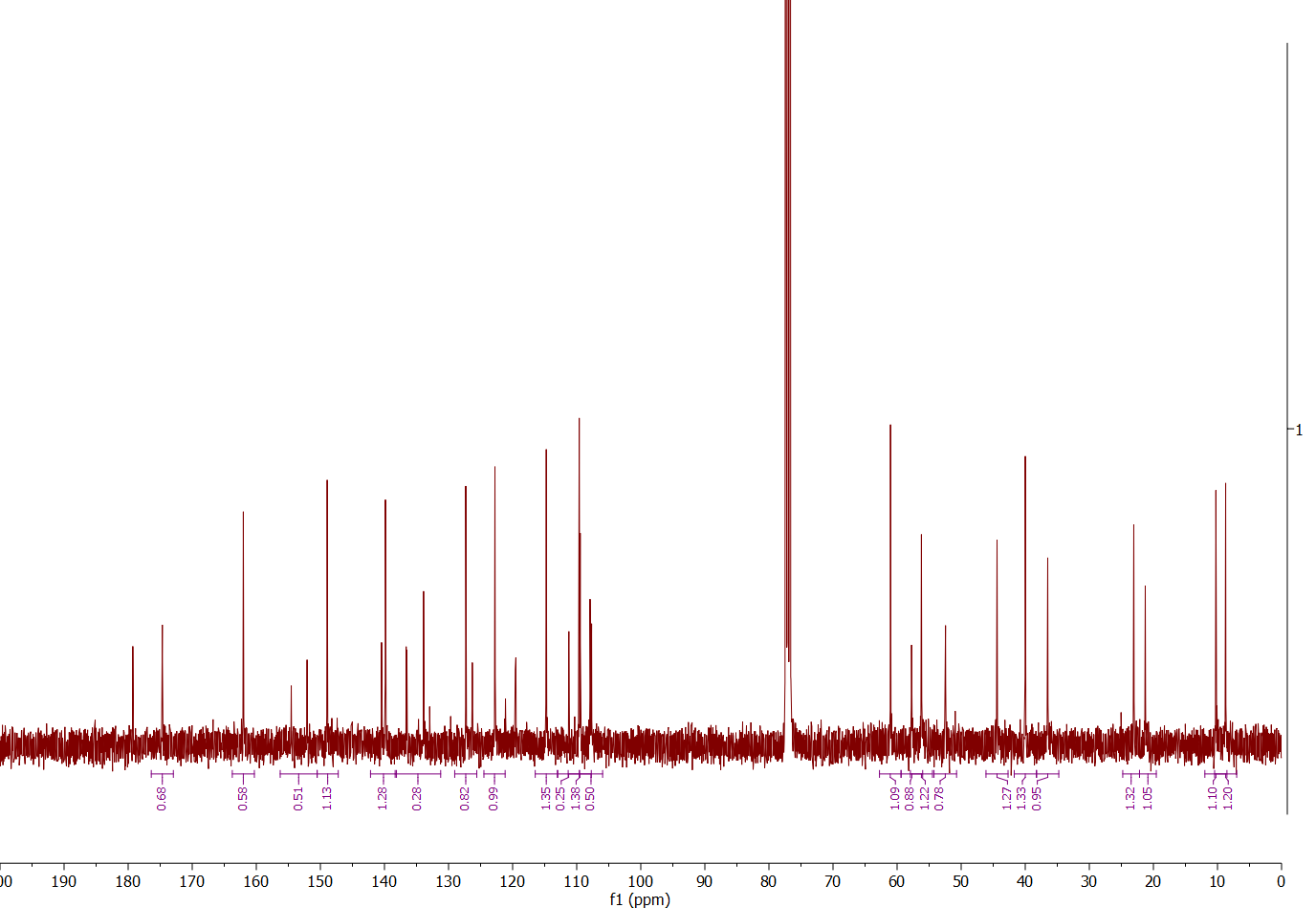


Figure 39S. ^13^C NMR spectrum of **IIIh**.

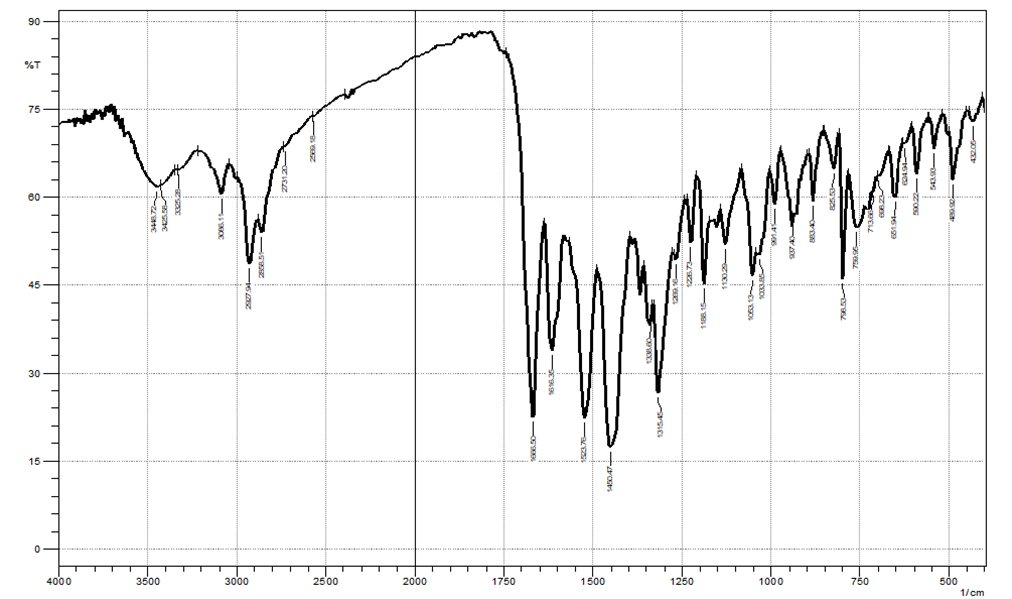


Figure 40S. IR spectrum of **IIIi**.


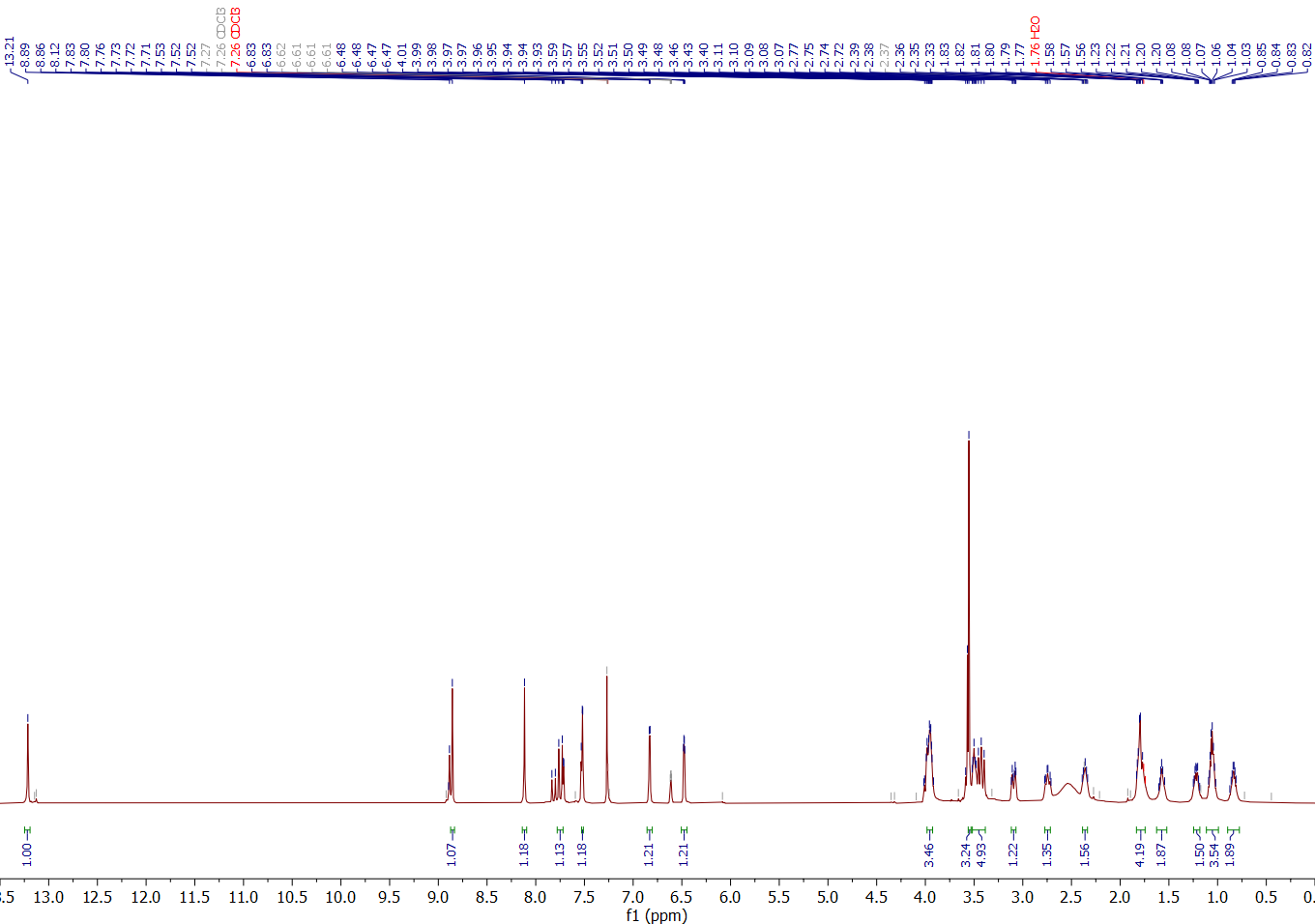


Figure 41S. ^1^H NMR spectrum of **IIIi**


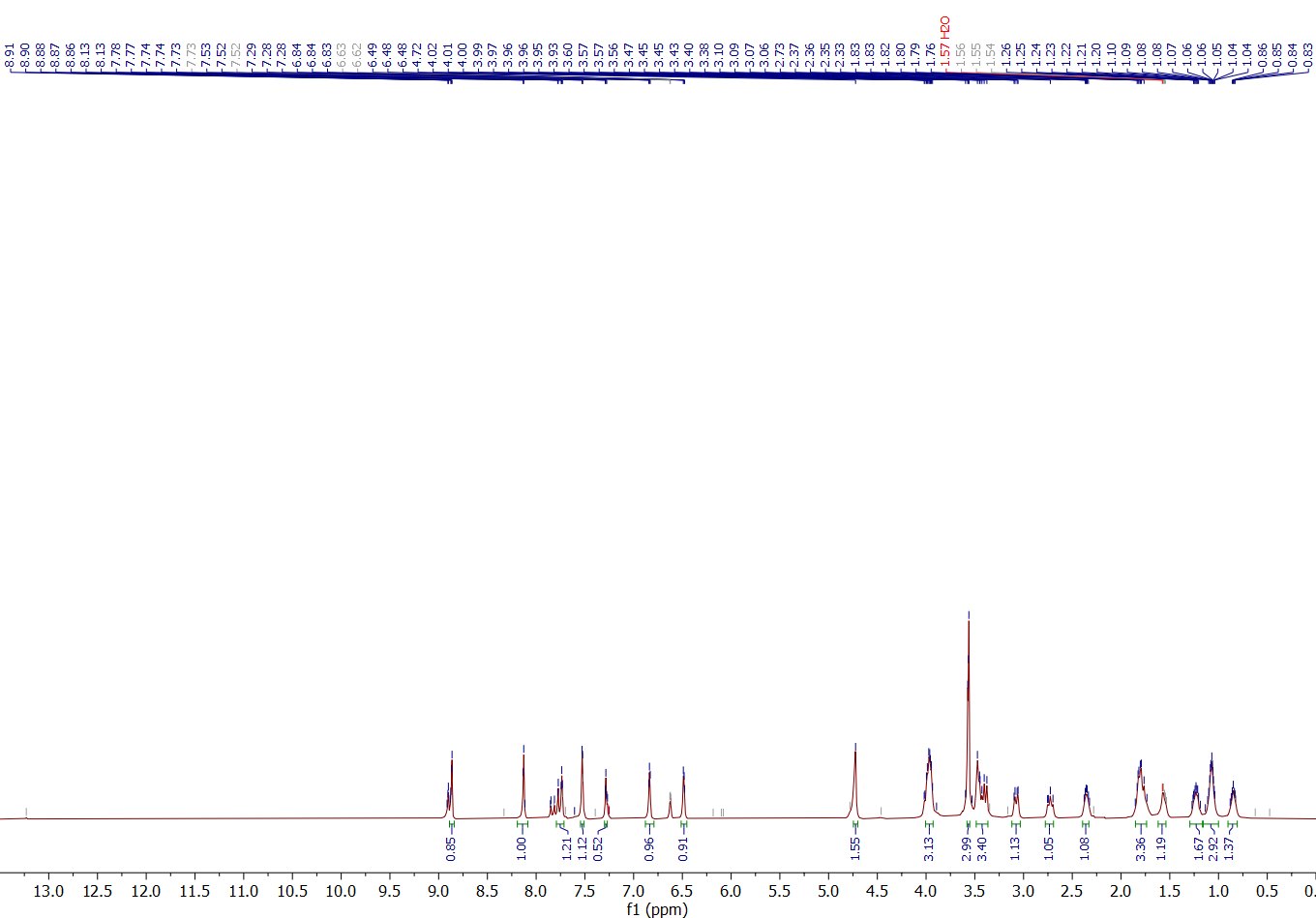


Figure 42S. ^1^H NMR (D_2_O) spectrum of **IIIi**


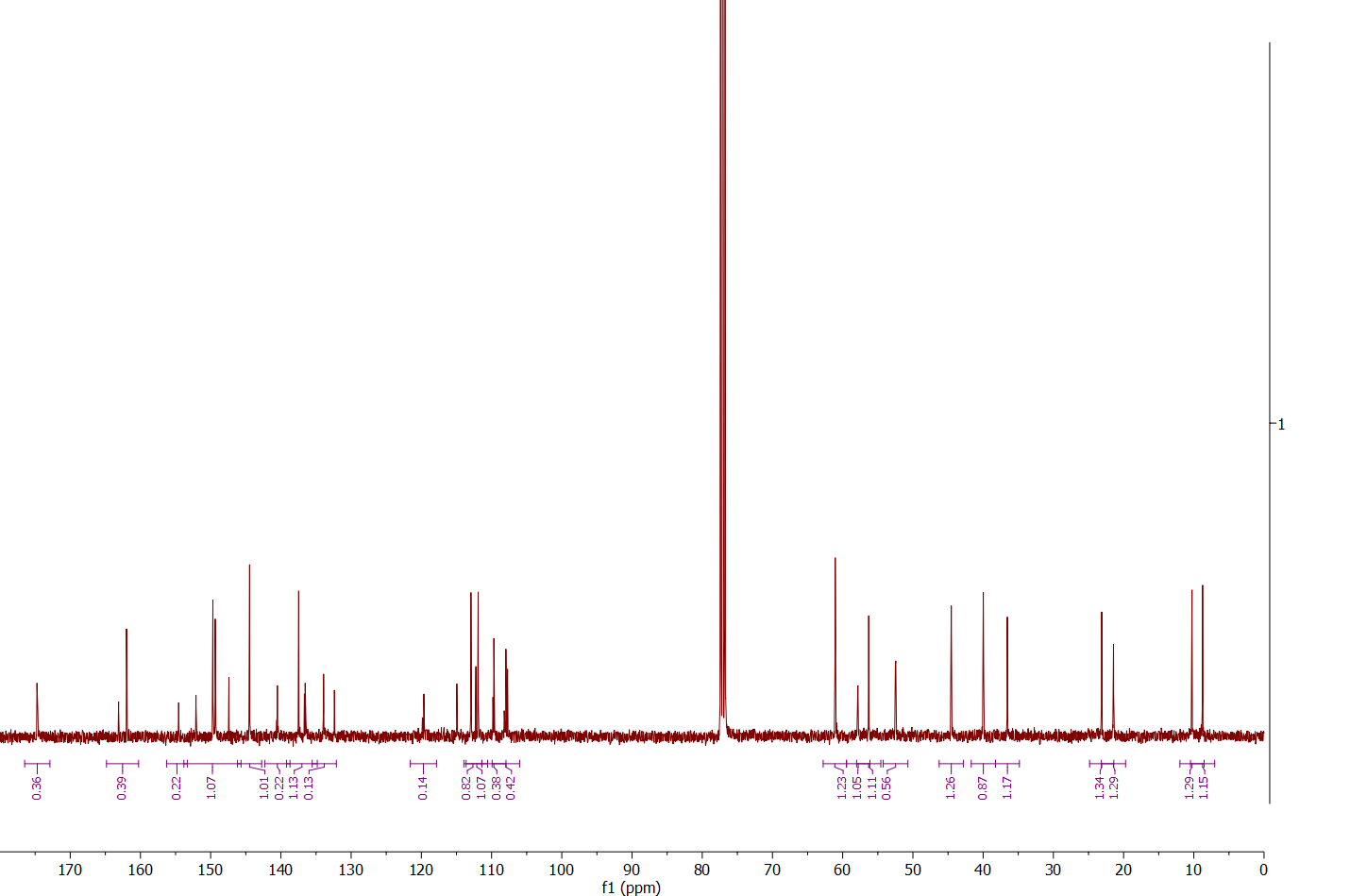


Figure 43S. ^13^C NMR spectrum of **IIIi**.

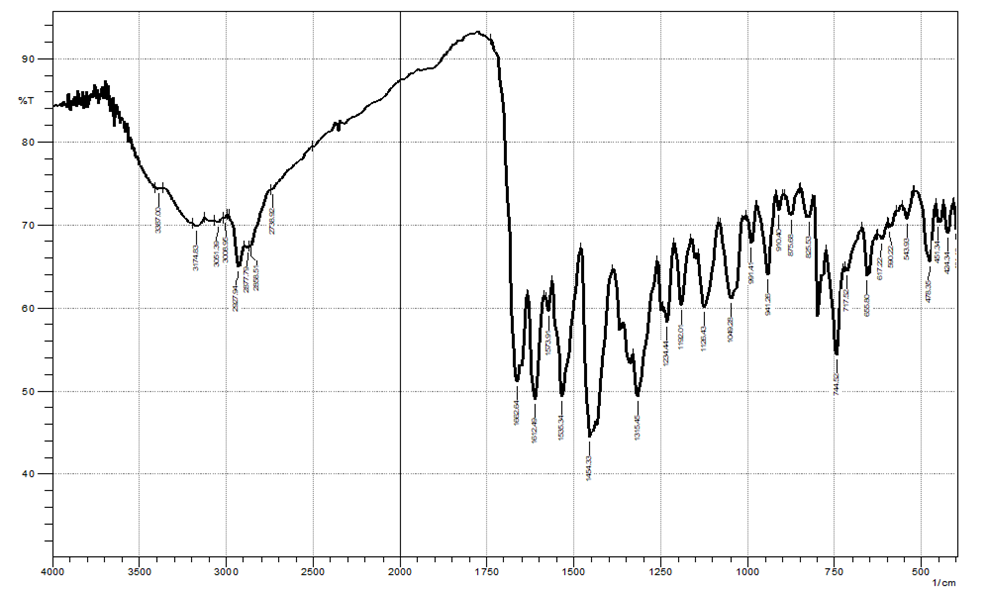


Figure 44S. IR spectrum of **IIIj**.


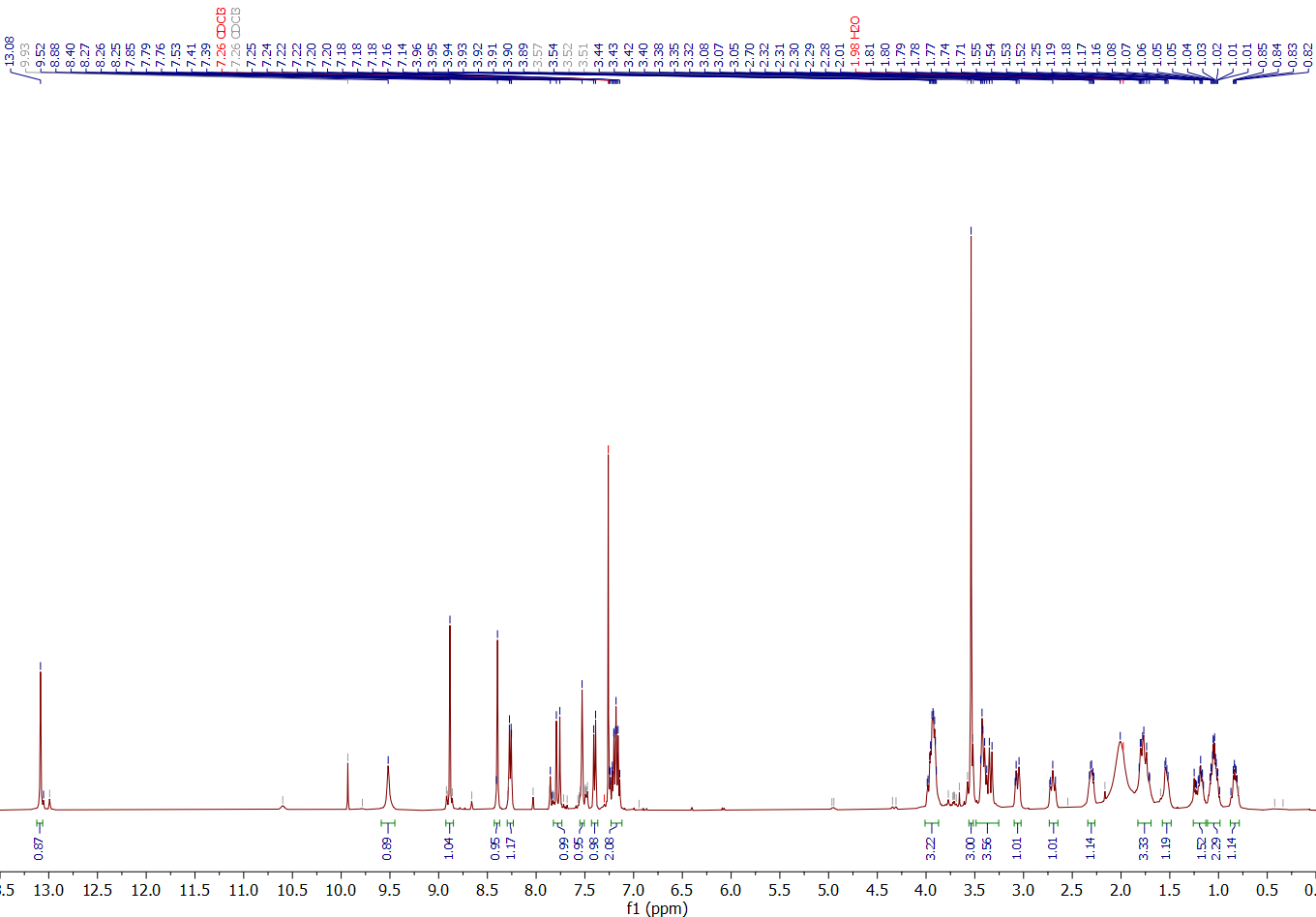


Figure 45S. ^1^H NMR spectrum of **IIIj**.


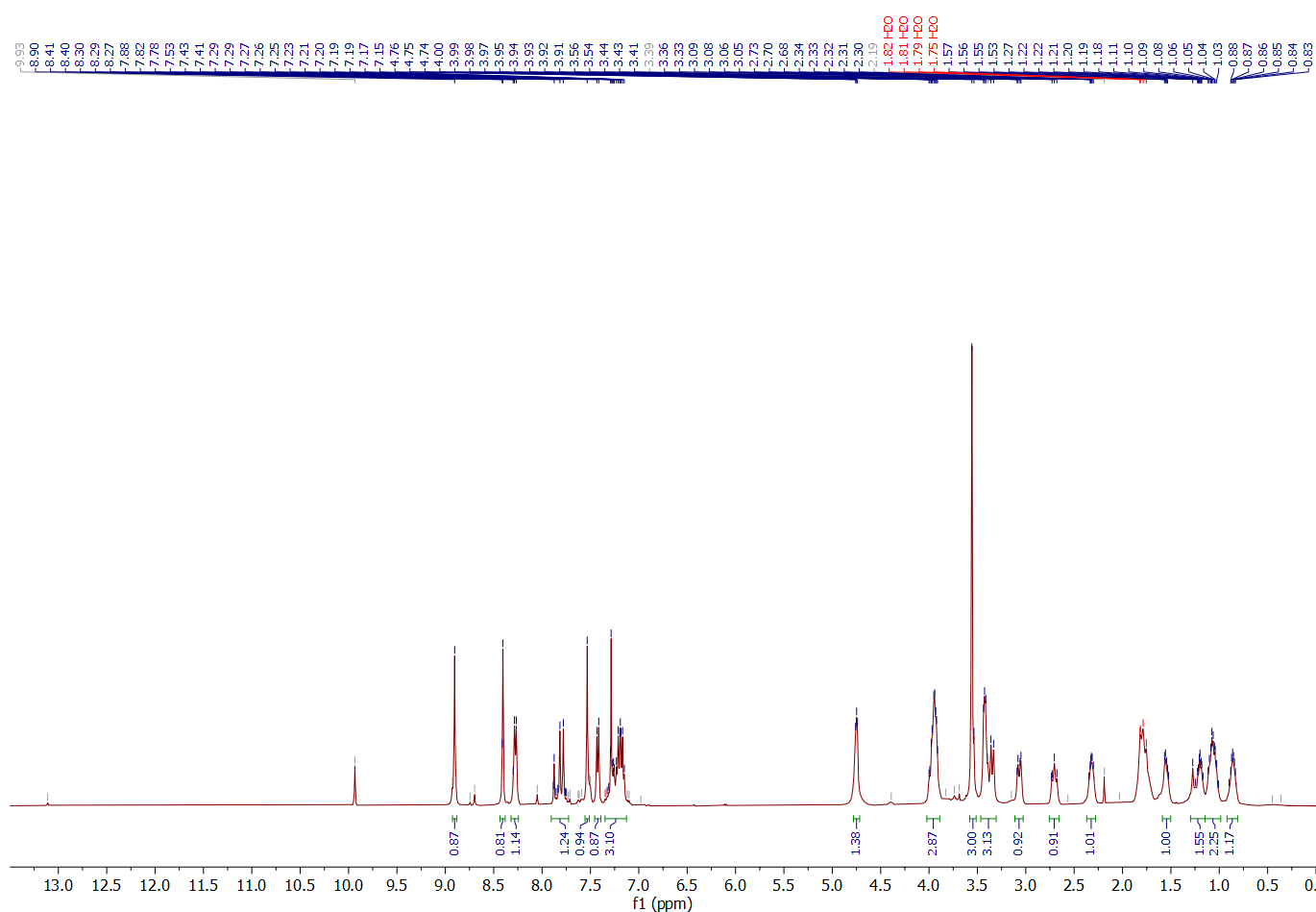


Figure 46S. ^1^H NMR (D_2_O) spectrum of **IIIj**.


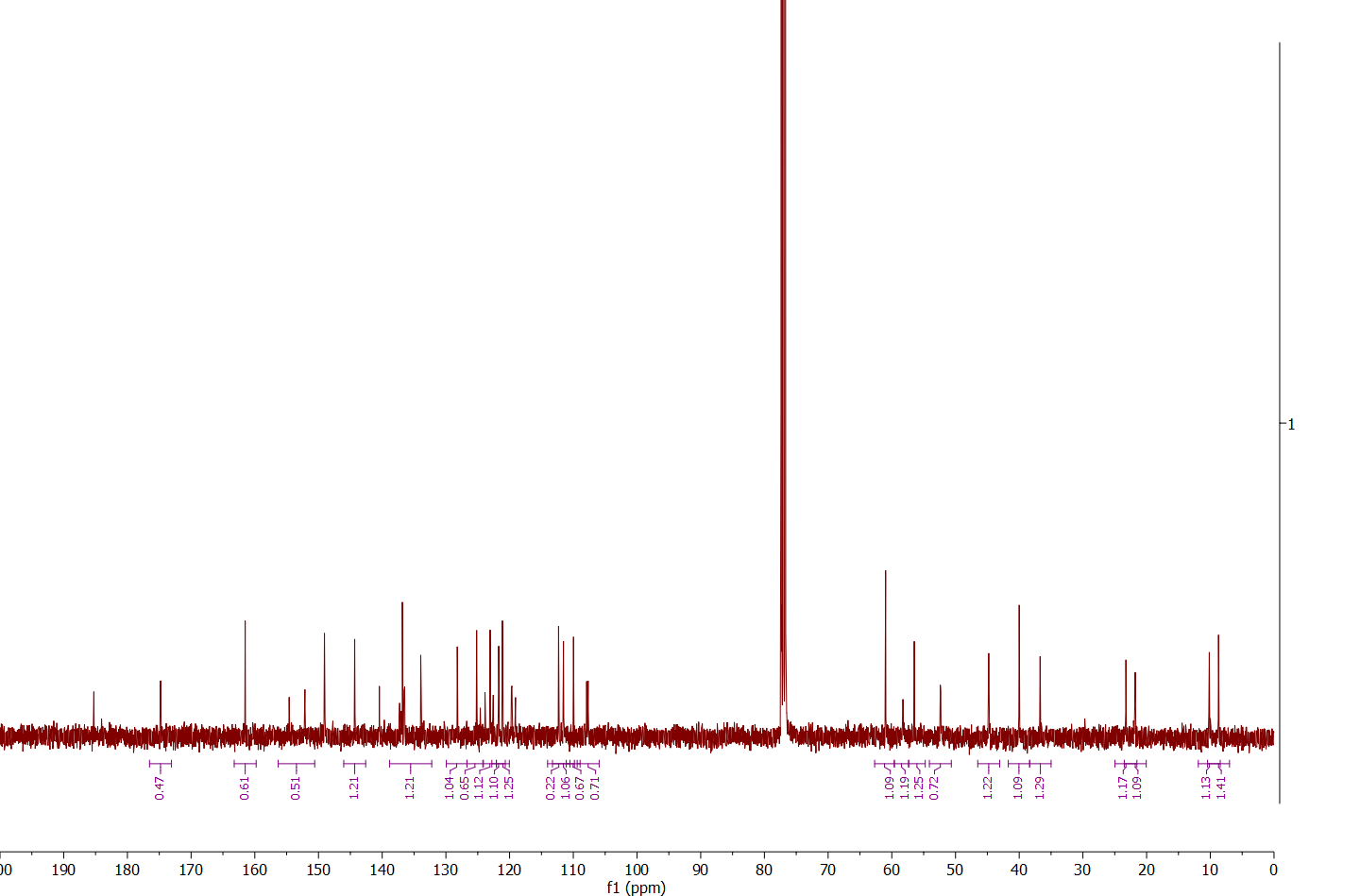


Figure 47S. ^13^C NMR spectrum of **IIIj**.

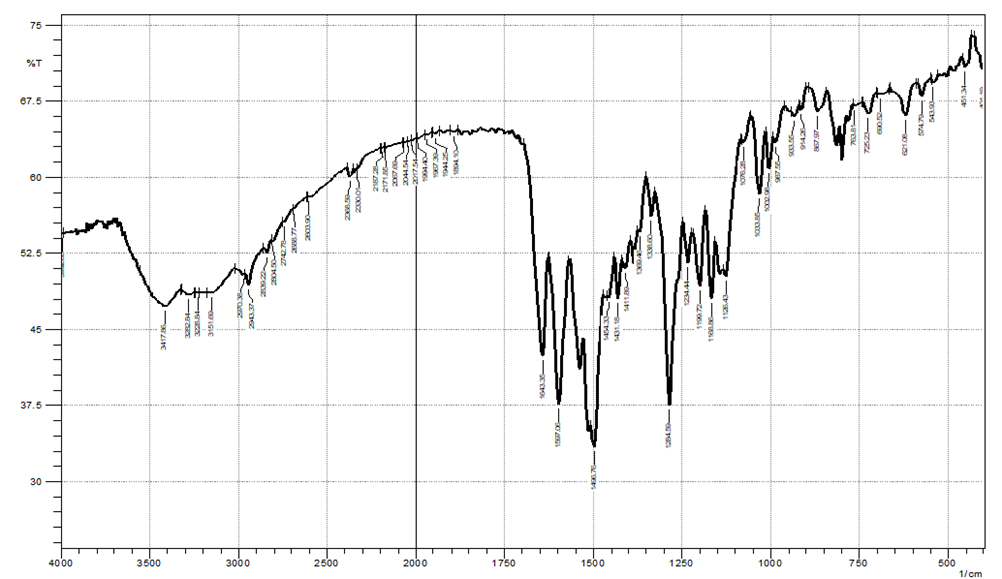


Figure 48S. IR spectrum of **VIa**.


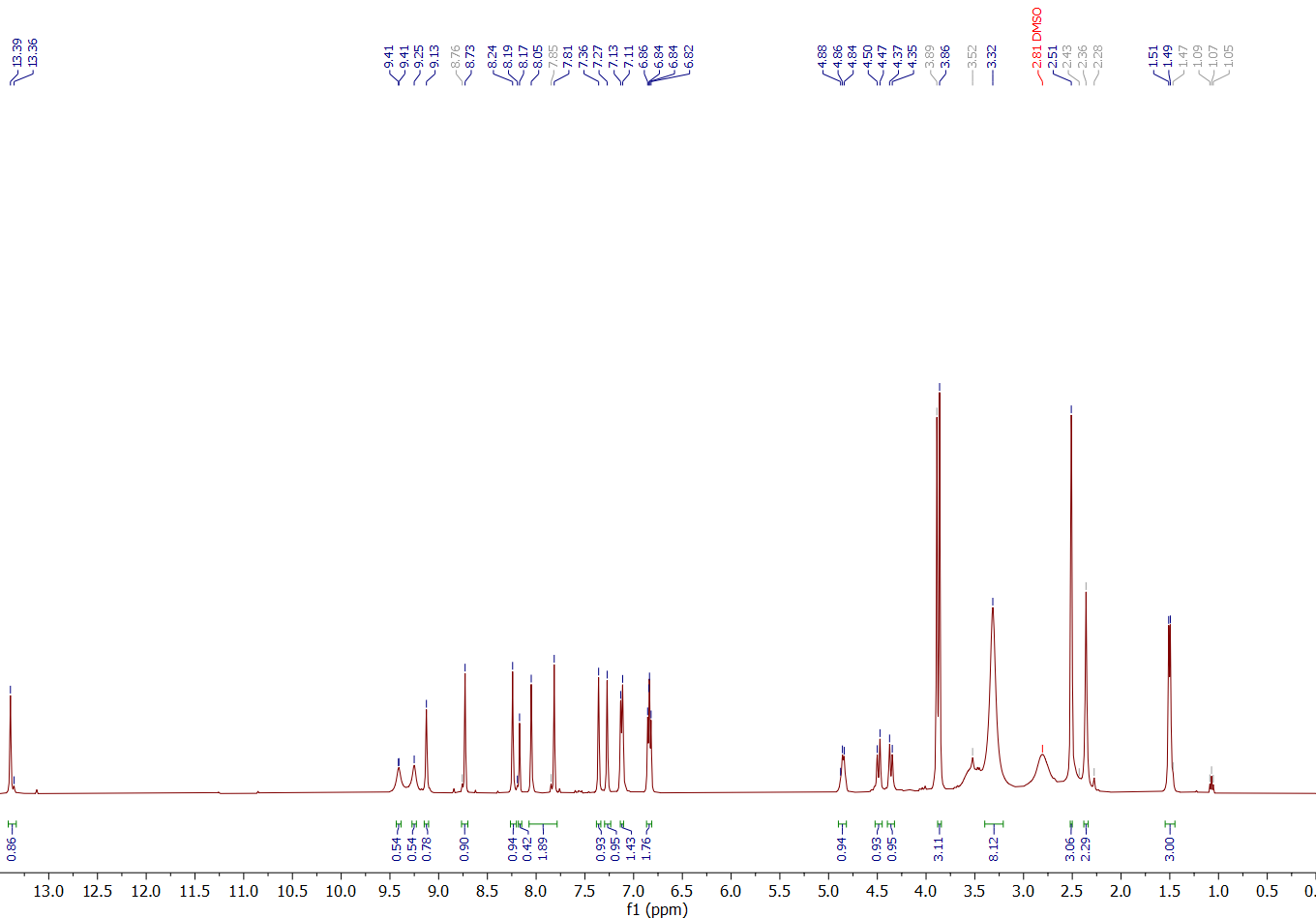


Figure 49S. ^1^H NMR spectrum of **VIa**.

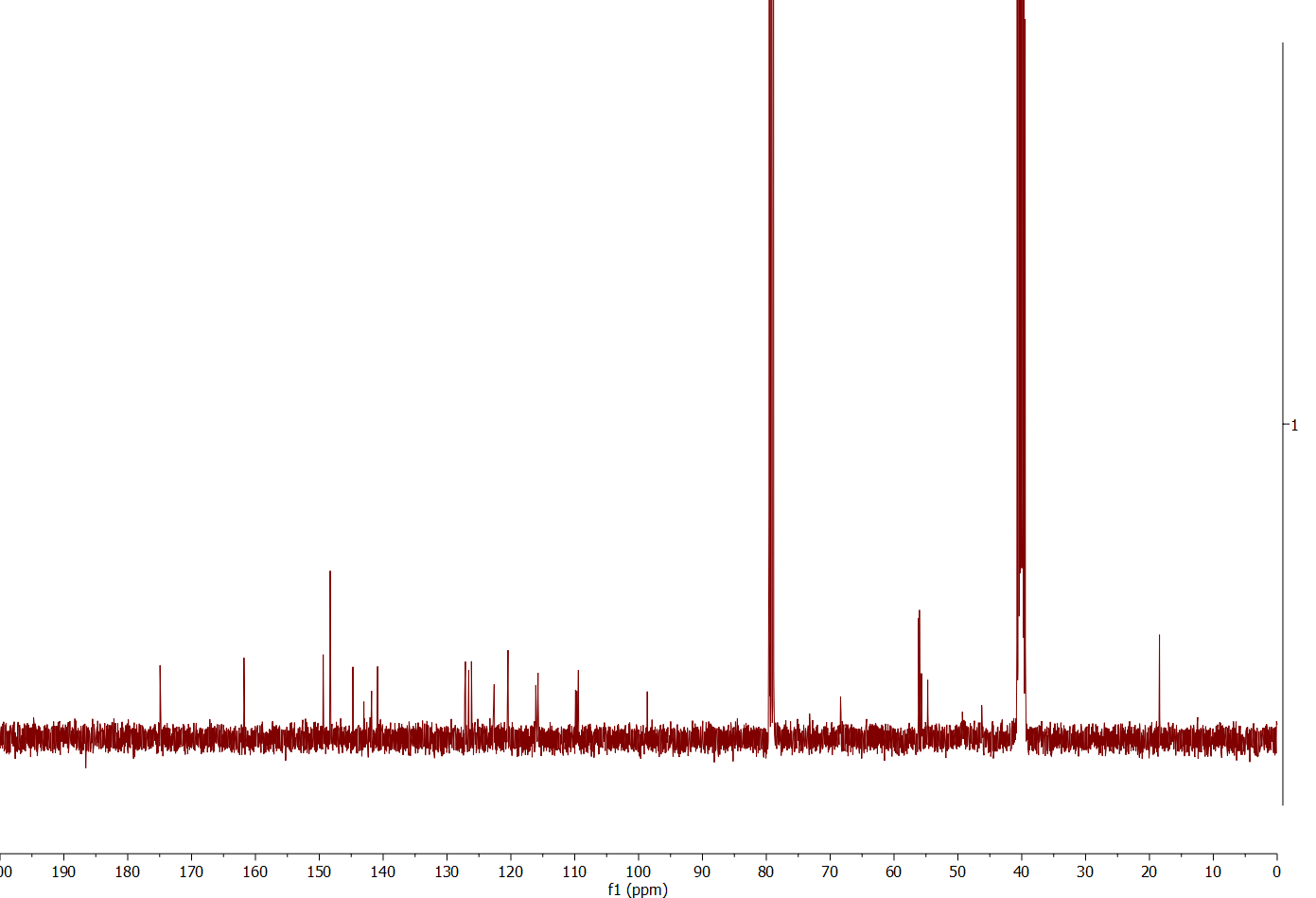


Figure 50S. ^13^C NMR spectrum of **VIa**.

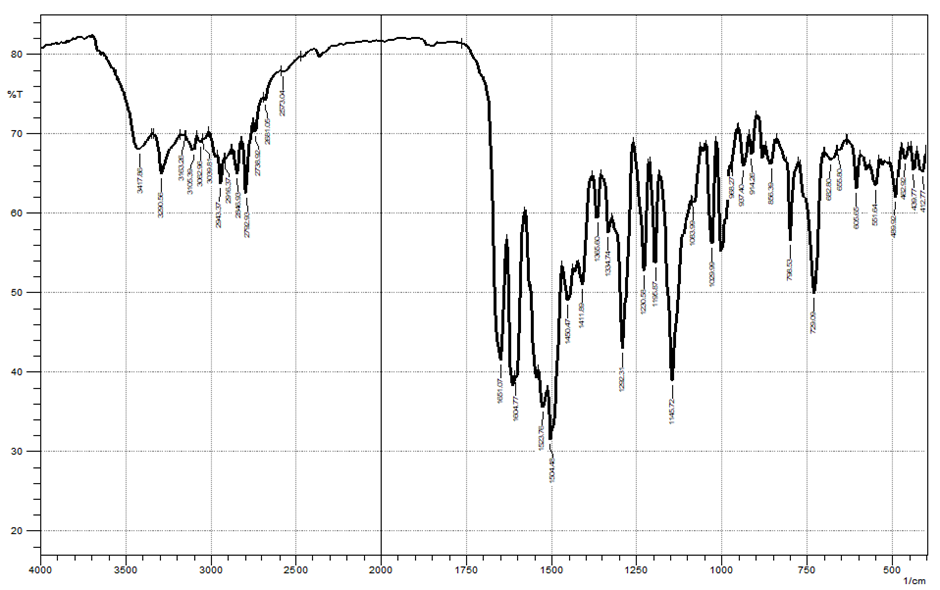


Figure 51S. IR spectrum of **VIb**.


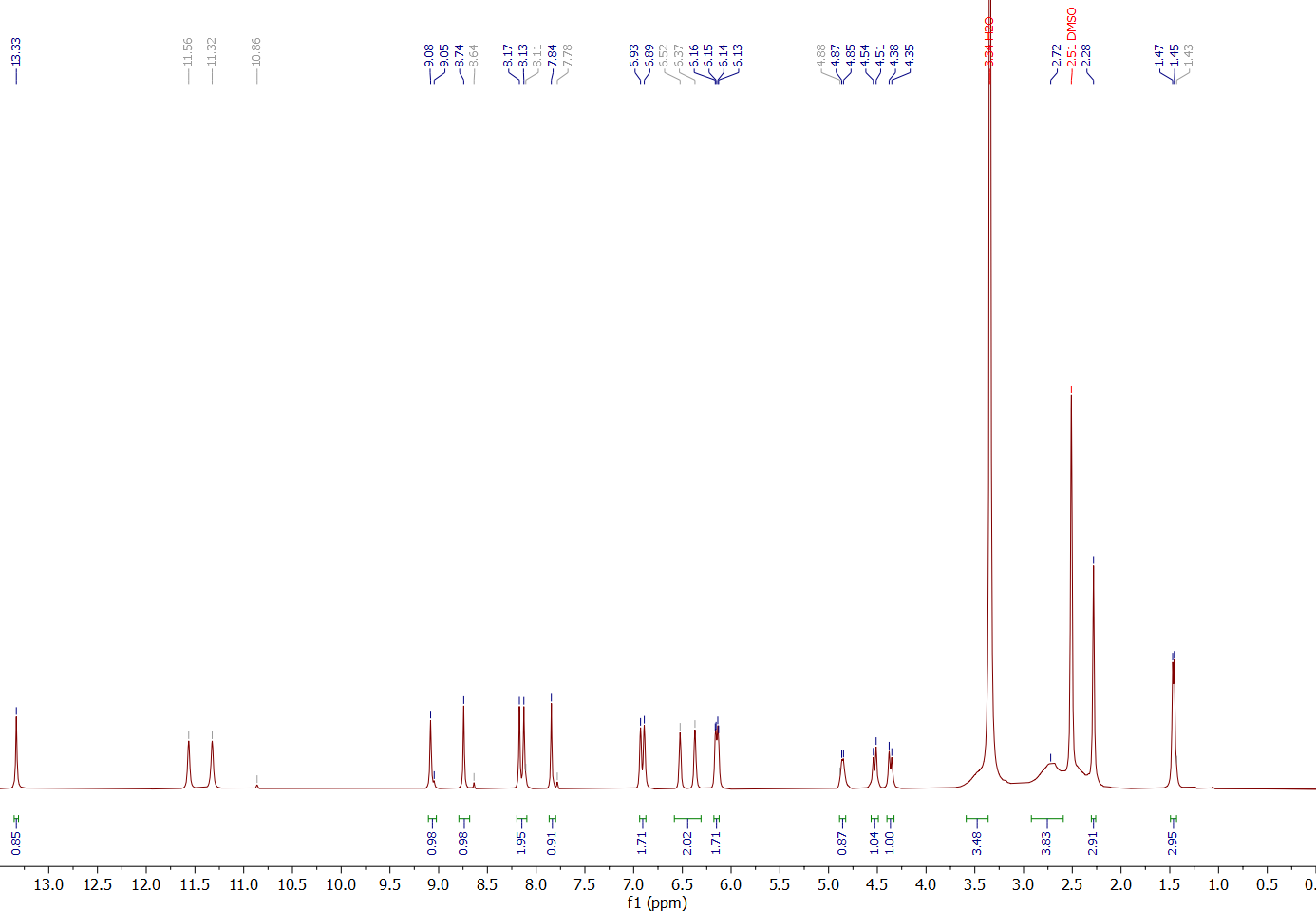


Figure 52S. ^1^H NMR spectrum of **VIb**.


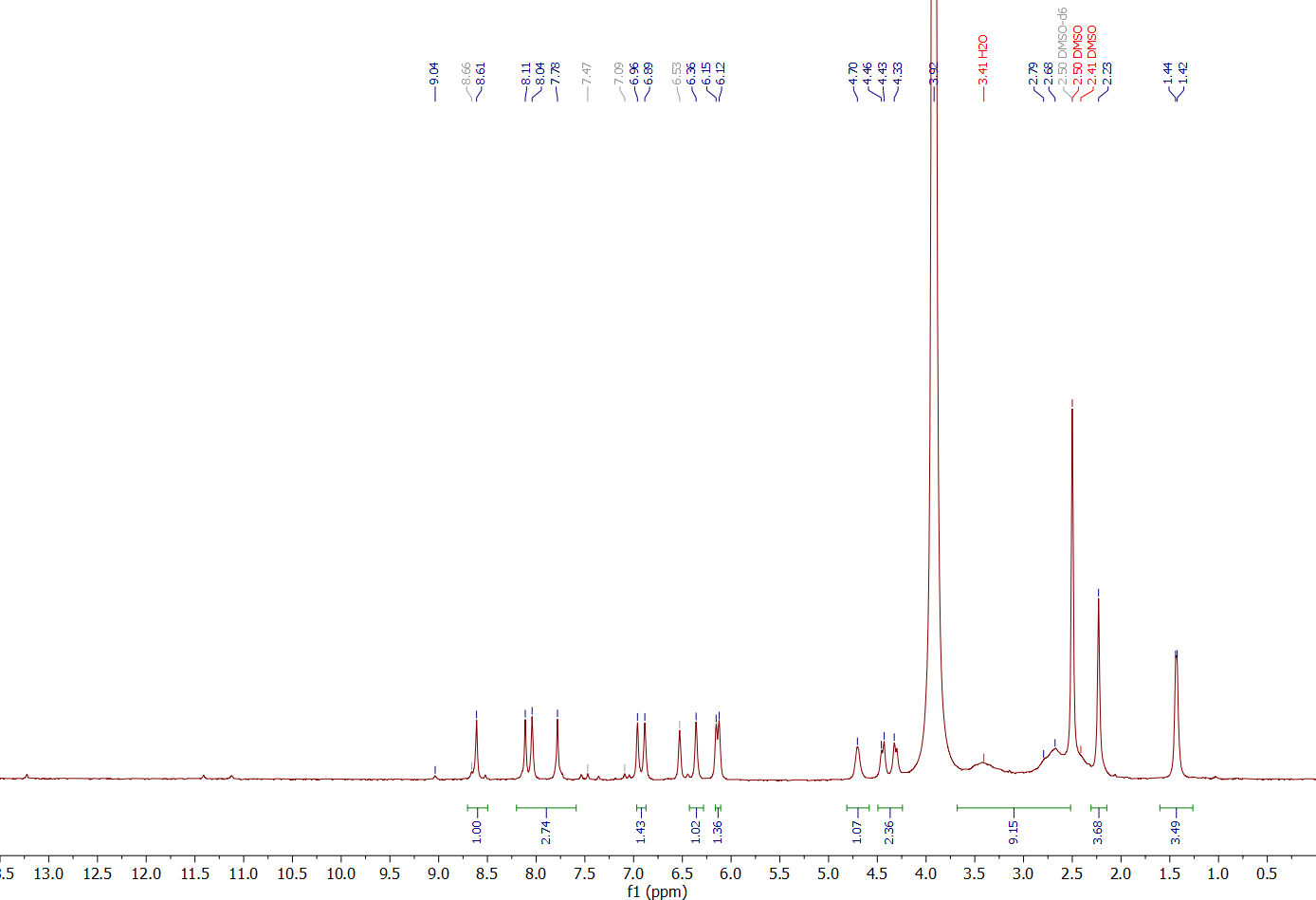


Figure 53S. ^1^H NMR (D_2_O) spectrum of **VIb**.


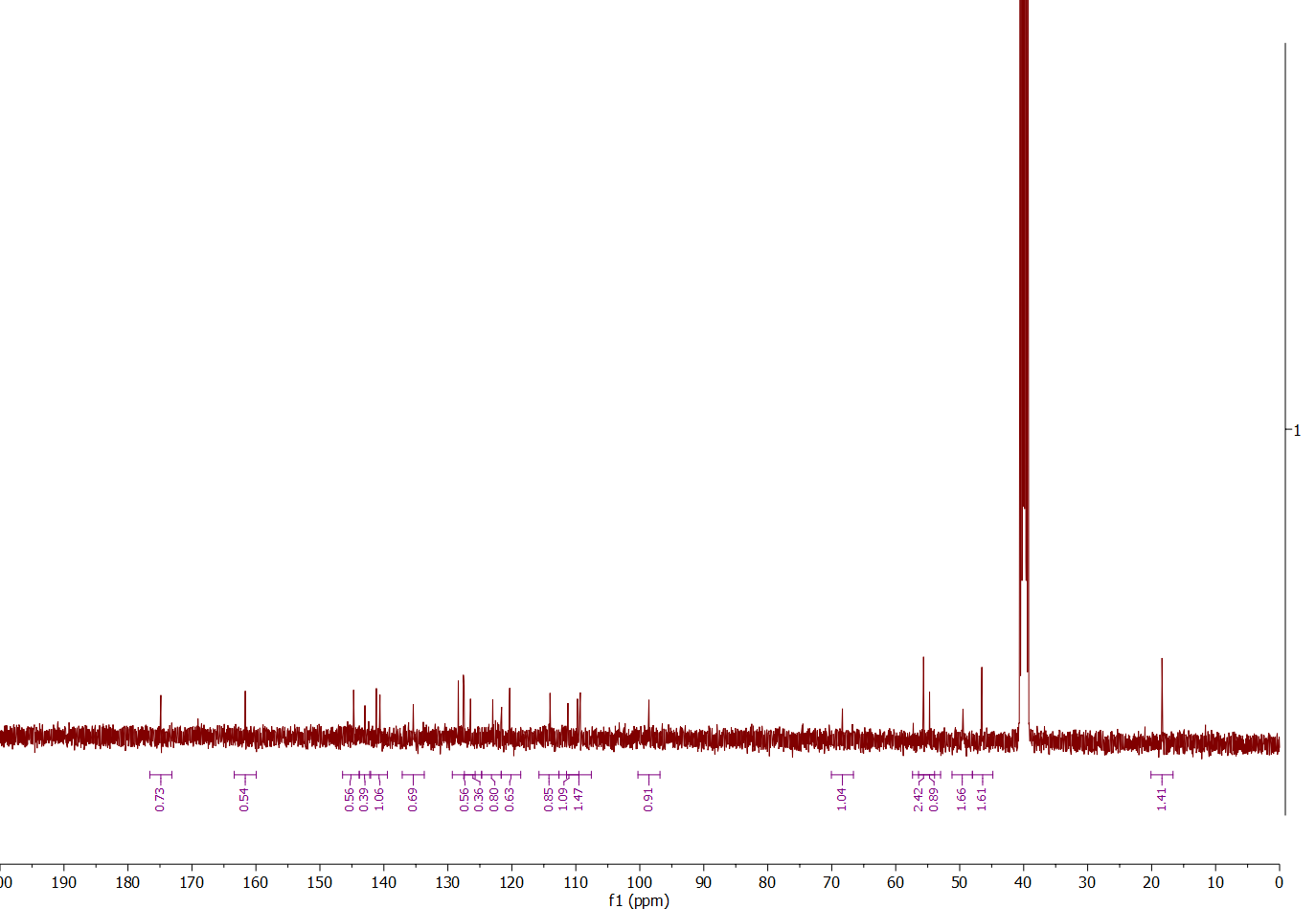


Figure 54S. ^13^C NMR spectrum of **VIb**.

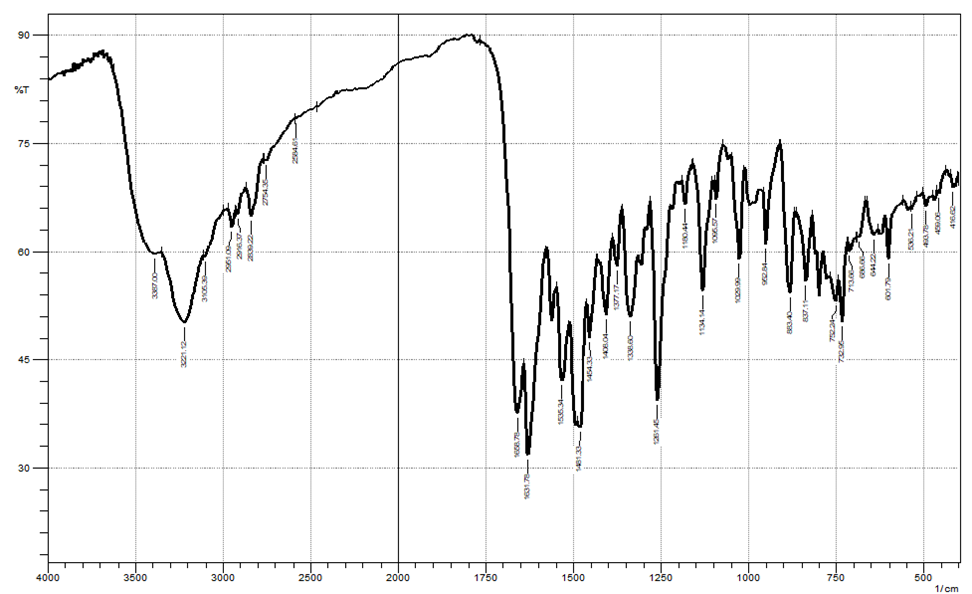


Figure 55S. IR spectrum of **VIIIb**.


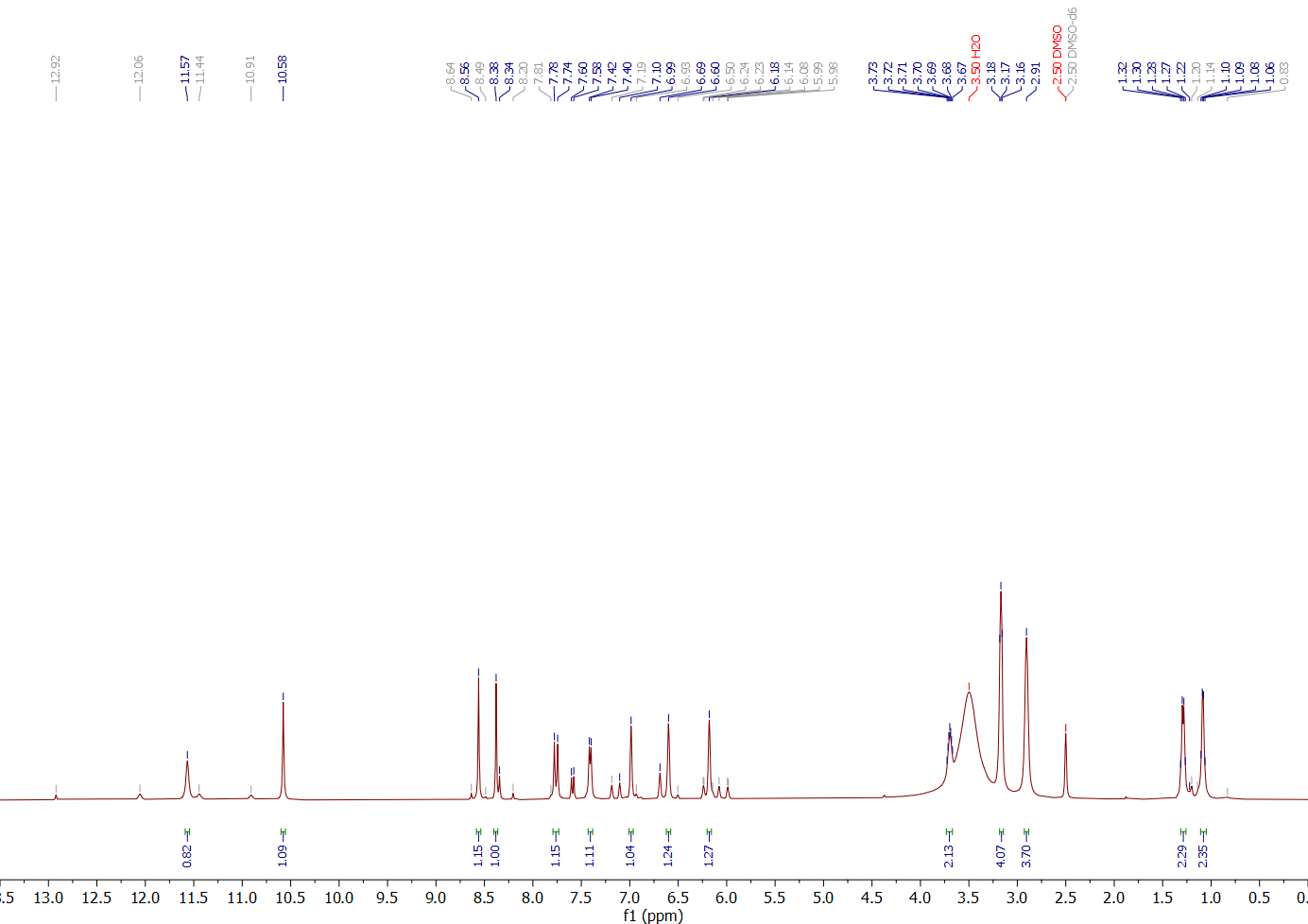


Figure 56S. ^1^H NMR spectrum of **VIIIb**.


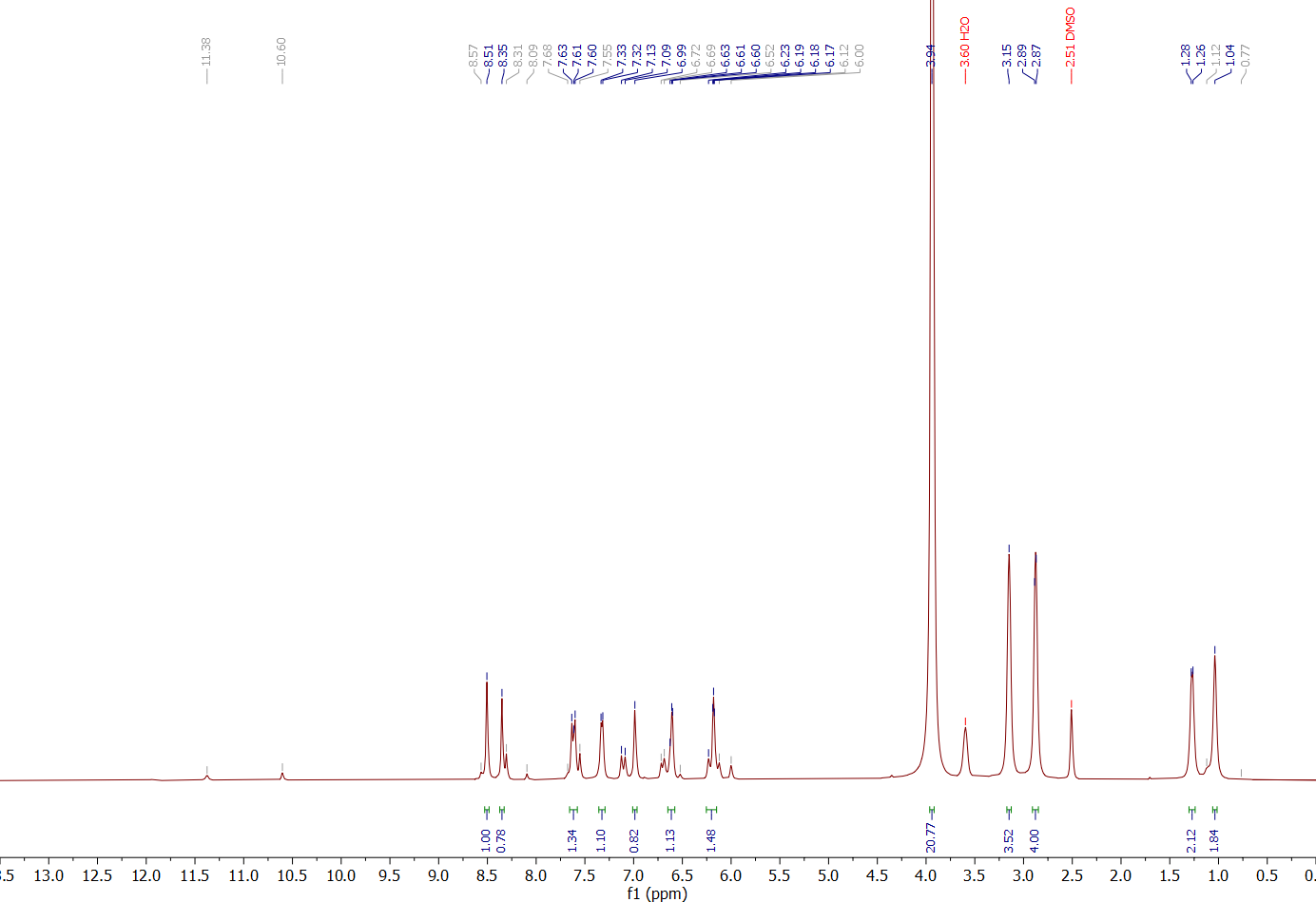

Figure 57S. ^1^H NMR (D_2_O) spectrum of **VIIIb**.
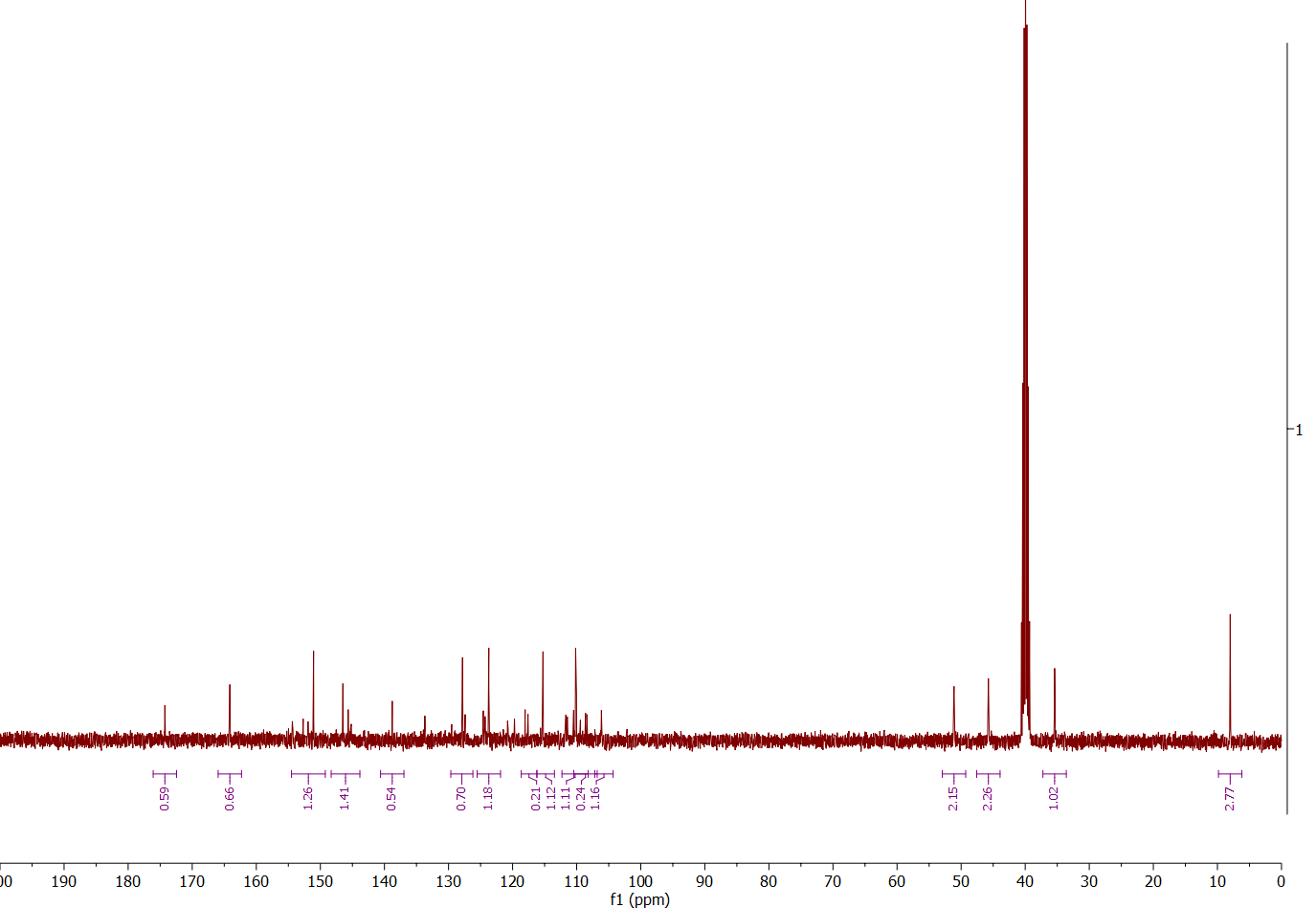


Figure 58S. ^13^C NMR spectrum of **VIIIb**.

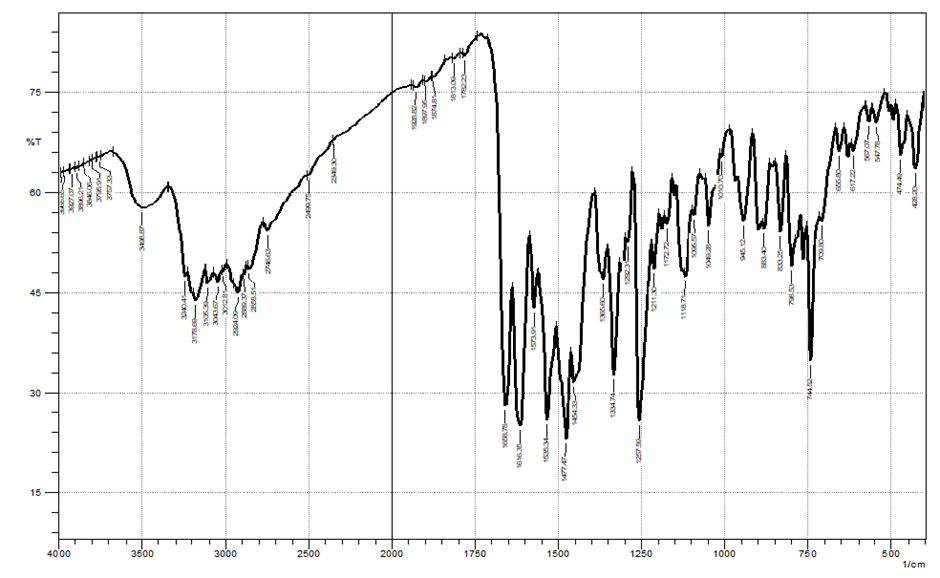


Figure 59S. IR spectrum of **VIIIc**.


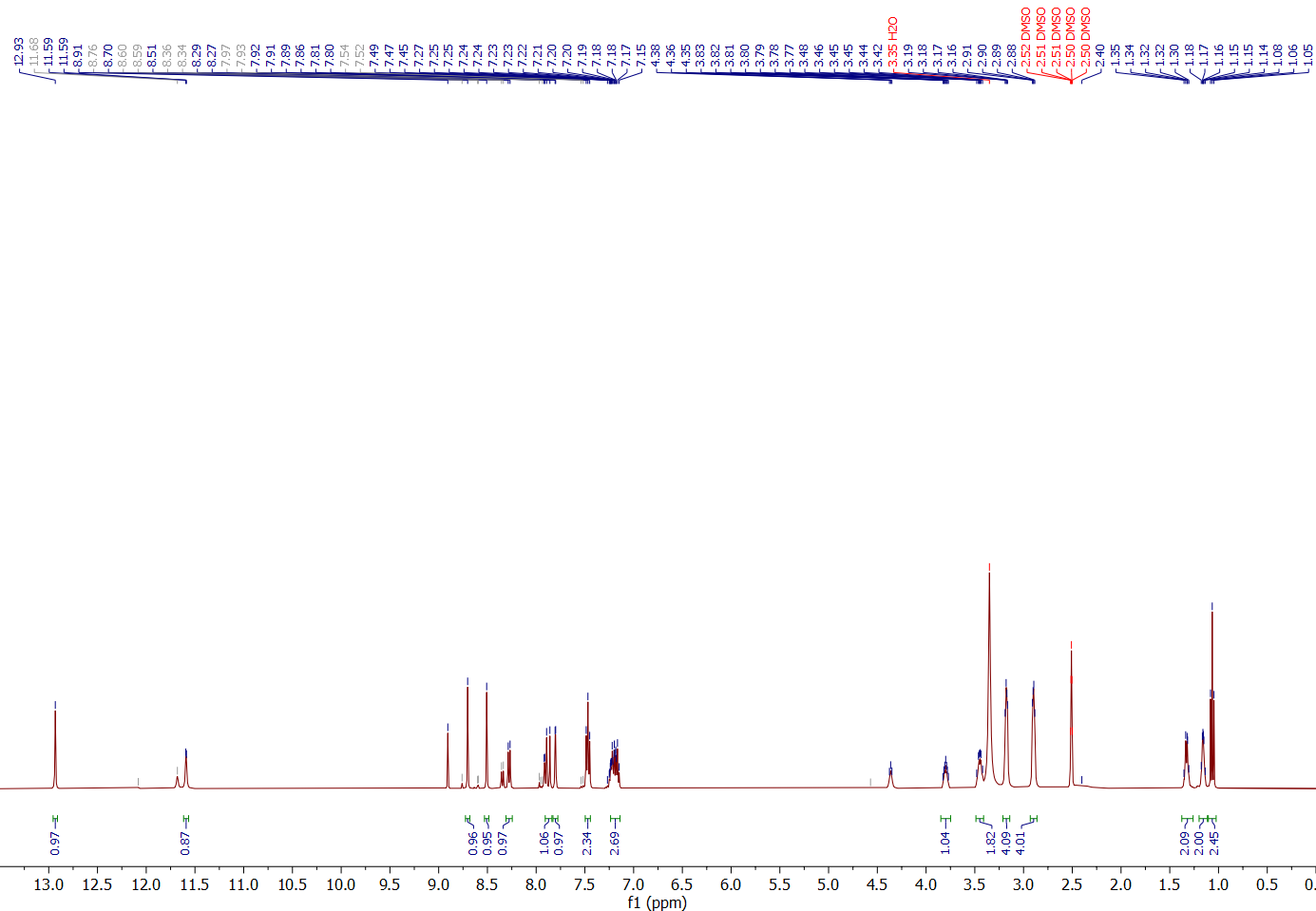


Figure 60S. ^1^H NMR spectrum of **VIIIc**.


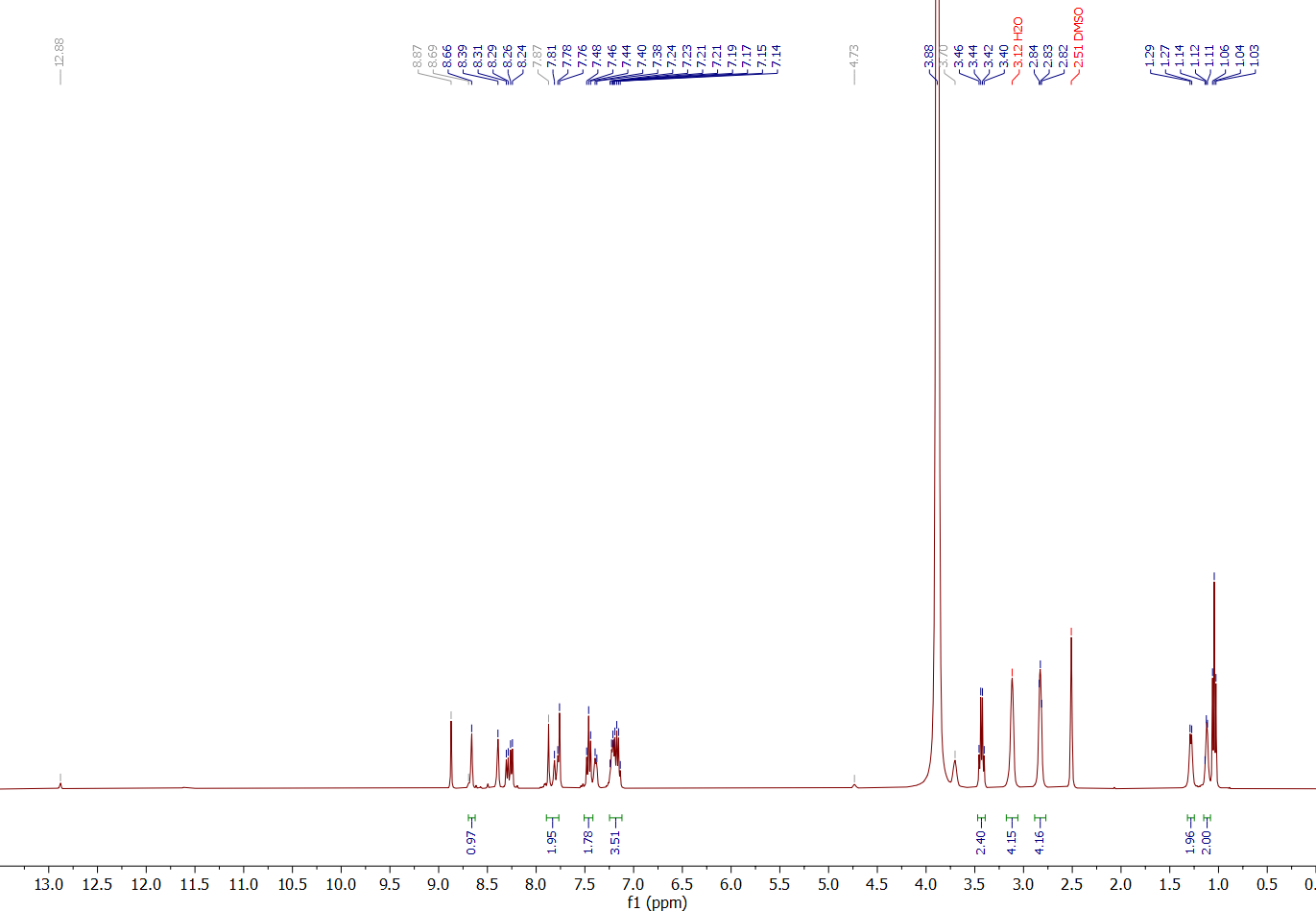


Figure 61S. ^1^H NMR (D_2_O) spectrum of **VIIIc**.


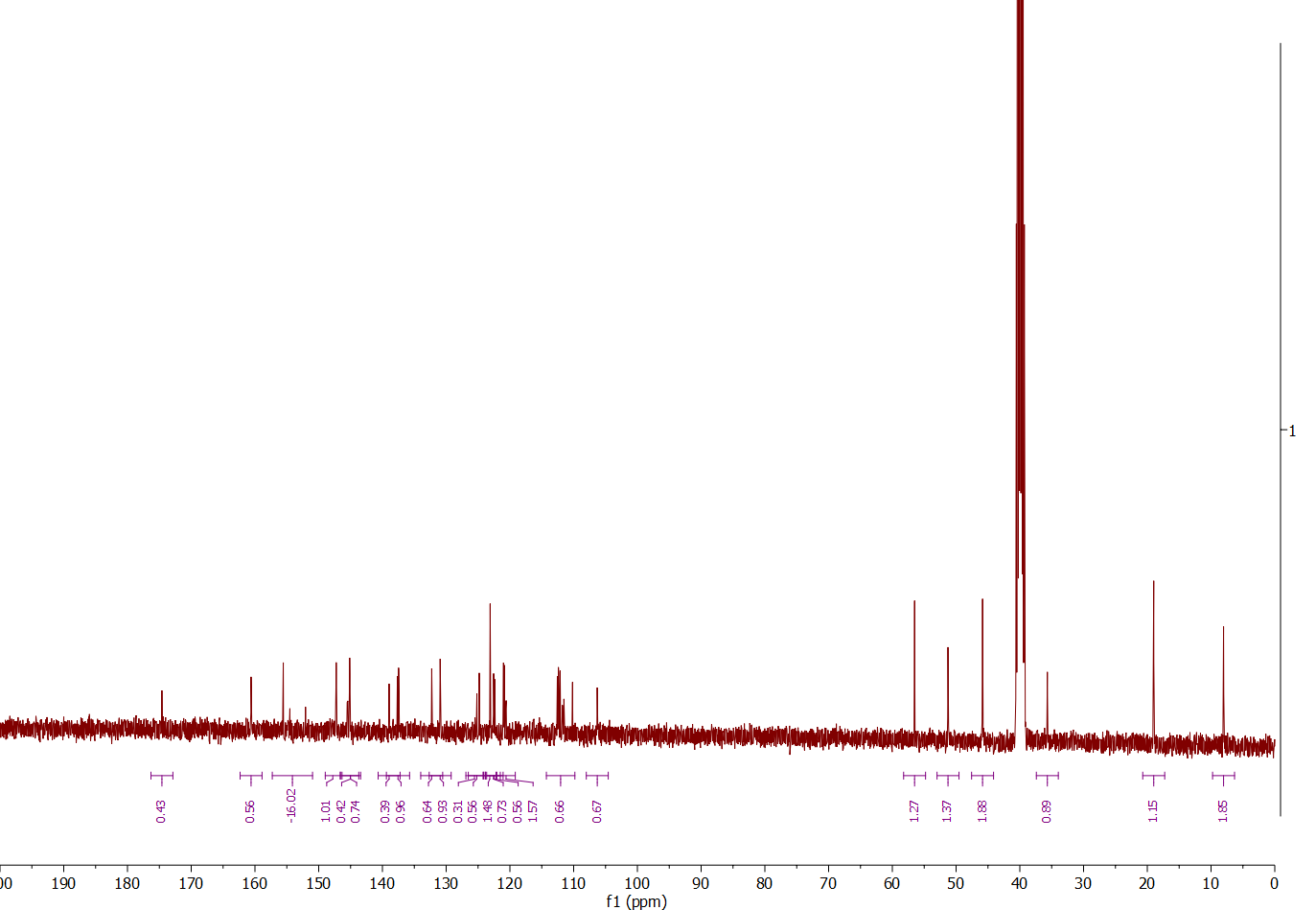


Figure 62S. ^13^C NMR spectrum of **VIIIc**.

Table 1S. Percentage growth inhibition (GI%) of full NCI-60 panel single-dose assay of moxifloxaxin derivatives (**I**, **II**, **IIIa-j** and **IV**).

| Cell line | I | II | IIIa | IIIb | IIIc | IIId | IIIe | IIIf | IIIg | IIIh | IIIi | IIIj | IV |
| --- | --- | --- | --- | --- | --- | --- | --- | --- | --- | --- | --- | --- | --- |
| Leukemia | | | | | | | | | | | | | |
| CCRF-CEM | ---- | 24.21 | 21.79 | 36.21 | 36.43 | 70.39 | 68.35 | 97.47 | 44.74 | 12.1 | --- | 28.84 | ---- |
| HL-60(TB) | ---- | 25.93 | 26.45 | 27.17 | 19.44 | 21.49 | 24.2 | 140.35 | 15.95 | 10.95 | --- | --- | ---- |
| K-562 | ---- | 64.66 | 45.87 | 81.11 | 92.08 | 110.03 | 97.56 | 125.49 | 118.52 | 12.64 | --- | 96.74 | ---- |
| MOLT-4 | ---- | 22.94 | 20.69 | 36.87 | 39.57 | 70.53 | 40.66 | 97.66 | 45.06 | --- | --- | 25.87 | ---- |
| RPMI-8226 | ---- | 25.52 | 26.95 | 39.76 | 35.79 | 37.15 | 39.12 | 125.03 | 34.22 | 13.56 | --- | 21.85 | ---- |
| SR | ---- | 81.35 | 91.8 | 124.61 | 103.21 | 133.52 | 124.33 | 113.43 | 128.12 | --- | --- | 112.25 | ---- |
| Non-Small Cell Lung Cancer | | | | | | | | | | | | | |
| A-549/ATCC | ---- | ---- | --- | --- | --- | 11.28 | 29.83 | 121.44 | 16.89 | --- | --- | --- | ---- |
| EKVX | ---- | 11.24 | --- | 10.04 | --- | 13.78 | 33.42 | 75.08 | --- | --- | --- | --- | ---- |
| HOP-62 | ---- | ---- | --- | --- | --- | --- | --- | --- | 11.57 | --- | --- | --- | ---- |
| HOP-92 | ---- | 16.19 | 24.72 | 49.78 | 25.49 | 30.49 | 32.58 | 20.49 | 42.14 | --- | --- | 20.43 | ---- |
| NCI-H226 | ---- | ---- | --- | --- | --- | --- | 13.5 | 12.7 | --- | --- | --- | --- | ---- |
| NCI-H23 | ---- | ---- | --- | 12.11 | 14.26 | 30.86 | 18.19 | 44.56 | 11.69 | --- | --- | --- | ---- |
| NCI-H322M | ---- | ---- | --- | --- | 10.34 | --- | 11.72 | 44.58 | 11.03 | 10.22 | --- | --- | ---- |
| NCI-H460 | ---- | ---- | --- | --- | --- | 21.72 | 20.77 | 166.65 | 13.96 | --- | --- | --- | ---- |
| NCI-H522 | ---- | ---- | --- | --- | --- | 27.04 | 70.68 | 53.17 | 22.36 | --- | --- | --- | ---- |
| Colon Cancer | | | | | | | | | | | | | |
| COLO 205 | ---- | ---- | --- | --- | --- | --- | 14.54 | 64.42 | --- | --- | --- | --- | ---- |
| HCC-2998 | ---- | ---- | --- | --- | --- | 10.57 | 45.21 | 85.18 | --- | --- | --- | --- | ---- |
| HCT-116 | ---- | ---- | 18.91 | 32.1 | 46.4 | 68.67 | 67.33 | 159.07 | 49.47 | --- | --- | 43.31 | ---- |
| HCT-15 | ---- | 23.35 | 23.65 | 54.15 | 52.44 | 83.58 | 96.3 | 67.99 | 98.24 | --- | 16.36 | 42.57 | ---- |
| HT29 | ---- | 23.54 | 27.42 | 54.12 | 67.61 | 112.01 | 119.16 | 100.26 | 86.41 | --- | --- | 49.48 | ---- |
| KM12 | ---- | ---- | --- | --- | --- | 25.54 | 12.03 | 30.84 | --- | --- | --- | --- | ---- |
| SW-620 | ---- | ---- | --- | --- | --- | 19.28 | 52.89 | 99.56 | 10.4 | --- | --- | --- | ---- |
| CNS Cancer | | | | | | | | | | | | | |
| SF-268 | ---- | 13.94 | 14.8 | 17.1 | 16.42 | 14.53 | 20.59 | 17.9 | 17.6 | 10.24 | 14.76 | --- | ---- |
| SF-295 | ---- | ---- | --- | --- | --- | --- | 55.79 | --- | --- | --- | --- | --- | ---- |
| SF-539 | ---- | ---- | 16.29 | 12.26 | 64.51 | 108.68 | 160.41 | 109.11 | 27.26 | --- | --- | 11.78 | ---- |
| SNB-19 | ---- | ---- | --- | --- | --- | 21.75 | 19.51 | 73.77 | 11.7 | --- | --- | --- | ---- |
| SNB-75 | ---- | ---- | 11.32 | 16.45 | --- | --- | --- | --- | --- | --- | --- | --- | ---- |
| U251 | ---- | ---- | --- | 18.56 | 10.57 | 50.97 | 87.59 | 166.99 | 13.06 | --- | --- | --- | ---- |
| Melanoma | | | | | | | | | | | | | |
| LOX IMVI | ---- | 19.45 | 16.27 | 53.93 | 80.38 | 122.58 | 164.34 | 183.52 | 173.53 | --- | --- | --- | ---- |
| MALME-3M | ---- | ---- | --- | 13.72 | --- | --- | --- | 10.83 | 17.18 | --- | --- | --- | ---- |
| M14 | ---- | ---- | --- | --- | --- | --- | --- | --- | --- | --- | --- | --- | ---- |
| MDA-MB-435 | ---- | ---- | --- | --- | --- | --- | --- | --- | 15.31 | --- | --- | --- | ---- |
| SK-MEL-2 | ---- | ---- | --- | --- | --- | --- | --- | --- | --- | --- | --- | --- | ---- |
| SK-MEL-28 | ---- | ---- | --- | 19.03 | --- | --- | --- | --- | 13.76 | --- | --- | --- | ---- |
| SK-MEL-5 | ---- | 13.4 | 12.17 | 17.32 | 12.85 | 16.09 | 77.25 | --- | 16 | --- | 12.6 | --- | ---- |
| UACC-257 | ---- | ---- | --- | 20.5 | --- | --- | 14.74 | --- | --- | --- | --- | --- | ---- |
| UACC-62 | ---- | ---- | --- | 19.76 | --- | --- | --- | --- | 57.04 | --- | --- | --- | ---- |
| Ovarian Cancer | | | | | | | | | | | | | |
| IGROV1 | ---- | ---- | --- | --- | 24.67 | 41.11 | 48.98 | 83.64 | 24.77 | --- | --- | --- | ---- |
| OVCAR-3 | ---- | ---- | --- | --- | 16.94 | 48.76 | 70.64 | 173 | 18.3 | --- | --- | --- | ---- |
| OVCAR-4 | ---- | ---- | --- | --- | 12.13 | 10.02 | --- | 152.83 | --- | --- | --- | --- | ---- |
| OVCAR-5 | ---- | ---- | --- | --- | --- | --- | 12.7 | 61.57 | --- | --- | --- | --- | ---- |
| OVCAR-8 | ---- | ---- | --- | --- | --- | 12.7 | 46.05 | 25.83 | --- | --- | --- | --- | ---- |
| NCI/ADR-RES | ---- | ---- | --- | 10.57 | --- | 18.41 | 36.41 | --- | --- | --- | --- | --- | ---- |
| SK-OV-3 | ---- | ---- | --- | --- | --- | --- | --- | --- | --- | --- | --- | --- | ---- |
| Renal Cancer | | | | | | | | | | | | | |
| 786-0 | ---- | 33.57 | 33.39 | 30.75 | 15.82 | 62.3 | 49.7 | 162.15 | 14.7 | --- | --- | --- | ---- |
| A498 | 25.91 | 31.14 | 25.07 | 40.11 | 16.66 | --- | --- | --- | 25.69 | 18.01 | --- | --- | 20.22 |
| ACHN | ---- | ---- | --- | --- | 11.28 | 53.17 | 14.7 | 64.74 | 10.79 | --- | --- | --- | ---- |
| CAKI-1 | ---- | 10.98 | --- | 12.3 | 17.1 | --- | 17.08 | 17.11 | 21.24 | --- | 11.35 | --- | ---- |
| RXF 393 | ---- | ---- | --- | --- | --- | --- | --- | --- | --- | --- | --- | --- | ---- |
| SN12C | ---- | ---- | --- | 11.63 | 19.02 | 34.48 | --- | 76.23 | --- | --- | --- | --- | ---- |
| TK-10 | ---- | ---- | --- | --- | --- | --- | 35.1 | --- | 39.54 | --- | 18.92 | --- | ---- |
| UO-31 | 12.42 | 19.91 | 13.61 | 19.3 | 37.61 | 40.85 | 39.87 | 18.89 | 34.9 | 19.16 | --- | 22.84 | ---- |
| Prostate Cancer | | | | | | | | | | | | | |
| PC-3 | ---- | 14.56 | 13.09 | 23.64 | 24.9 | 39.63 | 31.11 | 54.87 | 14.48 | 13.17 | --- | 23.07 | ---- |
| DU-145 | ---- | ---- | --- | --- | --- | 26.4 | 85.38 | 53.52 | 47.55 | --- | 27.69 | --- | ---- |
| Breast Cancer | | | | | | | | | | | | | |
| MCF-7 | ---- | 12.6 | 15.87 | 24.15 | 29.22 | 55.89 | 58.71 | 127.66 | 43.88 | --- | --- | 44.96 | ---- |
| MDA-MB-231/ATCC | ---- | ---- | --- | 17.67 | 18.87 | 30.94 | --- | 127.35 | 11.57 | --- | --- | --- | ---- |
| HS 578T | ---- | ---- | --- | --- | --- | --- | --- | --- | --- | --- | --- | --- | ---- |
| BT-549 | ---- | ---- | --- | 15.2 | --- | --- | 23.33 | --- | 15.71 | --- | --- | --- | ---- |
| T-47D | ---- | ---- | --- | --- | 14.13 | --- | 36.94 | 130.39 | --- | --- | 11.84 | --- | ---- |
| MDA-MB-468 | ---- | 21.86 | 16.34 | 15.62 | 10.58 | 21.04 | --- | 45.16 | --- | --- | --- | --- | ---- |
| *Values below 10% are represented by (---). | | | | | | | | | | | | | |

Table 2S. Percentage growth inhibition (GI%) of full NCI-60 panel single-dose assay of (**VIa-c**) and (**VIIIa-c**).

| Cell line | VIa | VIb | VIc | VIIIa | VIIIb | VIIIc |
| --- | --- | --- | --- | --- | --- | --- |
| Leukemia | | | | | | |
| CCRF-CEM | 20.7 | 135.91 | --- | --- | 11.62 | 22.05 |
| HL-60(TB) | 16.66 | 79.68 | --- | --- | --- | --- |
| K-562 | 10.92 | 127.77 | 24.89 | 25.78 | --- | --- |
| MOLT-4 | 17.22 | 90.6 | --- | --- | --- | --- |
| RPMI-8226 | --- | 54.7 | --- | --- | --- | --- |
| SR | 13.51 | --- | --- | --- | --- | --- |
| Non-Small Cell Lung Cancer | | | | | | |
| A-549/ATCC | --- | 105.87 | --- | --- | --- | --- |
| EKVX | --- | 66.73 | --- | --- | --- | --- |
| HOP-62 | --- | 61.96 | --- | --- | --- | --- |
| HOP-92 | 73.81 | 71.96 | --- | 21.16 | 17.3 | 19.02 |
| NCI-H226 | --- | 50.01 | --- | 18.89 | --- | --- |
| NCI-H23 | --- | 58.86 | --- | --- | --- | --- |
| NCI-H322M | --- | 34.3 | --- | --- | --- | --- |
| NCI-H460 | --- | 99.54 | --- | --- | --- | --- |
| NCI-H522 | --- | 86.15 | --- | --- | --- | --- |
| Colon Cancer | | | | | | |
| COLO 205 | --- | 45.56 | --- | --- | --- | --- |
| HCC-2998 | --- | 77.4 | --- | --- | --- | --- |
| HCT-116 | --- | 180.12 | 10.95 | --- | --- | --- |
| HCT-15 | --- | 52.95 | --- | --- | --- | --- |
| HT29 | --- | 85.38 | --- | --- | --- | --- |
| KM12 | --- | 53.57 | --- | --- | --- | --- |
| SW-620 | --- | 146.35 | 15.84 | --- | --- | --- |
| CNS Cancer | | | | | | |
| SF-268 | --- | 40.88 | --- | 14.13 | --- | --- |
| SF-295 | --- | 29.72 | --- | --- | --- | --- |
| SF-539 | 12.42 | 61.52 | 10.99 | --- | --- | --- |
| SNB-19 | --- | 90.99 | --- | 14.23 | --- | --- |
| SNB-75 | --- | 25.58 | --- | --- | --- | --- |
| U251 | --- | 141.64 | --- | --- | --- | --- |
| Melanoma | | | | | | |
| LOX IMVI | --- | 188.5 | --- | --- | --- | --- |
| MALME-3M | 12.44 | 103.02 | --- | --- | --- | --- |
| M14 | --- | 179.82 | --- | --- | --- | --- |
| MDA-MB-435 | --- | 87.78 | --- | --- | --- | --- |
| SK-MEL-2 | --- | 16.51 | --- | --- | --- | --- |
| SK-MEL-28 | --- | 42.67 | --- | --- | --- | --- |
| SK-MEL-5 | 19.86 | 74.73 | --- | --- | --- | --- |
| UACC-257 | --- | 174.51 | --- | --- | --- | --- |
| UACC-62 | --- | 24.04 | --- | --- | --- | --- |
| Ovarian Cancer | | | | | | |
| IGROV1 | --- | 73.37 | --- | --- | --- | --- |
| OVCAR-3 | --- | 59.81 | --- | --- | --- | --- |
| OVCAR-4 | --- | 55.39 | --- | --- | --- | --- |
| OVCAR-5 | --- | 22.16 | --- | --- | --- | --- |
| OVCAR-8 | --- | 80.55 | --- | --- | --- | --- |
| NCI/ADR-RES | --- | --- | --- | --- | --- | --- |
| SK-OV-3 | --- | 38.41 | --- | --- | --- | --- |
| Renal Cancer | | | | | | |
| 786-0 | --- | 98.48 | --- | --- | --- | --- |
| A498 | --- | 59.38 | 29.47 | --- | --- | --- |
| ACHN | --- | 56.23 | --- | --- | --- | --- |
| CAKI-1 | 11.51 | 52.44 | --- | 12.49 | 10.02 | 11.72 |
| RXF 393 | 17.5 | 84.03 | --- | --- | --- | --- |
| SN12C | --- | 50.67 | 22.16 | 10.25 | --- | --- |
| TK-10 | --- | 12.16 | --- | --- | --- | --- |
| UO-31 | --- | 46.04 | --- | 10.5 | 14.38 | 18.39 |
| Prostate Cancer | | | | | | |
| PC-3 | --- | 72.08 | --- | --- | 12.55 | --- |
| DU-145 | --- | 58.04 | 10.08 | --- | --- | --- |
| Breast Cancer | | | | | | |
| MCF-7 | --- | 105.35 | --- | --- | --- | 23.65 |
| MDA-MB-231/ATCC | --- | 55.58 | --- | 12.45 | 14.91 | 16.39 |
| HS 578T | --- | 32.51 | --- | --- | --- | --- |
| BT-549 | --- | 40.46 | --- | --- | --- | --- |
| T-47D | --- | 52.8 | --- | --- | --- | 14.28 |
| MDA-MB-468 | 20.32 | 109.35 | --- | 16.07 | --- | --- |
| *Values below 10% are represented by (---). | | | | | | |


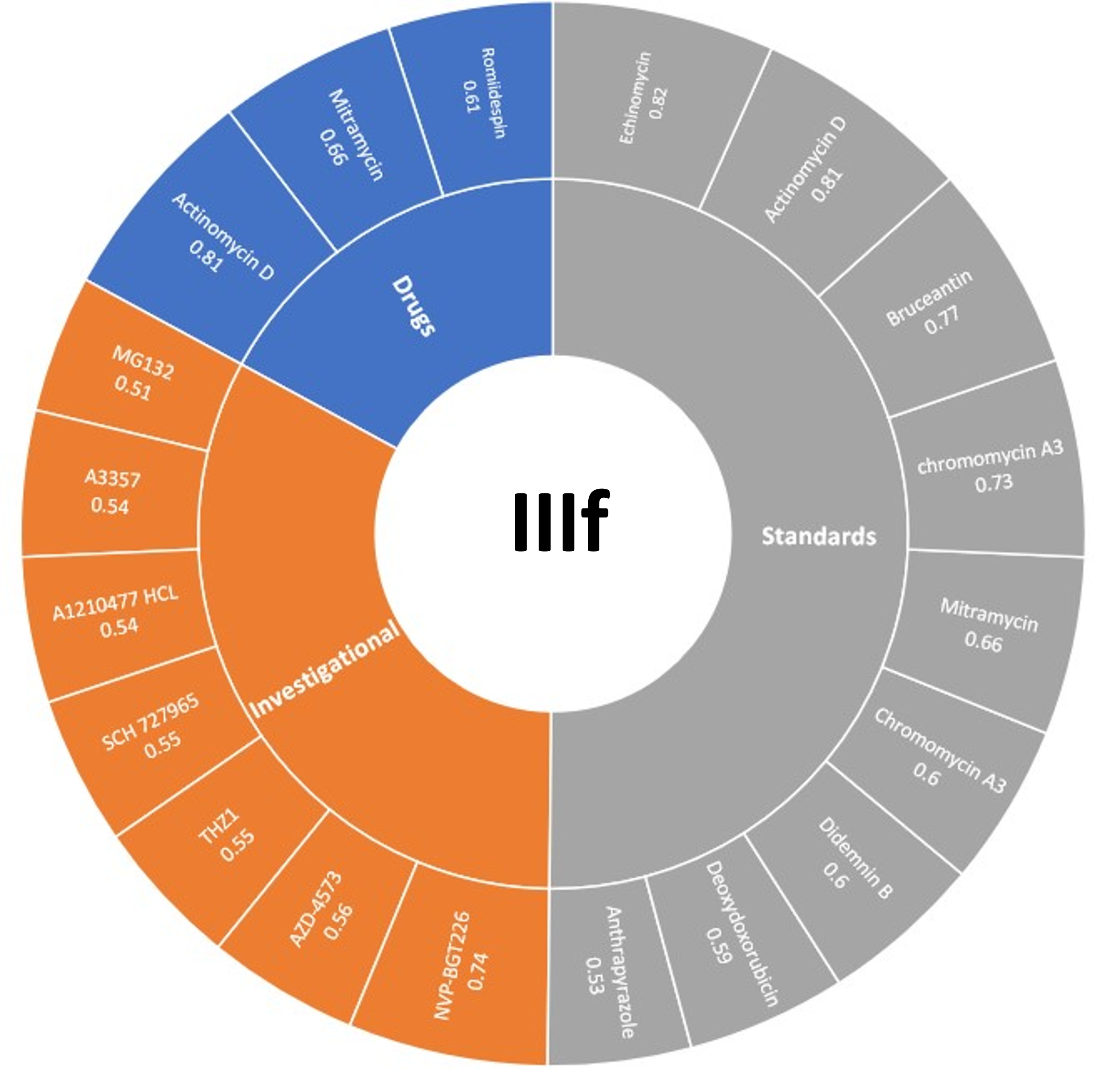


Figure 63S. COMPARE analysis results of **IIIf**.


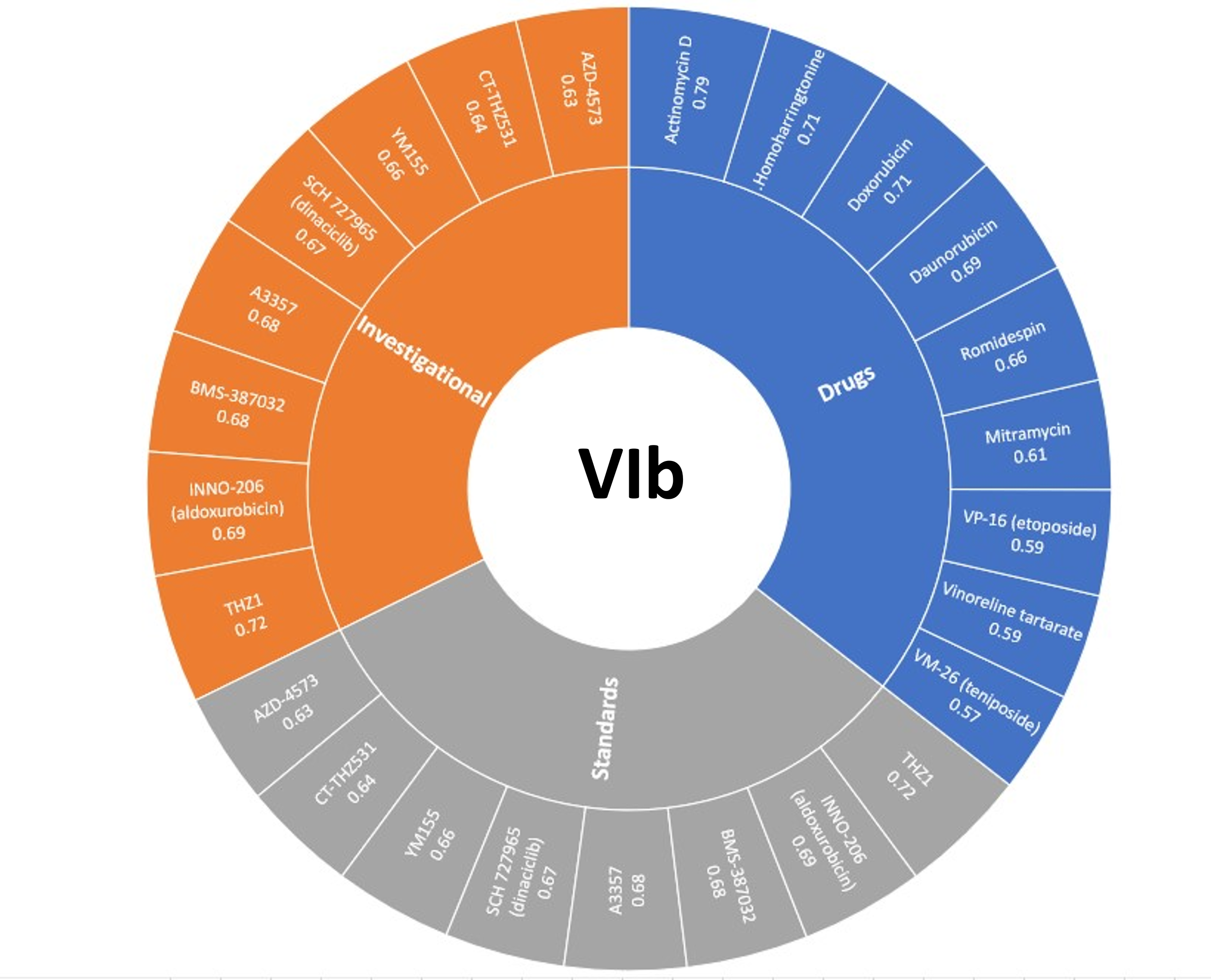


Figure 64S. COMPARE analysis results of **VIb**.


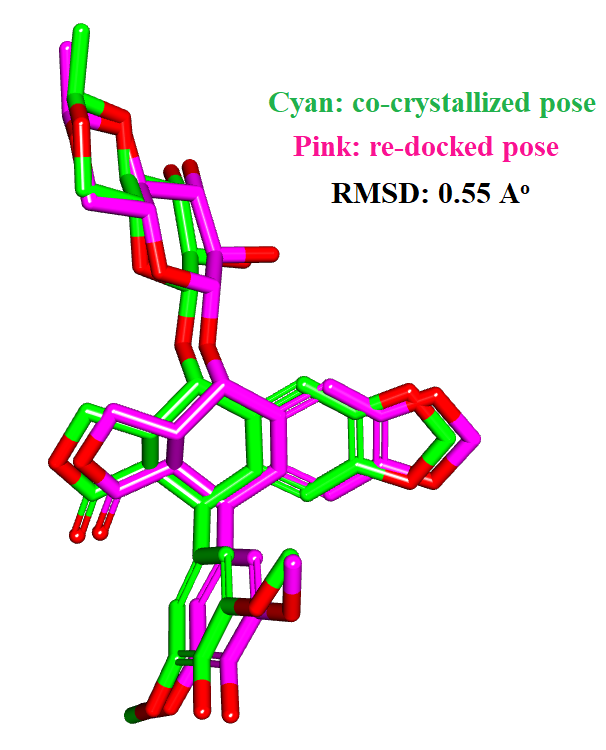


Figure 65S. Superimposition of the co-ordinates of co-crystallized etoposide (green) and its docked pose (pink), showing RMSD of 0.55 Å. (Dassault Systèmes. (2021). BIOVIA Discovery Studio Visualizer was used to generate this figure, https://discover.3ds.com/discovery-studio-visualizer-download )


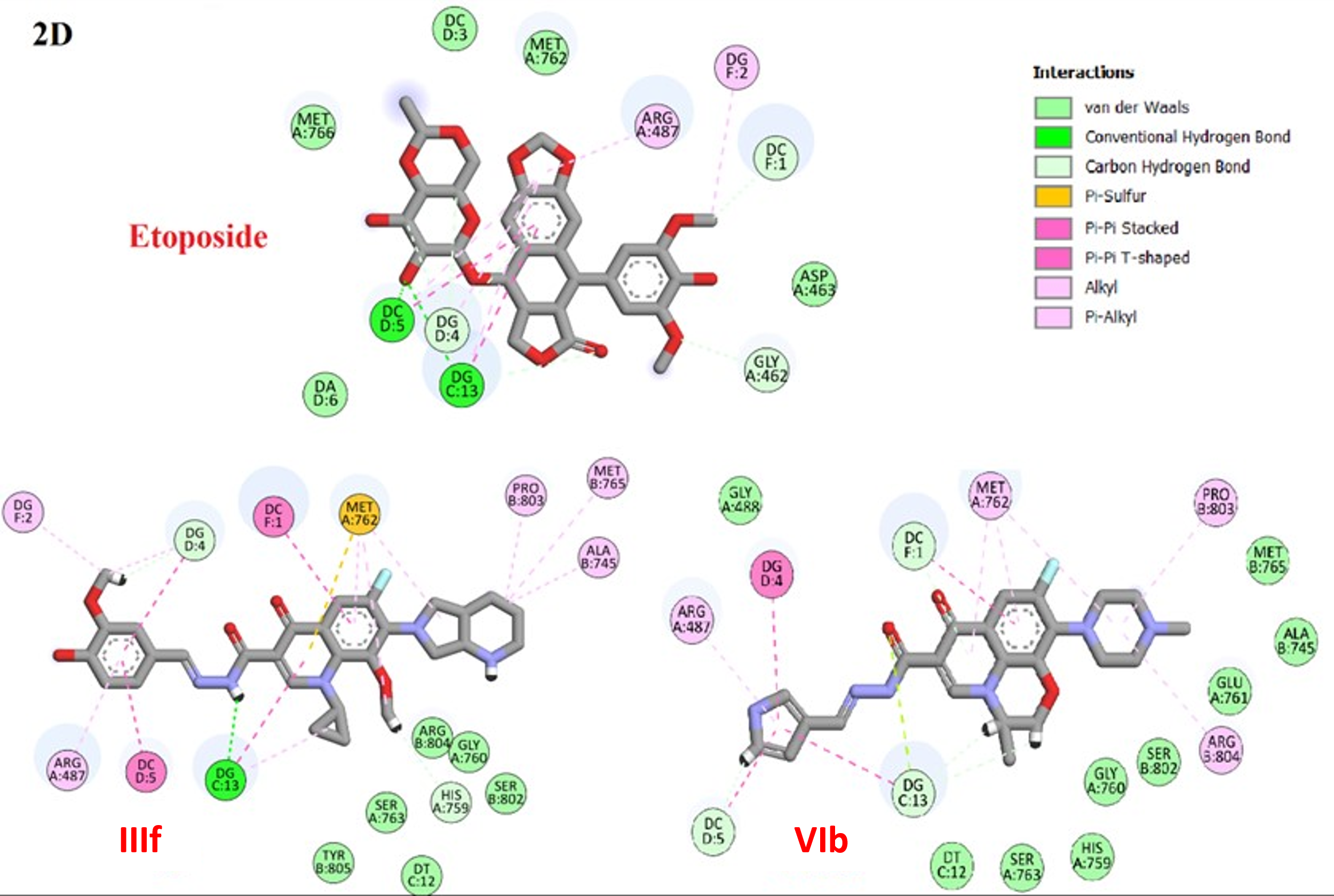


Figure 66S. 2D Interactions of **IIIf**, **VIb** and etoposide with topo II. (Dassault Systèmes. (2021). BIOVIA Discovery Studio Visualizer was used to generate this figure, https://discover.3ds.com/discovery-studio-visualizer-download )

## NCI-60 Human Tumor Cell Lines Screening single-dose assay results:


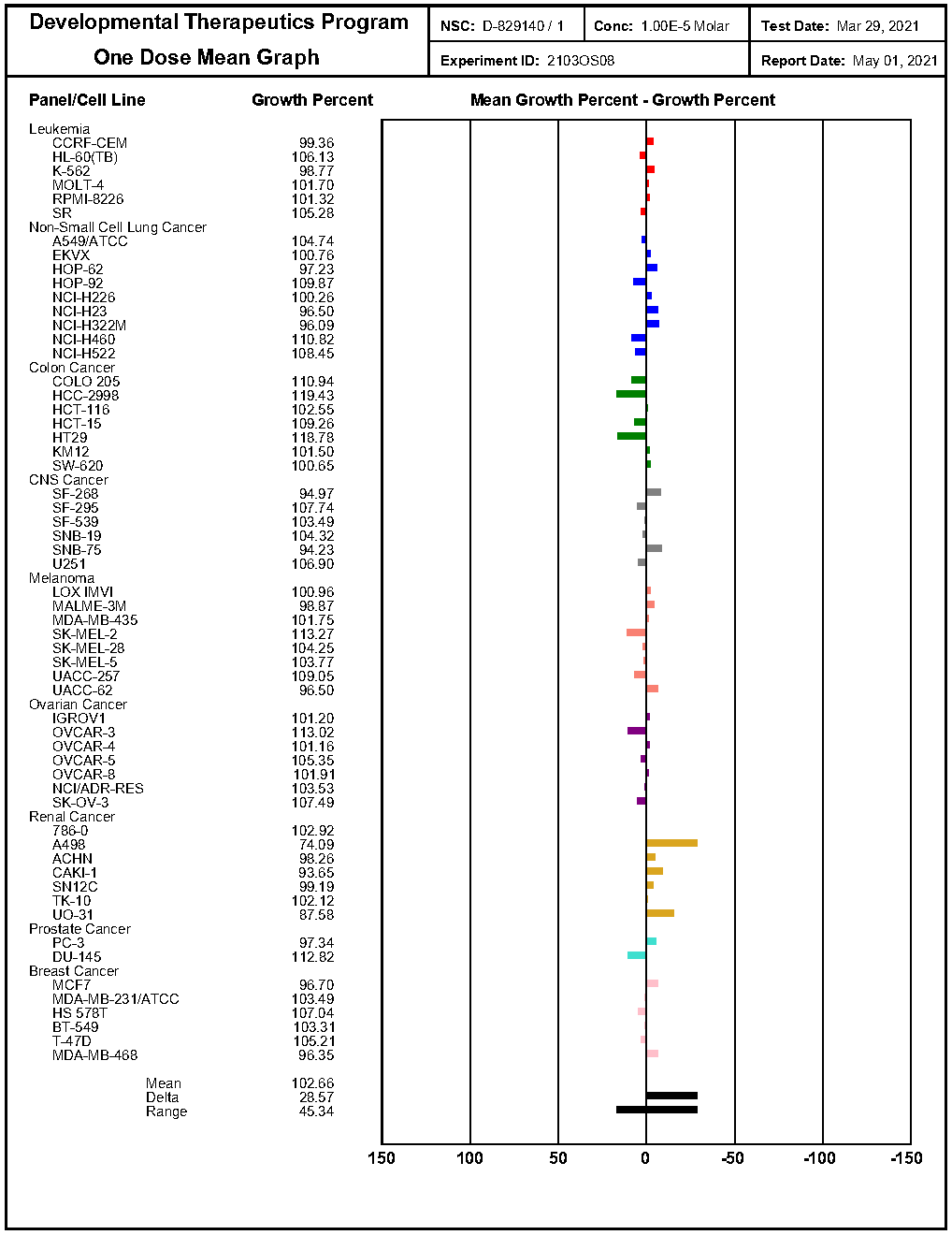


Figure 67S. NCI-60 single-dose assay result of **I**.


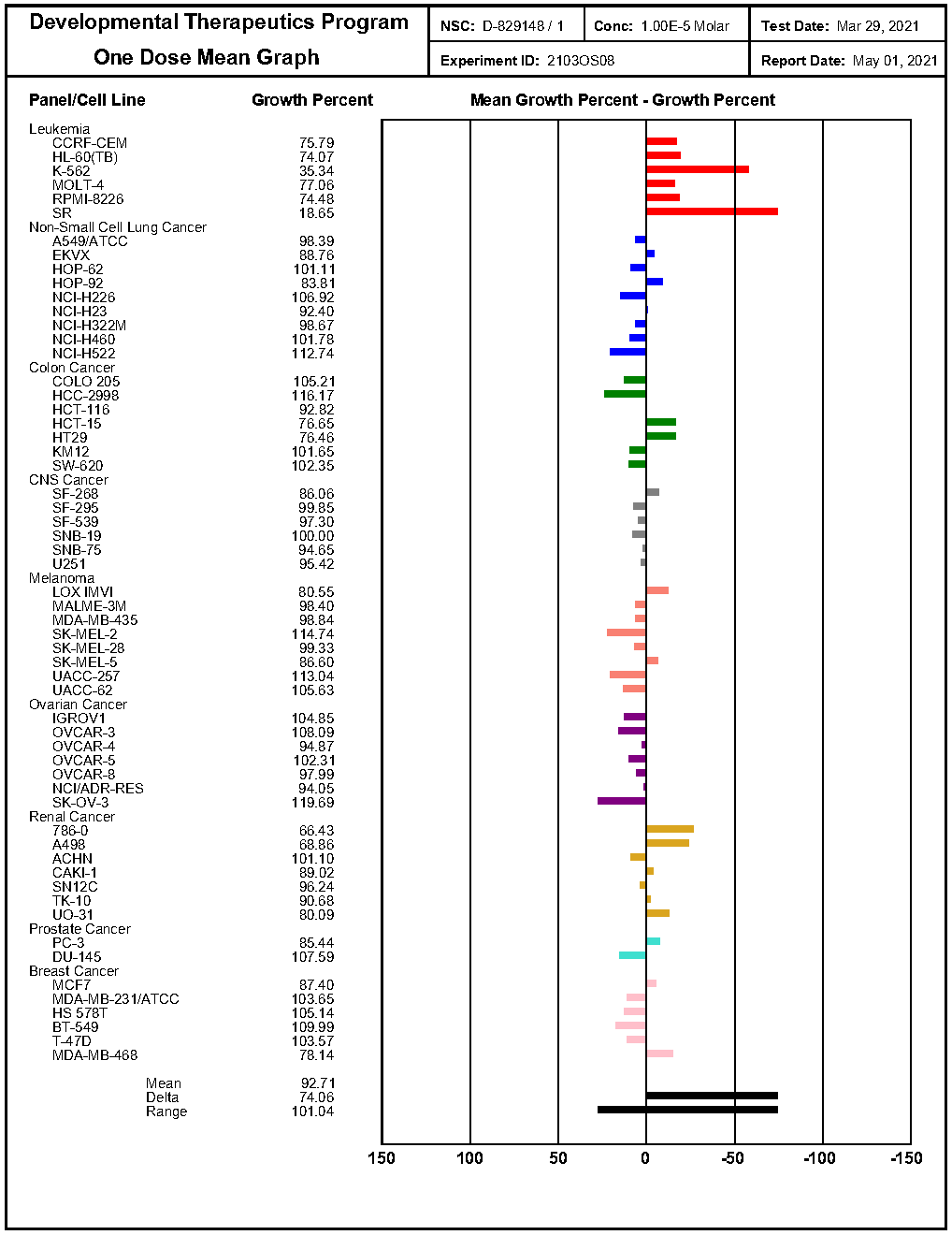


Figure 68S. NCI-60 single-dose assay result of **II**.


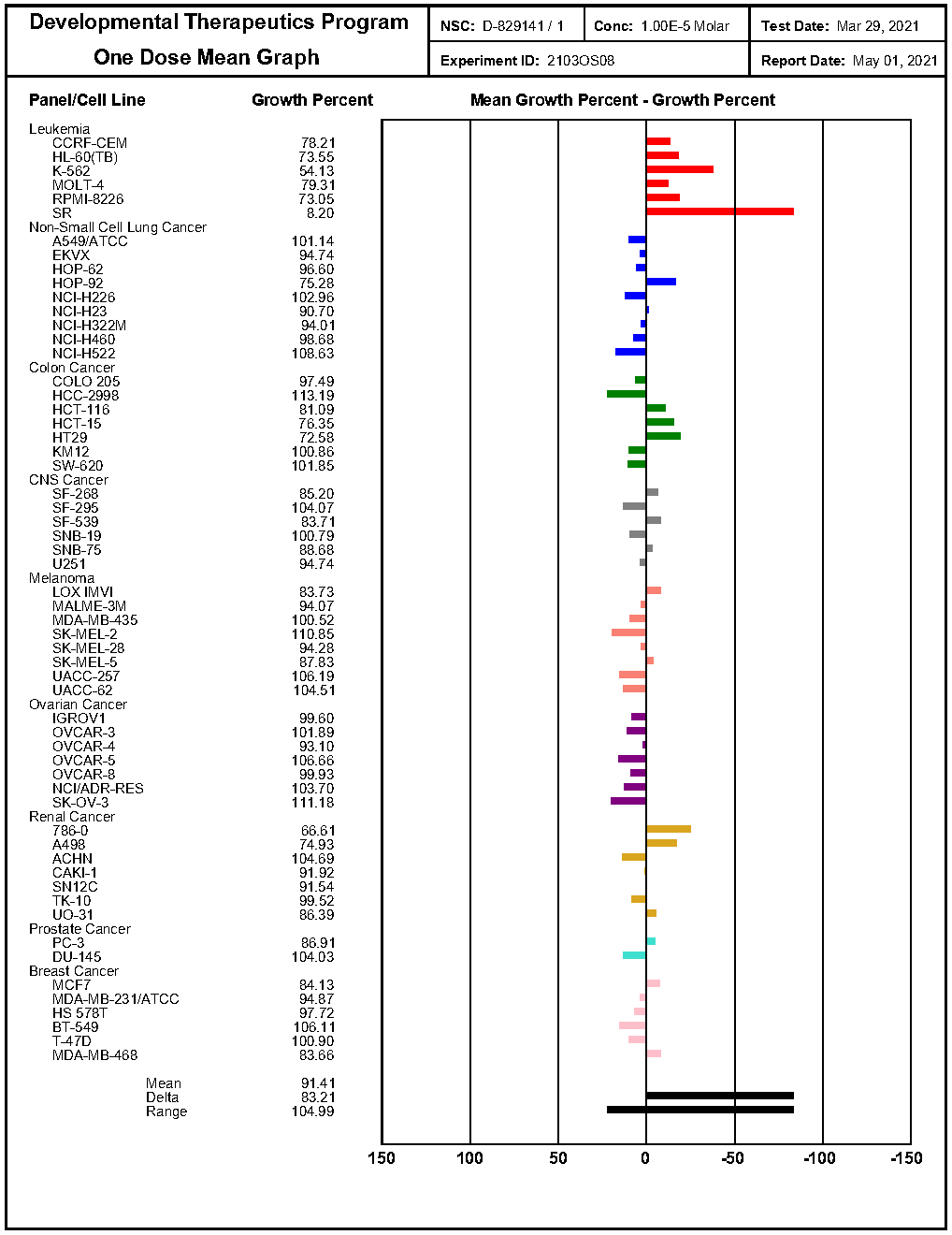


Figure 69S. NCI-60 single-dose assay result of **IIIa**.


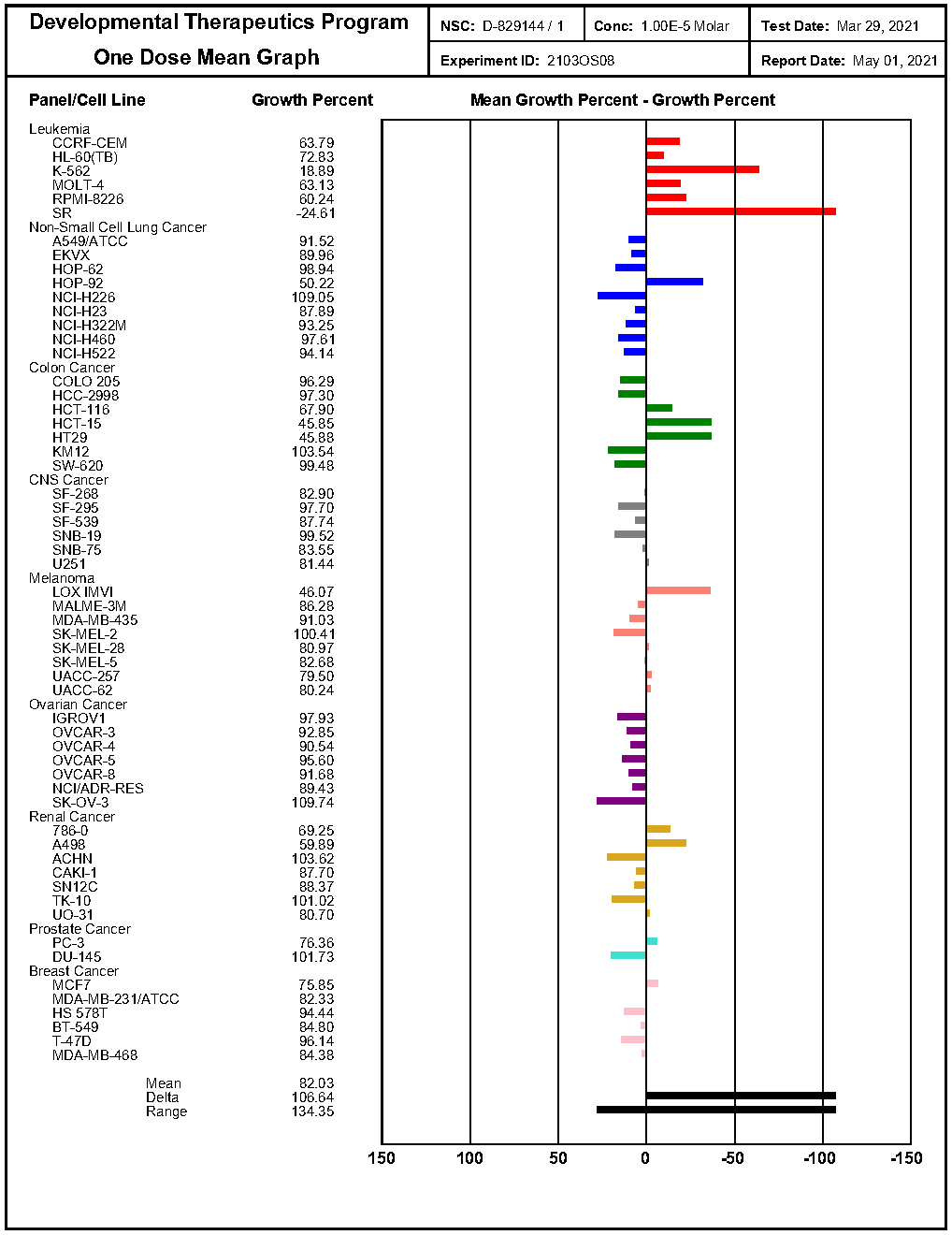


Figure 70S. NCI-60 single-dose assay result of **IIIb**.


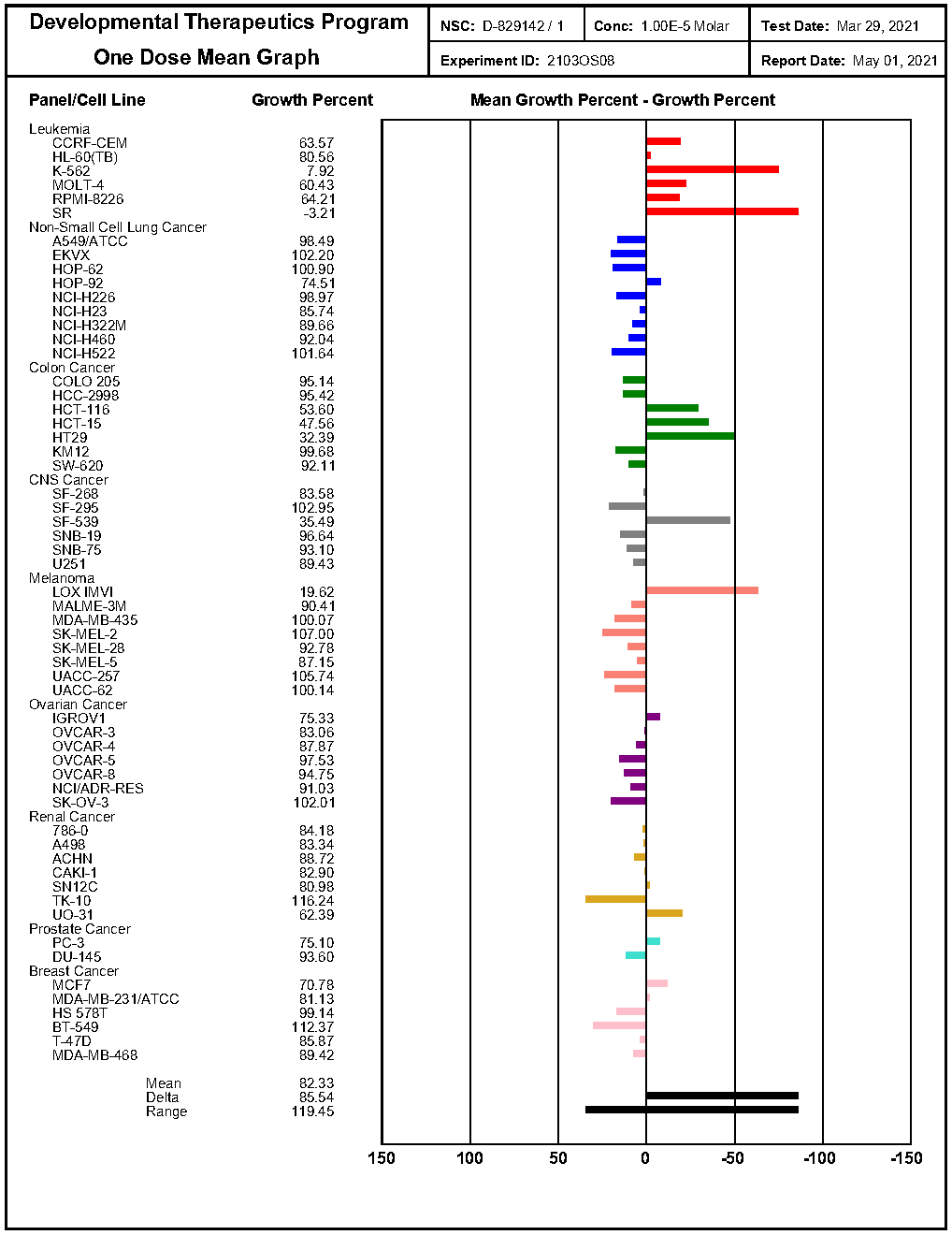


Figure 71S. NCI-60 single-dose assay result of **IIIc**.


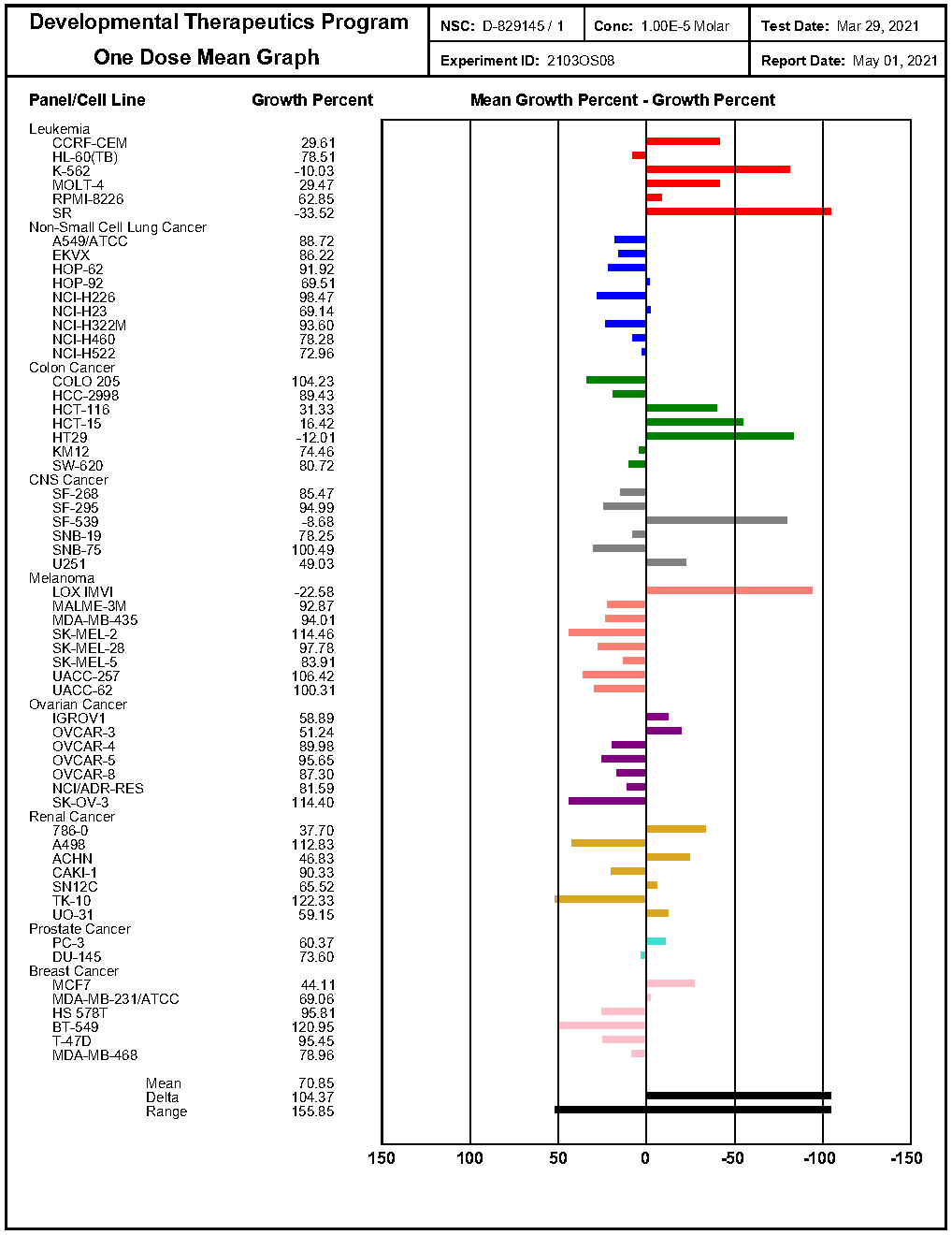


Figure 72S. NCI-60 single-dose assay result of **IIId**.


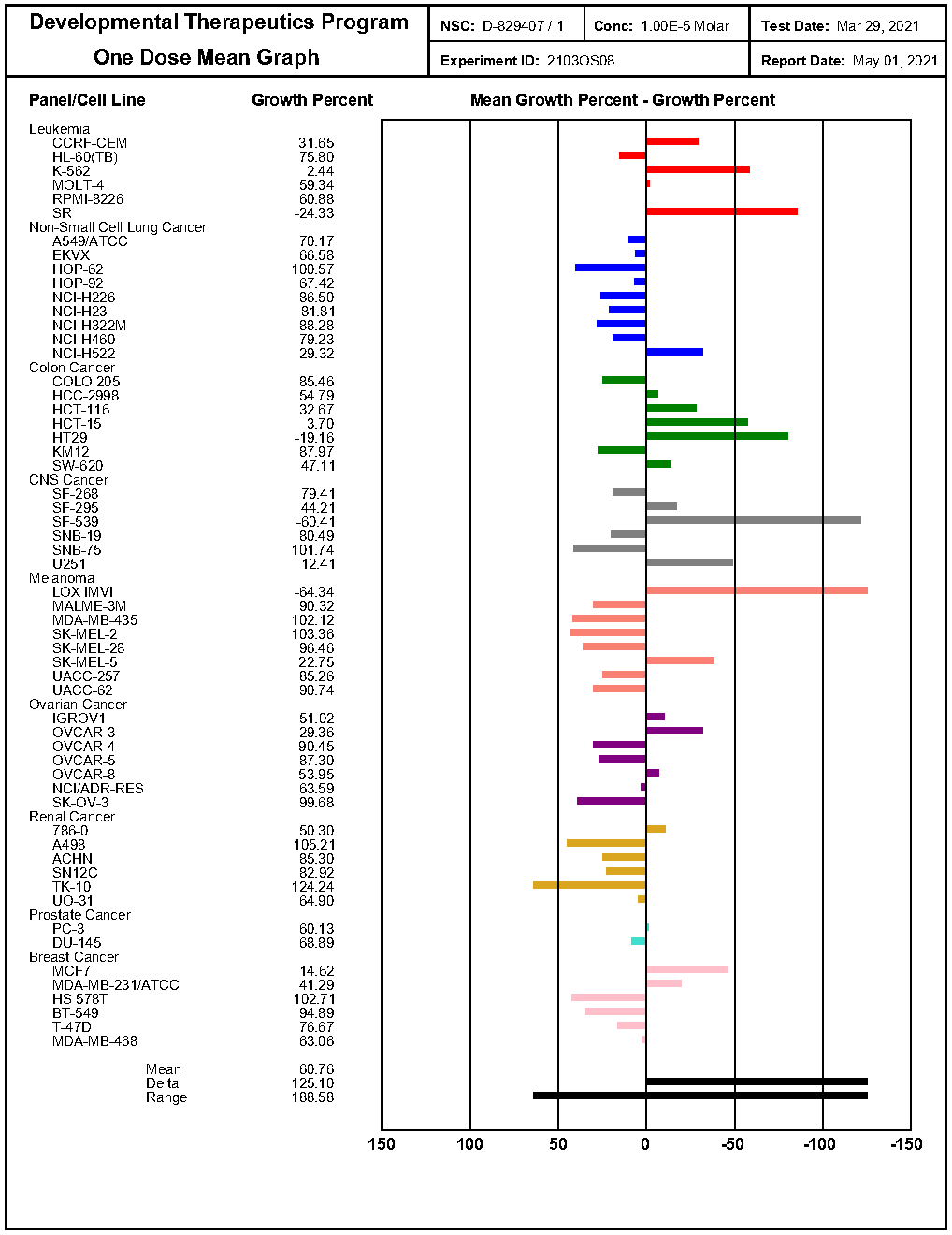


Figure 73S. NCI-60 single-dose assay result of **IIIe**.


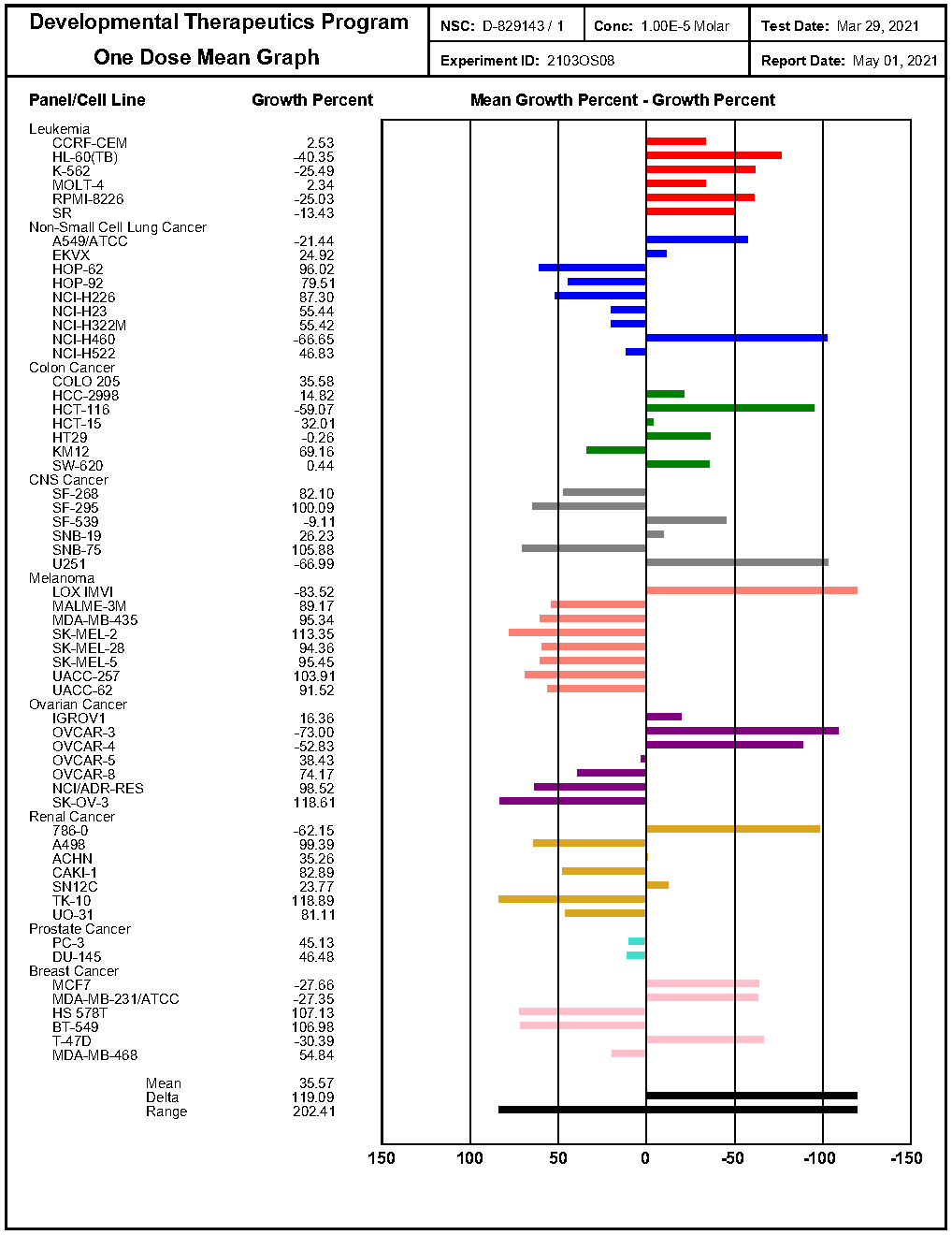


Figure 74S. NCI-60 single-dose assay result of **IIIf**.


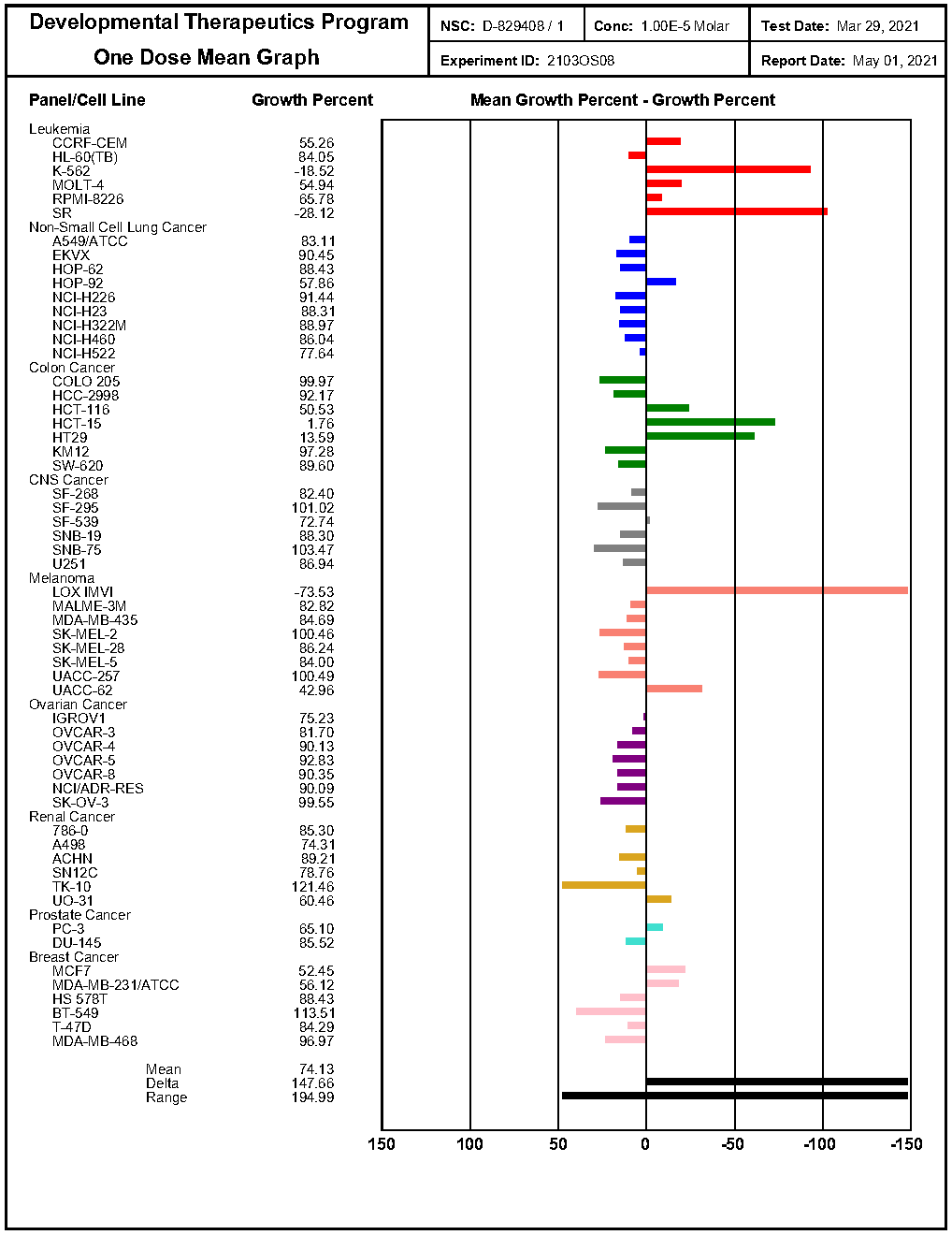


Figure 75S. NCI-60 single-dose assay result of **IIIg**.


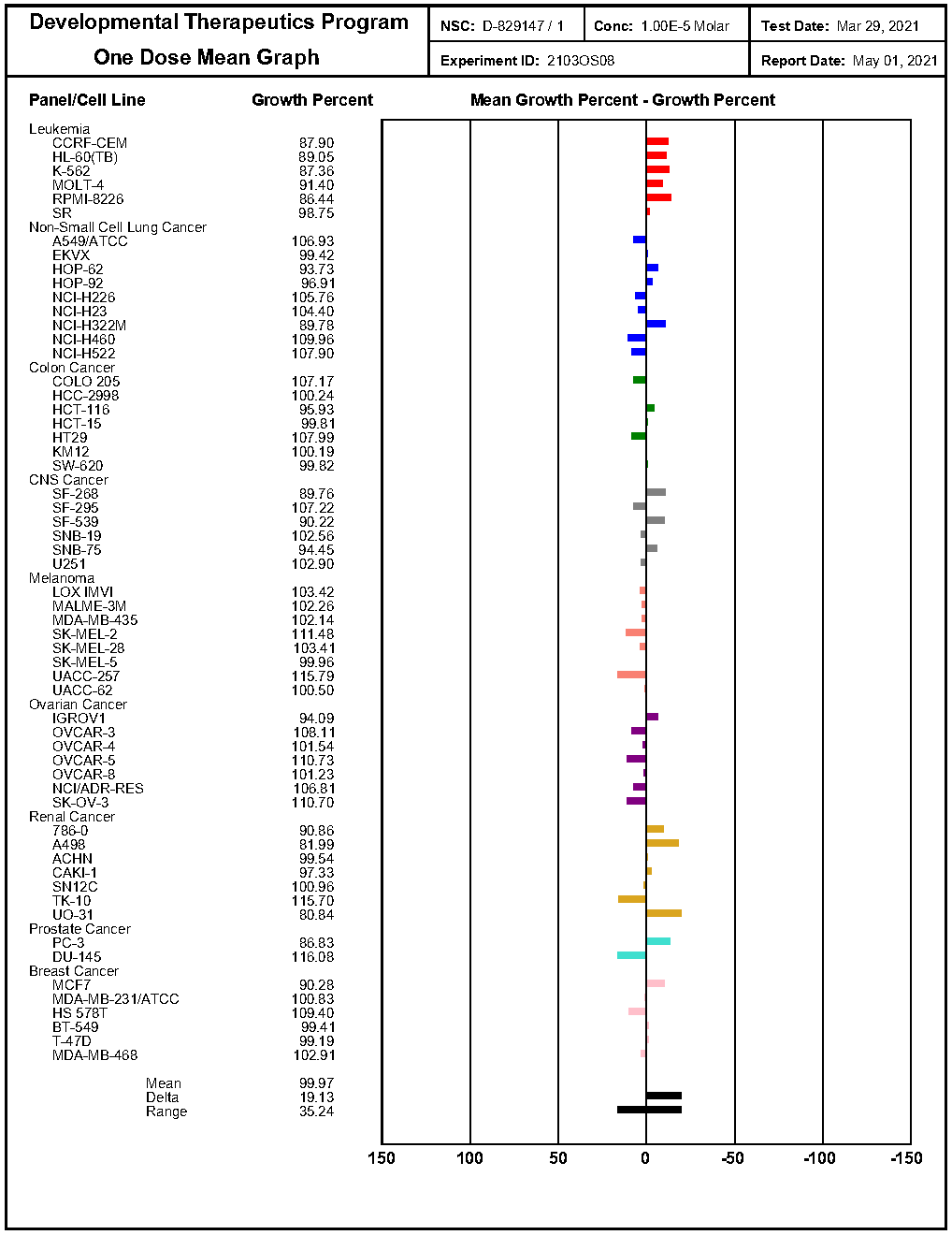


Figure 76S. NCI-60 single-dose assay result of **IIIh**.


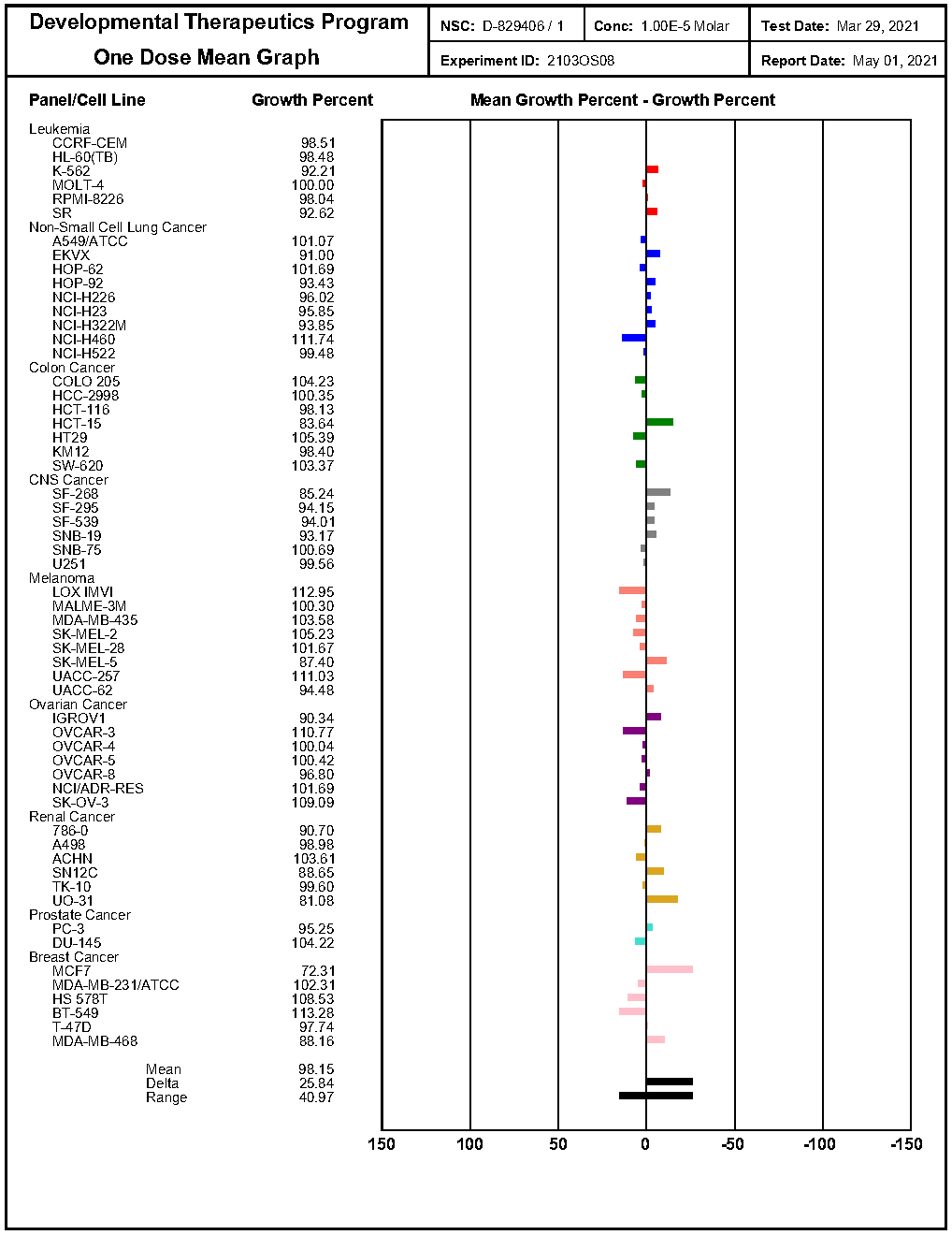


Figure 77S. NCI-60 single-dose assay result of **IIIi**.


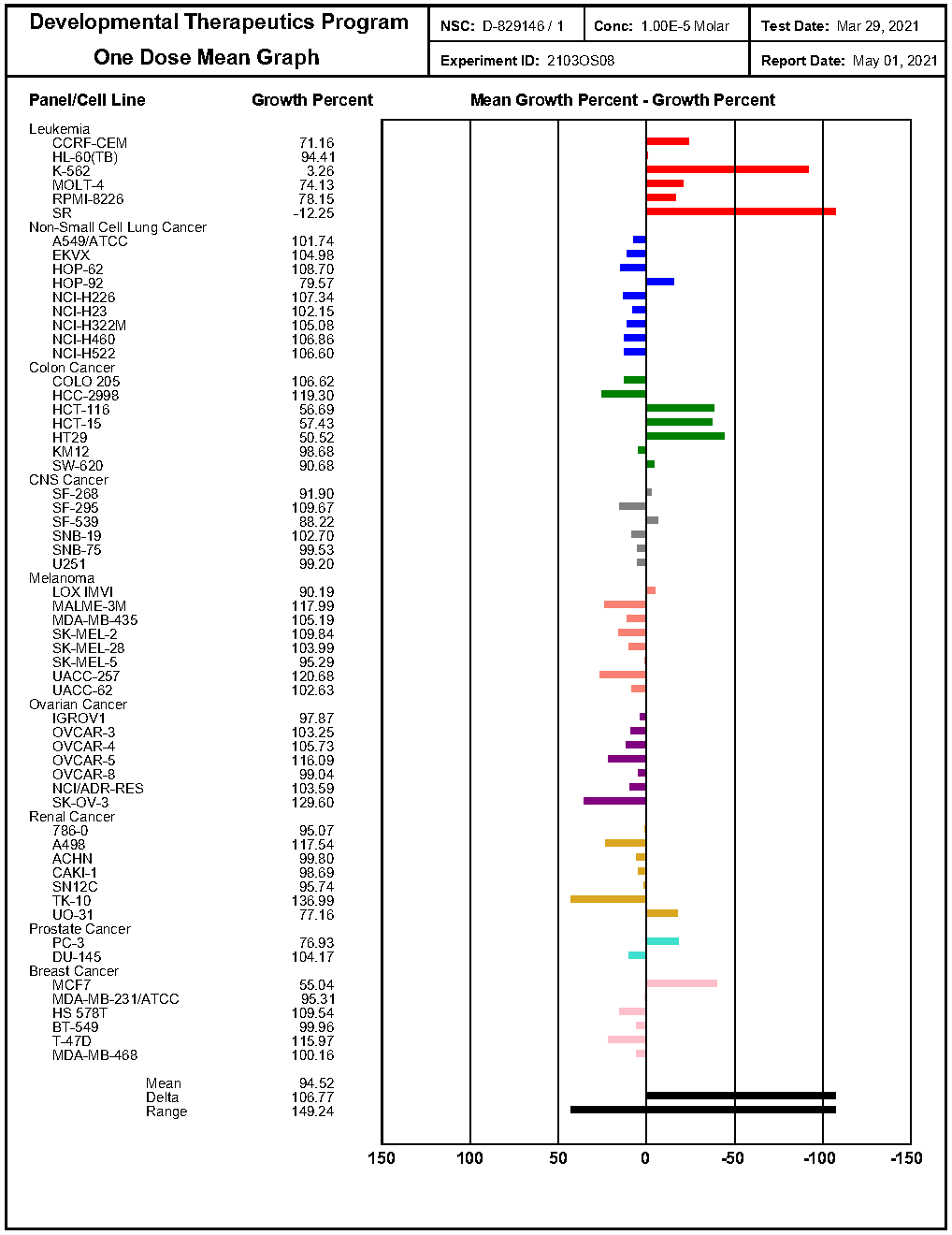


Figure 78S. NCI-60 single-dose assay result of **IIIj**.


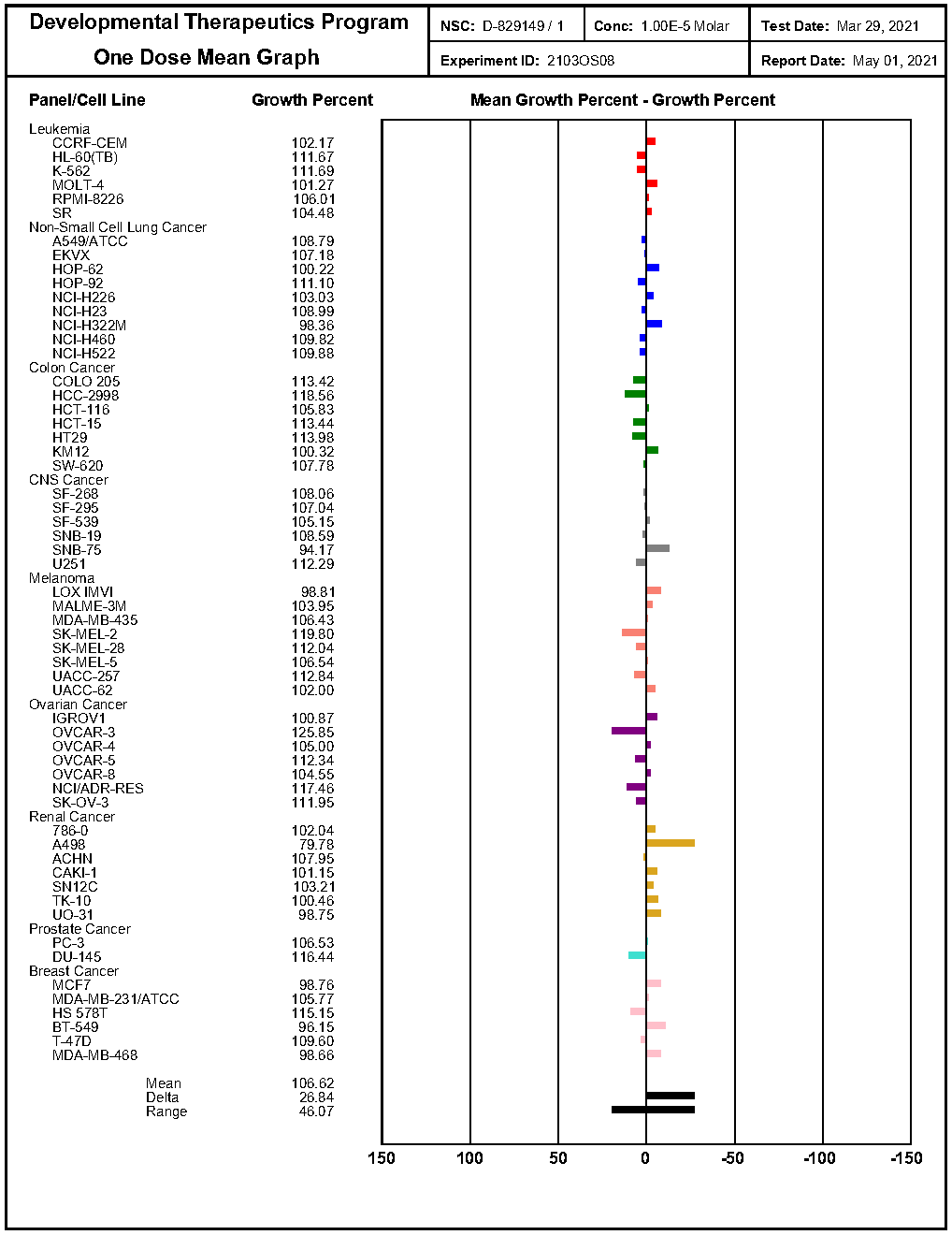


Figure 79S. NCI-60 single-dose assay result of **IV**.


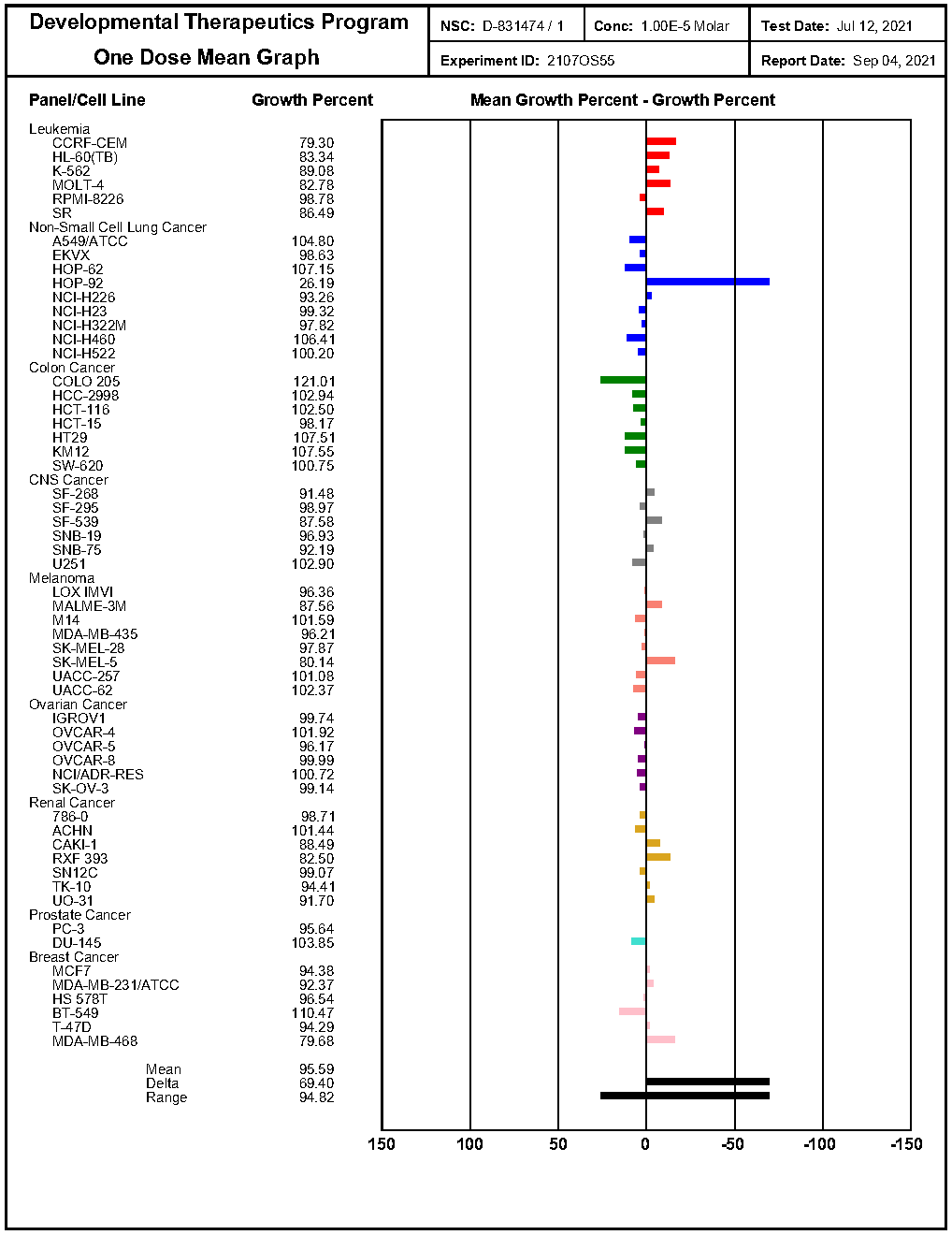


Figure 80S. NCI-60 single-dose assay result of **VIa**.


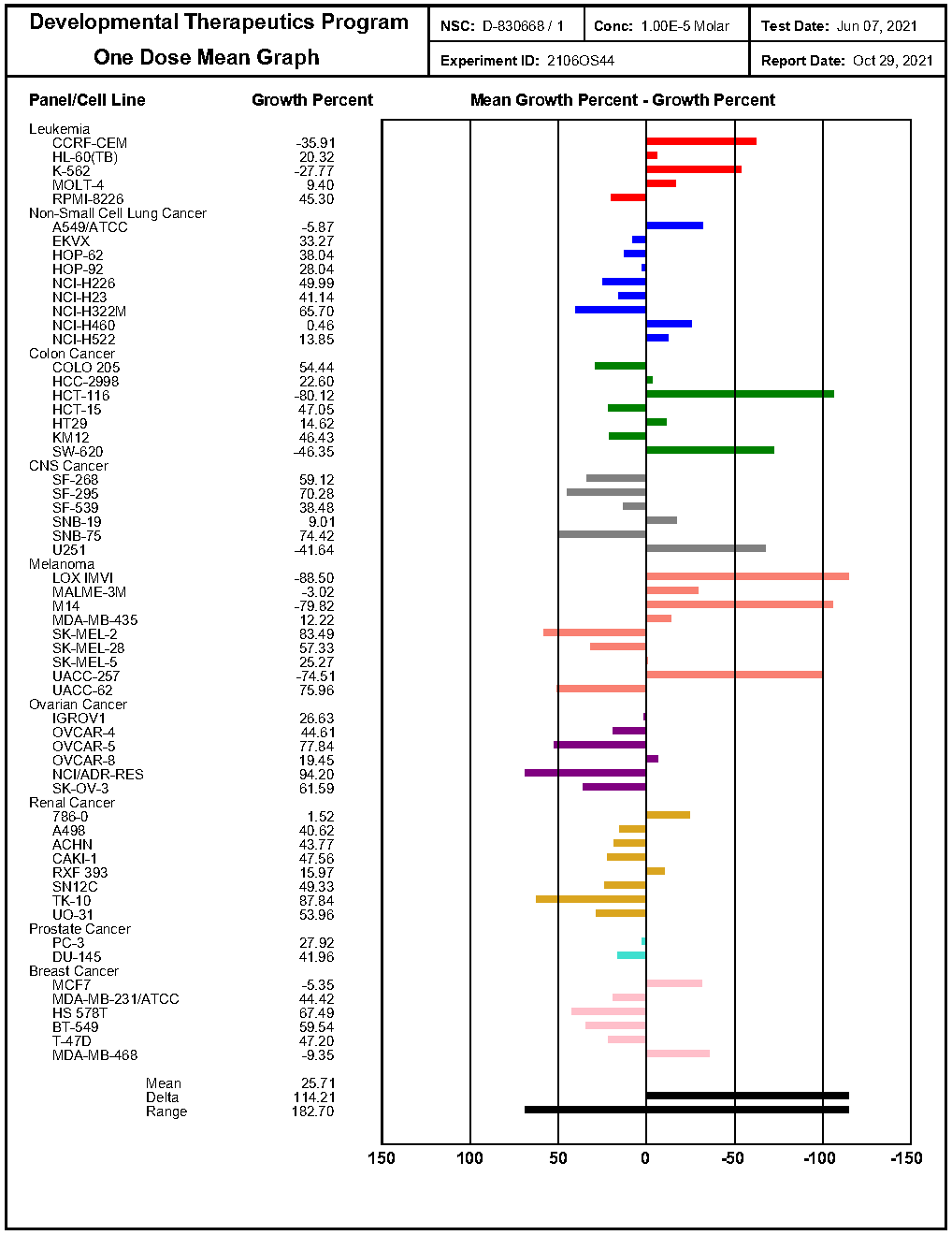


Figure 81S. NCI-60 single-dose assay result of **VIb**.


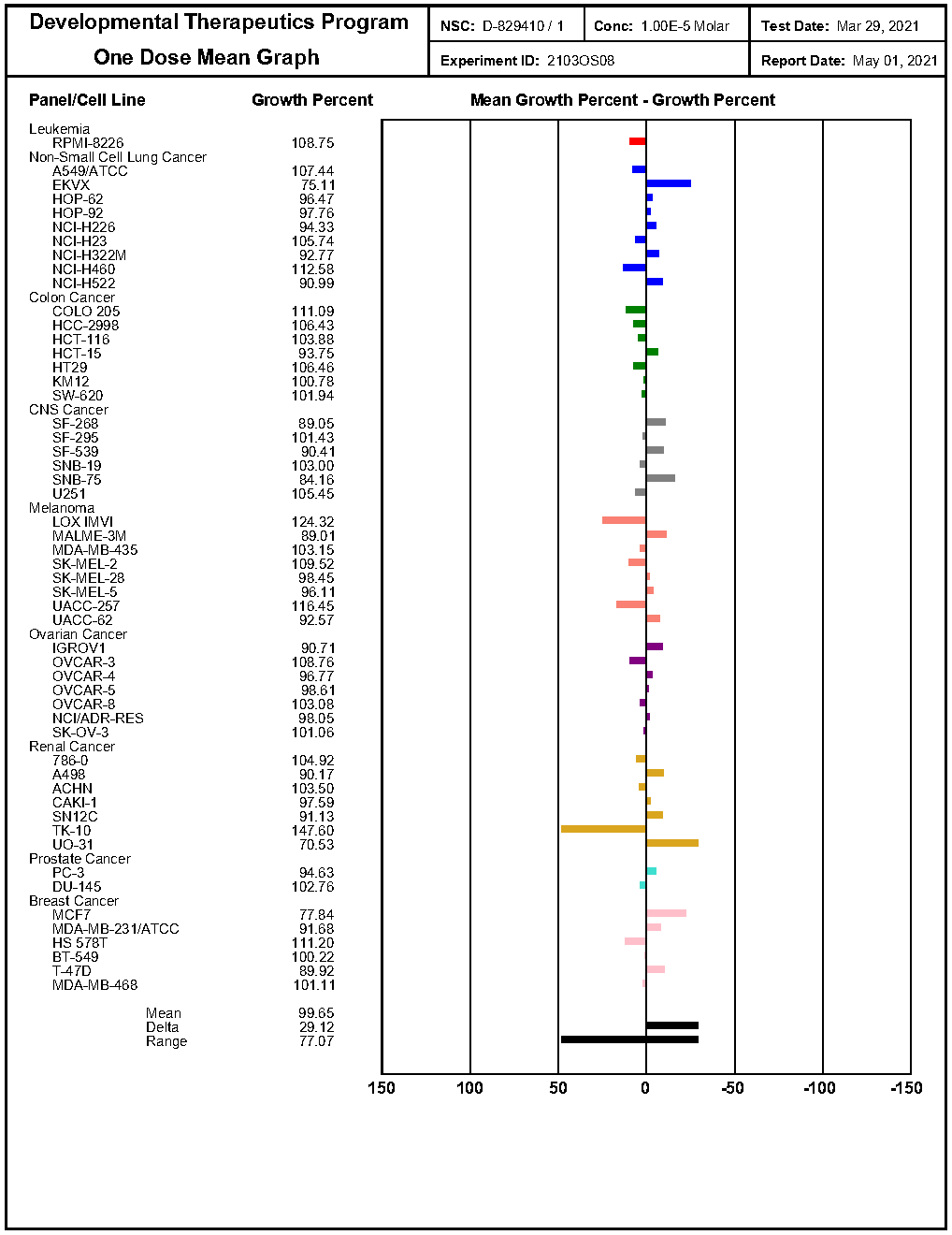


Figure 82S. NCI-60 single-dose assay result of **VIc**.


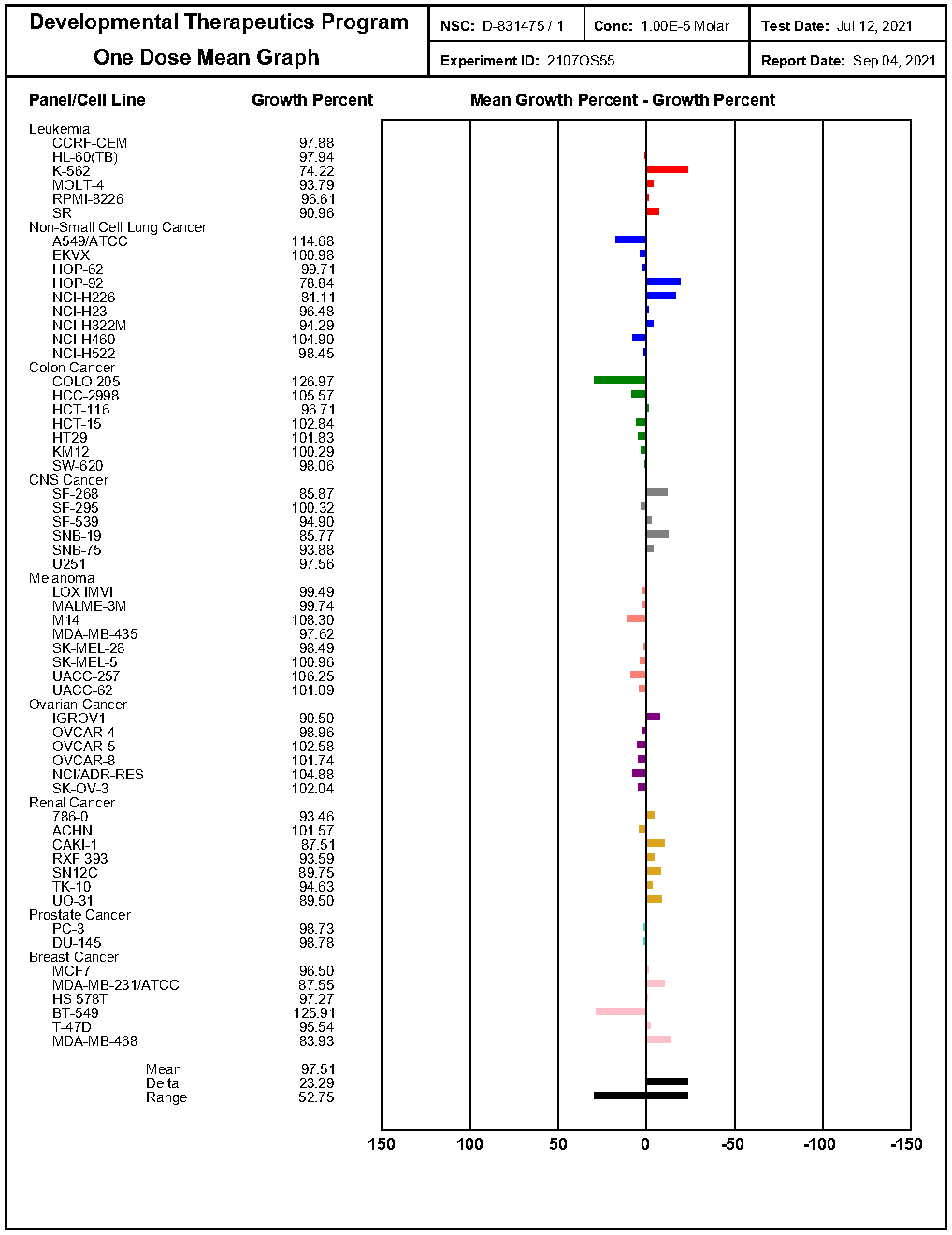


Figure 83S. NCI-60 single-dose assay result of **VIIIa**.


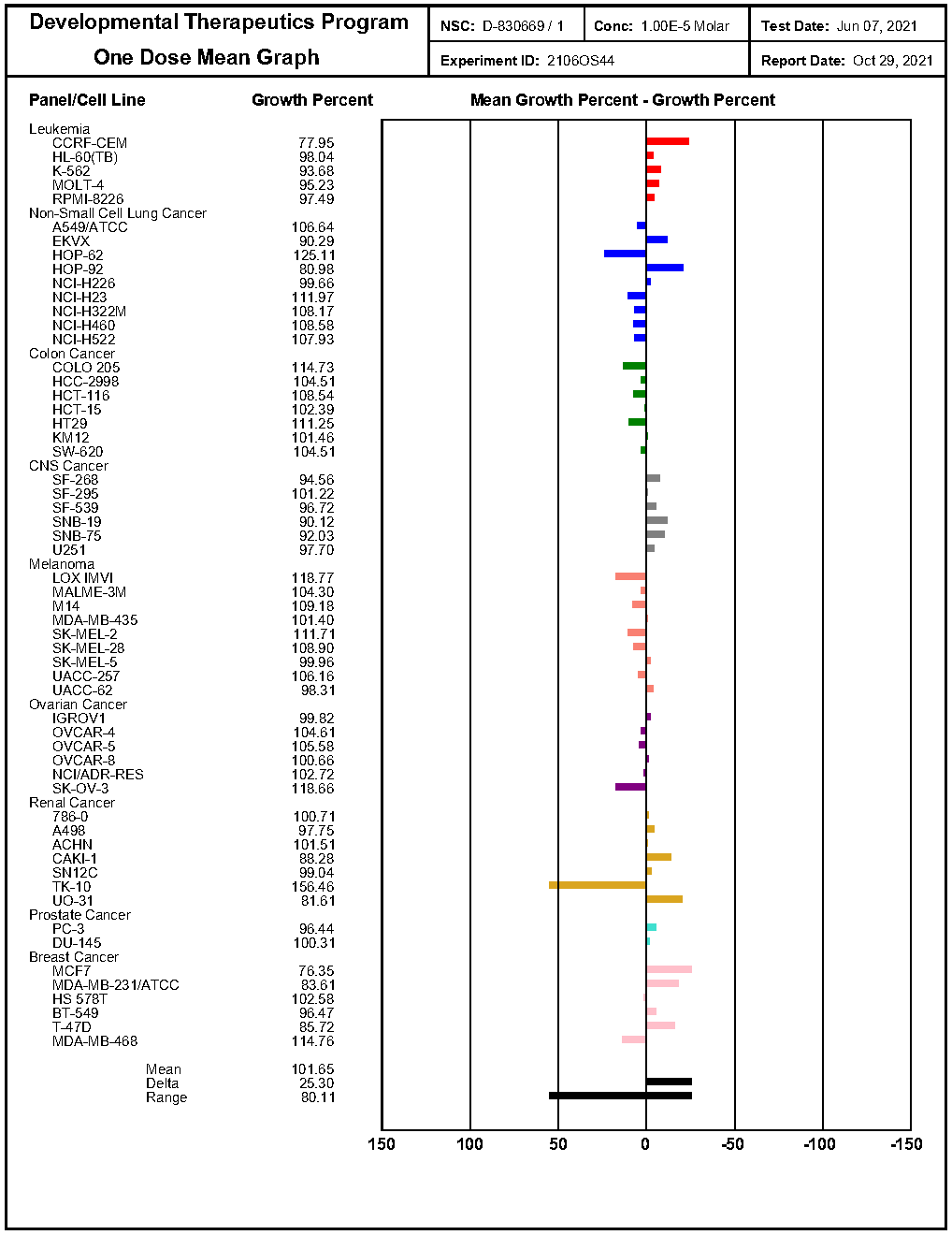


Figure 84S. NCI-60 single-dose assay result of **VIIIb**.


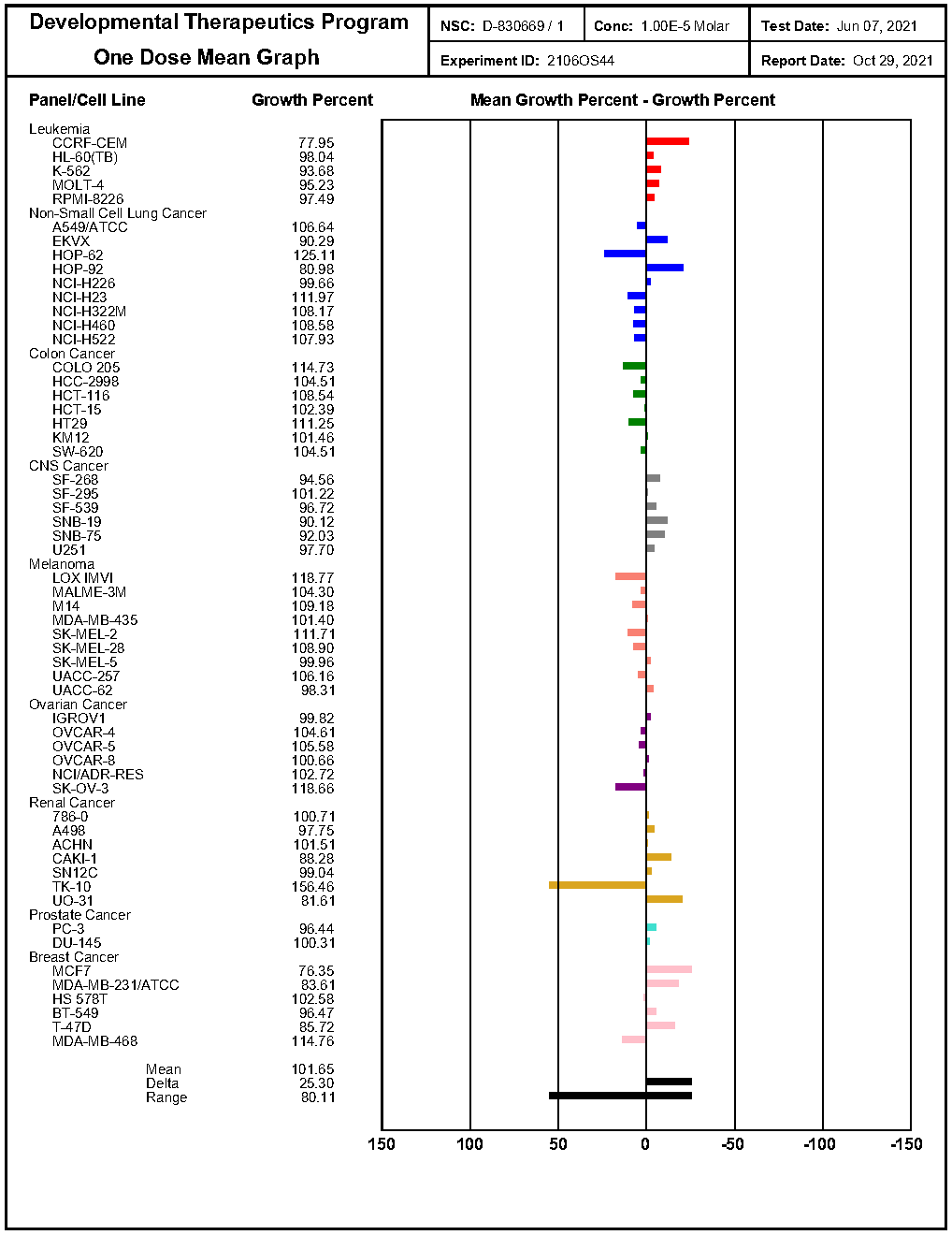


Figure 85S. NCI-60 single-dose assay result of **VIIIc**.

## NCI-60 Human Tumor Cell Lines Screening five-dose assay results:


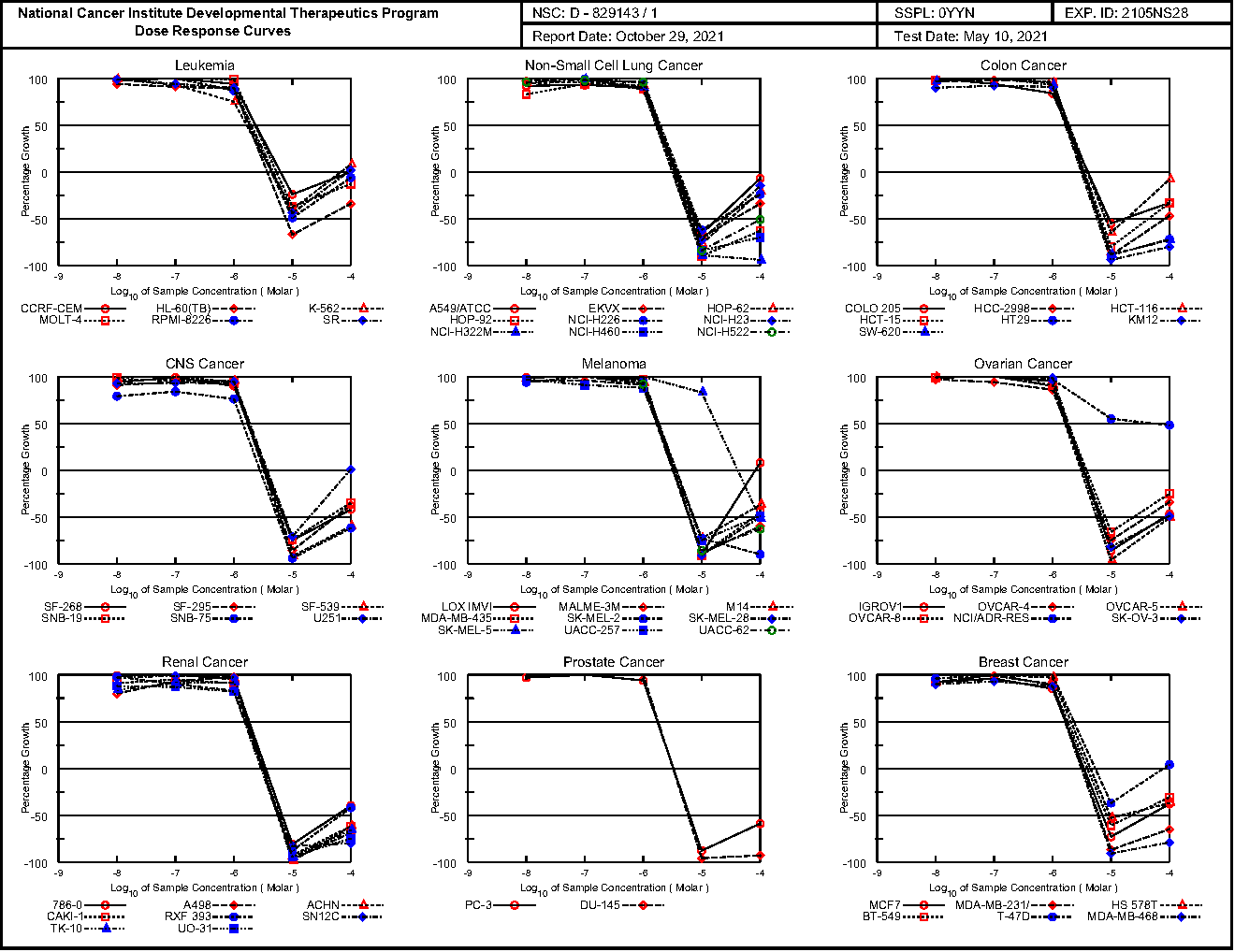


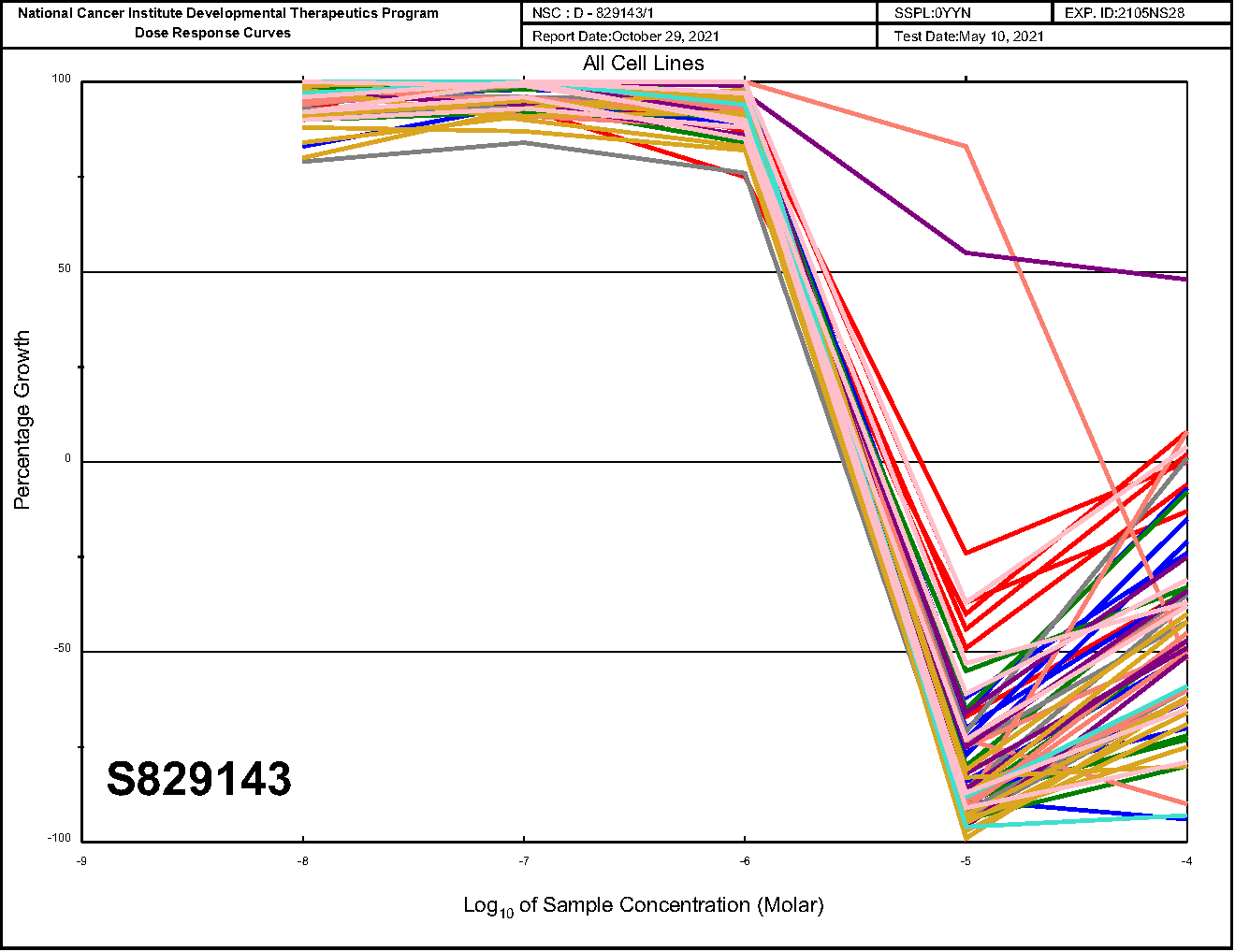


Figure 86S. NCI-60 five-dose assay result of **IIIf** (dose-response curves).


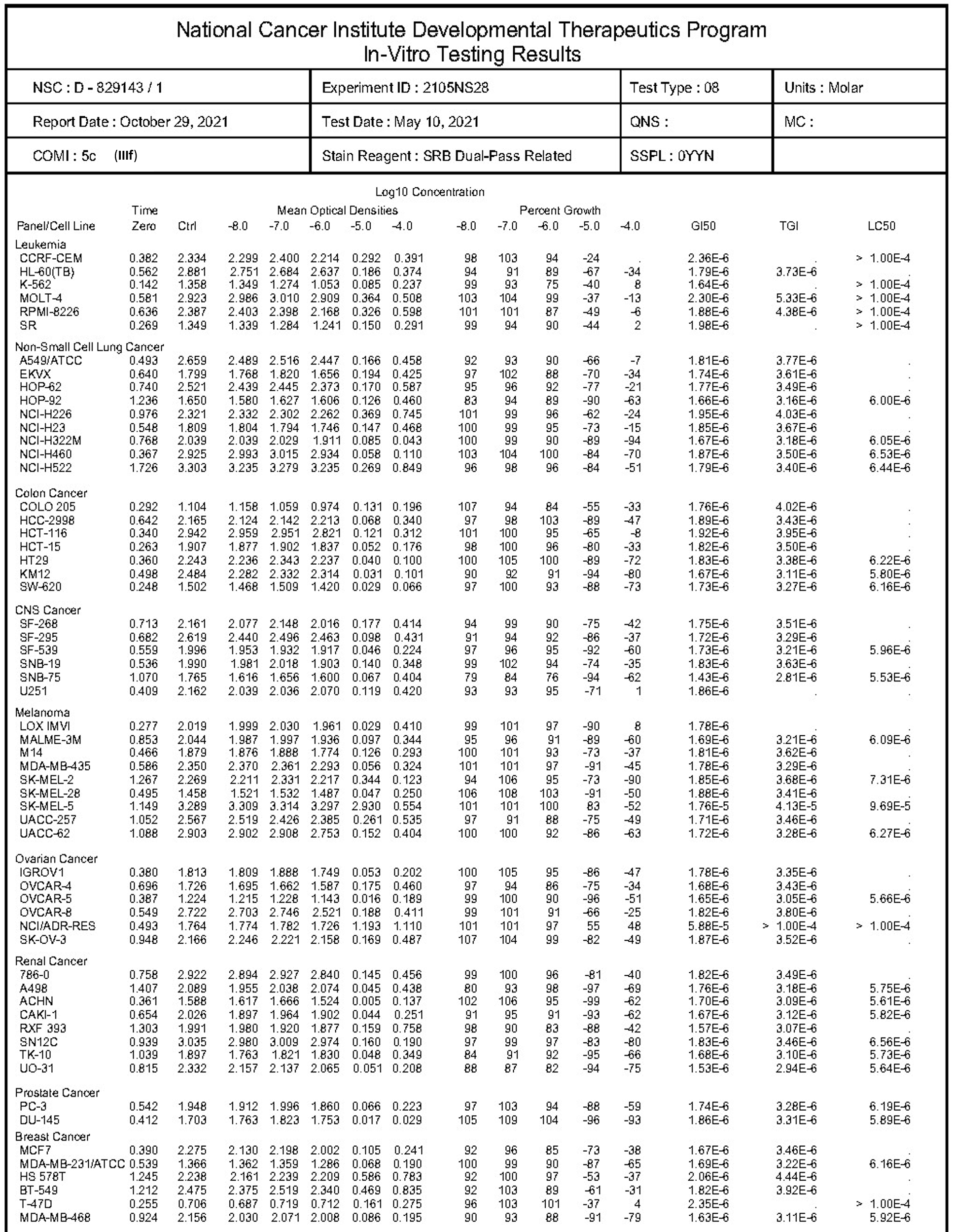


Figure 87S. NCI-60 five-dose assay result of **IIIf**.


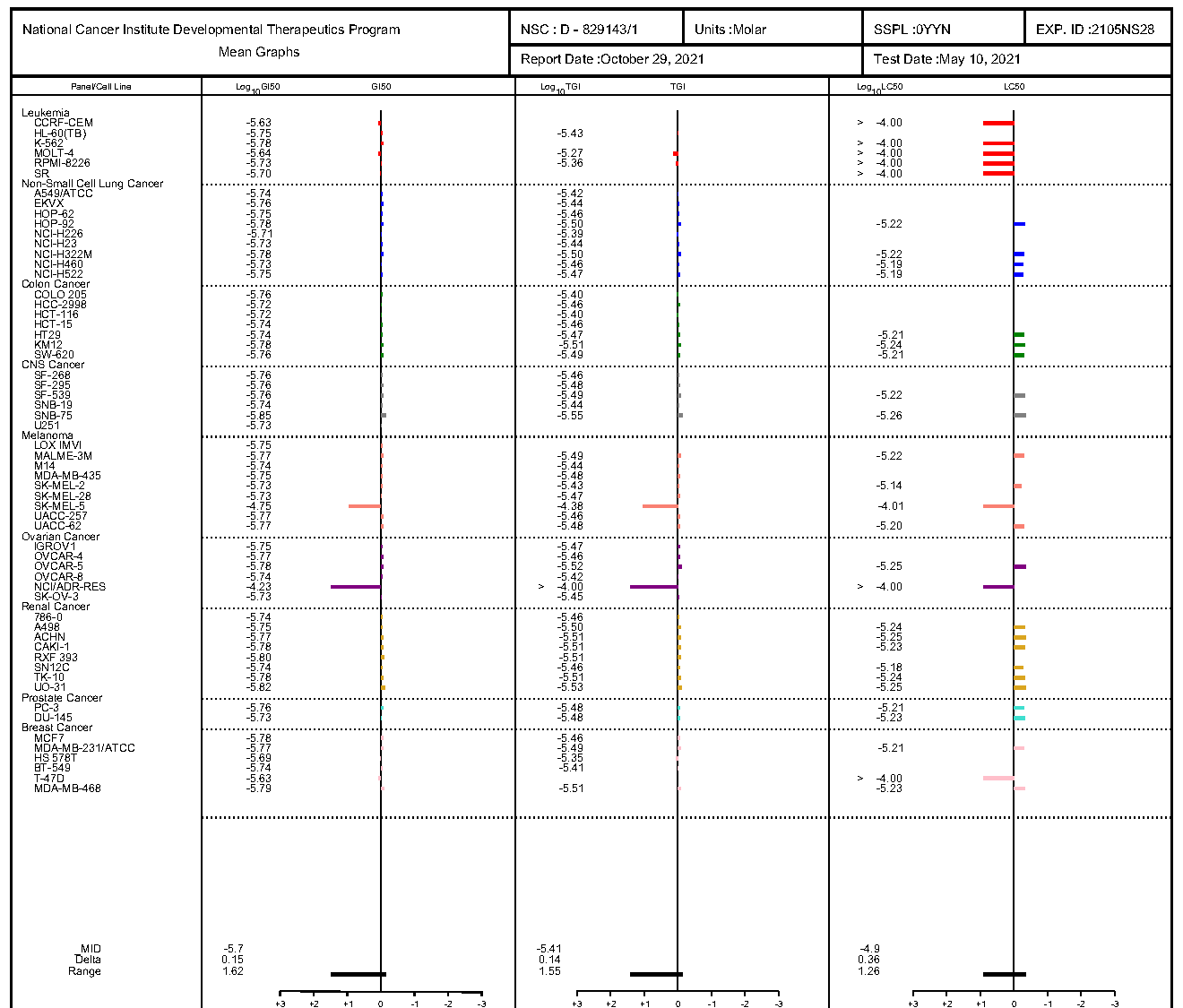


Figure 88S. NCI-60 five-dose assay result of **IIIf** (GI_50_, TGI and LC_50_).


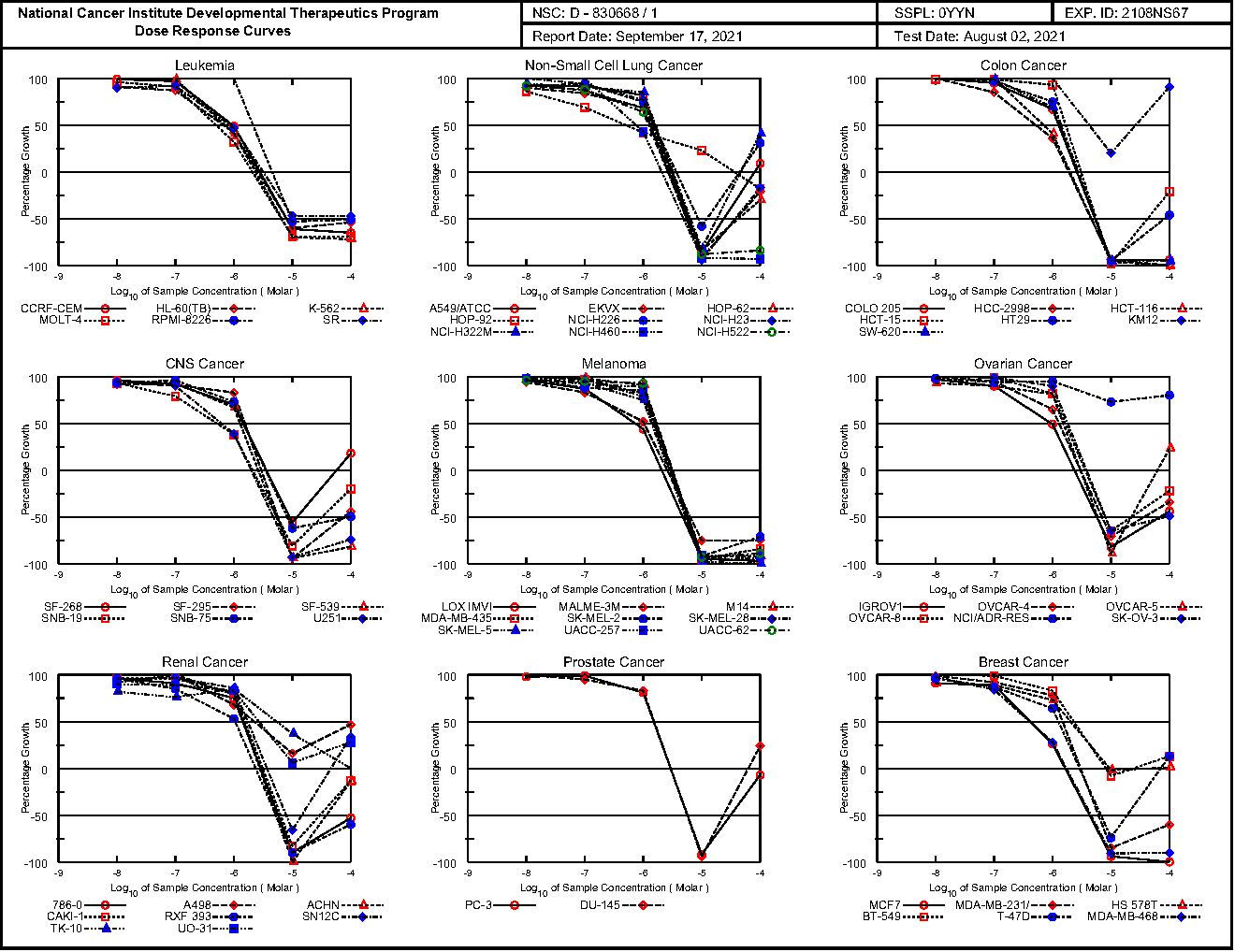

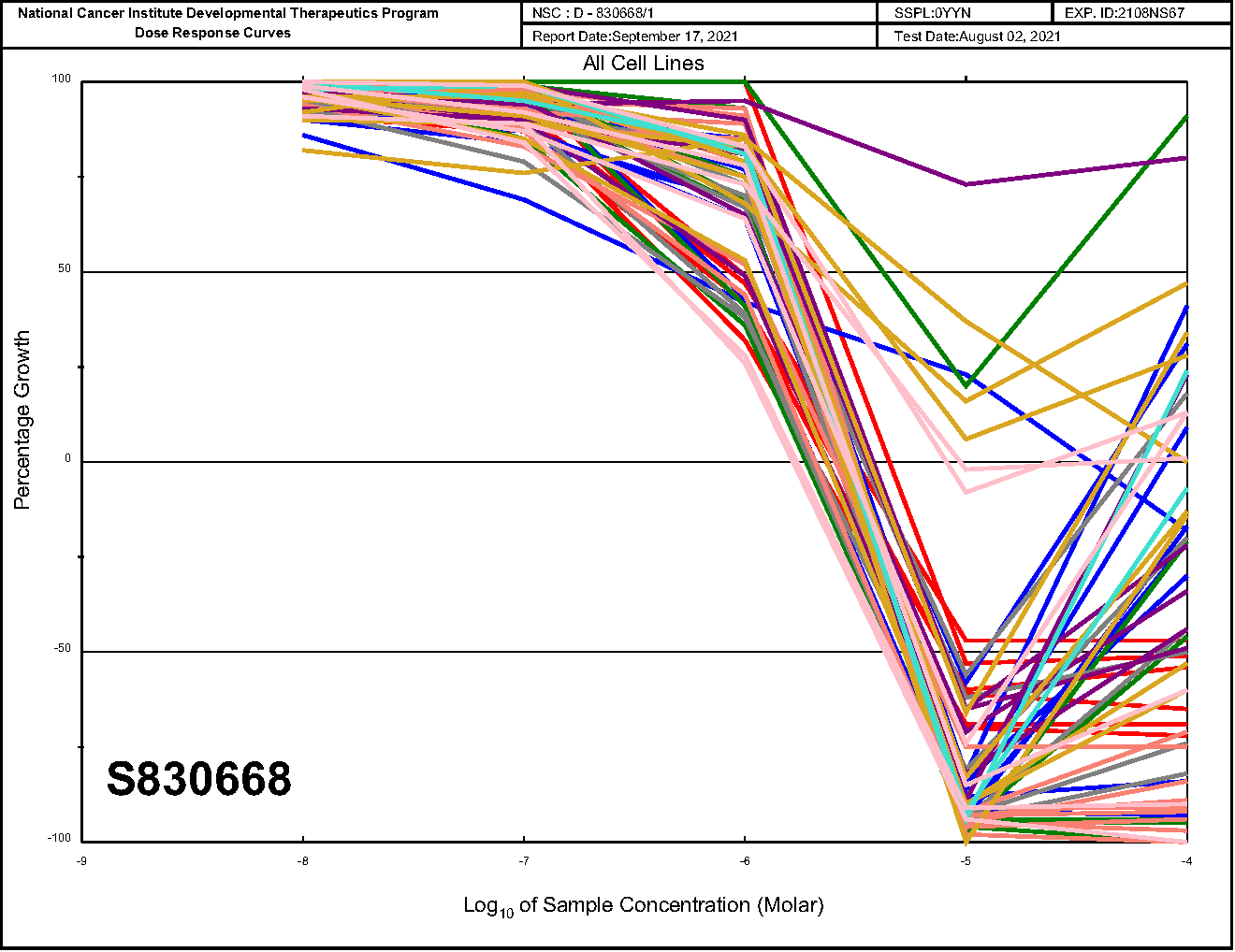


Figure 89S. NCI-60 five-dose assay result of **VIb** (dose-response curves).


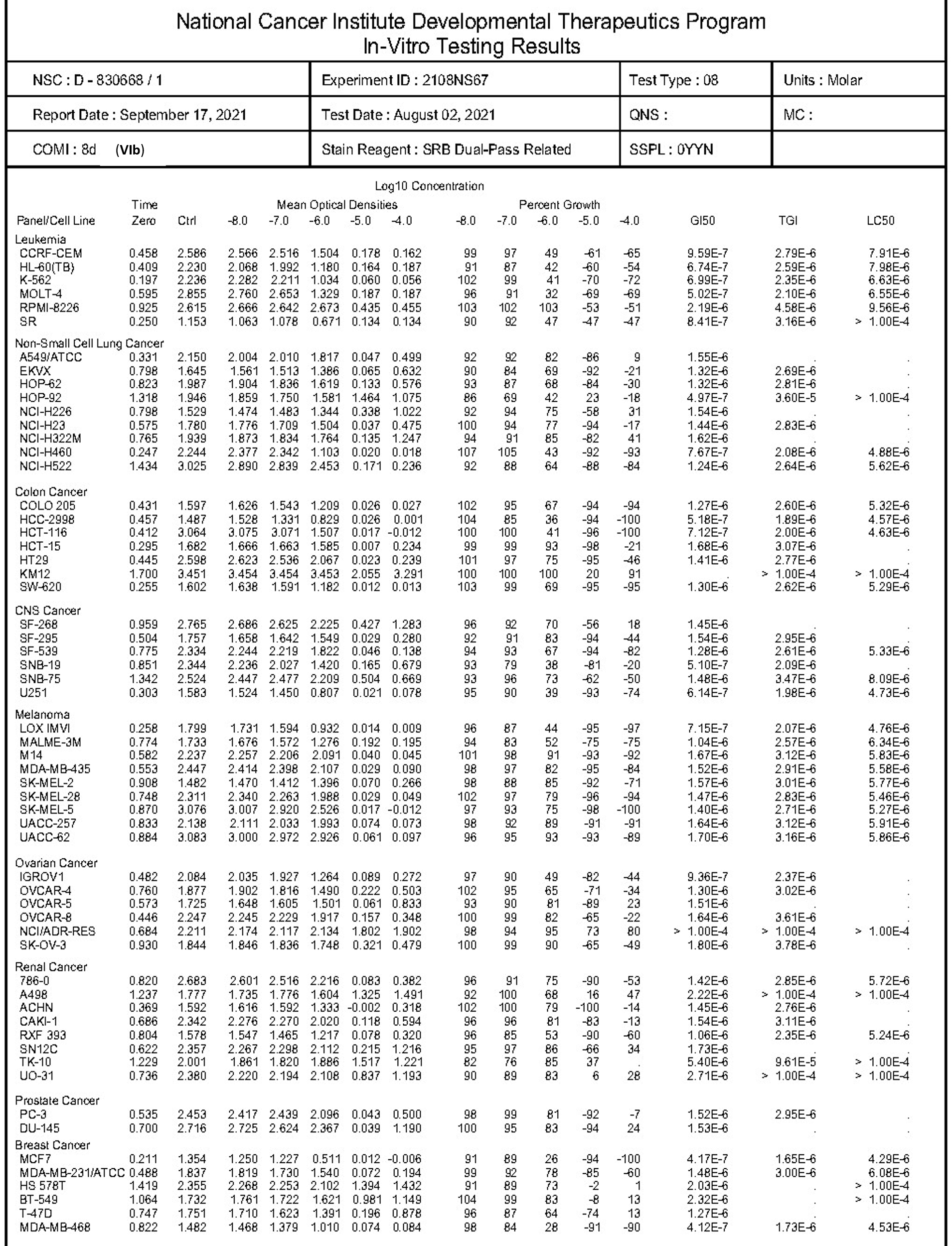


Figure 90S. NCI-60 five-dose assay result of **VIb**.


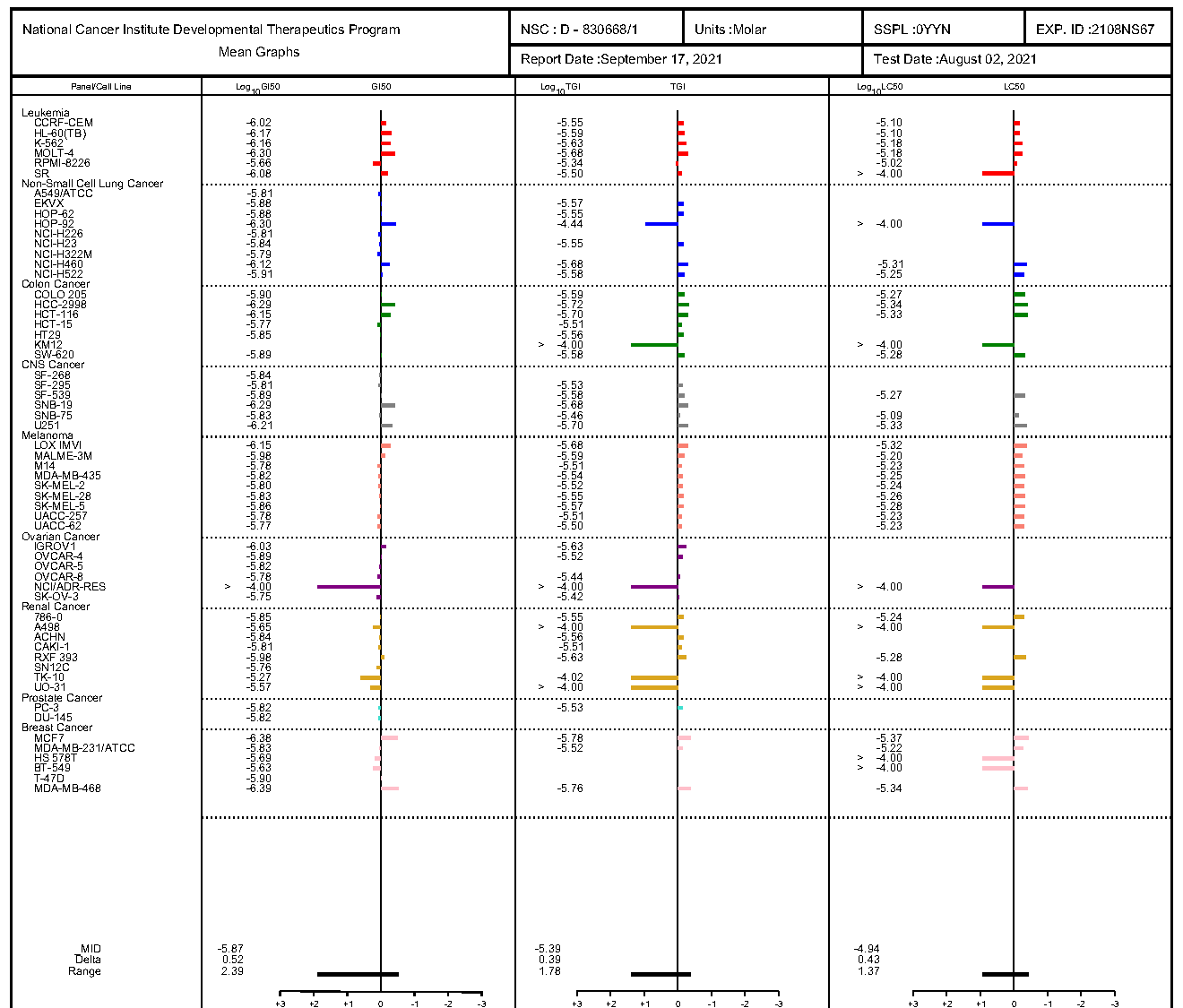


Figure 91S. NCI-60 five-dose assay result of **VIb** (GI_50_, TGI and LC_50_) .

**Topo I**

**DNA**

**Camptothecin**

**IIIf 50 µM**

**IIIf 100 µM**

**VIb 50 µM**

**VIb 100 µM**


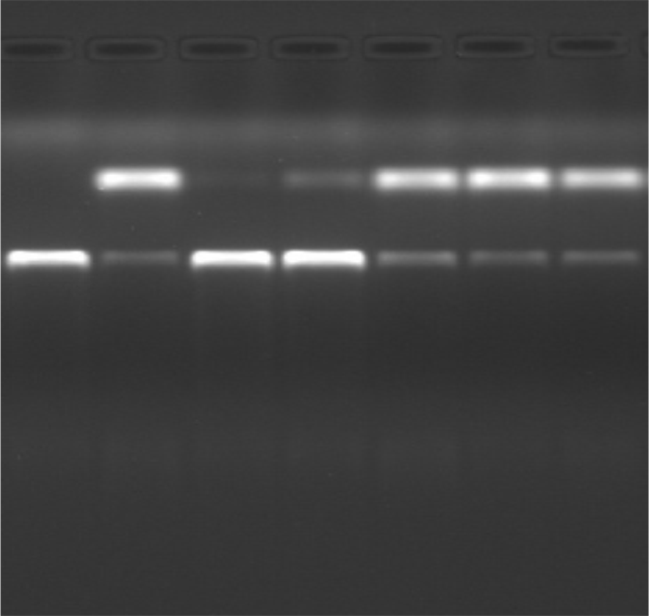


Figure 92S. Original gel for recombinant Topo I of (**IIIf**) and (**VIb**).

**Etoposide**

**VIb 50 µM**

**VIb 100 µM**

**IIIf 50 µM**

**IIIf 100 µM**

**Topo II**

**DNA**


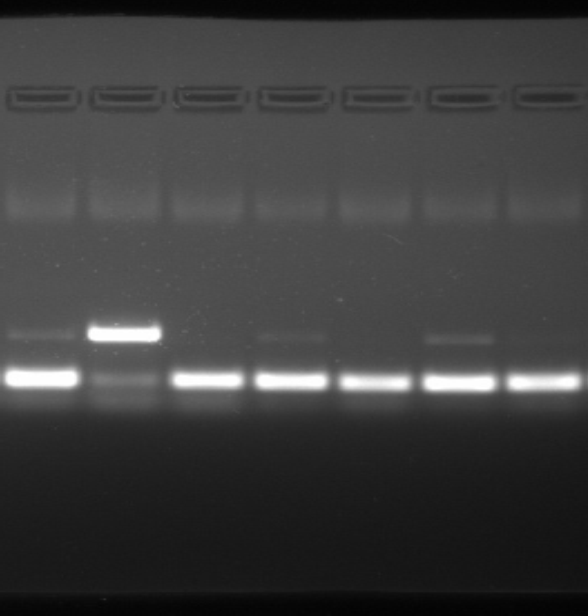


Figure 93S. Original gel for recombinant Topo II of (**IIIf**) and (**VIb**).
